# Fluorescence Quenching Properties and Bioimaging Applications of Readily Accessible Blue to Far-Red Fluorogenic Triazinium Salts

**DOI:** 10.1021/jacs.5c17428

**Published:** 2025-12-28

**Authors:** Veronika Šlachtová, Daniel Bím, Krisztina Németh, Eliza Barańska, Simona Bellová, Martin Dračínský, Rastislav Dzijak, Péter Kele, Tomáš Slanina, Milan Vrabel

**Affiliations:** † Institute of Organic Chemistry and Biochemistry of the CAS, Flemingovo nám. 2, 16000 Prague, Czech Republic; ‡ Department of Physical Chemistry, 52735 University of Chemistry and Technology, Prague, Technická 5, 166 28 Prague, Czech Republic; § Chemical Biology Research Group, Institute of Organic Chemistry, 557117 HUN-REN Research Centre for Natural Sciences, Magyar Tudósok Krt. 2., 1117 Budapest, Hungary

## Abstract

Fluorogenic probes, which become fluorescent only upon specific activation, enable no-wash imaging with an excellent signal-to-noise ratio. Despite significant progress in the development of such probes, challenges remain in achieving efficient quenching and substantial fluorescence enhancement across the whole visible spectrum while maintaining good synthetic accessibility. In this work, we introduce a new class of bioorthogonally activatable fluorogenic probes based on triazinium salts (Trz^+^), which act as highly efficient fluorescence quenchers. These Trz^+^-fluorophore conjugates are easily synthesized from common precursors and commercially available fluorophores, covering a broad spectral range from blue to far-red wavelengths. Mechanistic studies provide molecular insights into the quenching mechanism, attributed to the charged Trz^+^ core, which triggers substantial fluorescence turn-on upon bioorthogonal reaction with a strained dienophile. The versatility and ease of synthesis of these probes, along with their noteworthy photophysical properties, make them highly valuable for a wide range of bioimaging applications, including the visualization of intracellular organelles, drug molecules, HaloTag fusion proteins, genetically encoded intra- and extracellular proteins, cell surface antigens, and metabolically labeled glycoconjugates.

## Graphical abstract

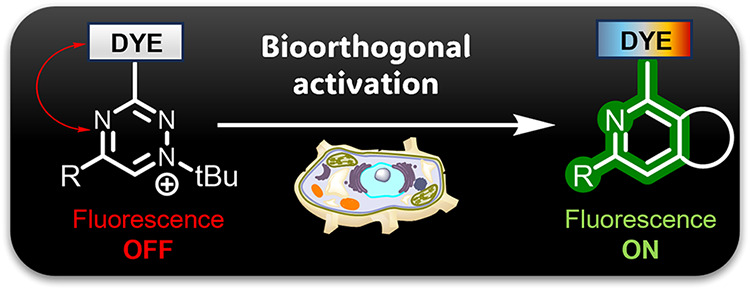

## Introduction

Fluorescence microscopy allows precise visualization of cellular structures and dynamic processes, with specificity achieved through targeted fluorescent labeling of biomolecules. This enables precise tracking of molecular interactions and cellular responses, which is crucial for understanding complex biological processes. In this context, small-molecule fluorophores play a critical role allowing labeling with minimal perturbation of the biological system.^1–3^ Prominent examples include visualization of genetically encoded self-labeling tag proteins or noncanonical amino acids (ncAAs).^4,5^ Advanced labeling studies aiming at cellular drug target identification is another active area of interest.^6^

Despite their beneficial photophysical properties such as high brightness and photostability,^7^ application of small synthetic fluorophores in living cells often comes along with significant background signal.^8^ Consequently, extensive washing steps are needed to achieve good signal-to-noise ratio (SNR). While compatible with fixed cells, extensive washing steps can be detrimental to living systems. An elegant solution to this problem is offered by fluorogenic probes.^9,10^ Such probes exist in a quenched, nonfluorescent state, and become fluorescent only after a specific activation event.^11^ Fluorogenic probes which can be activated through specific bioorthogonal reactions became a powerful tool to dynamically track a variety of biological events.^12–15^ These probes typically contain a fluorophore paired with a fluorescence-quenching group whose function is turned off upon new bond formation or elimination, restoring the native emission of the fluorophore.^9,11,16^ The best performing probes achieve excellent SNR, facilitating no-wash live cells imaging.^10^ The level of signal enhancement (or fluorescence turn-on value) is one of the key evaluation parameters of fluorogenic probes. In addition, having red-shifted absorbance and emission maxima is especially valued due to deeper penetration depth, low phototoxicity, and minimal autofluorescence in this spectral region.^17–19^ Multicolor imaging application utilizing single activatable fluorescence quencher is also of high demand. However, these attributes of fluorogenic probes remain difficult to achieve.

The pioneering work on bioorthogonally activatable probes was based on the Staudinger ligation^20^ and on the [3 + 2] azide–alkyne cycloaddition.^21,22^ More recent efforts resulted in the development of fluorogenic azides emitting in the red to far-red region.^23,24^ Other notable examples of functional groups rendering bioorthogonal probes fluorogenic include cyclooctynes,^22,25^ sydnones,^26,27^ and pyrones.^28^ A distinct strategy includes the in situ formation of fluorophores during the labeling reaction, such as pyrazolines^29–33^ and pyridazines.^34–37^

Due to the exceptional reaction kinetics and orthogonality to biological systems, the inverse electron-demand Diels–Alder reaction (IEDDA) between 1,2,4,5-tetrazines (Tz) and strained dienophiles is among the most versatile bioorthogonal tools.^38–41^ In 2010, Devaraj et al. reported the first fluorogenic Tz-BODIPY conjugates, leveraging the fluorescence quenching capability of the colored tetrazine scaffold.^42^ These conjugates, derived from a simple Tz-amine precursor, exhibited low fluorescence turn-on values (up to 20-fold). A follow-up study from the same laboratory demonstrated that linking the Tz core to the BODIPY fluorophore via a conjugated linker significantly enhanced the quenching, resulting in improved SNR in the range ∼500 nm (up to 1600 fold turn-on).^43^ Expanding this concept to coumarin dyes led to the development of ultrafluorogenic probes with remarkable fluorescence turn-on values (up to 11,000-fold at ∼480 nm).^44^ Our findings revealed that the fluorescence turn-on of these conjugated coumarin tetrazines is influenced by the nature of the dienophile,^45^ which was subsequently confirmed and rationalized through computational studies.^46–48^ In addition, Seoul-Fluor-Tz probes were recently found to provide differently colored click products with various dienophiles, with the turn-on ratios up to 1000-fold.^49^ However, the structural design requirements for such probes often introduce synthetic challenges, limiting their accessibility and scalability.^50,51^ By utilizing through-bond energy transfer (TBET),^52–59^ twist intramolecular charge transfer (TICT)^60^ or Förster resonance energy transfer (FRET),^60–62^ these probes can achieve substantial brightness enhancement (often several thousand fold), though this is often limited by wavelength-dependent quenching efficiency and complex synthesis (Figure 1).^63,64^ To overcome this limitation, Wombacher employed a Dexter-type electron transfer strategy by conjugating tetrazines to more red-shifted xanthene dyes via strategically positioned flexible linkers.^65^ While this approach expands the emission range, the enhancement in brightness comes at the cost of a complex synthesis process involving over 10 steps. Exploring photoinduced electron transfer (PET), Sauer et al. simplified the synthesis of a series of fluorogenic tetrazines starting from commercially available Tz-amines. These probes span the whole visible spectrum but reach only modest fluorescence enhancement (up to 40-fold).^66^ Despite these achievements, the number of moieties having the ability to serve as efficient quenchers of fluorescence that can be combined with various visible-NIR emitting fluorophores remains low.

**Figure 1:**
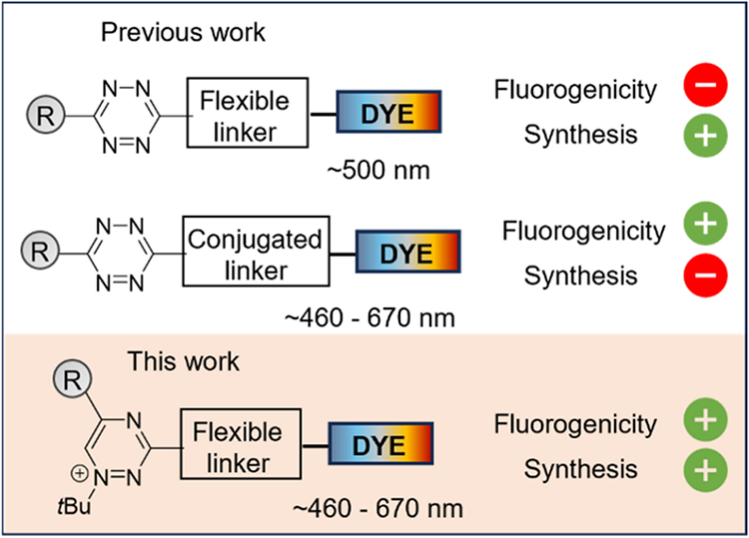
An overview of recent fluorogenic probe development highlighting the fluorogenicity/synthesis difficulty trade-off. The new triazinium probes are both easily synthetically available and highly fluorogenic.

Recently, we introduced triazinium ligation, a bioorthogonal reaction involving triazinium salts (Trz^+^).^67^ Compared to tetrazines, these charged dienes offer advantageous properties, including excellent cell permeability and stability. Trz^+^ linked to coumarin fluorophores via a conjugated phenyl linker demonstrated fluorogenic behavior upon reaction with *endo*-bicyclo­[6.1.0]­non-4-yn-9-ylmethanol (***endo*** **-BCN**).^68^ Building on these studies, we show here that Trz^+^ salts can act as highly effective, bioorthogonally activatable, versatile fluorescence quenchers. These probes are readily synthesized from accessible Trz^+^ precursors and commercially available fluorophores. Computational and spectral analysis have uncovered a distinctive quenching mechanism that enables the charged Trz^+^ core to uniquely quench fluorophores across the visible spectrum, from blue to far-red wavelengths. Finally, we demonstrate the successful application of these new probes in various bioimaging experiments.

## Results and Discussion

### Design and Synthesis of Fluorogenic Triazinium Salts

Our work began with the design and synthesis of a versatile triazinium precursor suitable for use with commercially available active esters of various fluorophores to generate fluorogenic conjugates. The presence of an electron-donating piperidinyl group at the aryl moiety in the C5 position was found to enhance the fluorogenicity of the resulting conjugates significantly. This structural modification is key to observing the broad-spectrum quenching properties of the triazinium salts. Following this design principle, we synthesized the key compound **Trz** ^**+**^ **1** in four steps starting from brominated **SMeTrz**. The synthetic route involved a Buchwald-Hartwig reaction, *tert*-butylation, Liebeskind-Srogl cross-coupling, and in situ acidic N-Boc deprotection (Figure 2A).^68^

**Figure 2:**
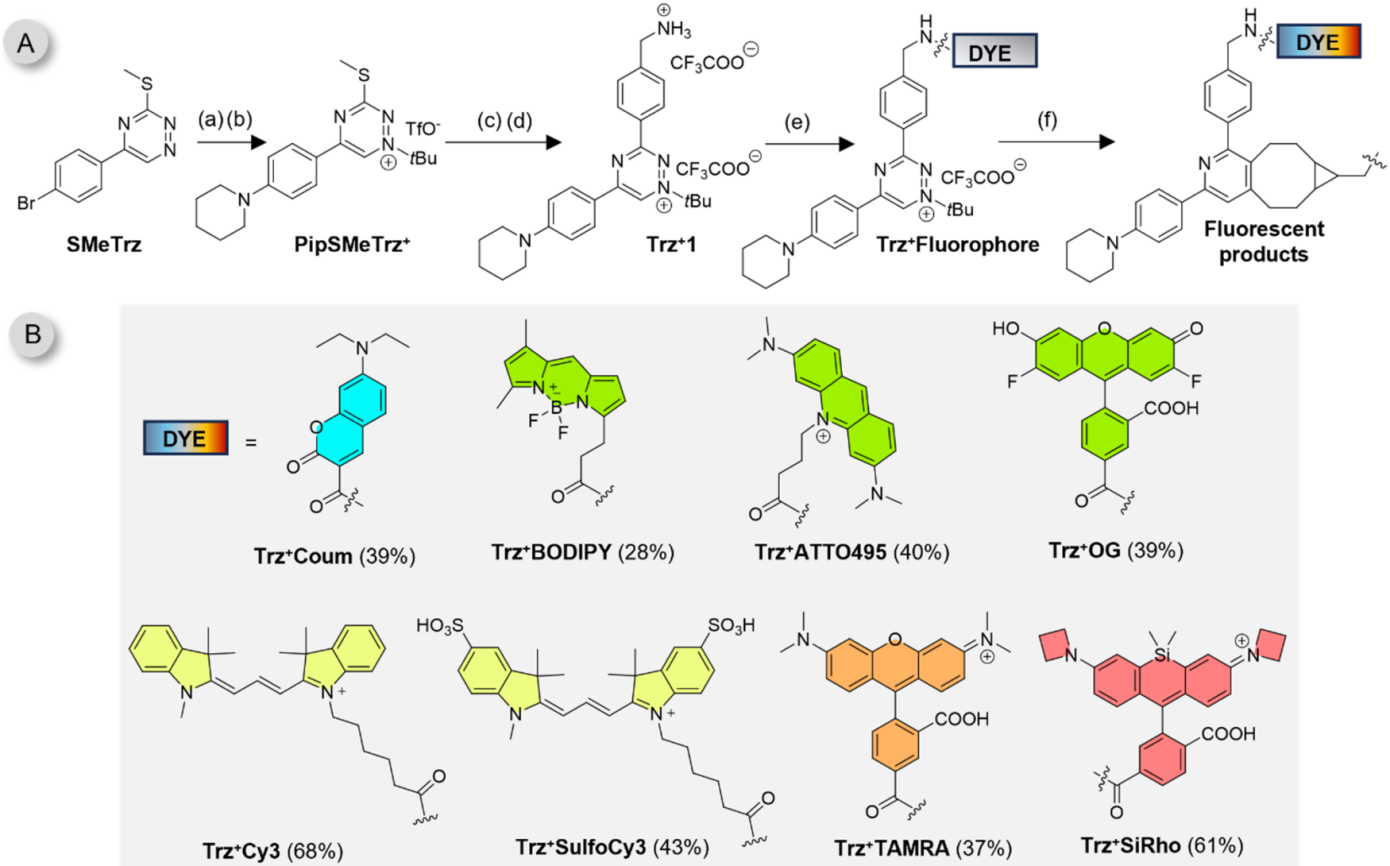
(A) Synthetic route to triazinium-fluorophore conjugates. (a) piperidine, Cs_2_CO_3_, XPhos, Pd_2_(dba)_3_, dioxane, 80%. (b) TfOH, isobutene, DCM, 88%. (c) ArB­(OH)_2_, Pd­(PPh_3_)_4_, CuTC, dioxane, (d) TFA, DCM, 55% over two steps. (e) Dye-active ester, acetonitrile or DMF, (f) BCN (Figure S1). CuTC = copper­(I) thiophene-2-carboxylate, dba = dibenzylideneacetone. (B) structures of studied fluorophores with their isolated yields; the ring fill color represents the emission color of the respective fluorophore.

This sequence provided **Trz** ^**+**^ **1** in an overall yield of 34% from inexpensive and readily available starting materials. The compound shows good stability in a buffered solution (PBS, pH= 7.4, 37 °C) and in the presence of excess glutathione within 24 h (Table S8, S9 and Figures S21–S23).

The amino group of **Trz** ^**+**^ **1** offers a convenient attachment site for fluorophores (or other substituents), which are commercially available as active esters. Using simple peptide couplings, we prepared a series of triazinium-fluorophore conjugates (**Trz** ^**+**^ **Fluorophore**) with fluorescence emissions ranging from the blue to the far-red region (λ_em_ ∼460–670 nm), achieving yields between 28% and 68% after isolation (Figure 2B). For comparison studies, we also synthesized a series of tetrazine-fluorophore conjugates (**TzFluorophore**) starting from methyl tetrazine amine (**MeTzNH**_**2**_, SI). We used this structurally similar Tz derivative, which is commercially available and reacts with BCN at a similar rate as **Trz** ^**+**^ **1** (*k*_2_ ∼ 45 M^–1^. s^–1^, Figure S20). It should be noted that other Tz derivatives were reported to react faster, especially with the TCO dienophile.^69^

### Photophysical Properties of Trz^+^Fluorophores

The absorption properties of **Trz** ^**+**^ **1** are significantly different from the tetrazine analogue **MeTzNH**_**2**_ which can be inferred from the distinct colors of their solutions (Figure 3A,3B “before”). The aqueous solution of tetrazine has the typical pink-reddish color (λ_max_ = 521 nm), while the **Trz** ^**+**^ **1** solution is significantly darker with an intense panchromatic absorption between ca. 350 and 700 nm (λ_max_ at 426 and 523 nm, Figure 3A,3B). This broad absorption band primarily results from the presence of the piperidinyl group on the aryl moiety at the C5 position, as compounds without this substituent are almost colorless. The extinction coefficient of **MeTzNH**_**2**_ at 521 nm is 180 M^–1^ cm^–1^ and **Trz** ^**+**^ **1** is 15 000 M^–1^ cm^–1^ at 426 nm and 18 000 M^–1^ cm^–1^ at 523 nm, respectively, underlying the higher absorptivity of the triazinium scaffold. The **MeTzNH**_**2**_ **-BCN** click product solution is colorless, whereas the **Trz** ^**+**^ **1** click product solution is pale yellow. Notably, neither of these solutions exhibited any detectable fluorescence (Table 1 and SI).

**Table 1:** Photophysical Properties of the **T­(r)­z^(+)^** Probes and Their ***endo*-BCN** Click Products^a^

Probe	λ_Abs_ (nm) **T(r)z** ^**(+)**^ ^b^		λ_Abs_ (nm) T(r)z^**(+)**^ **+ BCN** ^b^		λ_Em_ (nm) **T(r)z** ^**(+)**^ **+ BCN** ^b^		Φ*_f_* **T(r)z** ^**(+)**^ ^c^		Φ*_f_* **T(r)z** ^**(+)**^ **+ BCN** ^c^		fluorescence turn-on^d^	
**Trz** ^**+**^ **1**	272	275	330	310	n.r.	n.r.	ND	ND	ND	ND	ND	ND
	425	426	421	409								
	523	523	517	530								
**MeTzNH**_**2**_	261	262	276	276	n.r.	n.r.	ND	ND	ND	ND	ND	ND
	320	320										
	532	521										
**Trz** ^**+**^ **Coum**	425	425	417	423	465	469	0.0029	<0.001	0.024	0.014	16	15
**TzCoum**	430	424	432	424	463	466	0.0191	0.0107	0.053	0.022	9	2
**Trz** ^**+**^ **BODIPY**	508	504	506	504	514	510	0.0190	0.0053	0.064	0.151	5	54
**TzBODIPY**	506	504	506	504	511	510	0.0139	0.0064	0.713	0.469	73	87
**Trz** ^**+**^ **ATTO495**	503	500	503	501	527	523	0.0084	0.0013	0.131	0.083	16	56
**Trz** ^**+**^ **OG**	500	498	500	500	525	534	0.0194	0.0128	0.680	0.362	48	44
**Trz** ^**+**^ **Cy3**	548	545	548	545	572	561	0.0075	0.0019	0.188	0.027	25	14
**TzCy3**	545	545	548	545	570	559	0.0764	0.0137	0.682	0.035	15	2
**Trz** ^**+**^ **SulfoCy3**	552	553	552	553	565	565	0.0058	0.0014	0.144	0.051	27	36
**TzSulfoCy3**	553	552	553	549	565	565	0.1352	0.0318	0.521	0.065	5	2
**Trz** ^**+**^ **TAMRA**	553	549	553	549	581	579	0.0018	0.0011	0.026	0.098	18	84
**TzTAMRA**	553	549	553	549	582	578	0.0851	0.055	0.098	0.112	1	2
**Trz** ^**+**^ **SiRho**	631	637	631	648	674	669	0.1267	0.0085	0.726	0.614	2	45

a Left column: 20% DMSO in PBS (pH = 7.4), Right column: 50% MeCN in H_2_O

b λ_Abs_ (*c* = 1 μM) before and after addition of 10 equiv BCN

c Fl. quantum yields (Φ_*f*_ *)* measured in triplicate with 7-(diethylamino)­coumarin-3-CA, BODIPY FL, Cy3 CA, 5-TAMRA CA, JF 646 CA as standards. CA = carboxylic acid, n.r. = not relevant (no fluorescence observed), ND = not determined

d Turn-on calculated by dividing the integrated emission areas.

**Figure 3:**
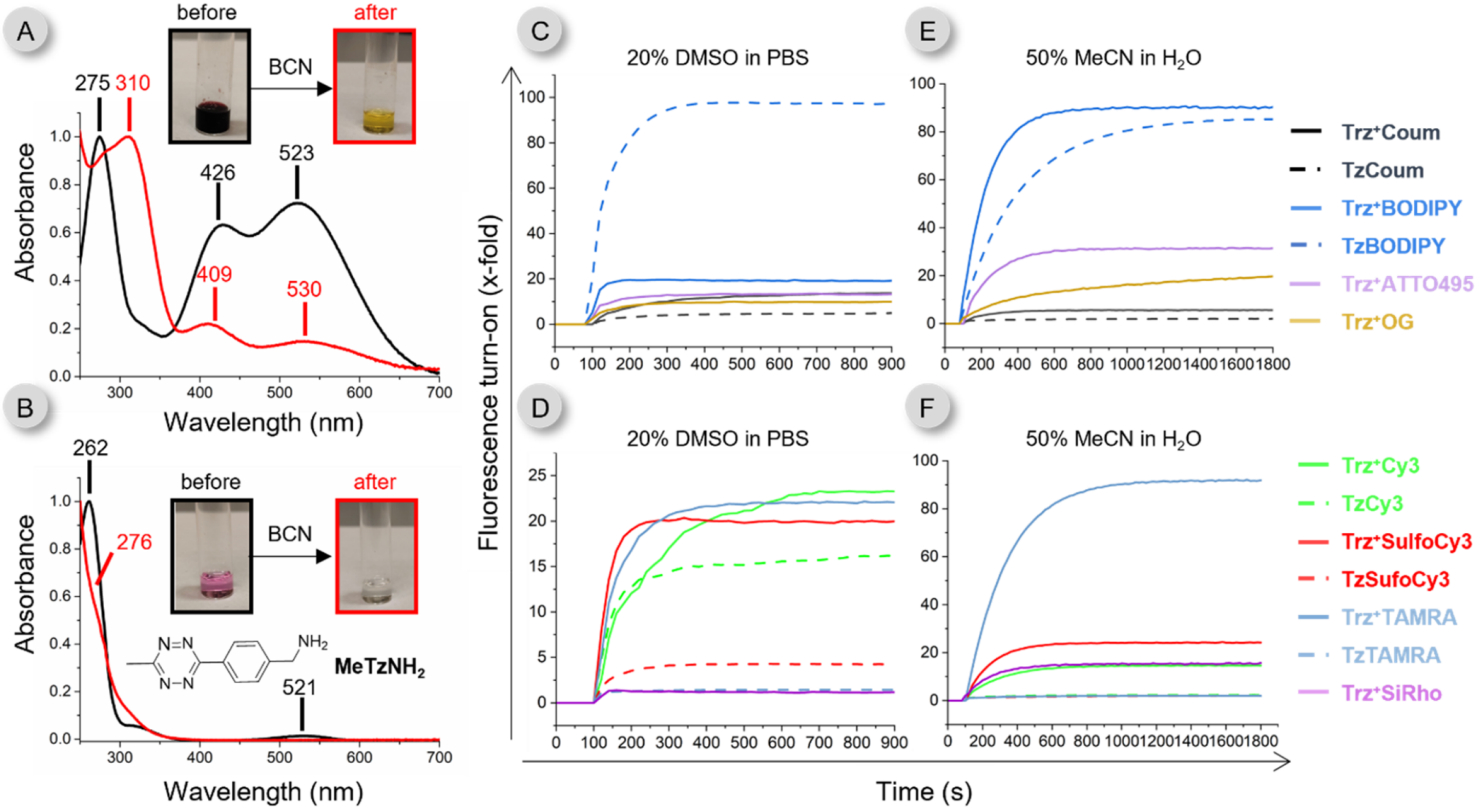
(A) Absorption spectrum of **Trz** ^**+**^ **1** before and after reaction with BCN in 50% MeCN/H_2_O (1:1) mixture. (B) Absorption spectrum of **MeTzNH**_**2**_ before and after reaction with BCN in 50% MeCN/H_2_O (1:1) mixture. Photos show vials containing 2.5 mM solution of the compounds in the same solvent mixture. (C and D) Fluorescence turn-on of the conjugates as a function of time in PBS containing 20% DMSO. (E and F) Fluorescence turn-on of the conjugates as a function of time in 50% MeCN in H_2_O mixture.

We next characterized the emission properties of the **Trz** ^**+**^ **Fluorophore** probes, their **Tz** analogues and their click products with ***endo*** **-BCN** (Figure S1). All probes were freshly purified by analytical HPLC prior to the measurements (Figure S4) to eliminate any potential interference from fluorescent impurities. The resulting photophysical properties are summarized in Table 1 (details can be found in Tables S1, S2 and Figures S2–S5). Measurements were performed in two solvent systems: MeCN/H_2_O (1:1, *v*/*v*) mixture and more biologically relevant PBS buffer (pH = 7.4) containing 20% (*v*/*v*) DMSO, all conducted in triplicate. Organic cosolvents were used to prevent precipitation of the probes, which complicates both measurements and data analysis.

Because there are different ways of calculating the fluorescence turn-on values reported in the literature,^8,52,55–58,70^ we decided to compare different methods to obtain these numbers. First, by dividing the fluorescence intensity of the click product at the emission maximum by the residual fluorescence intensity of the quenched probe, second by dividing the respective integrated areas of the whole emission signals, and third by dividing the fluorescence quantum yields of the probes before and after the click reaction. All these values are summarized in Table S2. The values presented in Table 1 were calculated using the second method, based on the integrals of the whole emission signals. In general, the highest turn-on values were obtained by the first method, while the other two gave comparable results. The triazinium core was generally more effective in quenching fluorescence than the tetrazine moiety. This was evident from the lower fluorescence quantum yields (Φ_*f*_) of the initial **Trz** ^**+**^-**fluorophore** conjugates compared to their tetrazine counterparts (on average by the factor of ∼15).

The fluorescence was successfully restored after the click reaction with ***endo*** **-BCN**. The Φ_*f*_ values of the click products from Tz reactions were generally higher than those from Trz^+^ reactions, indicating a residual quenching of the fluorophore in the Trz^+^ click product. This is consistent with weak broad absorption of the Trz^+^ click product capable of energy transfer from the excited chromophore (Figure 3A, red line). Differences in fluorescence turn-on values between the two solvent systems depend on the conjugate, with some probes exhibiting stronger fluorescence in the buffered system and others in the MeCN/H_2_O mixture. Overall, dyes conjugated to the Trz^+^ core showed higher fluorescence turn-on values than the Tz probes, except for the BODIPY conjugate.

Unlike tetrazines, even TAMRA (**Trz** ^**+**^ **TAMRA**) and silicon rhodamine (**Trz** ^**+**^ **SiRho**) probes were efficiently quenched by the triazinium moiety. These probes exhibited fluorescence light-up values of 84-fold for TAMRA and 45-fold for SiRho in the MeCN/H_2_O mixture, exceeding previously reported values for conjugated tetrazines^8^ and approaching those of difficult to prepare close-proximity tetrazine-dye conjugates in reaction with BCN.^65^

We also measured the kinetics of fluorescence turn-on during the click reaction in both solvent systems (Figures 3 and S6) at low micromolar concentration. As expected, the fluorescent signal increased more rapidly in PBS containing 20% DMSO, reaching a plateau after approximately 10 min, whereas in the MeCN/H_2_O (1:1) mixture, it took about 20 min to reach maximum fluorescence intensity.

### Fluorescence Quenching Mechanism

Prompted by the experimental results showing promising fluorescence quenching properties of the triazinium core, superior to the tetrazine analogues, we aimed to clarify the molecular mechanisms behind this effect. The fluorescence enhancement observed in fluorogenic click reactions is directly related to the relative fluorescence quantum yield before and after the IEDDA process. An ideal fluorogenic quencher should completely suppress the fluorophore’s emission prior to the click reaction and fully restore it afterward. To compare the relative fluorescence enhancements of the studied Trz^+^ and Tz-based molecules, we determined the relative fluorescence emission by dividing the fluorescence quantum yield of Trz^+^ and Tz conjugates, as well as their corresponding BCN adducts, by the fluorescence quantum yield of the unsubstituted fluorescent dyes (Figure 4A). This approach enables a direct comparison of the relative quenching efficiencies of Trz^+^ and Tz before and after the click reaction.

**Figure 4:**
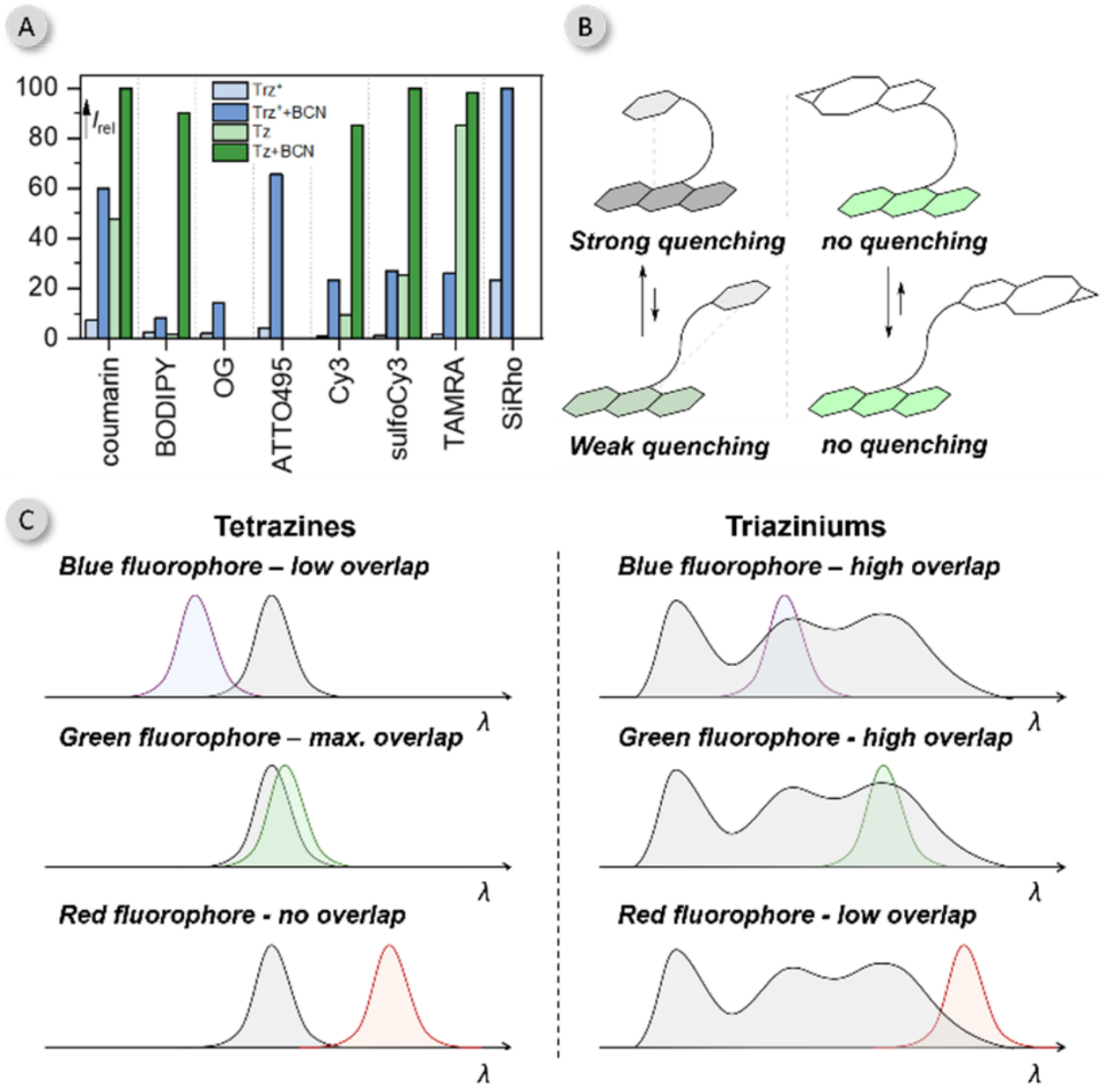
(A) Relative fluorescence emission of Trz^+^ and Tz derivatives normalized to the fluorescence quantum yield of the unsubstituted fluorescent dye in water. (B) Schematic representation of the stacked (top) and nonstacked “open” conformations (bottom) of dyes and quenchers before and after the click reaction. (C) Schematic comparison illustrating the quenching efficiencies of different fluorophores by Trz^+^ and Tz.

In general, the relative quenching efficiency correlates with the spectral overlap between the fluorophore’s emission and the quencher’s absorption (Figures 4A and S12–S15). Tetrazine exhibits a weak absorption maximum at approximately 520 nm (ε_max_ = 180 M^–1^ cm^–1^), making it an efficient quencher for fluorophores with similar excited-state energies, typically those in the green spectral region (e.g., BODIPY). After the IEDDA reaction, the BCN adducts of Tz lose their visible-light absorption, thereby fully restoring the native emission of the fluorophore.

In contrast, Trz^+^ demonstrates a much stronger absorption in the visible range, with an intense absorption maximum (ε_max_ = 18,000 M^–1^ cm^–1^), allowing it to universally quench a broad range of fluorophores across the 450–650 nm spectral range. However, the BCN adducts of Trz^+^ retain absorption in the visible region (Figure 3A), which prevents complete restoration of the fluorophore’s emission. This residual absorption reduces the turn-on fluorescence values for Trz^+^, thereby limiting its overall fluorescence enhancement efficiency.

Fluorophores and quenchers in our study are connected by a nonconjugated, flexible linker. As a result, the fluorophores and quenchers remain electronically isolated, with each component retaining its individual electronic properties. This is confirmed by the perfect overlap of the conjugate spectrum with the sum of the individual component spectra (Figures S7 and S8). Notably, increasing the concentration of the Trz^+^ quencher does not induce aggregation but instead reduces the molar extinction coefficient of the fluorophore by approximately 50% (Figure S9).

To investigate the conformational flexibility of the linker and its role in enabling a through-space coupling between fluorophore and quencher in Trz^+^- and Tz-fluorophore conjugates, we performed computational screening of their conformational spaces and the preferred geometries. Our analysis revealed that all Trz^+^-fluorophore conjugates exclusively adopt a stacked conformation (Figure 4B), where the Trz^+^ core and fluorophore π-system interact at distances of approximately 3.4–3.8 Å. This suggests that static quenching plays a significant role in the overall fluorescence quenching efficiency of these conjugates.

To experimentally inspect the quenching mechanism, we studied the fluorescence quenching of **TAMRA** by **Trz** ^**+**^ **1**. In this intermolecular system, Stern–Volmer analysis indicated a mixed dynamic and static quenching mechanism, with a strong preference for static quenching (Figures S8–S10). This finding supports the formation of a weakly emissive, noncovalent ground-state complex between **TAMRA** and **Trz** ^**+**^ **1**.

In the intramolecular system, **Trz** ^**+**^ **TAMRA**, fluorescence decay analysis revealed two distinct components. The major short decay (τ_fl_ = 0.17 ns) closely resembled **TAMRA** in the presence of a high excess of **Trz** ^**+**^ **1** (10 equiv), while the residual long decay component (τ_fl_ = 1.7 ns) was similar to that of the noncomplexed fluorophore (τ_fl_(TAMRA) = 2.0 ns). This indicates that **Trz** ^**+**^ **TAMRA** might exist in an equilibrium between a “stacked” and an “open” conformer, each contributing differently to fluorescence quenching (Figure 4B). Both the quenching efficiency within individual conformers and their relative equilibrium significantly influence the overall fluorescence quenching observed in triazinium derivatives.

Similarly, most of the Tz-fluorophore conjugates adopt the analogous “stacked” conformations; however, conformational sampling revealed a stronger preference for nonstacked “open” conformations (Table S3), in which the Tz core and the fluorophore’s π-system are not in direct contact. Namely, a preferred open configuration was found for **TzOG** and **TzTAMRA** derivatives.

While the absence of a low-energy stacked conformation in our sampling does not definitively exclude its accessibility—considering the inherent limitations and accuracy of the sampling method—it suggests a weaker attractive interaction between the Tz and fluorophore fragments compared to Trz^+^ complexes. This finding is furthermore consistent with calculated intermolecular interaction energies (Table S4), favoring Trz^+^-fluorophore interaction by a difference of ∼6.5 kcal/mol. We attributed this difference to the lack of a positive charge in the Tz-based quencher, which diminishes the electrostatic interactions that contribute to stacking in Trz^+^-fluorophore conjugates. As a result, the reduced static quenching in Tz-fluorophore conjugates likely plays a role in the lower fluorescence quenching efficiency and fluorescence turn-on values observed for Tz-based probes relative to Trz^+^-based probes, as shown in Table 1.

Next, we investigated the quenching mechanism to determine whether excited-state electron transfer (PeT) or energy transfer plays the dominant role. Our analysis shows that although ground-state electron transfer from the fluorophore to the quencher is unfavorable, excitation enables exothermic PeT for both Trz^+^- and Tz-based quenchers (for details, see SI). The click reaction with *endo*-BCN induces a substantial negative shift in the reduction potentials of the Trz^+^ and Tz cores, making the fluorescence electron-transfer-induced quenching in the click products unlikely, even in the excited state.

To evaluate the energy transfer mechanism, we calculated time-dependent DFT (TDDFT) vertical excitation energies for both quenchers and fluorophores. Our results show that the Trz^+^ quencher exhibits low-energy absorption bands with significant intensity in the same spectral region or slightly below that of the investigated fluorophores (*V*_exc,calcd_ ∼ 490 nm). In contrast, Tz quenchers display their first intense transitions at *V*_exc,calcd_ ∼ 270 nm, suggesting that Trz^+^ is a more efficient quencher through an energy-transfer mechanism than Tz. In the click products, however, the *V*_exc,calcd_ values shift to higher energies (*V*_exc,calcd_ ∼ 330 nm for Trz^+^+BCN and ∼260 nm for Tz+BCN), reducing the likelihood of fluorescence quenching through energy transfer. We determined the spectral overlap integrals of all studied fluorophores and quenchers and calculated the Förster radius to be typically in the 9–13 Å range for tetrazines (except for **TzBODIPY** with *R*_0_ = 24 Å, exhibiting excellent quenching), while being much higher (30–53 Å) for Trz^+^ (see SI). This indicates that Förster resonance energy transfer (FRET) may be considered to play a major role in fluorescence quenching. The conformation of the studied conjugates can affect the quenching efficiency. As the computed average distance of the fluorophore and quencher is ∼4–6 Å for the “stacked” conformer and ∼10–14 Å for the “open” conformer, tetrazines in the open conformer have a fluorophore-quencher distance comparable to their Förster radius. Also, the preference for the open conformer for the Trz^+^ BCN click products caused by the decreased electrostatic interaction (i.e., the absence of the charged triazinium moiety) causes relatively high fluorescence turn-on values even for Trz^+^-derivatives retaining non-negligible spectral overlaps after the click reaction.

In summary, our calculations and spectroscopic investigations indicate that both Trz^+^ and Tz quench fluorescence mostly through the FRET mechanism, with PeT playing a minor role, as demonstrated by the correlation of the quenching efficiency with the spectral overlap (Figures S14–S15). The trends in fluorescence quenching are illustrated in a simplified graphic (Figure 4C). Tetrazines efficiently quench green fluorophores with similar excited-state energies, while triazinium salts exhibit a more universal quenching ability, effectively quenching most visible light-absorbing fluorophores. Fluorophores with similar spectral properties are generally quenched with comparable efficiency, with two notable exceptions: **TzSulfoCy3** vs **TzTAMRA** and **Trz** ^**+**^ **OG** vs **Trz** ^**+**^ **ATTO495**. **TzTAMRA** is quenched neither prior to nor after the click reaction. This observation can likely be attributed to the preference of **TzTAMRA** to form an open conformation, reducing the static quenching efficiency (Table S4). In analogy, **Trz** ^**+**^ **ATTO495** exhibits a significantly higher fluorescence turn-on after the click reaction compared to similar fluorophores (**OG**). This behavior is likely due to the decrease of attractive interactions between the BCN adduct and the **ATTO495** fluorophore, which promotes the population of the open conformation after the click reaction, thereby reducing residual quenching in the BCN conjugate.

### Application of Trz^+^Fluorophores in bioimaging

Despite the increasing use of biologically active small molecules in biological and preclinical studies, visualizing them in live cells remains challenging, limiting the ability to obtain detailed information about their distribution and mode of action.^71^ In this context, fluorogenic probes offer a significant advantage, as they eliminate the need for washing steps to remove excess labeling reagent. Given their favorable fluorogenic properties and unique quenching mechanism, we explored the potential of the new triazinium fluorophores in such studies.

With the goal of identifying the most promising derivatives, we first performed a series of preselection experiments inside living cells treated with a BCN-triphenylphosphonium conjugate (**BCN-TPP**), which we previously developed for intracellular fluorescence labeling studies.^36^ The TPP moiety ensures that the reaction takes place specifically in mitochondria.^72^ To install the dienophile, live U2OS osteosarcoma cells were treated with 5 μM **BCN-TPP** for 15 min and were subsequently washed to remove any extracellular compound. Cells that were not treated with **BCN-TPP** served as a control (w/o). The fluorogenic probes were then added for 15 min at 1 μM concentration. The cells were inspected on a confocal microscope without additional washing steps (Figures S24 and S26) and the fluorescent signal intensities were quantified by flow cytometry (Figures 5B and S25). Under these experimental conditions, the most prominent fluorescent signal formed in cells treated with **Trz** ^**+**^ **Coum** and **Trz** ^**+**^ **BODIPY**. The signal formed in cells treated with **Trz** ^**+**^ **ATTO495** and **Trz** ^**+**^ **TAMRA** was somewhat weaker. **Trz** ^**+**^ **Cy3** provided also excellent labeling signal but accompanied by slightly higher background fluorescence in control cells. Cells labeled with **Trz** ^**+**^ **SiRho** probe also showed notable fluorescent signal buildup, as compared to the control group. (Figure S24). Among the tetrazine probes, only **TzBODIPY** and **TzCy3** led to the formation of specific fluorescent signals. The high signal from **TzTAMRA** probe turned out to be unspecific as it was also present in control cells.

**Figure 5:**
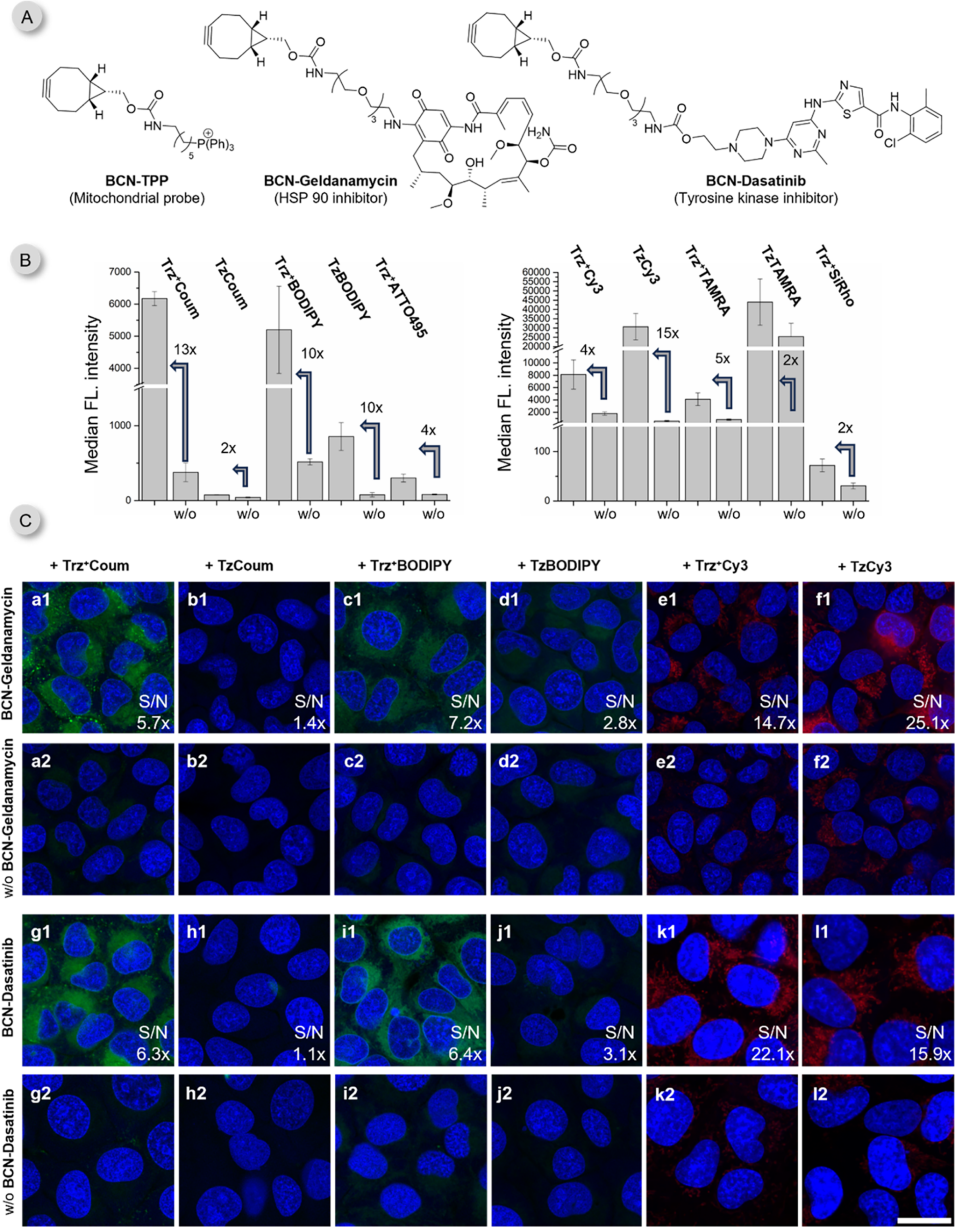
No-wash live cell intracellular labeling. (A) Structures of BCN-tagged probes. (B) Data from flow cytometry analysis of cells treated for 15 min with 5 μM **BCN-TPP**, washed, and subsequently treated for 15 min with 1 μM **T­(r)­z** ^**(+)**^ **Fl** (mean ± SD, *n* = 3). (C) Representative images from confocal microscopy of live U2OS cells treated for 2h with 2 μM **BCN-Geldanamycin** (a–f) or **BCN-Dasatinib** (g–l), washed, and subsequently treated for 30 min with 1 μM of **T­(r)­z** ^**(+)**^ **Fl**. Cells that were not incubated with BCN-tagged probes were used as controls (labeled as w/o). The images were acquired without additional washing steps and using the same microscope set up for the same dyes. Nuclei were stained with Hoechst 33342 or DRAQ5 and are shown in blue. Signal from the probes is shown in green or red, respectively. Scale bar is 20 μm. w/o = without. The signal-to-noise ratios (S/N, *n* ≥ 3 cells) are indicated in the bottom, right corner (see ESI for details). The fluorescence turn-on values are in Tables S13 and S14.

To assess the potential of our newly developed fluorogenic triazinium dyes for visualizing drug molecules in live cells, we synthesized a BCN-tagged geldanamycin (**BCN-Geldanamycin**, Figure 5A; for synthesis details, see SI). Geldanamycin is a well-characterized inhibitor of HSP90 chaperones and has been extensively studied for its anticancer properties.^73^ Although a geldanamycin–fluorescein conjugate has been previously reported,^74^ it was employed exclusively in fluorescence polarization assays for screening HSP90 inhibitors. To the best of our knowledge, fluorescent imaging of geldanamycin in live cells has not been described to date.

To explore this possibility, we evaluated a panel of fluorogenic triazinium probes for their ability to label **BCN-geldanamycin** in living osteosarcoma cells. Representative imaging results are shown in Figure 5C (for extended experiments see Figure S32). Our data clearly demonstrate that several of the new triazinium probes yield substantially improved contrast and SNR compared to their tetrazine analogues (compare a1 vs b1, c1 vs d1 etc. in Figure 5C). Notably, most tetrazine dyes failed to label geldanamycin under otherwise identical conditions.

To confirm that the observed labeling performance was not limited to geldanamycin, we extended our study to another clinically relevant drug, dasatinib. Dasatinib is an orally administered multikinase inhibitor targeting BCR-ABL and SRC family kinases and is widely used in the treatment of chronic myeloid leukemia.^75^ Live cells treated with **BCN-dasatinib** followed by labeling with triazinium dyes exhibited clear intracellular fluorescence in most cases, which was absent in untreated control cells. As with geldanamycin, tetrazine probes either failed to label dasatinib effectively or generated high background fluorescence (for extended experiments see Figure S31).

Collectively, these results highlight the superior performance of fluorogenic triazinium reagents for live-cell imaging of small-molecule drugs. In many cases, triazinium dyes enabled visualization with high SNR where analogous tetrazines were ineffective.

To evaluate the performance of cell-impermeable probes containing sulfo groups for specific cell surface labeling applications, we prepared BCN-PEG3-conjugated Concanavalin-A **(BCN-Con-A)**. Concanavalin-A (Con-A) is a lectin that binds cell surface carbohydrates with high specificity for α-d-mannose and α-d-glucose residues.^76^ To label these glycoconjugates, live U2OS cells were treated with 10 μg/mL **BCN-Con-A** for 15 min and then washed to remove unbound conjugate. Cells untreated with **BCN-Con-A** served as controls. After the addition of 2.5 μM fluorogenic probes for 30 min, the cells were inspected on a confocal microscope without washing (Figures 6A and S30). Both extracellular probes, **Trz^+^OG** and **Trz^+^SulfoCy3** derivatives, clearly labeled **BCN-Con-A** on the cell surface. Part of the high signal observed on cells labeled with **TzSulfoCy3** was unspecific.

**Figure 6:**
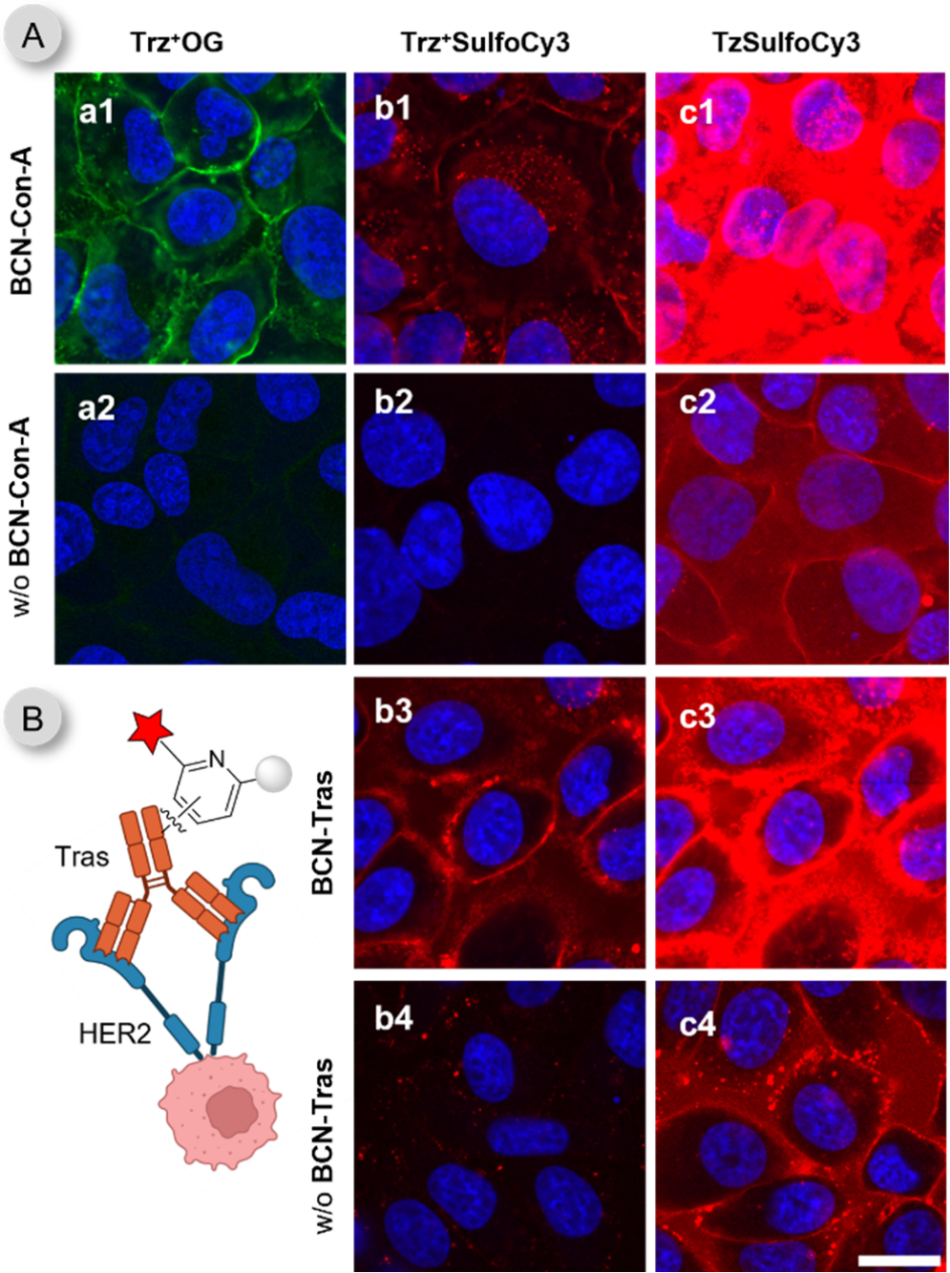
Extracellular no-wash live cell labeling. Confocal microscope images of (A) Live U2OS cells were treated with **BCN-Con-A** (10 μg/mL for 15 min) and subsequently for 30 min with 2.5 μM of the indicated fluorogenic probes. Cells that were not incubated with **BCN-Con-A** were used as controls (labeled as w/o **BCN-Con-A**). (B) Live SKBR3 cells treated with **BCN-Tras** (10 μg/mL for 1 h) and subsequently for 30 min with 2.5 μM of the indicated fluorogenic probes. Cells that were not incubated with **BCN-Tras** were used as controls (labeled as w/o **BCN-Tras**). The images were acquired without additional washing steps. Nuclei were stained with DRAQ5 or Hoechst 33342 and are shown in blue. Further experiments performed at different microscope settings are available in the SI. Scale bar is 20 μm.

Next, we assessed the performance of the sulfoCy3-containing probes in bioimaging using the therapeutic monoclonal antibody trastuzumab (**Tras**).^77^ Trastuzumab (Herceptin) was the first HER2-targeted agent approved for clinical use in breast cancer patients.^78^ The human epidermal growth factor receptor 2 (HER2) is a tyrosin kinase receptor overexpressed in approximately 25% of human breast cancers and is relevant target for numerous therapeutic strategies.

Modified **BCN-Tras** (10 μg/mL) was incubated with live HER2-overexpressing SKBR3 cells for 1 h, followed by washing to remove excess conjugate. The cells were then treated with fluorogenic probes (2.5 μM) for 30 min without additional washing steps. Control cells were not incubated with **BCN-Tras**. The **Trz** ^**+**^ **SulfoCy3** probe successfully labeled **BCN-Tras**, enabling visualization of the HER2 receptor on the cell surface. In comparison, using **TzSulfoCy3** resulted in a more intense fluorescent signal, though it was accompanied by reduced specificity and a lower signal-to-noise ratio as shown in Figures 6B and S29. These experiments highlight the potential of the cell-impermeable probes for fluorogenic labeling of membrane-associated glycoconjugates and antigens with excellent SNR.

HaloTag technology is a widely used protein-labeling system in molecular biology applications.^79^ The principle behind this system involves fusing a protein of interest to HaloTag, a self-labeling enzyme that utilizes chloroalkane ligands to form a covalent bond within its active site.^80^ To assess the compatibility of the triazinium probes with this technology, we first tested the previously described **BCN-HaloTag** ligand.^81^ Unfortunately, this compound led to significant background labeling despite extensive washing steps even in control cells not expressing the HaloTag. Additionally, the instability of the compound made it difficult to handle it practically. Consequently, we switched to preparing **HaloTrz** ^**+**^ **Coum** ligand, which allows the covalent attachment of the quenched **Trz** ^**+**^ **Coum** to the HaloTag fusion protein (Figure 7A). We used an endoplasmic reticulum targeted (ER) HaloTag in our experiments to restrict the reaction to this cellular compartment. This has been achieved by fusing the ER retention sequence KDEL to the N-terminal leader sequence of human IgK signal peptide (MDMRVLAQLLGLLLLCFPGARC). U2OS cells expressing this ER-targeted HaloTag were treated with 2 μM of the **HaloTrz** ^**+**^ **Coum** ligand for 30 min. After removing excess reagent, 5 μM of **BCN-PEG3-NH2** ^45^ was added for 15 min. To our satisfaction, cells labeled with **HaloTrz** ^**+**^ **Coum** and the BCN probe exhibited bright fluorescent signals, whereas control cells showed no fluorescence. Colocalization with the commercially available ER Tracker Red confirmed that the labeling was specific to the ER compartment (Figures 7B and S35 and S36).

**Figure 7:**
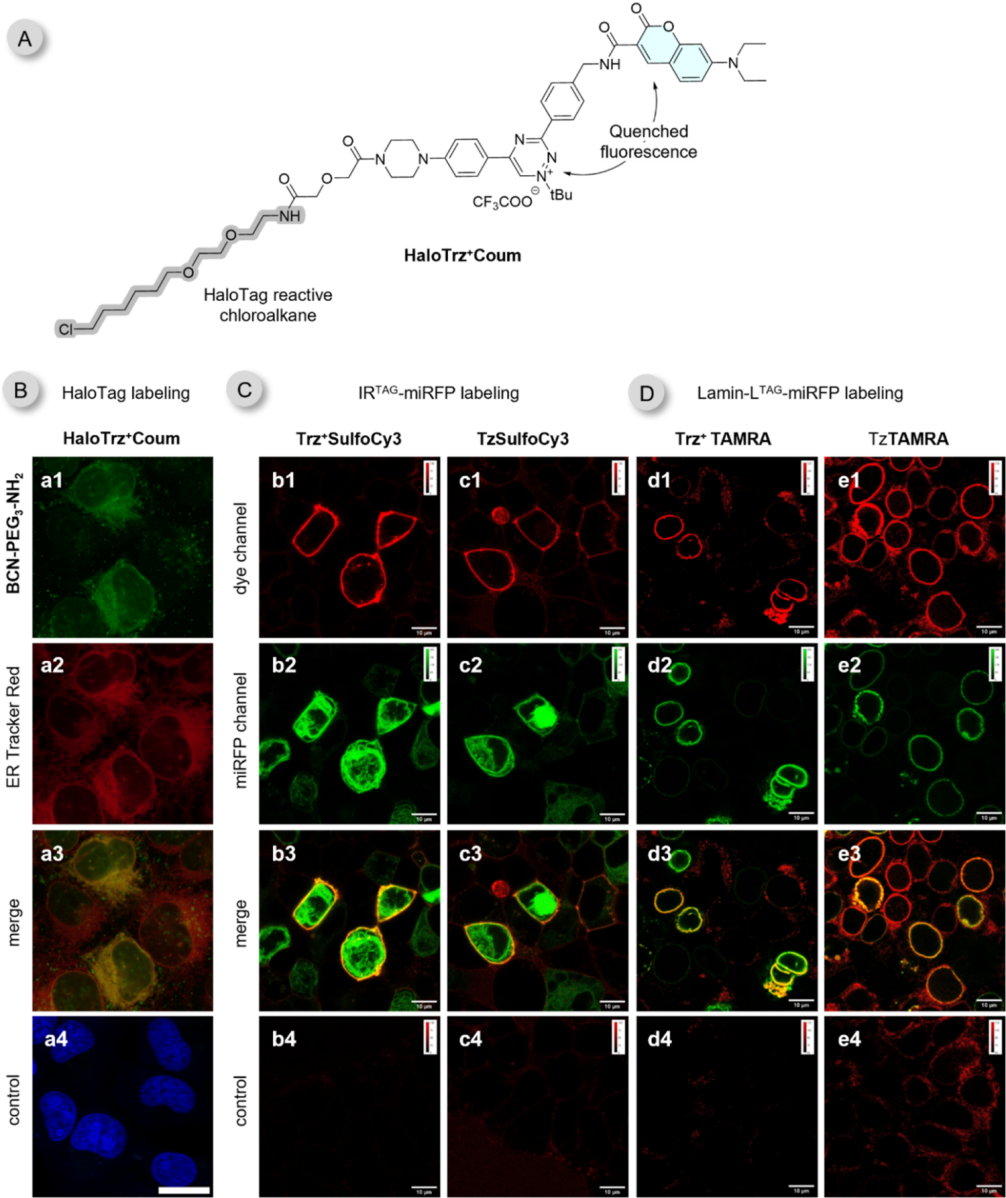
No-wash live cell labeling of extra- and intracellular target proteins. (A) Structure of **HaloTrz** ^**+**^ **Coum**. (B) Labeling of live U2OS cells expressing ER-targeted HaloTag. Conditions: cells were treated with **HaloTrz** ^**+**^ **Coum** (2 μM for 30 min) and after washing for 15 min with 5 μM **BCN-PEG3-NH2**. Colocalization was performed with ER-Tracker Red. Control includes cells treated only with **HaloTrz** ^**+**^ **Coum** (2 μM for 30 min). Cell nuclei were stained with DRAQ5 and are shown in blue. Further experiments can be found in SI (Figure S35 and S36). Scale bar is 20 μm. (C) Labeling of **IR** ^**TAG**^ **-miRFP** containing **BCN-Lys** on live HEK293T cells. Conditions: cells were treated with the indicated probe (0.1 μM for 30 min). (D) Labeling of **Lamin-L** ^**TAG**^ **-miRFP** containing **BCN-Lys** on live HEK293T cells. Conditions: cells were treated with the indicated probe (0.1 μM for 4 h). Dye channel is shown in red and miRFP in green. Controls include cells not expressing the **BCN-Lys** modified proteins and treated with fluorogenic probes. Scale bar is 10 μm.

Another widely used strategy for site-specific protein labeling is amber stop codon suppression.^82,83^ This method enables the incorporation of artificial amino acids with diverse functional groups into proteins in engineered cells in response to the TAG codon. **BCN-Lys** is one such amino acid, facilitating the site-specific installation of BCN groups into proteins.^84,85^ We have prepared insulin receptor **IR** ^**TAG**^ **-miRFP** and **Lamin-L** ^**TAG**^ **-miRFP** fusion constructs to evaluate the triazinium probes in extra- and intracellular protein labeling schemes, respectively. In these constructs, **BCN-Lys** was incorporated into the extracellular domain of the **IR**, while in case of **Lamin**, it is situated intracellularly within a linker (**L**) between **Lamin** and the reporter protein **miRFP** (Figures 7C,7D and S37).

HEK293T cells transfected with **IR** ^**TAG**^ **-miRFP** were labeled with 0.1 μM cell-impermeable **Trz** ^**+**^ **SulfoCy3** or with **TzSulfoCy3** which was used for comparison. Following the reaction, the cells were subjected to confocal microscopy imaging without a preceding washing step (no-wash conditions). Images confirmed successful and specific labeling with both probes as suggested by the excellent colocalization with the miRFP signal (Figure 7c). At the same time, no labeling was observed in nontransfected control cells.

Cells expressing **Lamin-L** ^**TAG**^ **-miRFP**, were treated with 0.1 μM cell-permeable **Trz** ^**+**^ **TAMRA** or **TzTAMRA**. Again, cells were subjected to confocal microscopy imaging without a prior washing step to evaluate the effects of the markedly different fluorogenicities of the two probes. Both TAMRA probes demonstrated efficient and specific intracellular labeling of **Lamin**, which colocalized with miRFP. However, a significant difference in background fluorescence was observed between the two probes. The virtually nonfluorogenic tetrazine dye, showed a considerable nonspecific signal, which was even more pronounced in case of nontransfected cells (Figure 7e4). These experiments highlight the successful application of the new fluorogenic triazinium probes in two widely used systems, HaloTag and amber codon suppression, enabling specific protein labeling in live cells under no-wash conditions.

Next, we evaluated the fluorogenic triazinium probes in metabolic incorporation studies. The incorporation of sialic acids bearing clickable groups into cellular glycoconjugates, known as metabolic glycoengineering (MGE), is a powerful strategy for probing the complex structure and function of this critical class of biomolecules.^86–88^ A sialic acid analog bearing BCN group (**BCN-Sia**) has previously been used to image glycans in live zebrafish.^89^ We aimed to utilize **BCN-Sia** in our studies to assess whether the cell-permeable and cell-impermeable probes could selectively visualize glycoconjugates on the cell surface and within live cells. Additionally, we sought to determine whether the two heterodienes, Trz^+^ and Tz, could be combined or if a preferential pairing would be more effective in this system.

Our experiments began by growing U2OS cells in the presence of 1 mM **BCN-Sia** for 48 h to allow its incorporation into glycoconjugates. Cells grown without **BCN-Sia** served as controls. After washing the excess sugar, a cell-impermeable probe (2.5 μM) was applied for 30 min. The medium was then replaced with one containing 1 μM of the cell-permeable probe for an additional 15 min, and the cells were imaged using confocal microscopy. Several combinations of cell-permeable and cell-impermeable probes successfully labeled the respective glycoconjugates (Figure S38). Overall, we observed that in these experiments, the Tz probes were slightly more efficient for extracellular labeling, while the triazinium probes provided better intracellular signals. One of the most effective combinations for dual labeling included the use of **TzSulfoCy3** derivative for cell membrane glycoconjugates and the **Trz** ^**+**^ **Coum** probe for intracellular pool of the glycoconjugates (Figure 8).

**Figure 8:**
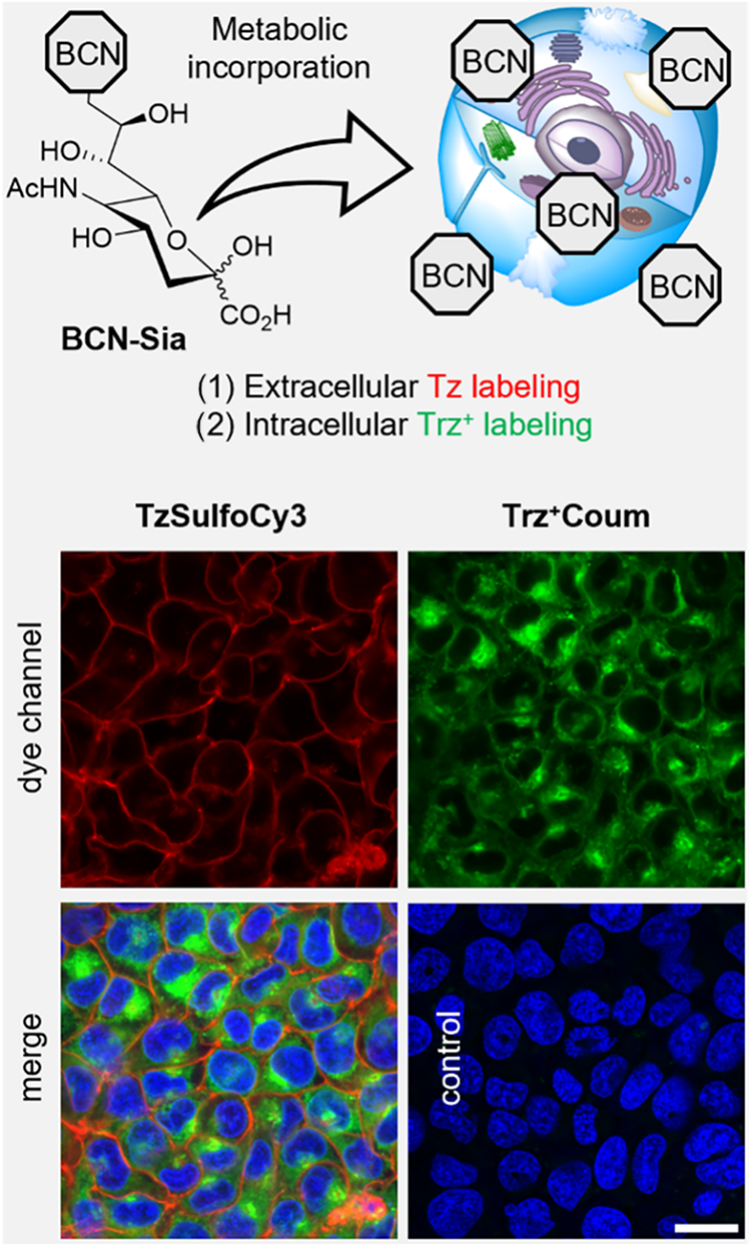
Example of dual no-wash live cell labeling of metabolically incorporated **BCN-Sia**. U2OS cells were treated with **BCN-Sia** (1 mM for 48 h), washed and subsequently labeled with 2.5 μM **TzSulfoCy3** for 30 min, then for 15 min with 1 μM **Trz** ^**+**^ **Coum**. Control cells were not fed with **BCN-Sia** but were treated with **TzSulfoCy3** and **Trz** ^**+**^ **Coum**. Intracellular click labeling is shown in green and extracellular in red. Cell nuclei were stained with DRAQ5 and are shown in blue. Pictures were acquired without additional washing steps. Further experiments are shown in the SI (Figure S38). Scale bar 20 μm.

These experiments further demonstrate the versatility of the fluorogenic triazinium probes in labeling important extracellular and intracellular biomolecules under no-wash conditions in live cells. Importantly, our toxicity studies did not show any significant impact of the probes on cell viability under the experimental conditions (Table S15 and Graph S2).

## Conclusion

In this work, we introduce the triazinium core as a highly effective fluorescence-quenching moiety, which can be bioorthogonally activated in reactions with the strained dienophile BCN, leading to efficient fluorescence restoration. The key triazinium-fluorophore conjugates can be synthesized from simple starting materials, enabling easy access to a range of fluorogenic Trz^+^ probes that span the entire visible spectrum. We characterized the photophysical properties of these probes and performed detailed computational studies that revealed their unique fluorescence quenching mechanism. We identified the fluorescence quenching mechanism based on three key factors (i) high propensity of Trz^+^ for the formation of stacked nonemissive conformers, (ii) strong and broad-band absorption inducing large spectral overlap with fluorophores and (iii) electron-poor nature of their π-systems allowing for PeT. All these factors can efficiently suppress the emission of a broad range of fluorophores. By comparing the new triazinium probes with equivalent tetrazines, we demonstrate the universal applicability of the Trz^+^ fluorophores in various bioimaging applications. These include imaging of small molecules, such as organelle-targeted probes and drugs, cell surface antigens, HaloTag fusion proteins, proteins containing artificial amino acids, and metabolically engineered glycoconjugates. All experiments were conducted in live cells under no-wash conditions. Trz^+^ probes outperformed their Tz counterparts in many cellular experiments, particularly in labeling intracellular targets, where many Tz probes failed or provided significantly lower signal-to-noise ratios. Given their synthetic accessibility and excellent performance in cells, we believe that these new fluorogenic triazinium probes represent an important addition to bioorthogonally activatable fluorescence-quenching moieties, supporting the expanding use of these tools in diverse applications.

## Supplementary Material







## Notes

Synthetic procedures, characterization data of all new compounds (^1^H, ^13^C NMR and HR MS), cell labeling studies, photophysical and fluorescence measurements, computational studies and additional cellular experiments (PDF)
Copies of NMR spectra (PDF)
XYZ coordinates of the calculated structures (ZIP)
