# Supplementary material for: Fluorescence Quenching Properties and Bioimaging Applications of Readily Accessible Blue to Far-Red Fluorogenic Triazinium Salts
Source: J Am Chem Soc. 2025 Dec 28;148(1):1183–96. doi: 10.1021/jacs.5c17428 (PMC12814330; doi:10.1021/jacs.5c17428)
Supplement: Supplementary file 1 [file ja5c17428_si_001.pdf]

Fluorescence Quenching Properties and Bioimaging Applications of Readily Accessible Blue to Far-Red Fluorogenic Triazinium Salts

Veronika Šlachtová,[a] Daniel Bím,[a,b] Krisztina Németh,[c] Eliza Barańska,[a] Simona Bellová,[a] Martin Dračínský,[a] Rastislav Dzijak,[a] Péter Kele,[c] Tomáš Slanina,[a]* and Milan Vrabel[a]*

[a] Institute of Organic Chemistry and Biochemistry of the CAS, Flemingovo nám. 2, 16000, Prague, Czech Republic. *Email: slanina@uochb.cas.cz, vrabel@uochb.cas.cz.

[b] Department of Physical Chemistry, University of Chemistry and Technology, Prague, Technická 5, 166 28 Prague, Czech Republic

[c] Chemical Biology Research Group, Institute of Organic Chemistry, HUN-REN Research Centre for Natural Sciences, Magyar Tudósok Krt. 2., 1117 Budapest, Hungary

Table of Contents

General information 3

Source of commercial chemicals 3

Instrumentation 4

Synthetic procedures 4

Synthesis of precursor **Trz$^+$s** 5

Initial pathway **(A)** toward **(Trz$^+$1)** 5

Improved alternative pathway **(B)** toward **(Trz$^+$1)** 6

Synthesis of fluorogenic conjugates 8

Synthesis of **HaloTrz$^+$Coum** ligand 16

Synthesis of pyridazinedione-BCN rebridging reagent 22

Synthesis of modified Dasatinib drug 24

HPLC-MS analysis of Trz$^+$Fluorophores and the respective click products with *endo*-BCN-OH 26

Normalized absorption spectra 28

Absorption spectra 34

Determination of extinction coefficients 39

Fluorescence turn-on measurements 39

Fluorescence time-lapse measurements 47

Fluorescence quantum yield determination 52

Spectroscopy experiments 52

Methods and experimental setup 52

Measured spectra 53

Computational Details 62

Conformational sampling and density functional theory calculations 62

Gibbs free energy calculations 62

Reduction potentials calculations 63

Example ORCA inputs 63

Optimized structures 63

Calculated properties of Trz$^+$/Tz-fluorophore conjugates 63

Reaction kinetics of Trz$^+$1 and Tz1 with *endo*-BCN-OH 70

HPLC-MS analysis of Trz$^+$1 stability under biologically relevant conditions 71

BCN-TPP mitochondrial labeling 73

Fluorescence intensity from BCN-TPP mitochondrial labeling 75

Additional BCN-TPP mitochondrial labeling 76
SKBR3 cells Herceptin labeling 77
Rebridging of Herceptin with BrPD-PEG3-BCN **(Herceptin-BrPD4)** 77
Modification of Herceptin with NHS-PEG3-BCN 77
SDS-PAGE analysis of Herceptin modification 78
Intact mass analysis of mAb Herpectin-PEG3-BCN conjugate 80
Herceptin cell labeling microscopy 80
BCN-ConA cellular labeling 81
Model drug's cellular labeling 82
Fluorescence intensity from BCN-drug labeling 87
Additional model drug's cellular labeling 87
ER HaloTag cellular labeling 89
ER HaloTag plasmid production 89
Preparation of recombinant lentiviral particles 89
Oligonucleotide sequences 89
Modification of U2OS cells with ER HaloTag plasmid 89
(A) Transduction 89
(B) Transfection 90
ER HaloTag cell labeling microscopy 90
Amber suppression 92
Cell culture 92
Bioorthogonal labeling of live cells 93
Confocal imaging and analysis 93
Double labeling of BCN-Sia after metabolic incorporation 95
Cytotoxicity screening 97
Literature 98

General information

Source of commercial chemicals

The chemicals were obtained from commercial suppliers (Merck, ABCR, Fluorochem) and were used without further purification. *endo*-BCN-PEG3-amine (CAS 1883512-27-3) and *endo*-BCN (CAS 1263166-90-0) was obtained from SiChem, 7-(diethylamino)coumarin-3-carboxylic acid NHS ester

(CAS 139346-57-9) from abcr GmbH, Oregon Green™ 488 CA, Succinimidyl Ester, 5-isomer (catalog number: O6147), ER-TrackerTM Red (BODIPY™ TR Glibenclamide) (catalog number E34250) from ThermoFisher, Cyanine3 NHS ester (CAS 2632339-91-2), TAMRA NHS ester, 5-isomer (CAS 150810-68-7), Sulfo-Cyanine3 NHS ester (CAS 1518643-34-9) from Lumiprobe, ATTO 495, Succinimidyl Ester (catalog number AD 495) from ATTO-Tec, Janelia Fluor 646, free acid (catalog number 6993) from Tocris, BDP FL MethylTetrazine (CAS 2042193-77-9), Cy3-MethylTetrazine (CAS 1801924-47-9), *endo*-BCN-PEG3-NHS (2101206-94-2) ester from BroadPharm, Geldanamycin and Dasatinib.HCl (CAS 302962-49-8) from MedChemExpress. All dry solvents were dried by activated molecular sieves (3 and 4 Å) and stored under nitrogen. Herceptin (150 mg, Roche) monoclonal antibody was purchased from Všeobecná Fakultní Nemocnice v Praze.

Instrumentation

Solutions were concentrated on a rotary evaporator *Heidolph* equipped with a PC3001 VARIOpro pump from *Vacuubrand*. Flash column chromatography was performed using silica gel 60 (0.040–0.063 mm). Column chromatography was carried out on silica gel 60A (particle size: 40-60 µm) from *Acros Organics*. Mixtures of solvents are each stated as volume fractions. A CombiFlash® Rf+ from Teledyne ISCO or puriFlash® 5.250 from Interchim was used for flash column chromatography. ^{1}H, ^{13}C, and ^{19}F NMR spectra were measured at ambient temperature using 5 mm diameter NMR tubes. ^{13}C NMR spectra were proton decoupled Chemical shifts δ are quoted in ppm in relation to the chemical shift of the residual non-deuterated solvent peak ($CDCl_3$: δ(^{1}H) = 7.26, δ(^{13}C) = 77.2; MeOH-d_4: δ(^{1}H) = 3.31, δ(^{13}C) = 49.0; DMSO-d_6: δ(^{1}H) = 2.50; δ(^{13}C) = 39.4; CD_3CN-d_3: δ(^{1}H) = 1.94, δ(^{13}C) = 1.32). Coupling constants (*J*) are reported in Hertz. NMR spectra were recorded at ambient temperature (21 °C) in DMSO-d_6 or $CDCl_3$. Abbreviations in NMR spectra: app: apparent, br s: broad singlet, s: singlet, d: doublet, t: triplet, q: quartet, p: pentet, h: heptet, m: multiplet. ^{1}H- and ^{13}C-NMR spectra were measured at rt (25 °C) on a Bruker Avance III™ HD 400 MHz NMR system equipped with Prodigy cryo-probe or on a Bruker Avance III™ 500 MHz NMR spectrometer (^{1}H at 500.0 MHz, ^{13}C at 125.7 MHz). High-resolution mass spectra were recorded on an *Agilent* 5975C MSD Quadrupol or LTQ Orbitrap XL from *Thermo Fisher Scientific*. Thin-layer chromatography was performed on aluminum sheets from *Merck* (silica gel 60 F254, 20 × 20 cm). Visualization was accomplished with UV light (λ = 254 nm). UV/VIS spectroscopy was performed on a Cary 60 UV/VIS spectrophotometer from *Agilent Technologies*. The reaction progress was followed by HPLC-MS measurements on an LCMS-2020 system from *Shimadzu* equipped with a CORTECS C18 column (2.7 µm, 50 × 4.6 mm) or XSelect from Waters, C18, 4.6x50 mm, 2.5 µm. A gradient of MeCN + 0.05% HCOOH in water + 0.05% HCOOH was used for the analysis. Fluorescence measurements were performed on a Duetta spectrofluorometer (Horiba) or FluoMax4 spectrofluorometer (Jobin Yvon, Horiba). Cellular imaging experiments were performed by ZEISS LSM 980 confocal microscope (40×W objective). PAGE gel was scanned by fluorescence imaging using Amersham Typhoon Gel Scanner (Cytiva). Intact mass analysew acquired on Cyclic-IMS instrument and deconvoluted using MaxEnt1 algorithm.

Synthetic procedures

Synthesis and characterization of the following reagents were reported previously:

SMeTrz1,[1] SMeTrz2,[1] BODIPY-Ac1,[2] HaloTag-BCN,[3], HaloTag ligand,[4] *endo*-BCN-Sia,[5] TzCoum,[6] TzTAMRA,[6].

Synthesis of precursor **Trz$^+$s**

Initial pathway **(A)** toward **(Trz$^+$1)**

Scheme S1: Schematic overview of **Trz$^+$1** synthetic pathway (A)

SMeTrz2,[1] (0.700 mmol), boronic acid (2.0 equiv.), copper(I) thiophene-2-carboxylate (2.2 equiv.) and $Pd(PPh_3)_4$ (10 mol%) were placed in an argon flushed flask and anhydrous 1,4-dioxane (5 mL) was added. The mixture was stirred under argon at 95 °C for 12 h. After cooling to rt, the mixture was diluted with DCM (100 mL/mmol) and washed subsequently with 2 M aq. NaOH (70 mL) and sat. aq. NH_4Cl (70 mL). The organic phase was dried over Na_2SO_4, filtered, and concentrated under reduced pressure. The crude product was purified by silica gel column chromatography in EA/DCM gradient 5:95 → 20:80.

***tert*-Butyl (4-(5-(4-(piperidin-1-yl)phenyl)-1,2,4-triazin-3-yl)benzyl)carbamate (PipTrz1)**

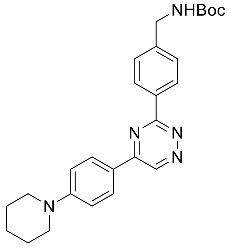

Yield: 240.0 mg (0.539 mmol, 77%) of an orange amorphous solid.

^{1}H NMR (401 MHz, $CDCl_3$): δ 9.45 (s, 1H), 8.62 – 8.55 (m, 2H), 8.22 – 8.16 (m, 2H), 7.45 (d, *J* = 8.6 Hz, 2H), 7.04 – 6.96 (m, 2H), 4.93 (br s, 1H), 4.42 (d, *J* = 5.5 Hz, 2H), 3.41 (t, *J* = 5.4 Hz, 4H), 1.77 – 1.63 (m, 6H), 1.48 (s, 9H).

^{13}C NMR (101 MHz, $CDCl_3$): δ 156.0, 154.8, 154.4, 143.5, 142., 140.4, 134.9, 129.2 (2×CH), 128.7, 127.8, 121.7, 114.6 (2×CH), 79.7, 48.8, 44.6, 28.5, 25.51, 24.5.

HRMS (ESI): m/z calcd. for $C_{26}H_{32}O_2N_5$ $[M+H]^+$ 446.25505, found 446.25519.

3-(4-(Ammoniomethyl)phenyl)-1-(*tert*-butyl)-5-(4-(piperidin-1-yl)phenyl)-1,2,4-triazin-1-ium 2,2,2-trifluoroacetate (Trz$^+$1)

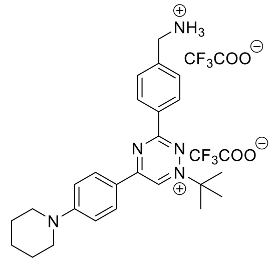

PipTrz1 (1.0 equiv., 0.539 mmol, 240.0 mg) was dissolved in dry DCM (5.3 mL) under argon and cooled to 0 °C. Isobutene gas was condensed in a Schlenk tube at -78 °C (dry ice/acetone bath). Subsequently, an excess of liquid isobutene (800 µL) was added using pre-chilled syringe followed by dropwise addition of trifloromethanesulfonic acid (3.3 equiv., 1.778 mmol, 157.0 µL) at 0 °C and the mixture was stirred at rt for 3 h. The crude product was concentrated by stream of air and purified by RP flash chromatography (RediSep Gold C18Aq 50 g column) in two portions. MeCN/H_2O (+ 0.1% TFA) gradient (flow rate 35 mL/min) 0:100 → 40:60 for 20 min. Product detection at λ = 525 and 410 nm, lyophilization.

Yield: 121.0 mg (0.192 mmol, 36%) of a dark red amorphous solid.

Note: A fraction containing **Trz$^+$1** was also in the mixture with the deprotected parent triazine isolated (m = 100 mg). After repetition of the alkylation procedure with this portion, additional **Trz$^+$1** was obtained: 74.0 mg (0.118 mmol, 22%) of a dark red amorphous solid.

^{1}H NMR (400 MHz, CD_3CN): δ 9.31 (s, 1H), 8.55 – 8.50 (m, 2H), 8.38 – 8.34 (m, 2H), 8.29 (s, 2H), 7.75 – 7.70 (m, 2H), 7.09 – 7.04 (m, 2H), 4.25 (s, 2H), 3.62 (t, *J* = 5.5 Hz, 4H), 1.87 (s, 9H), 1.77 – 1.63 (m, 6H).

^{19}F NMR (376 MHz, CD_3CN): δ -76.29, -79.30.

^{13}C NMR (101 MHz, CD_3CN): δ 166.4, 163.6, 156.6, 139.9, 135.6, 134.1, 132.9 (2×CH), 130.9 (2×CH), 130.0 (2×CH), 118.8, 114.4 (2×CH), 77.0, 48.9, 43.7, 28.6 (3×CH_3), 26.3, 25.0.

HRMS (ESI): m/z calcd. for $C_{25}H_{33}N_5$ [M+H^{2+}] 201.63625, found 201.63620

Improved alternative pathway **(B)** toward **(Trz$^+$1)**

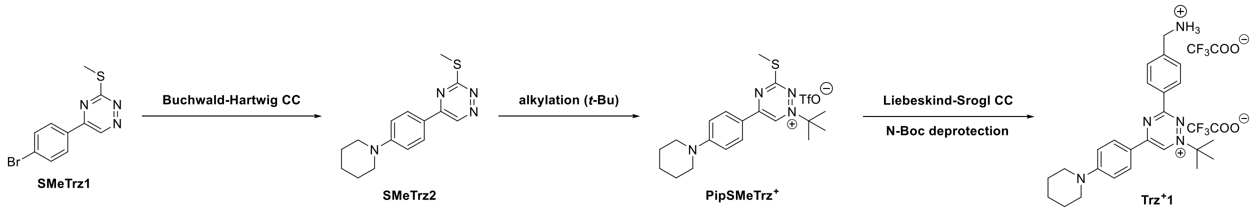

Scheme S2: Schematic overview of **Trz$^+$1** synthetic pathway (B)

Representative procedure A: SMeTrz1,[1] piperidine (1.1 equiv.), Cs_2CO_3 (2.8 equiv.), XPhos (0.3 equiv.) and $Pd_2(dba)_3$ (10 mol%) were placed in an argon-flushed flask and anhydrous 1,4-dioxane (10.0 mL/mmol) was added. The mixture was stirred under argon at 95 °C for 14 h. After cooling to rt, it was diluted with DCM (20 mL), filtered through Celite® 545 and washed with (1:4) EA/DCM (3×20

mL), and concentrated under reduced pressure. The crude product was purified by silica gel column chromatography (the solvent system is indicated below).

From **SMeTrz1** (1.766 mmol, 500.0 mg). Purification: (flash (Combi*Flash*® Rf+) column chromatography on silica (150 g) column (DCM/EtOAc gradient 100:0 → 50:50), (flow rate 60 mL/min) for 60 min. Product detection at λ =254 and 280 nm.

Yield: 390.0 mg (1.362 mmol, 77%) of a dark orange amorphous solid.

Representative procedure B: SMeTrz2 was dissolved in dry DCM (10.0 mL/mmol) under argon and cooled to 0 °C. Isobutene gas was condensed in a Schlenk tube at -78 °C (dry ice/acetone bath). Subsequently, an excess of liquid isobutene (500 µL/mmol) was added using pre-chilled syringe followed by dropwise addition of trifloromethanesulfonic acid (2.0 equiv.) at 0 °C and the mixture was stirred at rt for 2 h Crude product was concentrated by stream of air and purified by RP flash chromatography and lyophilized.

1-(*tert*-Butyl)-3-(methylthio)-5-(4-(piperidin-1-yl)phenyl)-1,2,4-triazin-1-ium

2,2,2-trifluoroacetate (PipSMeTrz$^+$)

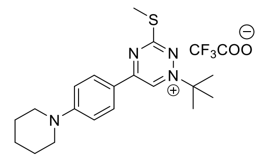

From **SMeTrz2** (1.362 mmol, 390.0 mg). Purification: (Purification: (RediSep Gold C18Aq 150 g column). MeCN/H_2O (+ 0.1% TFA) gradient (flow rate 60 mL/min) 10:90 → 80:20 for 20 min. Product detection at λ = 285 and 522 nm.

Yield: 536.0 mg (1.088 mmol, 80%) of a dark violet amorphous solid.

^{1}H NMR (400 MHz, CD_3CN): δ ^{1}H NMR (400 MHz, CD_3CN) δ 9.09 (s, 1H), 8.23 – 8.17 (m, 2H), 7.06 – 7.00 (m, 2H), 3.63 – 3.59 (m, 4H), 2.68 (s, 3H), 1.77 (s, 9H), 1.72 – 1.63 (m, 6H).

^{19}F NMR (376 MHz, CD_3CN): δ -76.56.

^{13}C NMR (101 MHz, CD_3CN): δ ^{13}C NMR (101 MHz, CD_3CN): δ 178.1, 161.9, 156.6, 133.8, 132.9 (2×CH), 117.6 (overlapped with deuterated solvent), 114.4 (2×CH), 76.8, 48.9, 28.5 (3×CH_3), 26.3, 25.0, 14.4.

HRMS (ESI): m/z calcd. for $C_{19}H_{27}N_4S$ [M$^+$] 343.19504, found 343.19509.

Representative procedure C: PipSMeTrz$^+$, boronic acid (2.0 equiv.), copper(I) thiophene-2-carboxylate (2.2 equiv.), and $Pd(PPh_3)_4$ (10 mol%) were placed in an argon flushed flask and anhydrous 1,4-dioxane (7.0 mL/mmol) was added. The mixture was stirred under argon at 60 °C for 2-4 h. After cooling to rt, the mixture was diluted with DCM (100 mL/mmol) and washed with sat. aq. $NaHCO_3$ (100 mL/mmol). The organic phase was dried over Na_2SO_4, filtered, and concentrated under reduced pressure. The residue was then re-dissolved in (9:1) MeCN/H_2O (+ 0.1% TFA) (100.0 mL/mmol), filtered through a pad of sand/C18® (Santiago; 230-400 MESH) and washed with (9:1)

MeCN/H_2O (+ 0.1% TFA) (3 × 20.0 mL/mmol). The filtrate was concentrated under reduced pressure and lyophilized. The crude product was purified by RP flash chromatography (the conditions are shown for each derivative separately) and lyophilized.

PipSMeTrz$^+$ (1.088 mmol, 536.0 mg) was used. Purification: (RediSep Gold C18Aq 150 g column). MeCN/H_2O gradient (flow rate 60 mL/min) 10:90 → 80:20 for 20 min. Product detection at λ = 285 and 522 nm.

Yield: 536.0 mg (1.088 mmol, 80%) of a dark violet amorphous solid.

***N*-Boc Deprotection**: To a solution of **N-BocTrz$^+$1** (504.0 mg, 0.818 mmol) in DCM (50.0 mL), excess of TFA (6.0 mL) was added dropwise at rt. The mixture was stirred at rt for 3 h. It was then concentrated under reduced pressure and purified by RP flash chromatography (RediSep Gold C18Aq 150 g column). MeCN/H_2O (+ 0.1% TFA) gradient (flow rate 60 mL/min) 5:95 → 35:65 for 20 min. Product detection at λ = 527 and 435 nm, lyophilization.

Yield: 373.0 mg (0.593 mmol, 72%) of a dark red solid.

Synthesis of fluorogenic conjugates

1-(*tert*-Butyl)-3-(4-((7-(diethylamino)-2-oxo-2*H*-chromene-3-carboxamido)methyl)phenyl)-5-(4-(piperidin-1-yl)phenyl)-1,2,4-triazin-1-ium 2,2,2-trifluoroacetate (Trz$^+$Coum)

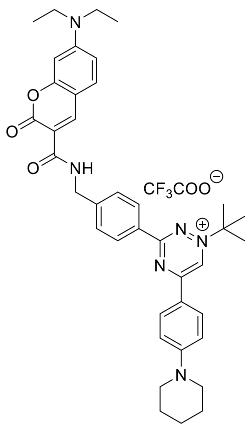

Trz$^+$1 (1.0 equiv., 12.9 mg, 0.020 mmol) and commercial 7-(diethylamino)coumarin-3-carboxylic acid NHS ester (1.1 equiv., 8.1 mg, 0.023 mmol) were dissolved in dry MeCN (510 µL) and the solution was cooled to 0 °C. DIPEA (5.0 equiv., 18.0 µL, 0.102 mmol) was added dropwise. After 3 h of stirring at rt, the reaction was diluted with (1:1) MeCN/H_2O (4.49 mL) mixture, acidified with TFA (10.0 equiv., 16.0 µL, 0.205 mmol), and the crude product was purified by preparative HPLC (Arion Plus (21.2 × 250 mm, 5 µm) column). MeCN/H_2O (+ 0.1% TFA) gradient (flow rate 15 mL/min) 40:60 → 95:5 for 25 min. Product detection at λ = 422 and 522 nm, lyophilization.

Yield: 6.0 mg (0.008 mmol, 39%) of a dark red solid.

^{1}H NMR (401 MHz, CD_3CN): δ 9.29 (s, 1H), 9.20 (t, *J* = 6.1 Hz, 1H), 8.64 (d, *J* = 0.7 Hz, 1H), 8.49 – 8.42 (m, 2H), 8.38 – 8.30 (m, 2H), 7.60 – 7.55 (m, 2H), 7.52 (d, *J* = 9.0 Hz, 1H), 7.10 – 6.99 (m, 2H), 6.77 (dd, *J* = 9.0, 2.6 Hz, 1H), 6.55 (dd, *J* = 2.4, 0.7 Hz, 1H), 4.68 (d, *J* = 6.1 Hz, 2H), 3.63 – 3.56 (m, 4H), 3.48 (q, *J* = 7.2 Hz, 4H), 1.86 (s, 9H), 1.77 – 1.63 (m, 6H), 1.19 (t, *J* = 7.1 Hz, 6H).

^{19}F NMR (377 MHz, CD_3CN): δ -76.59.

^{13}C NMR (101 MHz, CD_3CN): δ 166.7, 164.2, 163.6, 163.5, 158.8, 156.5, 153.9, 148.9, 146.9, 135.3, 132.8 (2×CH), 132.4, 132.3, 129.9 (2×CH), 129.0 (2×CH), 118.8, 114.4 (2×CH), 111.2, 110.7, 109.0, 97.0, 76.9, 48.9, 45.6, 43.6 (CONH<u>C</u>H$_2$), 28.6 (3×CH_3), 26.3, 25.0, 12.6 (2×CH_3).

HRMS (ESI): m/z calcd. for $C_{39}H_{45}O_3N_6$ [M^+] 645.35477, found 645.35418.

1-(*tert*-Butyl)-3-(4-((3-(5,5-difluoro-7,9-dimethyl-5*H*-5l4,6l4-dipyrrolo[1,2-c:2',1'-f][1,3,2]diazaborinin-3-yl)propanamido)methyl)phenyl)-5-(4-(piperidin-1-yl)phenyl)-1,2,4-triazin-1-ium 2,2,2-trifluoroacetate (Trz$^+$BODIPY)

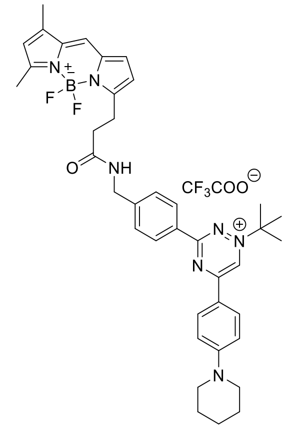

Trz$^+$1 (1.0 equiv., 14.2 mg, 0.022 mmol), HATU (1.5 equiv., 12.8 mg, 0.034 mmol), and **BODIPY-FL**[2] (1.0 equiv., 6.6 mg, 0.022 mmol) were dissolved in dry MeCN (800 µL) and the solution was cooled to 0 °C. Anhydrous DMF (200 µL) was then added to better solubilize the reagents. DIPEA (7.0 equiv., 20.0 µL, 0.112 mmol) was added dropwise. After 3 h of stirring at rt, the reaction was concentrated under reduced pressure and the crude product was purified by flash (Combi*Flash*® Rf+) column chromatography on pre-packed (*Interchim*) silica (25 g) column (DCM/MeOH (+ 0.1% TFA) gradient 98:2 →96:4), including wash with (90:10) MeCN/H_2O (+ 0.1% TFA) isocratic (flow rate 35 mL/min) for 30 min to eluate the pre-purified product. Product detection at λ =254 and 503 nm. After concentration, this crude product was re-purified by preparative HPLC (Arion Plus (21.2 × 250 mm, 5 µm) column). MeCN/H_2O (+ 0.1% TFA) gradient (flow rate 15 mL/min) 35:65 → 95:5 for 30 min. Product detection at λ = 503 and 285 nm, lyophilization.

Yield: 5.0 mg (0.006 mmol, 28%) of a dark brown solid.

^1H NMR (500 MHz, DMSO-d_6): δ 9.74 (s, 1H), 8.65 – 8.58 (m, 1H), 8.53 – 8.47 (m, 2H), 8.46 – 8.40 (m, 2H), 7.71 (s, 1H), 7.54 (d, *J* = 8.6 Hz, 2H), 7.20 – 7.15 (m, 2H), 7.11 (d, *J* = 4.0 Hz, 1H), 6.39 (d, *J* = 4.0 Hz, 1H), 6.31 (s, 1H), 4.43 (d, *J* = 6.0 Hz, 2H), 3.62 (t, *J* = 5.4 Hz, 3H overlapped with signal of residual CF_3COO^- counterion), 3.14 (t, *J* = 7.8 Hz, 2H), 2.62 (t, *J* = 7.8 Hz, 2H), 2.48 (s, 3H overlapped with signal of residual H_2O), 2.26 (s, 3H), 1.85 (s, 9H), 1.71 – 1.59 (m, 6H).

^{19}F NMR (470 MHz, DMSO-d_6): δ -73.88, -143.03, -143.10, -143.17, -143.24.

^{13}C NMR (126 MHz, DMSO-d_6): δ 171.1, 164.2, 162.6, 159.2, 157.6, 155.0, 145.5, 144.2, 134.5, 132.9, 132.0, 131.18, 128.8, 128.4 (2×CH), 128.0 (2×CH), 125.3, 120.3, 118.3, 116.6, 113.3 (2×CH), 75.1, 47.5, 41.9 (CONH$\underline{C}$H$_2$), 33.7, 28.0 (3×CH$_3$), 25.2, 24.0, 23.9, 14.5 (1×CH$_3$), 11.0 (1×CH$_3$).

HRMS (ESI): m/z calcd. for $C_{39}H_{45}ON_7BF_2$ [M$^+$] 676.37412, found 676.37403.

1-(*tert*-Butyl)-3-(4-((3-carboxy-4-(2,7-difluoro-6-hydroxy-3-oxo-3*H*-xanthen-9-yl)benzamido)methyl)phenyl)-5-(4-(piperidin-1-yl)phenyl)-1,2,4-triazin-1-ium 2,2,2-trifluoroacetate (Trz$^+$OG)

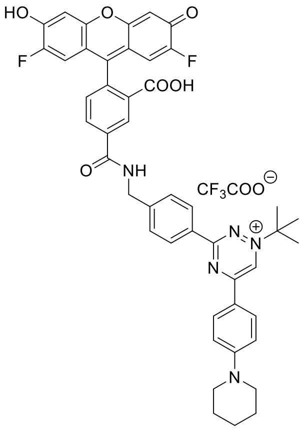

Trz$^+$1 (1.0 equiv., 6.8 mg, 0.011 mmol) and commercial Oregon Green™ 488 Carboxylic Acid, Succinimidyl Ester, 5-isomer (1.0 equiv., 5.0 mg, 0.010 mmol) were dissolved in dry MeCN (450 µL) and the solution was cooled to 0 °C. DIPEA (7.0 equiv., 12.0 µL, 0.069 mmol) was added dropwise. After 2 h of stirring at rt, more DIPEA (7.0 equiv., 12.0 µL, 0.069 mmol) was added for additional consumption of the remaining NHS ester and stirring continued for 4 h. The reaction was diluted with (2:1) MeCN/H_2O (4.55 mL) mixture, neutralized with TFA (14.0 equiv., 11.0 µL, 0.137 mmol) and the crude product was purified by preparative HPLC (Arion Plus (21.2 × 250 mm, 5 µm) column). MeCN/H_2O (+ 0.1% TFA) gradient (flow rate 15 mL/min) 20:80 → 75:25 for 35 min. Product detection at λ = 495 and 280 nm, lyophilization.

Yield: 3.5 mg (0.004 mmol, 39%) of a dark red solid.

^{1}H NMR (500 MHz, DMSO-d_6): δ 10.87 (br s, 2H), 9.75 (s, 1H), 9.60 (t, J = 6.1 Hz, 1H), 8.55 (d, J = 2.0 Hz, 1H), 8.53 – 8.49 (m, 2H), 8.48 – 8.44 (m, 2H), 8.33 (dd, J = 8.1, 1.5 Hz, 1H), 7.66 (d, J = 8.5 Hz, 2H), 7.44 (d, J = 8.1 Hz, 1H), 7.20 – 7.14 (m, 2H), 6.93 (d, J = 7.5 Hz, 2H), 6.59 (d, J = 11.3 Hz, 2H), 4.68 (d, J = 6.0 Hz, 2H), 3.65 – 3.58 (m, 4H overlapped with signal of residual CF_3COO^- counterion), 2.08 (s, 4H), 1.85 (s, 9H), 1.69 (p, J = 5.6 Hz, 2H), 1.65 – 1.59 (m, 4H).

^{19}F NMR (470 MHz, DMSO-d_6): δ -73.81, -139.80 (dd, J = 11.3, 7.8 Hz).

^{13}C NMR (126 MHz, DMSO-d_6) δ 167.8, 164.5 (d, $^1J_{C,F}$ = 73.7, 2×C-F), 162.6, 157.8 (d, $^2J_{C,F}$ = 32.3, 2×C-OH), 155.0, 154.0, 148.8, 147.7 (d, $^3J_{C,F}$ = 14.1, 2×C), 147.3, 146.9, 145.2, 136.2, 136.04, 134.8, 132.0 (2×CH), 131.3, 128.5 (2×CH), 128.1 (2×CH), 126.4, 125.0 (from 2D spectra), 124.0 (d, $^2J_{C,F}$ = 31.1, 2×CH), 113.9 (d, $^3J_{C,F}$ = 20.7, 2×CH), 118.3, 113.3 (2×CH), 107.7 (d, $^4J_{C,F}$ = 6.0, 2×CH), 104.8, 82.4, 75.1, 47.5, 42.7 (CONH$\underline{C}$H$_2$), 28.0 (3×CH$_3$), 25.2, 23.9.

HRMS (ESI): m/z calcd. for $C_{46}H_{40}O_6N_5F_2$ [M^+] 796.29412, found 796.29386.

10-(4-((4-(1-(*tert*-Butyl)-5-(4-(piperidin-1-yl)phenyl)-1,2,4-triazin-1-ium-3-yl)benzyl)amino)-4-oxobutyl)-3,6-bis(dimethylamino)acridin-10-ium 2,2,2-trifluoroacetate (Trz$^+$ATTO495)

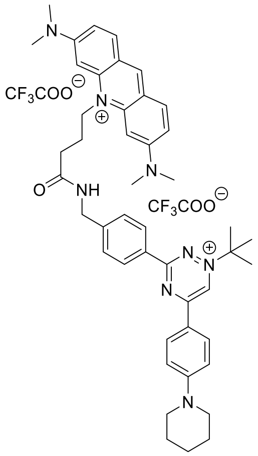

Trz$^+$1 (1.0 equiv., 6.3 mg, 0.010 mmol) and commercial ATTO 495, Succinimidyl Ester (1.0 equiv., 5.0 mg, 0.009 mmol) were dissolved in dry MeCN (450 µL) and the solution was cooled to 0 °C. DIPEA (7.0 equiv., 11.0 µL, 0.064 mmol) was added dropwise. After 1 h of stirring at rt, more DIPEA (7.0 equiv., 11.0 µL, 0.064 mmol) and HATU (1.2 equiv., 4.2 mg, 0.011 mmol) were added for an additional consumption of the remaining free acid and stirring continued for 4 h. The reaction was diluted with (2:1) MeCN/H_2O (4.55 mL) mixture, neutralized with TFA (14.0 equiv., 10.0 µL, 0.128 mmol) and the crude product was purified by preparative HPLC (Arion Plus (21.2 × 250 mm, 5 µm) column). MeCN/H_2O (+ 0.1% TFA) gradient (flow rate 15 mL/min) 5:95 → 85:15 for 30 min. Product detection at λ = 500 and 270 nm, lyophilization.

Yield: 3.5 mg (0.004 mmol, 40%) of a dark red solid.

^{1}H NMR (500 MHz, CD_3CN): δ 9.32 (s, 1H), 8.56 (s, 1H), 8.48 – 8.42 (m, 2H), 8.37 – 8.31 (m, 2H), 7.85 (d, *J* = 9.4 Hz, 2H), 7.71 (t, *J* = 5.8 Hz, 1H), 7.62 – 7.56 (m, 2H), 7.19 (dd, *J* = 9.3, 2.2 Hz, 2H), 7.08 – 7.02 (m, 2H), 6.89 (d, *J* = 2.6 Hz, 2H), 4.73 – 4.63 (m, 2H), 4.54 (d, *J* = 6.0 Hz, 2H), 3.66 – 3.56 (m, 4H), 3.27 (s, 12H), 2.66 (t, *J* = 6.1 Hz, 2H overlapped with signal of residual CF_3COO^- counterion), 2.30 – 2.19 (m, 2H), 1.85 (s, 9H), 1.74 – 1.64 (m, 6H).

^{19}F NMR (470 MHz, CD_3CN): δ -76.03.

^{13}C NMR (126 MHz, CD_3CN) δ 173.4, 166.7, 163.6, 157.06, 156.5, 147.0, 144.0, 143.9, 133.9 (2×CH), 132.8 (2×CH), 132.3, 129.8 (2×CH), 129.1 (2×CH), 118.8, 118.0, 115.3 (2×CH), 114.3 (2×CH), 93.7 (2×CH), 76.9, 48.9, 48.2, 43.5 (CONH$\underline{C}$H$_2$), 41.1 (4×CH_3), 32.2, 28.6 (3×CH_3), 26.3, 25.0, 21.6.

HRMS (ESI): m/z calcd. for $C_{46}H_{56}ON_8$ [$M+H^{2+}$] 368.22831, found 368.22816.

1-(6-((4-(1-(*tert*-Butyl)-5-(4-(piperidin-1-yl)phenyl)-1,2,4-triazin-1-ium-3-yl)benzyl)amino)-6-oxohexyl)-3,3-dimethyl-2-((E)-3-((E)-1,3,3-trimethylindolin-2-ylidene)prop-1-en-1-yl)-3*H*-indol-1-ium 2,2,2-trifluoroacetate (Trz$^+$Cy3)

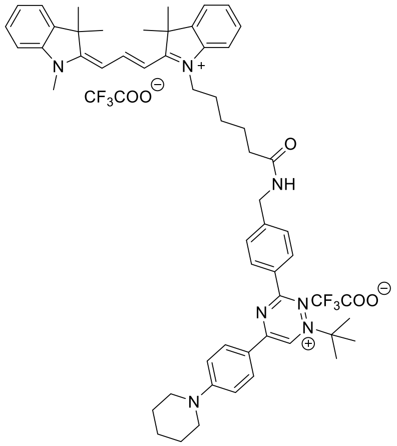

Trz⁺1 (1.0 equiv., 4.9 mg, 0.008 mmol) and commercial Cyanine3 NHS ester (1.0 equiv., 5.0 mg, 0.008 mmol) were dissolved in dry MeCN (360 µL) and the solution was cooled to 0 °C. DIPEA (10.0 equiv., 14.0 µL, 0.078 mmol) was added dropwise. After 1 h of stirring at rt, more DIPEA (10.0 equiv., 14.0 µL, 0.078 mmol) was then added for additional consumption of the remaining NHS ester, and stirring continued for 1 h. The reaction was diluted with 1:1 MeCN/H_2O (4.64 mL) mixture, acidified with TFA (20.0 equiv., 12.0 µL, 0.156 mmol), and the crude product was purified by preparative HPLC (Arion Plus (21.2 × 250 mm, 5 µm) column). MeCN/H_2O (+ 0.1% TFA) gradient (flow rate 15 mL/min) 30:70 → 85:15 for 30 min. Product detection at λ = 545 and 254 nm, lyophilization.

Yield: 5.5 mg (0.005 mmol, 68%) of a dark pink solid.

^{1}H NMR (500 MHz, DMSO-*d*$_6$): δ 9.18 (s, 1H), 7.96 – 7.91 (m, 3H), 7.88 – 7.84 (m, 2H), 7.78 (t, *J* = 13.5 Hz, 1H), 7.07 (ddd, *J* = 7.5, 2.9, 1.0 Hz, 2H), 6.98 – 6.93 (m, 2H), 6.91 – 6.86 (m, 4H), 6.75 – 6.71 (m, 2H), 6.63 – 6.58 (m, 2H), 5.93 (dd, *J* = 13.4, 7.2 Hz, 2H), 3.83 (d, *J* = 6.0 Hz, 2H), 3.56 (t, *J* = 7.6 Hz, 2H), 3.07 – 3.03 (m, 4H), 1.65 (t, *J* = 7.5 Hz, 2H), 1.52 (s, 3H), 1.28 (s, 9H), 1.22 – 1.17 (m, 3H), 1.15 – 1.11 (m, 15H), 1.09 – 1.03 (m, 6H).

^{19}F NMR (470 MHz, DMSO-*d*$_6$): δ -74.30.

^{13}C NMR (126 MHz, DMSO-*d*$_6$): δ 174.4, 173.6, 172.1, 164.2, 162.6, 155.0, 149.6, 145.6, 142.6, 141.9, 140.6, 140.5, 136.1, 132.0 (2×CH), 131.15, 128.6, 128.5, 128.4 (2×CH), 127.9 (2×CH), 125.2, 125.1, 118.3, 113.2 (2×CH), 111.5, 111.4, 102.9, 102.3, 75.1, 48.8, 47.5, 43.6, 41.8 (CONH$\underline{C}H_2$), 35.1, 31.3, 30.7, 28.0 (3×CH_3), 27.4 (2×CH_3), 27.2 (2×CH_3), 26.7, 25.8, 25.2, 24.9, 23.9.

HRMS (ESI): m/z calcd. for $C_{55}H_{67}ON_7$ [M+H^{2+}] 420.76981, found 420.77016.

1-(6-((4-(1-(*tert*-Butyl)-5-(4-(piperidin-1-yl)phenyl)-1,2,4-triazin-1-ium-3-yl)benzyl)amino)-6-oxohexyl)-3,3-dimethyl-2-((E)-3-((E)-1,3,3-trimethyl-5-sulfonatoindolin-2-ylidene)prop-1-en-1-yl)-3H-indol-1-ium-5-sulfonate, potassium salt (Trz⁺SulfoCy3)

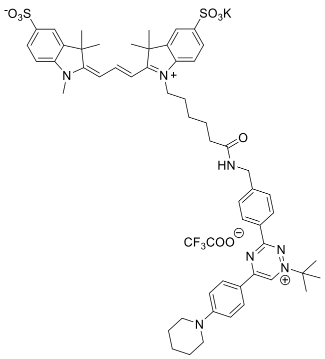

Trz$^+$1 (1.0 equiv., 8.2 mg, 0.013 mmol) and commercial Sulfo-Cyanine3 NHS ester (0.9 equiv., 8.8 mg, 0.012 mmol), and HATU (1.5 equiv., 7.4 mg, 0.020 mmol) were dissolved in dry DMF (770 µL) and the solution was cooled to 0 °C. DIPEA (10.0 equiv., 23.0 µL, 0.130 mmol) was then added dropwise. After 4 h of stirring at rt, the reaction was diluted with (1:1) MeCN/H_2O (4.23 mL) mixture, neutralized with TFA (10.0 equiv., 10.0 µL, 0.130 mmol) and the crude product was purified by preparative HPLC (Arion Plus (21.2 × 250 mm, 5 µm) column). MeCN/H_2O (+ 0.1% TFA) gradient (flow rate 15 mL/min) 10:90 → 85:15 for 25 min. Re-purified by column chromatography on silica gel in HILIC mode (puri*Flash*® from *InterChim,* SiO_2 (25 g) column). H_2O/Me_2CO (+ 0.1% TFA) gradient 0:100 → 25:75 and flow rate 25 mL/min for 20 min. Product detection at λ = 552 and 285 nm, Me_2CO removed under reduced pressure, lyophilization.

Yield: 6.5 mg (0.006 mmol, 43%) of a dark violet solid.

^{1}H NMR (401 MHz, DMSO-d_6): δ 9.70 (s, 1H), 8.47 (dd, *J* = 7.8, 4.4 Hz, 3H), 8.41 (d, *J* = 8.3 Hz, 2H), 8.33 (t, *J* = 13.4 Hz, 1H), 7.78 (dd, *J* = 9.1, 1.6 Hz, 2H), 7.67 – 7.61 (m, 2H), 7.50 (d, *J* = 8.2 Hz, 2H), 7.36 (dd, *J* = 13.2, 8.3 Hz, 2H), 7.24 (s, 1H), 7.16 (d, *J* = 9.4 Hz, 2H), 7.11 (s, 1H), 6.98 (s, 1H), 6.48 (dd, *J* = 13.5, 10.8 Hz, 2H), 4.38 (d, *J* = 5.9 Hz, 2H), 4.12 (d, *J* = 7.6 Hz, 2H), 3.65 – 3.57 (m, 7H), 2.19 (t, *J* = 7.3 Hz, 2H), 1.83 (s, 9H),1.78 – 7.60 (m, 23H).

^{13}C NMR (126 MHz, DMSO-d_6): δ 174.8, 173.9, 172.2, 164.1, 162.6, 154.9, 149.6, 145.8, 145.7, 145.6, 142.4, 141.8, 140.0, 139.9, 136.4 (from 2D spectra), 132.0 (2×CH), 131.1, 128.4 (2×CH), 127.8 (2×CH), 126.2, 126.0, 119.9, 119.7, 118.3, 113.2 (2×CH), 110.5, 103.3, 102.7, 75.1, 48.8, 47.5, 43.7, 41.7, 35.0, 31.8 (1×CH_3, from 2D spectra), 28.0 (3×CH_3), 27.4 (2×CH_3), 27.2 (2×CH_3), 26.6, 25.6, 25.2, 24.8, 23.9.

HRMS (ESI): m/z calcd. for $C_{56}H_{67}O_7N_7S_2$ $[M+H]^{2+}$ 500.72662, found 500.72668.

5-((4-(1-(*tert*-Butyl)-5-(4-(piperidin-1-yl)phenyl)-1,2,4-triazin-1-ium-3-yl)benzyl)carbamoyl)-2-(6-(dimethylamino)-3-(dimethyliminio)-3*H*-xanthen-9-yl)benzoate 2,2,2-trifluoroacetate (Trz$^+$TAMRA)

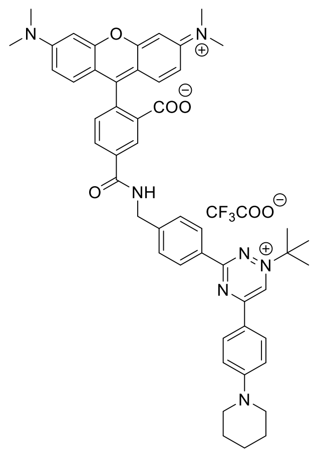

Trz⁺1 (1.0 equiv., 10.1 mg, 0.016 mmol) and commercial TAMRA NHS ester, 5-isomer (1.2 equiv., 10.1 mg, 0.019 mmol) were dissolved in dry MeCN (750 µL) and the solution was cooled to 0 °C. DIPEA (7.0 equiv., 20.0 µL, 0.112 mmol) was added dropwise. After 2 h of stirring at rt, more DIPEA (3.5 equiv., 10.0 µL, 0.056 mmol) and TAMRA NHS ester (0.5 equiv., 4.2 mg, 0.011 mmol) were then added for an additional consumption of the remaining **Trz⁺1** and stirring continued for 2 h. The reaction was diluted with (2:1) MeCN/H_2O (4.25 mL) mixture, neutralized with TFA (10.5 equiv., 13.0 µL, 0.168 mmol) and the crude product was purified by preparative HPLC (Arion Plus (21.2 × 250 mm, 5 µm) column). MeCN/H_2O (+ 0.1% TFA) gradient (flow rate 15 mL/min) 25:75 → 80:20 for 30 min. Product detection at λ = 555 and 254 nm, lyophilization.

Yield: 9.0 mg (0.010 mmol, 37%) of a dark purple solid.

^{1}H NMR (400 MHz, DMSO-d_6): δ 9.77 (s, 1H), 9.69 (t, J = 6.0 Hz, 1H), 8.77 (d, J = 1.8 Hz, 1H), 8.55 – 8.50 (m, 2H), 8.50 – 8.46 (m, 2H), 8.40 (dd, J = 8.1, 1.8 Hz, 1H), 7.70 – 7.66 (m, 2H), 7.62 (d, J = 7.8 Hz, 1H), 7.20 – 7.15 (m, 2H), 7.09 – 6.91 (m, 6H), 4.70 (d, J = 5.8 Hz, 2H), 3.62 (t, J = 5.5 Hz, 4H), 3.26 (s, 12H), 1.85 (s, 9H), 1.71 – 7.65 (m, 2H), 1.65 – 7.58 (m, 4H).

^{19}F NMR (376 MHz, DMSO-d_6): δ -73.76.

^{13}C NMR (126 MHz, DMSO-d_6): δ 165.9, 164.9, 164.2, 162.6, 155.0, 145.2, 136.2, 135.7, 132.0 (2×CH), 131.5 (from 2D spectra), 131.3, 130.43, 130.41 (2×CH), 129.8 (from 2D spectra), 128.5 (2×CH), 128.2 (2×CH), 120.5, 118.3, 118.1, 115.7, 115.0 (2×CH, from 2D spectra), 113.4, 113.3 (2×CH), 96.3 (2×CH), 75.1, 47.5, 42.8, 25.2, 23.9. Signal of two quart. carbon was not found.

HRMS (ESI): m/z calcd. for $C_{50}H_{52}O_4N_7$ [M^+] 814.40753, found 814.40830.

1-(*tert*-Butyl)-3-(4-((3,7-di(azetidin-1-yl)-5,5-dimethyl-3'-oxo-3'*H*,5*H*-spiro[dibenzo[b,e]siline-10,1'-isobenzofuran]-6'-carboxamido)methyl)phenyl)-5-(4-(piperidin-1-yl)phenyl)-1,2,4-triazin-1-ium 2,2,2-trifluoroacetate (Trz⁺SiRho)

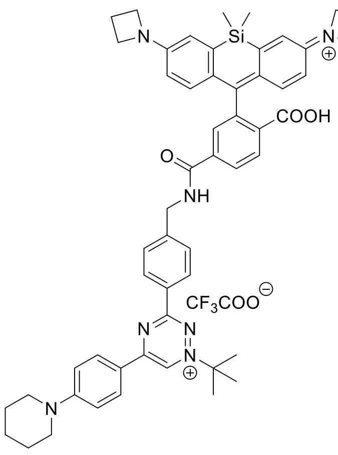

Commercial Janelia Fluor® 646 free acid (1.0 equiv., 5.0 mg, 0.008 mmol) and DSC (2.2 equiv., 4.6 mg, 0.018 mmol) were dissolved in dry DMF (500 µL). DIPEA (6.0 equiv., 7.0 µL, 0.049 mmol) was then added dropwise, followed by DMAP (10 mol%, 100.1 µg, 819 µmol). After 1 h of stirring at rt and quantitative NHS ester formation (LC-MS analysis), **Trz⁺1** (2.0 equiv., 10.3 mg, 0.016 mmol) was added in one portion, and stirring continued for 1 h. The reaction was diluted with (1:1) MeCN/H_2O (4.50 mL) mixture, acidified with TFA (10.0 equiv., 6.0 µL, 0.082 mmol) and the crude product was purified by preparative HPLC (Arion Plus (21.2 × 250 mm, 5 µm) column). MeCN/H_2O (+ 0.1% TFA) gradient (flow rate 15 mL/min) 35:65 → 95:5 for 30 min. Product detection at λ = 665 and 530 nm, lyophilization.

Yield: 5.0 mg (0.005 mmol, 61%) of a dark blue solid.

^{1}H NMR (401 MHz, DMSO-*d*$_6$): δ 9.73 (s, 1H), 9.50 – 9.44 (m, 1H), 8.51 – 8.46 (m, 2H), 8.45 – 8.39 (m, 2H), 8.17 (dd, *J* = 8.1, 1.4 Hz, 1H), 8.08 (d, *J* = 8.0 Hz, 1H), 7.74 (t, *J* = 1.1 Hz, 1H), 7.58 (d, *J* = 8.4 Hz, 2H), 7.20 – 7.12 (m, 2H), 6.74 (br s, 2H), 6.65 (d, *J* = 8.8 Hz, 2H), 6.34 (dd, *J* = 8.8, 2.6 Hz, 2H), 4.58 (d, *J* = 5.7 Hz, 2H), 3.84 (br s, 8H), 3.65 – 3.58 (m, 4H), 2.35 – 2.24 (m, 4H), 2.08 (s, 3H), 1.83 (s, 9H), 1.73 – 1.57 (m, 6H), 0.60 (s, 3H), 0.50 (s, 3H).

^{19}F NMR (377 MHz, DMSO-*d*$_6$): δ -74.59.

^{13}C NMR (101 MHz, DMSO-*d*$_6$): δ 164.9, 164.2, 162.6, 158.26, 157.97, 155.0, 150.9, 145.0, 136.0 (from 2D spectra), 132.0 (2×CH), 131.3, 129.2 (2×CH, from 2D spectra), 128.9 (from 2D spectra), 128.5 (2×CH), 128.2, 128.1, 123.4 (from 2D spectra), 118.3, 117.0, 116.0 (from 2D spectra), 114.7, 113.3 (2×CH), 112.7, 75.1, 51.9, 47.5, 42.7, 30.7, 28.0, 25.2, 23.9, 16.3, -0.1 (1×Si-CH_3), -1.3 (1×Si-CH_3). Signal of four quart. carbon was not found.

HRMS (ESI): m/z calcd. for $C_{54}H_{58}O_3N_7Si$ [M^+] 880.43649, found 880.43715.

Synthesis of **HaloTrz⁺Coum** ligand

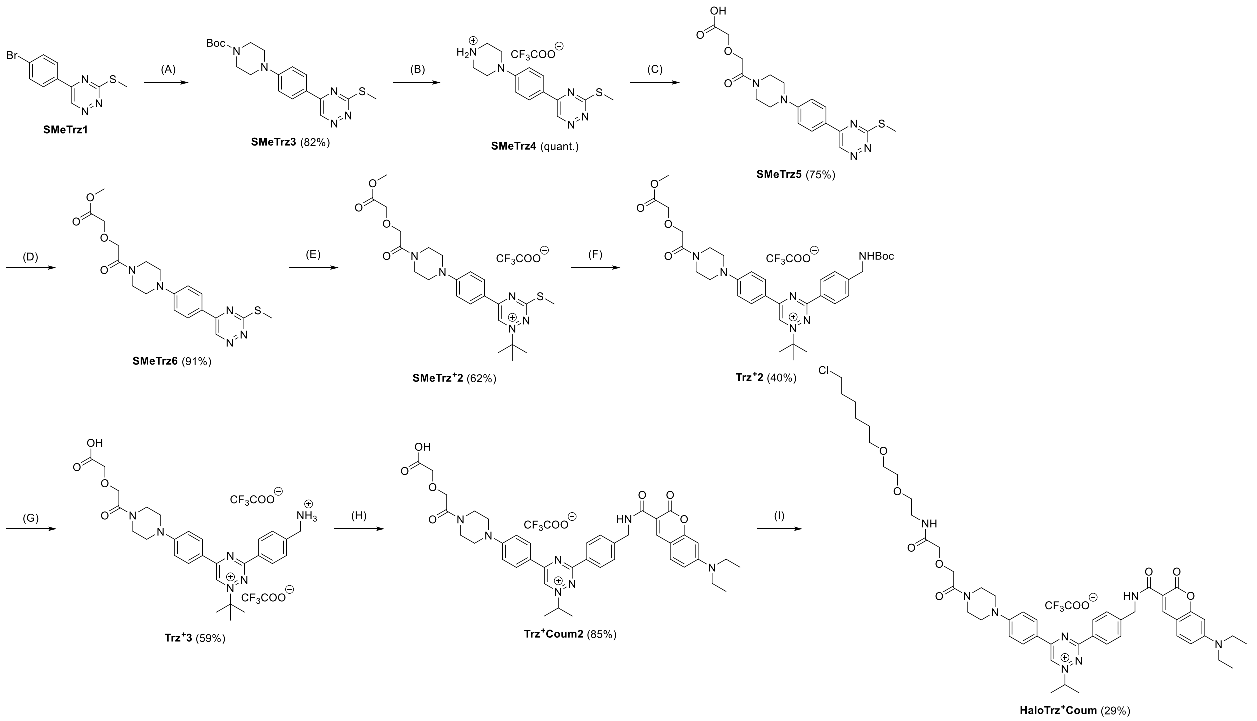

Scheme S3: Schematic overview of **HaloTrz⁺Coum** ligand synthetic pathway

tert-Butyl 4-(4-(3-(methylthio)-1,2,4-triazin-5-yl)phenyl)piperazine-1-carboxylate (SMeTrz3)

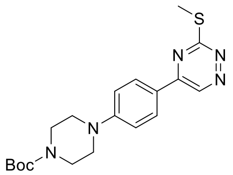

(A) Representative procedure B was applied. **SMeTrz1**[1] (1.000 mmol) was used. Purification: EA/DCM gradient 10:90 → 20:80.

Yield: 319.0 mg (0.823 mmol, 82%) of a bright yellow amorphous solid contaminated with small impurities.

^{1}H NMR (401 MHz, $CDCl_3$): δ 9.25 (s, 1H), 8.11 – 8.05 (m, 2H), 6.97 – 6.93 (m, 2H), 3.65 – 3.55 (m, 4H), 3.43 – 3.29 (m, 4H), 2.69 (s, 3H), 1.49 (s, 9H).

^{13}C NMR (101 MHz, $CDCl_3$): δ 173.2, 154.7, 154.0, 153.9, 141.4, 129.3 (2×CH), 122.6, 114.8 (2×CH), 80.3, 47.4, 28.5 (3×CH_3), 13.9 (1×CH_3).

HRMS (ESI): m/z calcd. for $C_{19}H_{26}O_2N_5S$ $[M+H]^+$ 388.18017, found 388.17987.

4-(4-(3-(Methylthio)-1,2,4-triazin-5-yl)phenyl)piperazin-1-ium (SMeTrz4)

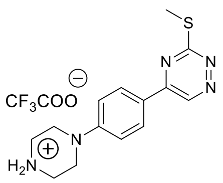

(B) To a solution of **SMeTrz3** (500.0 mg, 1.290 mmol) in DCM (50.0 mL), an excess of TFA (9.5 mL) was added dropwise at rt. The mixture was stirred at rt for 1 h. It was then concentrated under reduced pressure yielding a sufficiently pure product.

Yield: 517.0 mg (1.290 mmol, quant.) of a bright red solid.

^{1}H NMR (401 MHz, MeOH-d_4): δ 9.42 (s, 1H), 8.26 – 8.20 (m, 2H), 7.19 – 7.13 (m, 2H), 3.63 (dd, *J* = 6.2, 4.4 Hz, 4H), 3.35 (d, *J* = 2.7 Hz, 4H, overlapped with a signal of residual MeOH (3.34 ppm) remaining in the sample), 2.68 (s, 3H).

HRMS (ESI): m/z calcd. for $C_{14}H_{18}N_5S$ $[M+H]^+$ 288.12774, found 288.12769.

2-(2-(4-(4-(3-(Methylthio)-1,2,4-triazin-5-yl)phenyl)piperazin-1-yl)-2-oxoethoxy)acetic acid (SMeTrz5)

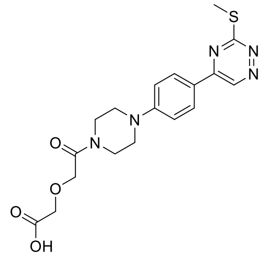

(C) SMeTrz4 (1.0 equiv., 517.0 mg, 1.290 mmol), and diglycolic anhydride (1.5 equiv., 166.1 mg, 1.936 mmol) were dissolved in dry DMF (60.0 mL) and the solution was cooled to 0 °C. DIPEA (5.0 equiv., 1.12 mL, 6.455 mmol) was added dropwise. After 1 h of stirring at rt, the reaction was diluted by (1:1) MeCN/H_2O (20.0 mL), filtered through a fritted adapter, washed with (1:1) MeCN/H_2O (2 × 20.0 mL and the crude product was dried under high vacuum. The filtrate was purified by RP flash (Combi*Flash*® Rf+) chromatography on pre-packed (RediSep Gold C18Aq) RP (50 g) column (MeCN/H_2O gradient 20:80 → 95:5), using a dry loading technique (flow rate 35 mL/min) for 30 min. Product detection at λ =254 and 399 nm, lyophilization.

Yield: 464.0 mg (1.150 mmol, 75%) of a dark orange solid.

^{1}H NMR (401 MHz, DMSO-d_6): δ 12.80 (s, 1H), 9.66 (s, 1H), 8.20 (d, *J* = 9.2 Hz, 2H), 7.10 (d, *J* = 9.2 Hz, 2H), 4.31 (s, 2H), 4.11 (s, 2H), 3.64 – 3.56 (m, 4H), 3.49 – 3.39 (m, 4H), 2.64 (s, 3H).

^{13}C NMR (101 MHz, DMSO-d_6): δ 171.8, 171.3, 167.3, 153.5, 153.4, 141.9, 129.3 (2×CH), 120.8, 114.1 (2×CH), 69.0, 67.6, 46.4, 46.2, 43.5, 40.6, 13.1 (1×CH_3).

HRMS (ESI): m/z calcd. for $C_{18}H_{20}O_4N_5S$ $[M-H]^-$ 402.12415, found 402.12400.

Methyl 2-(2-(4-(4-(3-(methylthio)-1,2,4-triazin-5-yl)phenyl)piperazin-1-yl)-2-oxoethoxy)acetate (SMeTrz6)

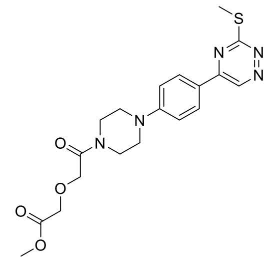

(D) SMeTrz5 (1.0 equiv., 398.0 mg, 0.984 mmol) was dissolved in dry THF (7.0 mL) under argon. The solution was cooled to 0 °C, and TEA (3.0 equiv., 525.4 µL, 2.951 mmol), followed by HATU (1.5 equiv., 561.1 mg, 1.476 mmol) was then added dropwise and in one portion, respectively. After 15 min of stirring at 0 °C for an acid activation, dry MeOH (2.0 mL) was added dropwise. The reaction was then stirred under argon at rt for 16 h. After concentration under reduced pressure, the mixture was diluted with DCM (100 mL/mmol) and washed with H_2O (100 mL/mmol). The organic phase was dried over Na_2SO_4, filtered, and concentrated under reduced pressure. The crude product was purified by flash (Combi*Flash*® Rf+) column chromatography on silica (100 g) column (DCM/MeOH gradient 96:4 → 90:10), (flow rate 60 mL/min) for 15 min. Product detection at λ =254 and 399 nm.

Yield: 373.0 mg (0.893 mmol, 91%) of a dark orange solid. This compound **SMeTrz6** contained 1,1,3,3-tetramethylurea which eluted with the product during the column chromatography.

^{1}H NMR (401 MHz, $CDCl_3$): δ 9.26 (s, 1H), 8.13 – 8.06 (m, 2H), 6.99 – 6.94 (m, 2H), 4.33 (s, 2H), 4.23 (s, 2H), 3.82 – 3.75 (m, 7H), 3.46 – 3.37 (m, 4H), 2.70 (s, 3H).

^{13}C NMR (101 MHz, $CDCl_3$): δ 173.3, 170.4, 167.0, 153.9, 153.7, 141.4, 129.3 (2×CH), 123.0, 115.0 (2×CH), 70.6, 68.1, 52.1 (1×COO$\underline{C}H_3$), 47.8, 47.3, 44.7, 41.5, 13.9 (1×S$\underline{C}H_3$).

HRMS (ESI): m/z calcd. for $C_{19}H_{24}O_4N_5S$ $[M+H]^+$ 418.15425, found 418.15435.

1-(*tert*-Butyl)-5-(4-(4-(2-(2-methoxy-2-oxoethoxy)acetyl)piperazin-1-yl)phenyl)-3-(methylthio)-1,2,4-triazin-1-ium trifluoromethanesulfonate (SMeTrz$^+$2)

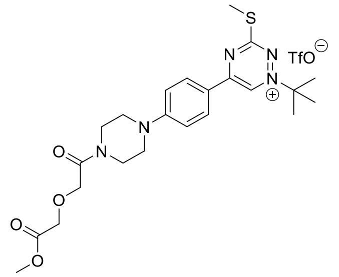

(E) Representative procedure B was applied. **SMeTrz6** (0.958 mmol, 400.0 mg) was used.

Purification: (RediSep Gold C18Aq 150 g column). MeCN/H_2O gradient (flow rate 60 mL/min) 10:90 → 80:20 for 20 min. Product detection at λ = 398 and 07 nm.

Yield: 369.0 mg (0.591 mmol, 62%) of a dark red amorphous solid.

^{1}H NMR (401 MHz, CD_3CN): δ 9.15 (s, 1H), 8.29 – 8.23 (m, 2H), 7.09 – 7.02 (m, 2H), 4.28 (s, 2H), 4.18 (s, 2H), 3.71 – 3.59 (m, 11H), 2.70 (s, 3H), 1.79 (s, 9H).

^{19}F NMR (376 MHz, CD_3CN): δ -75.74.

^{13}C NMR (101 MHz, CD_3CN): δ 178.3, 171.4, 168.2, 162.6, 156.5, 133.9, 132.7 (2×CH), 119.5, 114.6 (2×CH), 77.2, 70.3, 68.6, 52.2 (1×COO<u>C</u>H3), 47.0, 46.7, 44.4, 41.6, 28.5 5 (3×C<u>C</u>H3)., 14.5 (1×S<u>C</u>H3).

HRMS (ESI): m/z calcd. for $C_{23}H_{32}O_4N_5S$ $[M]^+$ 474.21695, found 474.21674.

3-(4-(((*tert*-Butoxycarbonyl)amino)methyl)phenyl)-1-(*tert*-butyl)-5-(4-(4-(2-(2-methoxy-2-oxoethoxy)acetyl)piperazin-1-yl)phenyl)-1,2,4-triazin-1-ium 2,2,2-trifluoroacetate (Trz⁺2)

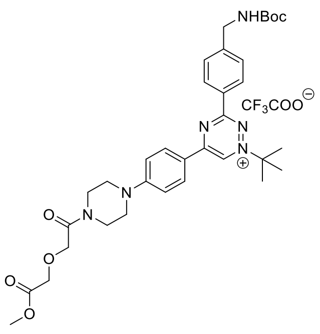

(F) Representative procedure C was applied. **SMeTrz⁺2** (0.592 mmol, 369.0 mg) was used.

Purification: (RediSep Gold C18Aq 150 g column). MeCN/H_2O (+ 0.1% TFA) gradient (flow rate 60 mL/min) 15:85 → 80:20 for 25 min. Product detection at λ = 494 and 411 nm, lyophilization.

Yield: 176.0 mg (0.236 mmol, 40%) of a dark red amorphous solid.

^{1}H NMR (401 MHz, CD_3CN): δ 9.42 (s, 1H), 8.53 – 8.48 (m, 2H), 8.46 – 8.41 (m, 2H), 7.54 (d, *J* = 8.1 Hz, 2H), 7.13 – 7.07 (m, 2H), 4.35 (d, *J* = 6.2 Hz, 2H), 4.29 (s, 2H), 4.19 (s, 2H), 3.73 – 3.62 (m, 11H), 1.89 (s, 9H), 1.43 (s, 9H).

^{19}F NMR (376 MHz, CD_3CN): δ -75.74.

^{13}C NMR (101 MHz, CD_3CN): δ 171.4, 168.3, 166.9, 164.4, 156.5, 147.7, 135.6, 132.6 (2×CH), 132.2, 129.9 (2×CH), 128.8 (2×CH), 120.2, 114.6 (2×CH), 77.3, 70.3, 68.6, 52.3 (1×COO<u>C</u>H3), 47.0, 46.8, 44.5, 41.6, 28.7 (3×COOC<u>C</u>H3), 28.6 (3×C<u>C</u>H3). Signal of one quat. carbon was not found.

HRMS (ESI): m/z calcd. for $C_{34}H_{45}O_6N_6$ $[M]^+$ 633.33951, found 633.33892.

3-(4-(Ammoniomethyl)phenyl)-1-(*tert*-butyl)-5-(4-(4-(2-(carboxymethoxy)acetyl)piperazin-1-yl)phenyl)-1,2,4-triazin-1-ium 2,2,2-trifluoroacetate (Trz⁺3)

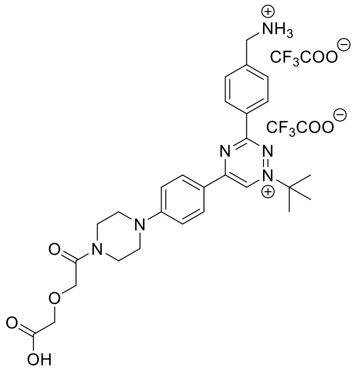

(G) To a solution of **Trz⁺2** (74.9 mg, 0.096 mmol) in (2:2:1) MeOH/THF/H_2O (6.0 mL) cooled to 0°C, 1.0M aq. LiOH.H_2O (4.0 equiv., 764 µL, 0.764 mmol) was added dropwise at 0°C. The mixture was stirred at 0°C for 1 h (LC-MS analysis). It was then neutralized by 1.0M TFA (4.0 equiv., 764.0 µL, 0.764 mmol) and lyophilized. The intermediate was then dissolved in dry MeCN (4.0 mL) and TFA (70.0 equiv., 513.0 µL, 6.697 mmol) was added dropwise at 0°C. The mixture was stirred at rt for 24 h and concentrated under reduced pressure. The crude product was purified by RP flash chromatography (RediSep Gold C18Aq 50 g column). MeCN/H_2O (+ 0.1% TFA) gradient (flow rate 35 mL/min) 5:95 → 55:45 for 20 min. Product detection at λ = 271 and 493 nm, lyophilization.

Yield: 42.0 mg (0.057 mmol, 59%) of a dark red amorphous solid.

^{1}H NMR (401 MHz, DMSO-*d₆*): δ 9.85 (s, 1H), 8.59 (d, *J* = 9.1 Hz, 2H), 8.57 – 8.47 (m, 5H), 7.80 – 7.73 (m, 2H), 7.19 (d, *J* = 9.1 Hz, 2H), 4.33 (s, 2H), 4.22 (q, *J* = 5.8 Hz, 2H), 4.13 (s, 2H), 3.73 – 3.59 (m, 8H), 1.87 (s, 9H).

^{19}F NMR (376 MHz, DMSO-d_6): δ -73.85.

^{13}C NMR (101 MHz, CD_3CN): δ 171.3, 167.4, 164.0, 163.2, 158.2, 157.9, 154.9, 139.5, 136.6, 132.5, 132.0, 129.6, 128.6, 119.4, 118.3, 115.4, 113.4, 75.4, 69.1, 67.6, 45.8, 45.5, 43.3, 41.8, 40.6, 28.0. Signal of one quart. carbon was not found.

HRMS (ESI): m/z calcd. for $C_{28}H_{35}O_4N_6$ $[M]^+$ 519.27143, found 519.27166.

1-(*tert*-Butyl)-5-(4-(4-(2-(carboxymethoxy)acetyl)piperazin-1-yl)phenyl)-3-(4-((7-(diethylamino)-4-methyl-2-oxo-2*H*-chromene-3-carboxamido)methyl)phenyl)-1,2,4-triazin-1-ium 2,2,2-trifluoroacetate (Trz⁺Coum2)

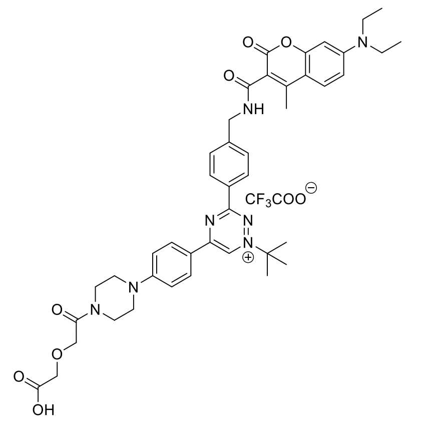

(H) Trz⁺3 (18.4 mg, 0.025 mmol) and commercial 7-(diethylamino)coumarin-3-carboxylic acid NHS ester (1.2 equiv. 10.9 mg, 0.031 mmol) were dissolved in dry MeCN (850 µL) and the solution was cooled to 0 °C. DIPEA (3.0 equiv., 13.0 µL, 0.076 mmol) was added dropwise. After 2 h of stirring at rt, the reaction was diluted with (2:1) MeCN/H_2O (up to 5.0 mL) mixture, neutralized with TFA (3.0 equiv., 5.2µL, 0.076 mmol) and the crude product was purified by preparative HPLC (Arion Plus (21.2 × 250 mm, 5 µm) column) in MeCN/H_2O (+ 0.1% TFA) gradient (flow rate 16 mL/min) 25:75 → 80:20 for 25 min. Product detection at λ = 490 and 424 nm, lyophilization.

Yield: 19.0 mg (0.022 mmol, 85%) of a dark red amorphous solid.

^{1}H NMR (401 MHz, CD_3CN): δ 9.37 (s, 1H), 9.24 – 9.17 (m, 1H), 8.65 (d, *J* = 0.7 Hz, 1H), 8.52 – 8.46 (m, 2H), 8.44 – 8.37 (m, 2H), 7.62 – 7.57 (m, 2H), 7.53 (d, *J* = 9.0 Hz, 1H), 7.10 – 7.03 (m, 2H), 6.77 (dd, *J* = 9.0, 2.5 Hz, 1H), 6.56 (dd, *J* = 2.5, 0.7 Hz, 1H), 4.69 (d, *J* = 6.1 Hz, 2H), 4.47 (s, 2H), 4.19 (s, 2H), 3.79 – 3.74 (m, 2H), 3.65 (dd, *J* = 6.8, 3.8 Hz, 4H), 3.56 – 3.52 (m, 2H), 3.48 (q, *J* = 7.2 Hz, 4H), 1.88 (s, 9H), 1.19 (t, *J* = 7.1 Hz, 6H).

^{19}F NMR (376 MHz, CD_3CN): δ -76.59.

^{13}C NMR (101 MHz, CD_3CN): δ 172.4, 171.6, 166.9, 164.4, 164.2, 163.5, 158.8, 156.3, 153.9, 148.9, 147.1, 135.5, 132.6, 132.3, 132.2 (2×CH), 130.0 (2×CH), 129.1 (2×CH), 120.3, 114.6 (2×CH), 111.2, 110.7, 109.0, 97.0, 77.4, 72.4, 71.9, 46.4, 45.6, 43.9, 43.7 (CONH$\underline{C}H_2$), 42.1, 28.7 (3×CH_3), 12.6 (2×CH_3).

HRMS (ESI): m/z calcd. for $C_{42}H_{48}O_7N_7$ [M^+] 762.36097, found 762.36074.

1-(*tert*-Butyl)-5-(4-(4-(18-chloro-5-oxo-3,9,12-trioxa-6-azaoctadecanoyl)piperazin-1-yl)phenyl)-3-(4-((7-(diethylamino)-2-oxo-2*H*-chromene-3-carboxamido)methyl)phenyl)-1,2,4-triazin-1-ium 2,2,2-trifluoroacetate (HaloTrz⁺Coum)

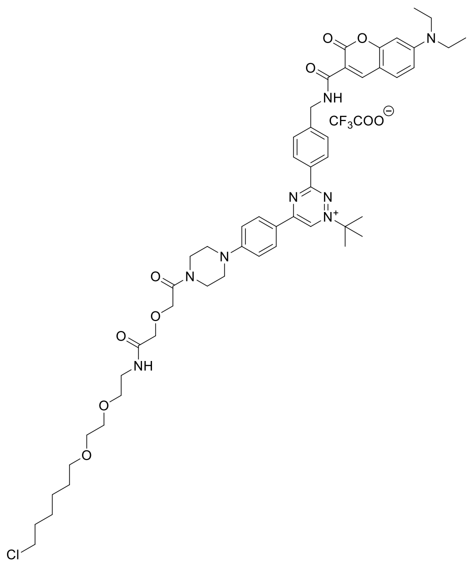

(I) Trz⁺Coum2 (5.5 mg, 0.006 mmol), HATU (1.2 equiv., 2.90 mg, 0.008 mmol), and **HaloTag ligand** 2-[2-(6-chlorohexoxy)ethoxy]ethanamine (1.2 equiv., 2.50 mg, 0.008 mmol) were dissolved in dry MeCN (275 µL) and the solution was cooled to 0 °C. DIPEA (7.0 equiv., 8.00 µL, 0.044 mmol) was added dropwise. After 2 h of stirring at rt, the reaction was diluted with (2:1) MeCN/H_2O (4.55 mL) mixture, neutralized with TFA (7.0 equiv., 3.00 µL, 0.044 mmol) and the crude product was purified by preparative HPLC (Arion Plus (21.2 × 250 mm, 5 µm) column in MeCN/H_2O (+ 0.1% TFA) gradient (flow rate 16 mL/min) 30:70 → 85:15 for 20 min. Product detection at λ = 500 and 424 nm, lyophilization.

Yield: 2.0 mg (0.002 mmol, 29%) of a dark red solid.

^{1}H NMR (401 MHz, CD_3CN): δ 9.37 (s, 1H), 9.21 (t, *J* = 6.0 Hz, 1H), 8.67 (s, 1H), 8.54 – 8.48 (m, 2H), 8.45 – 8.39 (m, 2H), 7.64 – 7.58 (m, 2H), 7.55 (d, *J* = 8.9 Hz, 1H), 7.47 (s, 1H), 7.11 – 7.06 (m, 2H), 6.79 (dd, *J* = 9.0, 2.4 Hz, 1H), 6.57 (dd, *J* = 2.5, 0.7 Hz, 1H), 4.71 (d, *J* = 6.1 Hz, 2H), 4.33 (s, 2H), 4.00 (s, 2H), 3.72 (br s, 2H), 3.68 – 3.61 (m, 4H), 3.59 – 3.54 (m, 6H), 3.53 – 3.46 (m, 9H), 3.44 – 3.34 (m, 5H), 1.88 (s, 9H), 1.79 – 1.69 (m, 2H), 1.58 – 1.49 (m, 2H), 1.46 – 1.30 (m, 5H), 1.19 (t, *J* = 7.0 Hz, 6H).

^{19}F NMR (376 MHz, CD_3CN): δ -76.10.

HRMS (ESI): m/z calcd. for $C_{52}H_{68}O_8N_8Cl$ [M^+] 967.48432, found 967.48566.

Synthesis of pyridazinedione-BCN rebridging reagent

Synthesis and characterization of the precursor pyridazinedione core was reported previously. [7]

***tert*-Butyl (15-(4,5-dibromo-2-ethyl-3,6-dioxo-3,6-dihydropyridazin-1(2*H*)-yl)-13-oxo-3,6,9-trioxa-12-azapentadecyl)carbamate (BrPD2)**

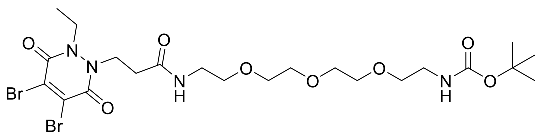

3-(4,5-dibromo-2-ethyl-3,6-dioxo-3,6-dihydropyridazin-1(2H)-yl)propanoic acid **(BrPD1)** (1.0 equiv., 50.0 mg, 0.135 mmol), HATU (1.5 equiv., 77.1 mg, 0.203 mmol) and commercial H2N-PEG3-NHBoc (1.1 equiv., 5.0 mg, 0.008 mmol) were dissolved in dry (2:1) MeCN/DMSO (1.50 mL) and the solution was cooled to 0 °C. DIPEA (1.5 equiv., 35.0 µL, 0.203 mmol) was added dropwise. After 30 min of stirring at rt, it was diluted with 1:1 MeCN/H_2O (3.50 mL) mixture, acidified with FA (2.0 equiv., 10.0 µL, 0.270 mmol), and the crude product was purified by preparative HPLC (Arion Plus (21.2 × 250 mm, 5 µm) column). MeCN/H_2O (+ 0.05% FA) gradient (flow rate 15 mL/min) 15:85 → 95:5 for 20 min. Product detection at λ = 266 and 344 nm, lyophilization.

Yield: 53.0 mg (0.082 mmol, 63%) of yellow oil.

^{1}H NMR (401 MHz, DMSO-d_6): δ 8.10 (t, *J* = 5.7 Hz, 1H), 6.74 (t, *J* = 5.8 Hz, 1H), 4.22 (t, *J* = 7.0 Hz, 2H), 4.08 (q, *J* = 7.1 Hz, 2H), 3.53 – 3.45 (m, 8H), 3.41 – 3.31 (m, 4H overlapped with signal of residual H_2O (3.33 ppm) remaining in the sample), 3.15 (q, *J* = 5.9 Hz, 2H), 3.05 (q, *J* = 6.1 Hz, 2H), 2.42 (t, *J* = 7.0 Hz, 2H), 1.36 (s, 9H), 1.13 (t, *J* = 7.0 Hz, 3H).

^{13}C NMR (101 MHz, DMSO-d_6): δ 169.2, 155.6, 153.1, 152.6, 135.5, 135.2, 77.6, 69.77, 69.73, 69.6, 69.5, 69.1, 69.0, 43.6, 42.0, 38.6, 33.1, 28.2 (3×CH_3), 12.6 (1×CH_3).

HRMS (ESI): m/z calcd. for $C_{22}H_{37}O_8N_4Br_2$ $[M+H]^+$ 643.09703, found 643.09727.

15-(4,5-Dibromo-2-ethyl-3,6-dioxo-3,6-dihydropyridazin-1(2*H*)-yl)-13-oxo-3,6,9-trioxa-12-azapentadecan-1-aminium 2,2,2-trifluoroacetate (BrPD3)

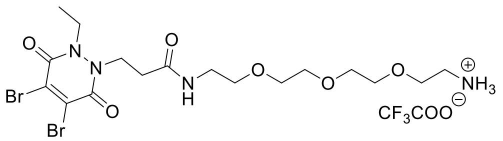

BrPD2 (1.0 equiv., 53.0 mg, 0.082 mmol) was dissolved in dry DCM (2.0 mL) and TFA (40.0 equiv., 250.0 µL, 3.265 mmol) was added dropwise at 0 °C. The mixture was stirred at rt for 1 h and concentrated under reduced pressure. The crude product was dissolved in (1:1) MeCN/H_2O (5.0 mL), and lyophilized. The crude **BrPD3** was obtained 57.0 mg (0.087 mmol, quant.) as yellow oil in satisfactory quality without additional purification.

^{1}H NMR (401 MHz, DMSO-d_6): δ 8.12 (t, *J* = 5.7 Hz, 1H), 7.93 – 7.72 (br s, 3H), 4.23 (t, *J* = 7.0 Hz, 2H), 4.08 (q, *J* = 7.0 Hz, 2H), 3.63 – 3.46 (m, 10H), 3.34 (t, *J* = 6.1 Hz, 2H), 3.15 (q, *J* = 6.0 Hz, 2H), 2.98 (q, *J* = 5.7 Hz, 2H), 2.42 (t, *J* = 6.9 Hz, 2H), 1.13 (t, *J* = 7.0 Hz, 3H).

^{19}F NMR (377 MHz, CD_3CN): δ -74.60.

^{13}C NMR (101 MHz, DMSO-d_6): δ 169.30, 153.1, 152.7, 135.5, 135.2, 69.69 (2×CH_2), 69.64, 69.5, 68.9, 66.7, 43.6, 42.0, 38.6, 38.5, 33.1, 12.6 (1×CH_3).

HRMS (ESI): m/z calcd. for $C_{17}H_{29}O_6N_4Br_2$ $[M^+]$ 543.04484, found 543.04472.

((1*R*,8*S*,9*s*)-Bicyclo[6.1.0]non-4-yn-9-yl)methyl (15-(4,5-dibromo-2-ethyl-3,6-dioxo-3,6-dihydropyridazin-1(2*H*)-yl)-13-oxo-3,6,9-trioxa-12-azapentadecyl)carbamate (BrPD4)

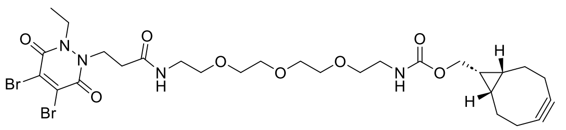

BrPD3 (1.0 equiv., 18.0 mg, 0.027 mmol), and commercial ***endo*-BCN-NHS** (1.3 equiv., 10.4 mg, 0.036 mmol) were dissolved in dry DMF (1.00 mL) and the solution was cooled to 0 °C. DIPEA (3.0 equiv., 14.0 µL, 0.082 mmol) was added dropwise. After 2 h of stirring at rt, it was diluted with 1:1 MeCN/H_2O (4.00 mL) mixture, acidified with FA (3.0 equiv., 4.0 µL, 0.082 mmol), and the crude product was purified by preparative HPLC (Arion Plus (21.2 × 250 mm, 5 µm) column). MeCN/H_2O (+ 0.05% FA) gradient (flow rate 15 mL/min) 20:80 → 95:5 for 20 min. Product detection at λ = 266 and 344 nm, lyophilization. **Note: This product was found unstable during storage in a fridge (at -8 °C) as solid for several weeks. The degradation process resulted in a complex mixture of various compounds.**

Yield: 8.0 mg (0.011 mmol, 41%) of yellow oil.

^{1}H NMR (401 MHz, DMSO-d_6): δ 8.10 (t, J = 5.7 Hz, 1H), 7.08 (t, J = 5.7 Hz, 1H), 4.22 (t, J = 7.0 Hz, 2H), 4.14 – 3.97 (m, 4H), 3.52 – 3.47 (br s, 7H), 3.44 – 3.28 (m, 6H), 3.20 – 3.05 (m, 4H), 2.42 (t, J = 7.0 Hz, 2H), 2.30 – 2.08 (m, 5H), 1.61 – 1.44 (m, 2H), 1.26 (p, J = 8.6 Hz, 1H), 1.14 (t, J = 7.0 Hz, 3H), 0.93 – 0.79 (m, 2H).

^{13}C NMR (101 MHz, DMSO-d_6): δ 169.2, 156.4, 153.1, 152.6, 135.5, 135.2, 99.0, 69.7, 69.6, 69.59, 69.53, 69.1, 68.9, 61.3, 43.5, 42.0, 40.0, 38.5, 33.1, 28.5, 20.8, 19.5, 17.6, 12.5 (1×CH_3).

HRMS (ESI): m/z calcd. for $C_{28}H_{41}O_8N_4Br_2$ [M^+] 719.12845, found 719.12857.

Synthesis of modified Dasatinib drug

2-(4-(6-((5-((2-chloro-6-methylphenyl)carbamoyl)thiazol-2-yl)amino)-2-methylpyrimidin-4-yl)piperazin-1-yl)ethyl (2,5-dioxopyrrolidin-1-yl) carbonate (Dasatinib-NHS)

Dasatinib.HCl (16.0 mg, 0.031 mmol) and *N,N'*-disuccinimidyl carbonate (2.0 equiv., 15.6 mg, 0.061 mmol) were suspended in dry DMF (1.5 mL) at rt. DIPEA (3.0 equiv., 16.0 µL, 0.092 mmol) was then added dropwise at rt and the stirring continued for 14 h. This crude reaction mixture was then used in the next step.

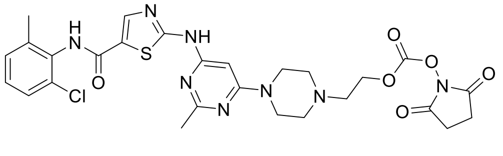

***N*-(6-(4-(1-((1*R*,8*S*,9*s*)-Bicyclo[6.1.0]non-4-yn-9-yl)-3,17-dioxo-2,7,10,13,18-pentaoxa-4,16-diazaicosan-20-yl)piperazin-1-yl)-2-methylpyrimidin-4-yl)-5-((2-chloro-6-methylphenyl)carbamoyl)thiazol-2-aminium formate (Dasatinib-BCN)**

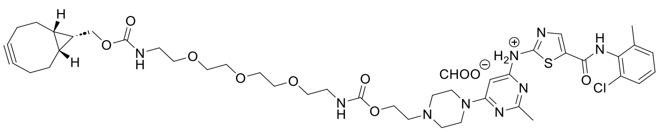

To the crude Dasatinib NHS ester, commercial ***endo*-BCN-PEG3-amine** (1.5 equiv., 16.9 mg, 0.046 mmol) was added in one portion followed by dropwise addition of DIPEA (3.0 equiv., 16.0 µL, 0.092 mmol). After 5 h of stirring at rt, it was diluted with 1:1 MeCN/H_2O (3.50 mL) mixture, acidified with FA (6.0 equiv., 7.0 µL, 0.183 mmol), and the crude product was purified by preparative HPLC (Arion Plus (21.2 × 250 mm, 5 µm) column). MeCN/H_2O (+ 0.05% FA) gradient (flow rate 15 mL/min) 5:95 → 85:15 for 20 min. Product detection at λ = 233 and 321 nm, lyophilization.

Yield:13.0 mg (0.014 mmol, 46%) of a white solid.

^{1}H NMR (401 MHz, MeOD): δ 8.15 (s, 1H), 7.35 (dd, *J* = 7.3, 2.3 Hz, 1H), 7.29 – 7.19 (m, 2H), 6.01 (s, 1H), 4.59 (s, 1H), 4.22 (t, *J* = 5.7 Hz, 2H), 4.12 (d, *J* = 8.1 Hz, 2H), 3.71 – 3.58 (m, 14H), 3.54 (q, *J* = 5.5 Hz, 4H), 3.33 – 3.26 (m,4H, overlapped with deuterated solvent), 2.70 (q, *J* = 5.8 Hz, 2H), 2.62 (t, *J* = 5.2 Hz, 4H), 2.47 (s, 3H), 2.32 (s, 3H), 2.26 – 2.09 (m, 6H), 1.65 – 1.49 (m, 2H), 1.35 (p, *J* = 8.6 Hz, 1H), 0.99 – 0.85 (m, 2H).

^{13}C NMR (101 MHz, MeOD): δ 167.4, 165.2, 164.4, 163.2, 158.5, 142.2, 140.3, 134.3, 134.2, 130.1, 129.5, 128.3, 126.8, 116.7 (from 2D spectra), 99.5 (2×CH), 83.8 (1×CH_3), 71.5 (3×CH_2), 71.2 (3×CH_2), 71.0, 70.9, 63.7, 63.1, 58.1, 54.0, 44.9, 41.7, 41.6, 30.1 (2×CH_2), 25.6, 21.9 (2×CH_2), 21.3 (1×CH_3), 18.9, 18.7. Signal of one quat. carbon was not found.

HRMS (ESI): m/z calcd. for $C_{42}H_{56}O_8N_9ClNaS$ [M+Na] 904.35533, found 904.35544.

(Geldanamycin-BCN)

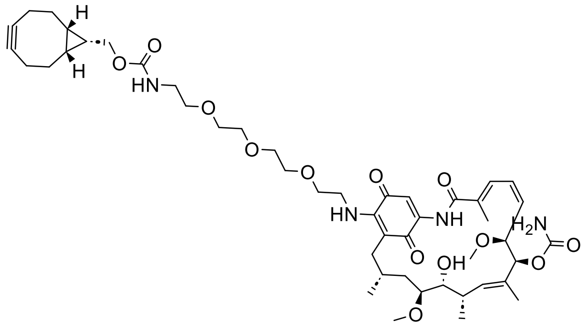

Geldanamycin (1.0 equiv., 15.0 mg, 0.027 mmol), and commercial ***endo*-BCN-PEG3-amine** (1.0 equiv., 9.9 mg, 0.027 mmol) were dissolved in dry $CHCl_3$ (800 µL) and the solution was cooled to 0 °C. DIPEA (10.0 equiv., 47.0 µL, 0.268 mmol) was added dropwise. After 64 h of stirring at rt, it was acidified with FA (10.0 equiv., 12.0 µL, 0.268 mmol) and concentrated under reduced pressure. The mixture was then re-dissolved in (4:1) MeCN/H_2O (5.0 mL), and the crude product was purified by preparative HPLC (Arion Plus (21.2 × 250 mm, 5 µm) column). MeCN/H_2O (+ 0.05% FA) gradient (flow rate 15 mL/min) 25:75 → 95:5 for 20 min. Product detection at λ = 233 and 334 nm, lyophilization.

Yield:14.0 mg (0.016 mmol, 58%) of a dark violet solid.

^{1}H NMR (401 MHz, $CDCl_3$): δ 9.17 (s, 1H), 7.27 (s, 1H), 6.95 (d, *J* = 11.5 Hz, 1H), 6.66 (s, 1H), 6.62 – 6.53 (m, 1H), 6.99 – 6.91 (m, 2H), 5.25 – 5.15 (m, 2H), 4.77 (br s, 1H), 4.36 – 4.26 (m, 2H), 4.15 (d, *J* = 8.1 Hz, 2H), 3.84 – 3.52 (m, 16H), 3.45 (dt, *J* = 8.8, 2.9 Hz, 1H), 3.40 – 3.33 (m, 5H), 3.27 (s, 3H), 2.79 – 2.63 (m, 2H), 2.38 (dd, *J* = 14.1, 10.6 Hz, 1H), 2.33 – 2.15 (m, 6H), 2.02 (d, *J* = 1.3 Hz, 3H), 1.81 – 1.69 (m, 6H), 1.64 – 1.52 (m, 3H), 1.36 (q, *J* = 8.3 Hz, 1H), 1.01 – 0.90 (m, 8H).

HRMS (ESI): m/z calcd. for $C_{47}H_{68}O_{13}N_4Na$ [M+Na] 919.46751, found 919.46802.

HPLC-MS analysis of Trz$^+$Fluorophores and the respective click products with *endo*-BCN-OH

10 mM solution of **Trz$^+$Fluorophore** (5 µL) in DMSO (100%) (Figure S1A1-S1I1) was combined in 90 µL of MeCN/H_2O (1:1) with 100 mM solution of ***endo*-BCN-OH** (5 µL) in DMSO (100%). 10 mM solution of **Trz$^+$OG** (5 µL) in DMSO (100%) was combined in 90 µL of MeCN (100%) with 100 mM solution of ***endo*-BCN-OH** (5 µL) in DMSO (100%) to ensure proper solubility (Figure S1C). The final concentration of each **Trz$^+$Fluorophore** was 0.5 mM, and ***endo*-BCN-OH** was used in 10-molar excess. The resulting mixture was incubated in a shaker at rt for 1 h and then analyzed by HPLC-MS on Luna C18 column (3 µm, 10 × 4.6 mm) (Figure S1).

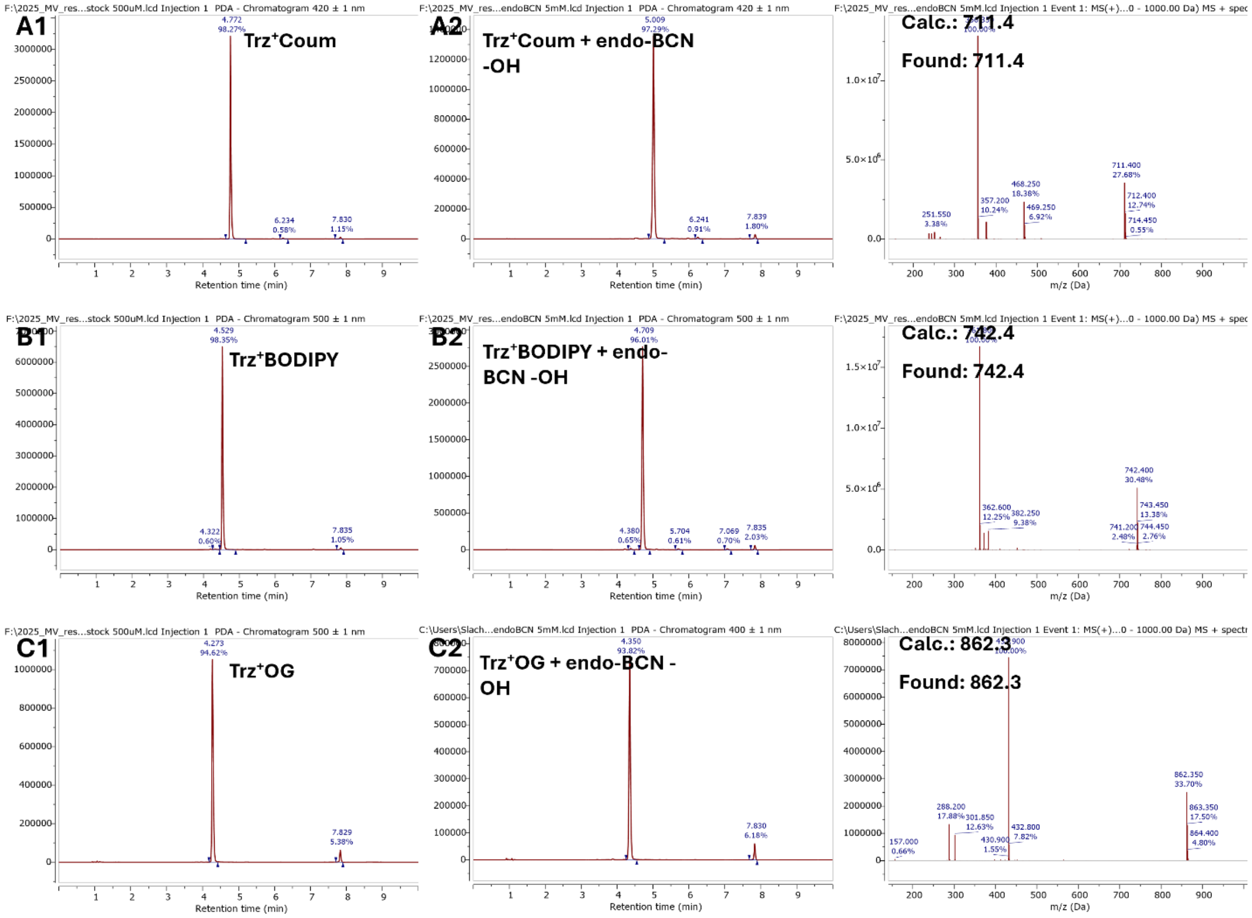

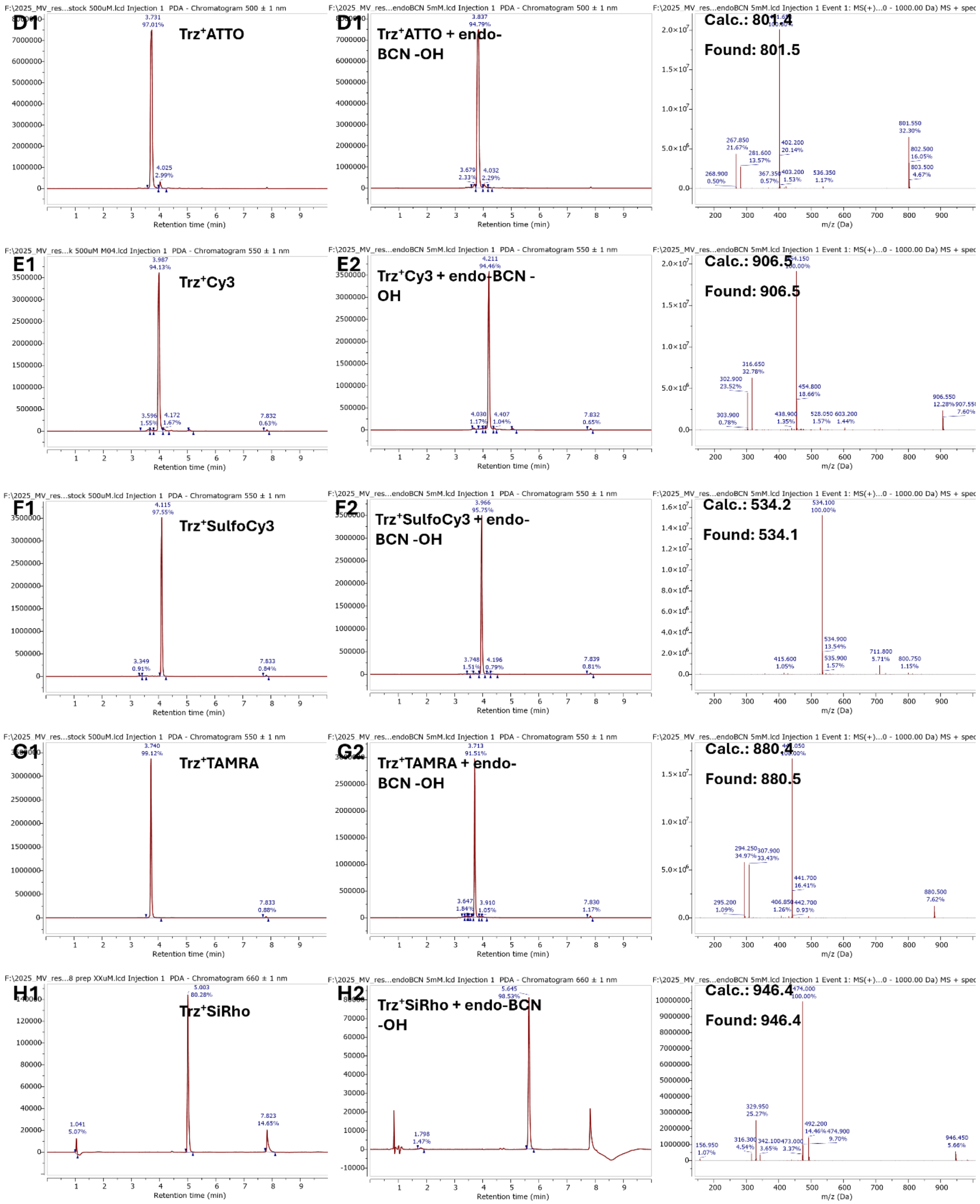
D1
Trz+ATTO
D1
Trz+ATTO + endo-BCN -OH
Calc.: 801.4
Found: 801.5
E1
Trz+Cy3
E2
Trz+Cy3 + endo-BCN -OH
Calc.: 906.5
Found: 906.5
F1
Trz+SulfoCy3
F2
Trz+SulfoCy3 + endo-BCN -OH
Calc.: 534.2
Found: 534.1
G1
Trz+TAMRA
G2
Trz+TAMRA + endo-BCN -OH
Calc.: 880.4
Found: 880.5
H1
Trz+SiRho
H2
Trz+SiRho + endo-BCN -OH
Calc.: 946.4
Found: 946.4
Retention time (min)
m/z (Da)

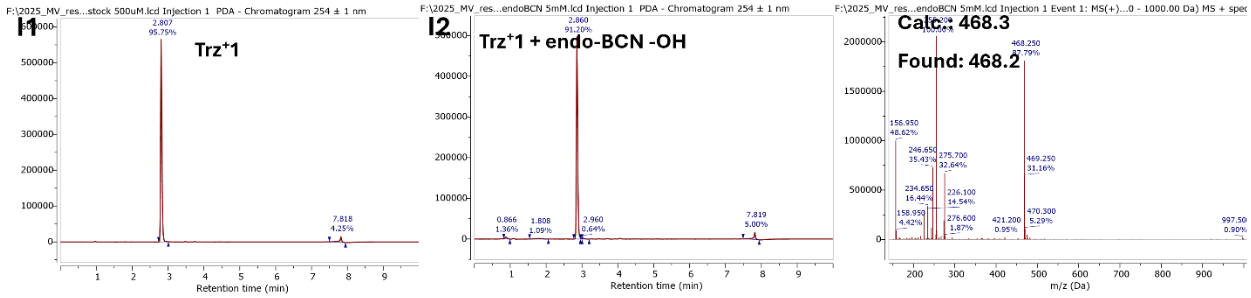

Figure S1: Exported HPLC-MS chromatogram of **Trz⁺Fluorophore** before (A) and after the reaction with ***endo*-BCN** (B) with the corresponding MS chromatogram of the click product on the right (λ = 254 / 420 / 500 / 550 / 660nm) after 1 h. Solvent system: solvent A: H_2O + 0.05% HCOOH; solvent B: MeCN + 0.05% HCOOH. Gradient: 5% B → 95% B (6.0 min), then 95% B (0.5 min), then 95% B → 5% B (0.5 min) and 5% B (3.0 min). The signal at 7.8 min is an impurity from the column.

Normalized absorption spectra

Absorbance measurement was performed as follows: 1 µL of the 10 mM stock solution in DMSO/H_2O (1:1) of each probe was pre-mixed in 200 µL of 100% DMSO or MeCN/H_2O (1:1) and diluted into 800 µL of the 100% PBS buffer (1×, pH = 7.4) or MeCN/H_2O (1:1) to the final 10 µM concentration. It was necessary to use **MeTzNH₂** at the final 50 µM concentration. The cuvette was immediately inserted into the spectrophotometer and the measurement was started. The absorbance of the corresponding click products formed in the reaction with ***endo*-BCN** was measured in the same way. Prior to the final dilution, the pre-mixed mixture was incubated with ***endo*-BCN** (1 µL from 100 mM fresh stock in MeCN/H_2O (1:1)) ca. 30 min to ensure complete conversion. The plots are depicted in Figure S2.

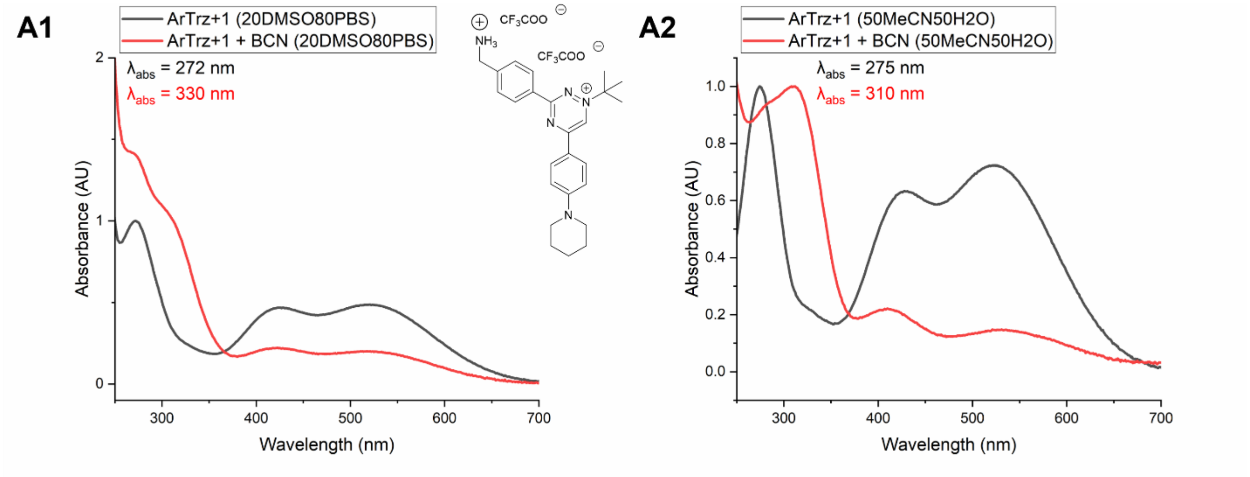

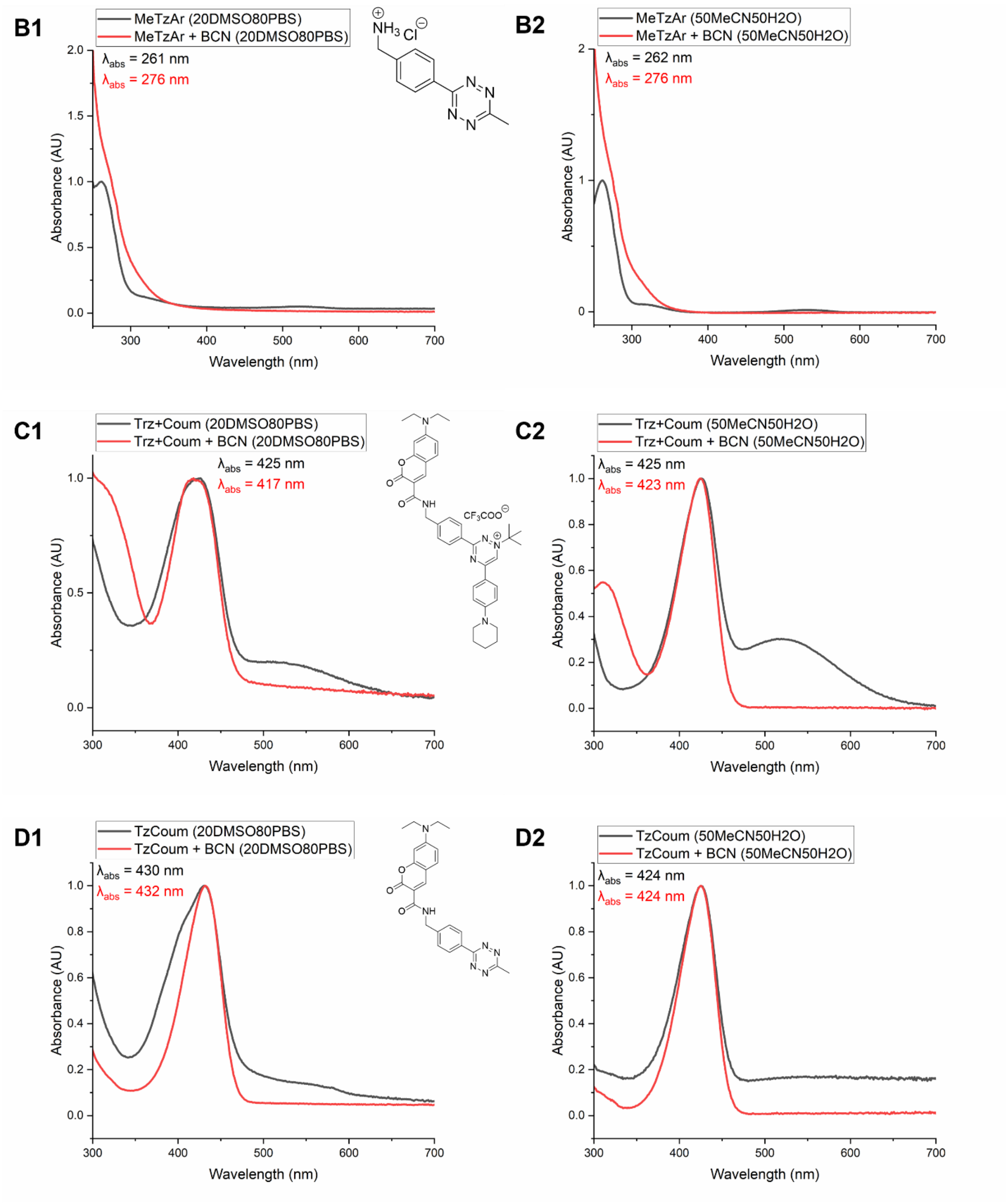
B1
MeTzAr (20DMSO80PBS)
MeTzAr + BCN (20DMSO80PBS)
λabs = 261 nm
λabs = 276 nm
Absorbance (AU)
Wavelength (nm)
B2
MeTzAr (50MeCN50H2O)
MeTzAr + BCN (50MeCN50H2O)
λabs = 262 nm
λabs = 276 nm
Absorbance (AU)
Wavelength (nm)
C1
Trz+Coum (20DMSO80PBS)
Trz+Coum + BCN (20DMSO80PBS)
λabs = 425 nm
λabs = 417 nm
Absorbance (AU)
Wavelength (nm)
C2
Trz+Coum (50MeCN50H2O)
Trz+Coum + BCN (50MeCN50H2O)
λabs = 425 nm
λabs = 423 nm
Absorbance (AU)
Wavelength (nm)
D1
TzCoum (20DMSO80PBS)
TzCoum + BCN (20DMSO80PBS)
λabs = 430 nm
λabs = 432 nm
Absorbance (AU)
Wavelength (nm)
D2
TzCoum (50MeCN50H2O)
TzCoum + BCN (50MeCN50H2O)
λabs = 424 nm
λabs = 424 nm
Absorbance (AU)
Wavelength (nm)

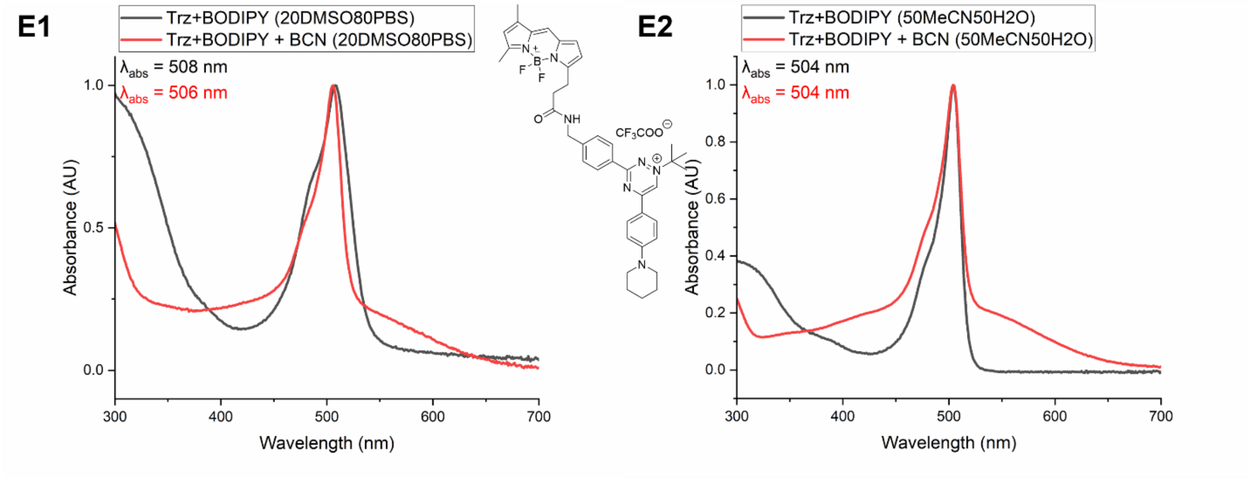
E1
Trz+BODIPY (20DMSO80PBS)
Trz+BODIPY + BCN (20DMSO80PBS)
λ_{abs} = 508 nm
λ_{abs} = 506 nm
Absorbance (AU)
Wavelength (nm)
E2
Trz+BODIPY (50MeCN50H2O)
Trz+BODIPY + BCN (50MeCN50H2O)
λ_{abs} = 504 nm
λ_{abs} = 504 nm
Absorbance (AU)
Wavelength (nm)
$CF_3COO^{\ominus}$

F1

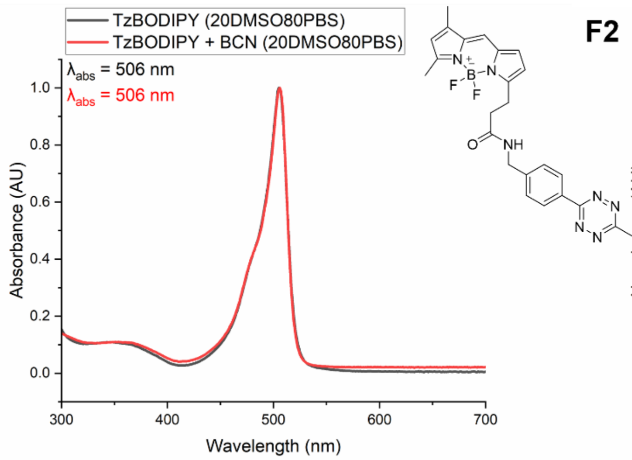

F2

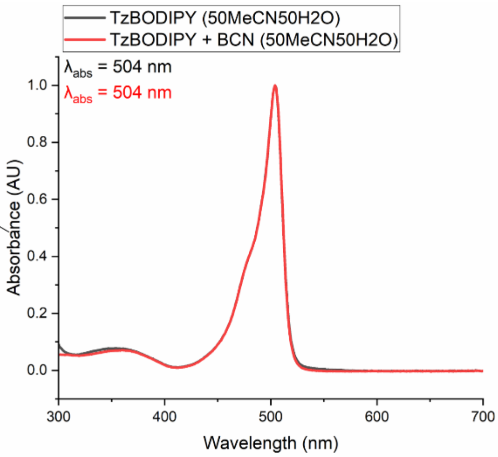

G1

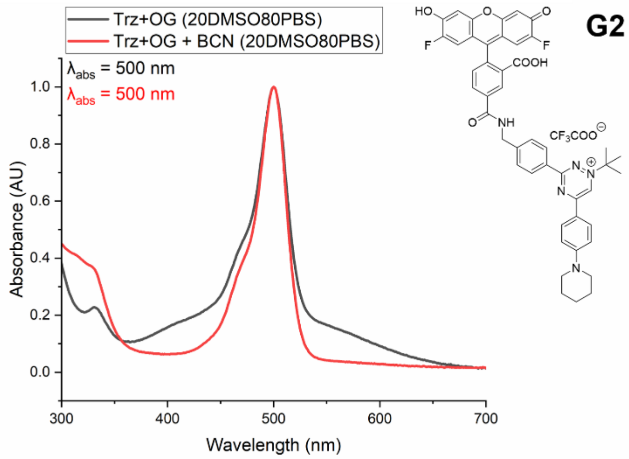

G2

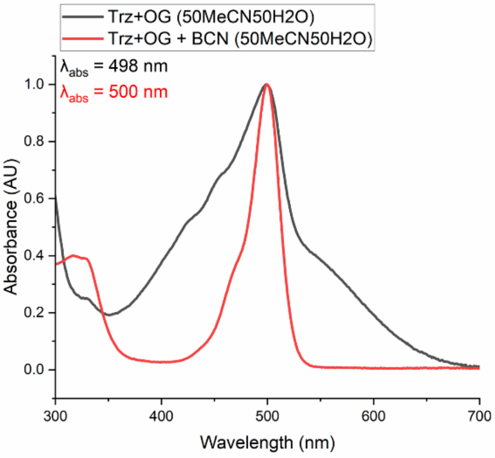

H1

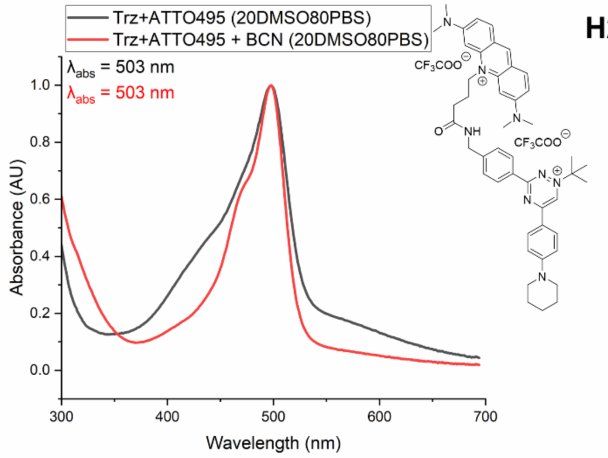

H2

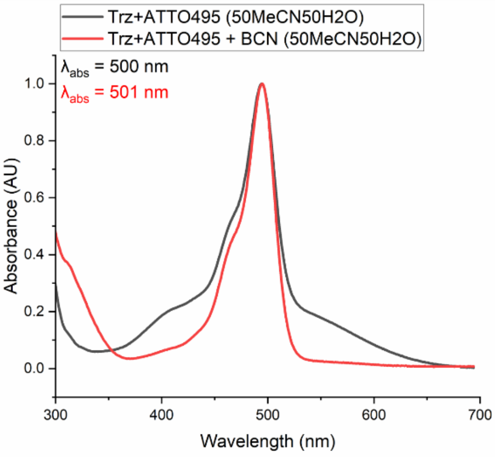

I1

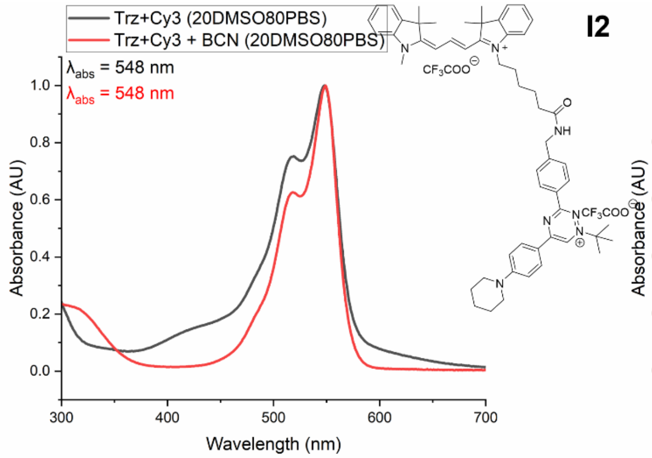

I2

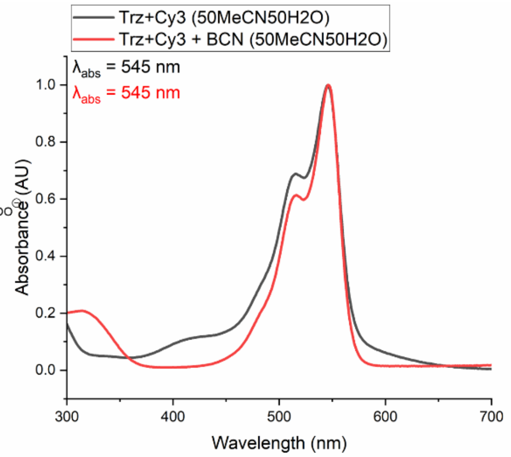

J1

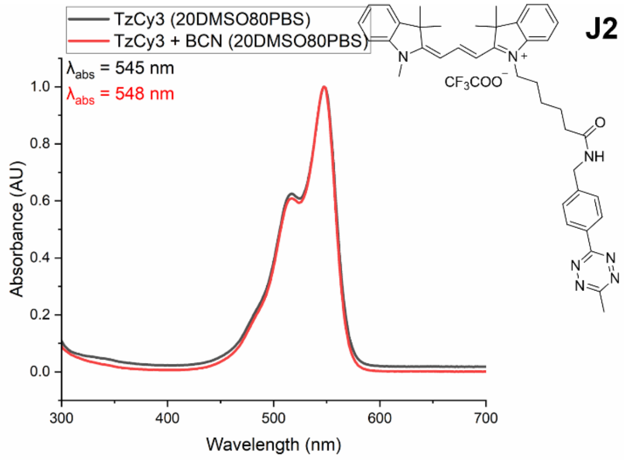

J2

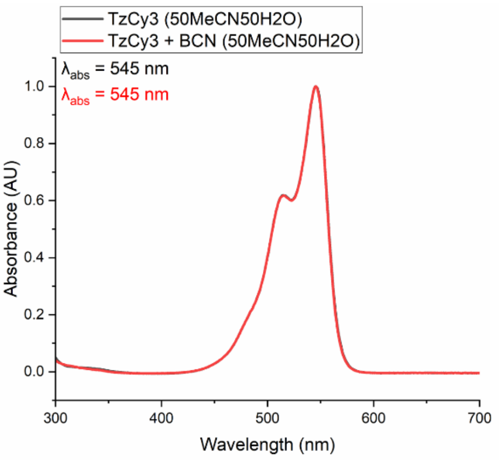

K1

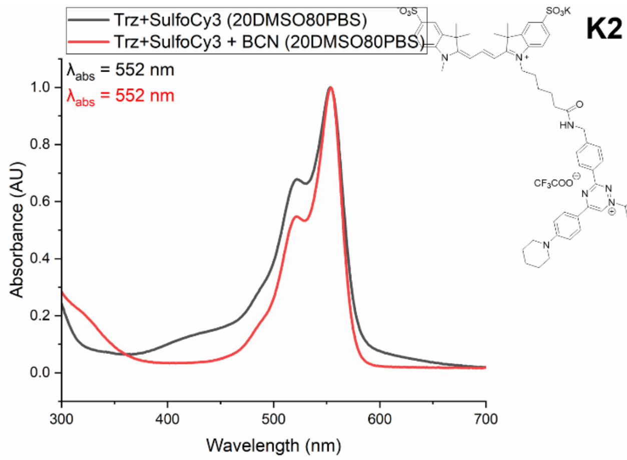

K2

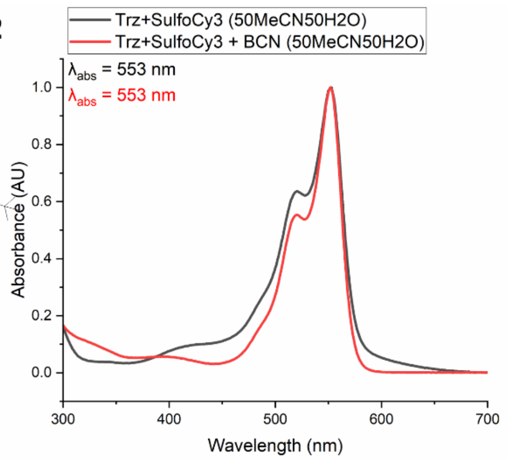

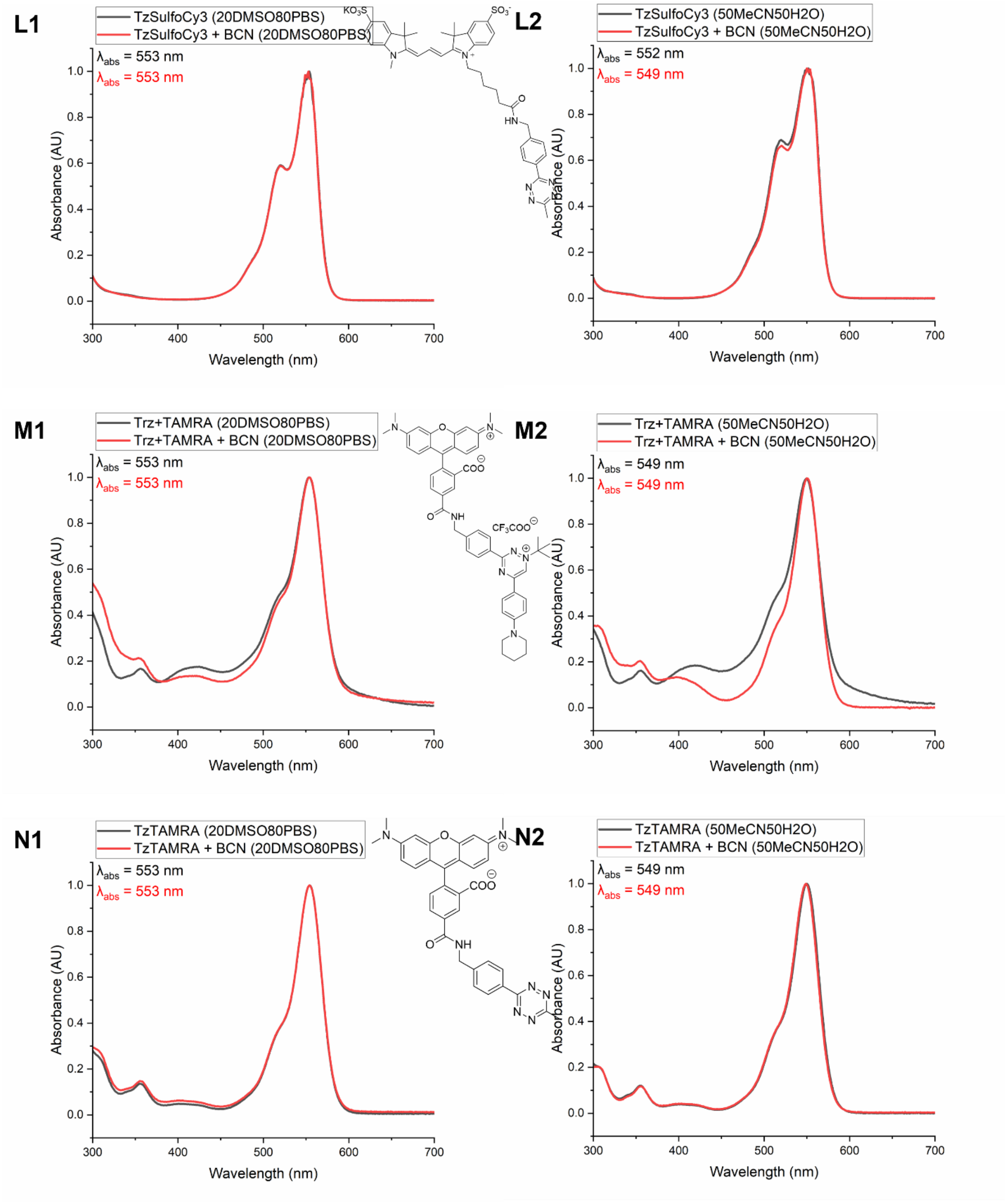
L1
TzSulfoCy3 (20DMSO80PBS)
TzSulfoCy3 + BCN (20DMSO80PBS)
λabs = 553 nm
λabs = 553 nm
Absorbance (AU)
Wavelength (nm)
L2
TzSulfoCy3 (50MeCN50H2O)
TzSulfoCy3 + BCN (50MeCN50H2O)
λabs = 552 nm
λabs = 549 nm
M1
Trz+TAMRA (20DMSO80PBS)
Trz+TAMRA + BCN (20DMSO80PBS)
λabs = 553 nm
λabs = 553 nm
M2
Trz+TAMRA (50MeCN50H2O)
Trz+TAMRA + BCN (50MeCN50H2O)
λabs = 549 nm
λabs = 549 nm
N1
TzTAMRA (20DMSO80PBS)
TzTAMRA + BCN (20DMSO80PBS)
λabs = 553 nm
λabs = 553 nm
N2
TzTAMRA (50MeCN50H2O)
TzTAMRA + BCN (50MeCN50H2O)
λabs = 549 nm
λabs = 549 nm

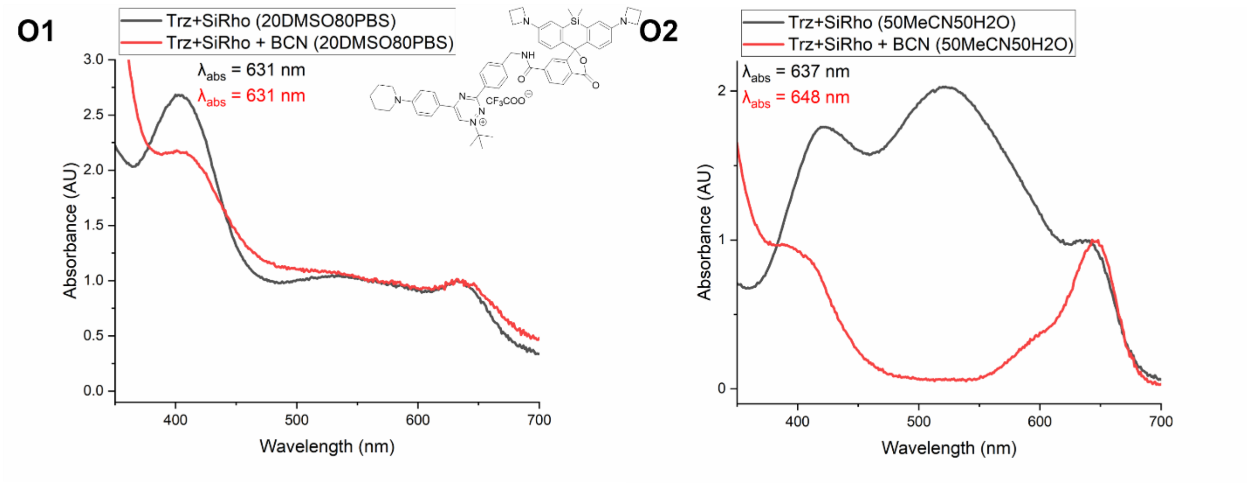

Figure S2: Absorbance spectra of **T(r)z$^{(+)}$Fluorophores** and their click products formed in the reaction with ***endo*-BCN** in DMSO/PBS buffer (1:4) r or MeCN/H_2O (1:1). Absorbance spectra were normalized to the highest signal of each fluorophore. **ArTrz$^+$1** = **Trz$^+$1**.

Absorption spectra

Absorbance measurement was performed as follows: 1 µL of the 10 mM stock solution in DMSO/H_2O (1:1) of each probe was pre-mixed in 200 µL of 100% DMSO or MeCN/H_2O (1:1) and diluted into 800 µL of the 100% PBS buffer (1×, pH = 7.4) or MeCN/H_2O (1:1) to the final 10 µM concentration. It was necessary to use **MeTzNH^2** at the final 50 µM concentration. For the The cuvette was immediately inserted into the spectrophotometer, and the measurement was started (Figure S3). The absorbance of the corresponding click products formed in the reaction with ***endo*-BCN** was measured the same. Prior the final dilution, the pre-mixed mixture was incubated with ***endo*-BCN** (1 µL from 100 mM fresh stock in MeCN/H_2O (1:1)) ca. 30 min to ensure complete conversion. The plots are depicted in Figure S3.

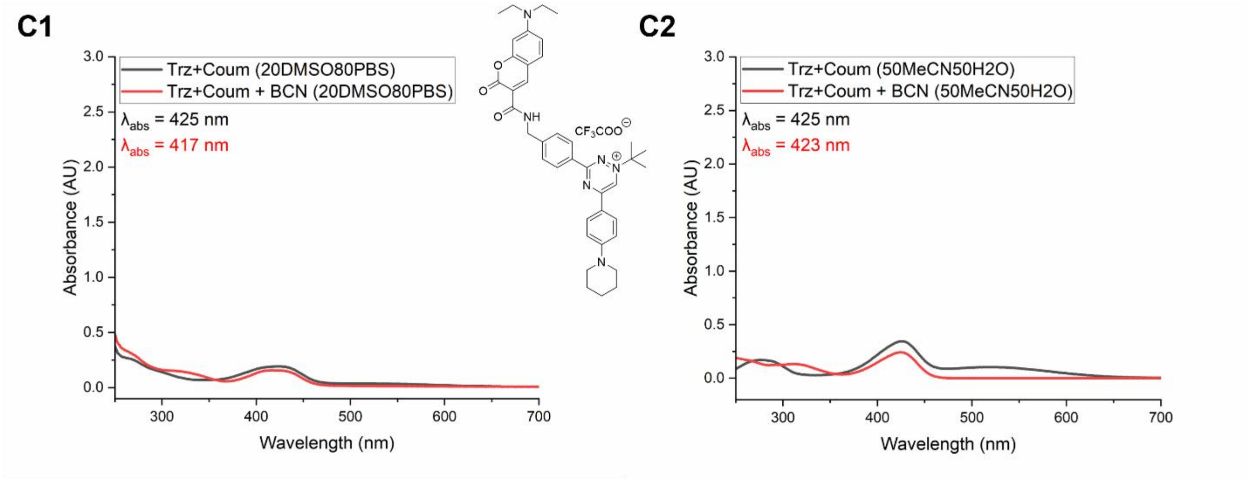

D1

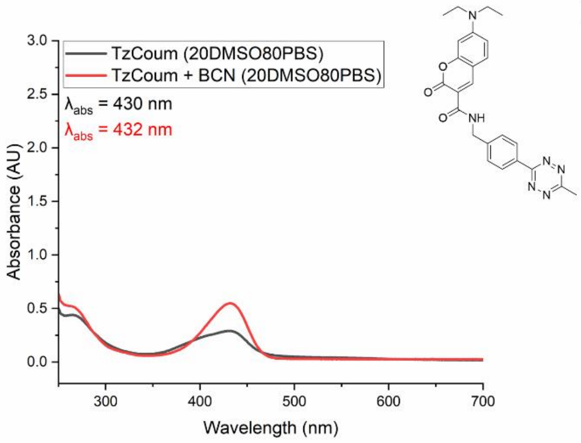

D2

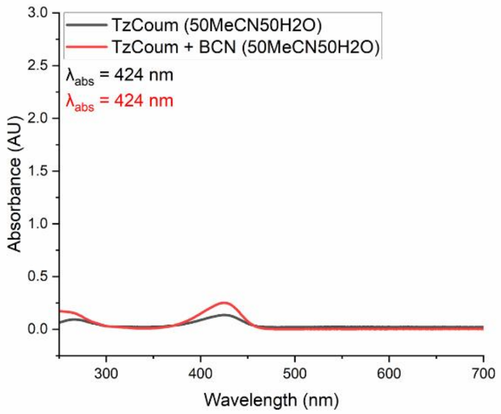

E1

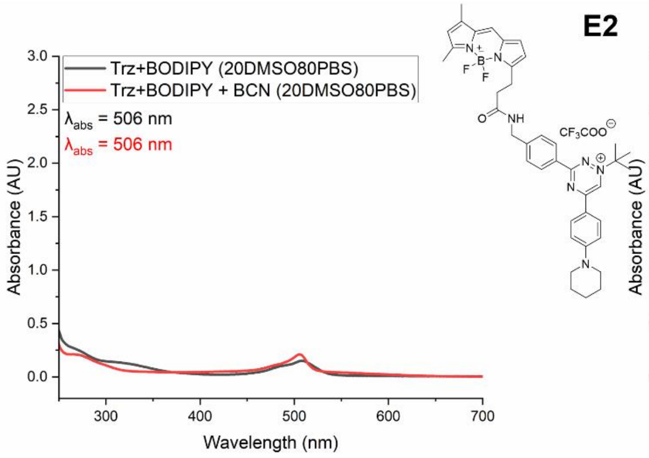

E2

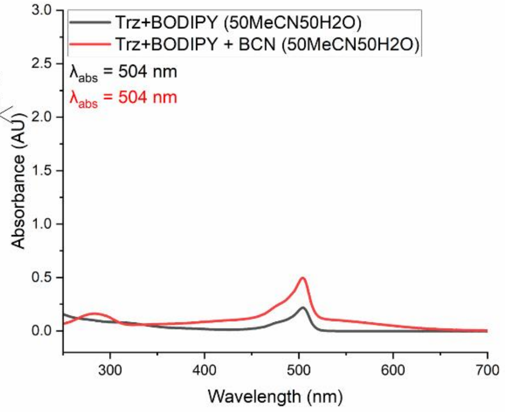

F1

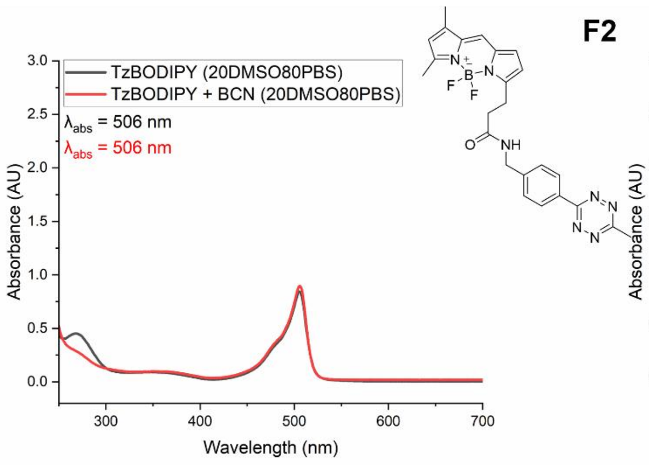

F2

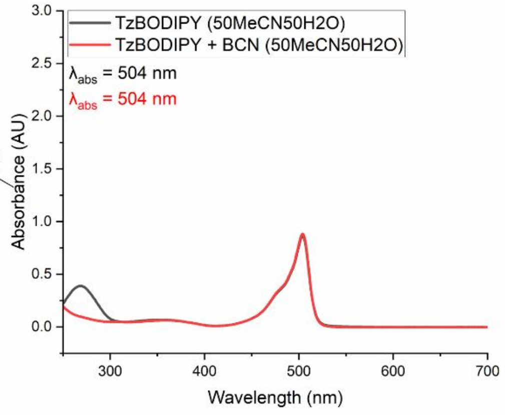

G1

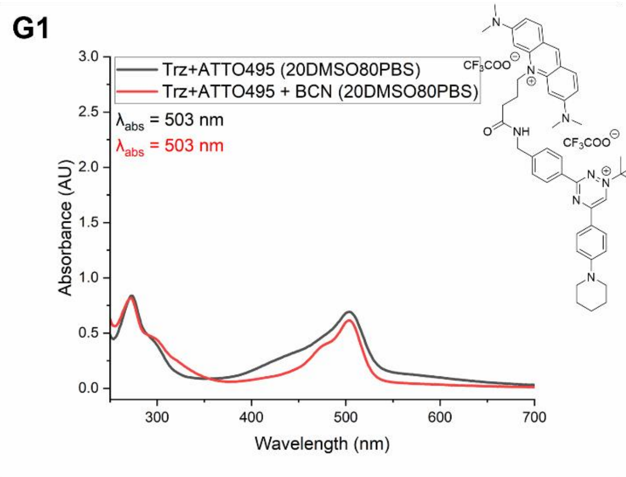

G2

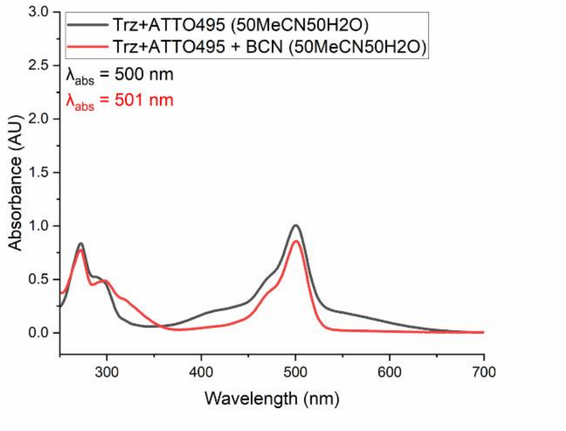

H1

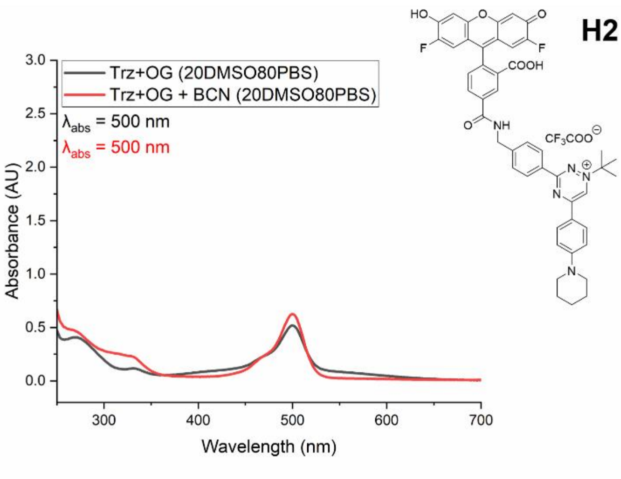

H2

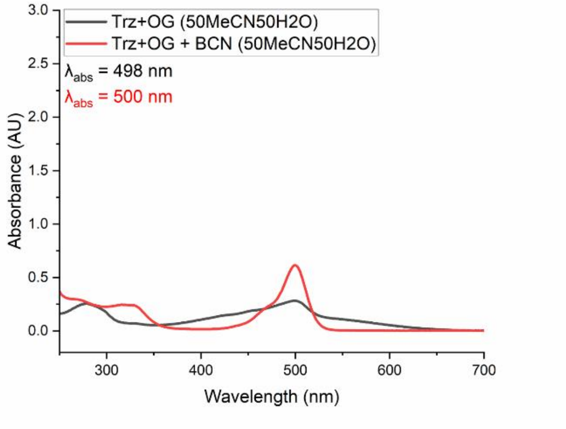

I1

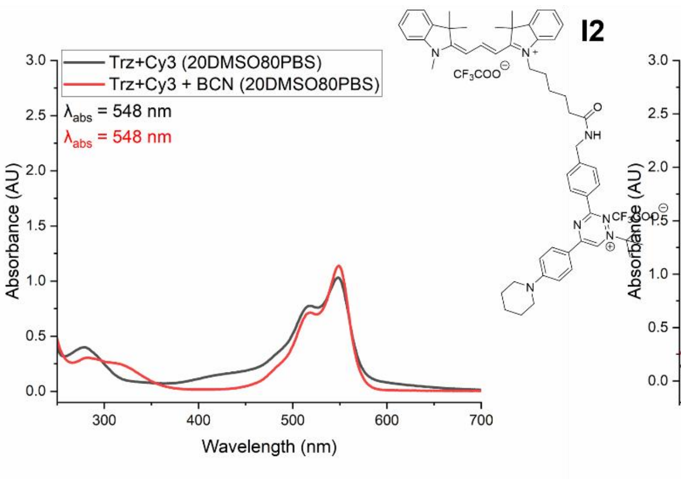

I2

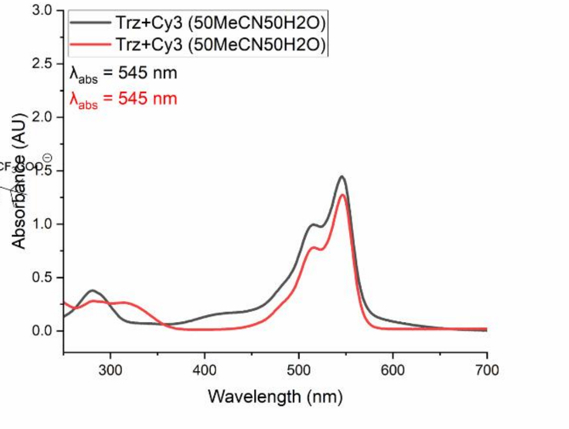

J1

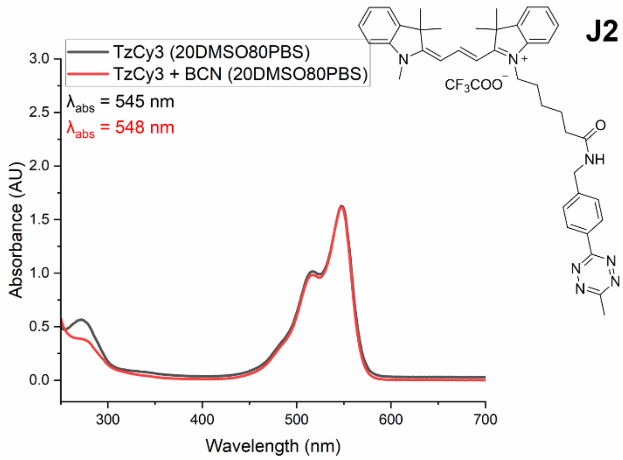

J2

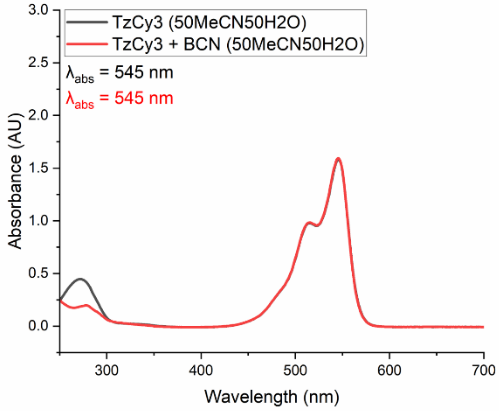

K1

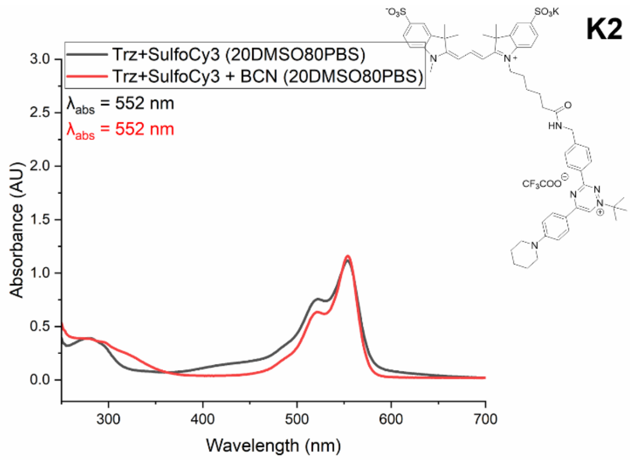

K2

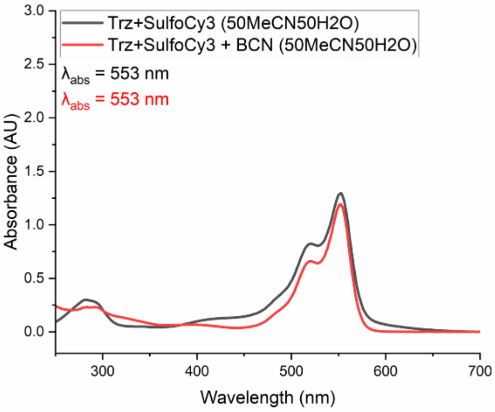

L1

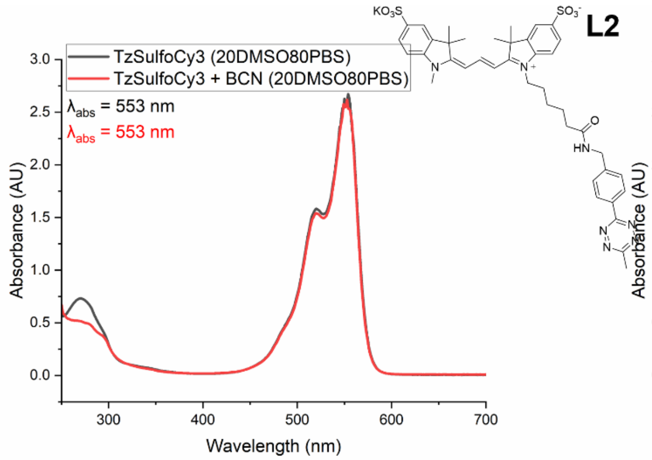

L2

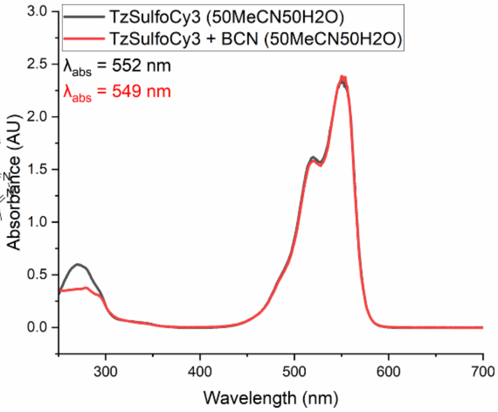

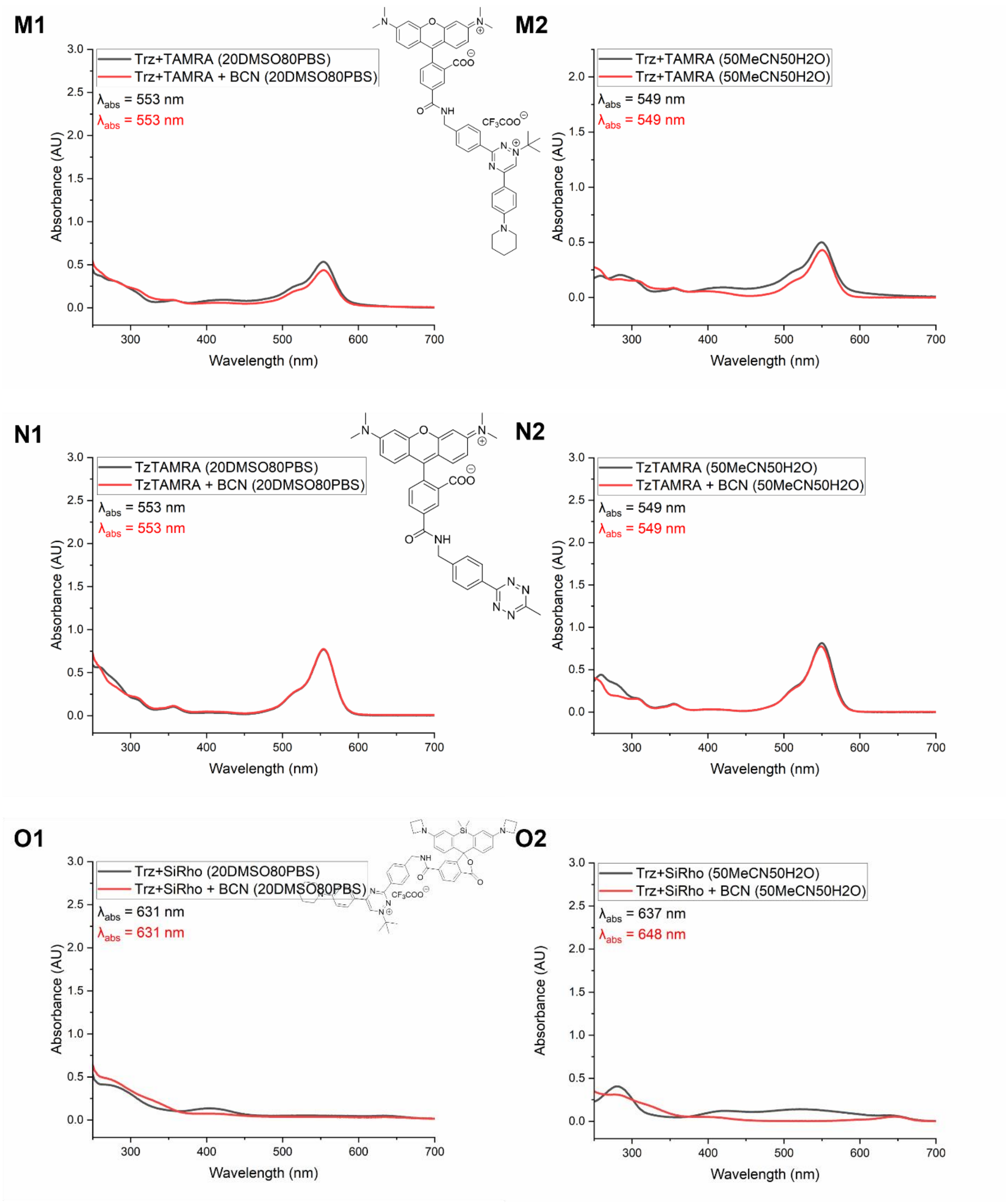

Figure S3: Absorbance spectra of **T(r)z[(+)]Fluorophores** and their click products formed in the reaction with ***endo*-BCN** in DMSO/PBS buffer (1:4) or MeCN/H_2O (1:1). Absorbance spectra were not normalized to the highest signal of each fluorophore.

Determination of extinction coefficients

Extinction coefficients for T(r)z$^{(+)}$ quenchers were determined by their absorbance measurement at 4 different concentrations. Absorbance measurement was performed as follows: 0.5, 1.0, 1.5, or 2.0 μL of the 10 mM stock solution in DMSO/H_2O (1:1) of **Trz$^+$1** was diluted up to 1.00 mL of the MeCN/H_2O (1:1) to the final 5, 10, 15 or 20 μM concentration. The cuvette was inserted into the spectrophotometer and the measurement was started. The plot is depicted in Graph S1. Absorbance measurement of **MeTzNH2** was adjusted (due to low absorptivity) as follows: 5, 10, 15, or 20 μL of the 10 mM stock solution in DMSO/H_2O (1:1) of **MeTzNH2** was diluted up to 1.00 mL of the MeCN/H_2O (1:1) to the final 50, 100, 150 or 200 μM concentration. All spectra were recorded in three independent measurements for each compound. Each absorbance value of the corresponding T(r)z$^{(+)}$ at absorption maximum was measured and plotted against concentration (fitted with single linear function: y = ax + b).

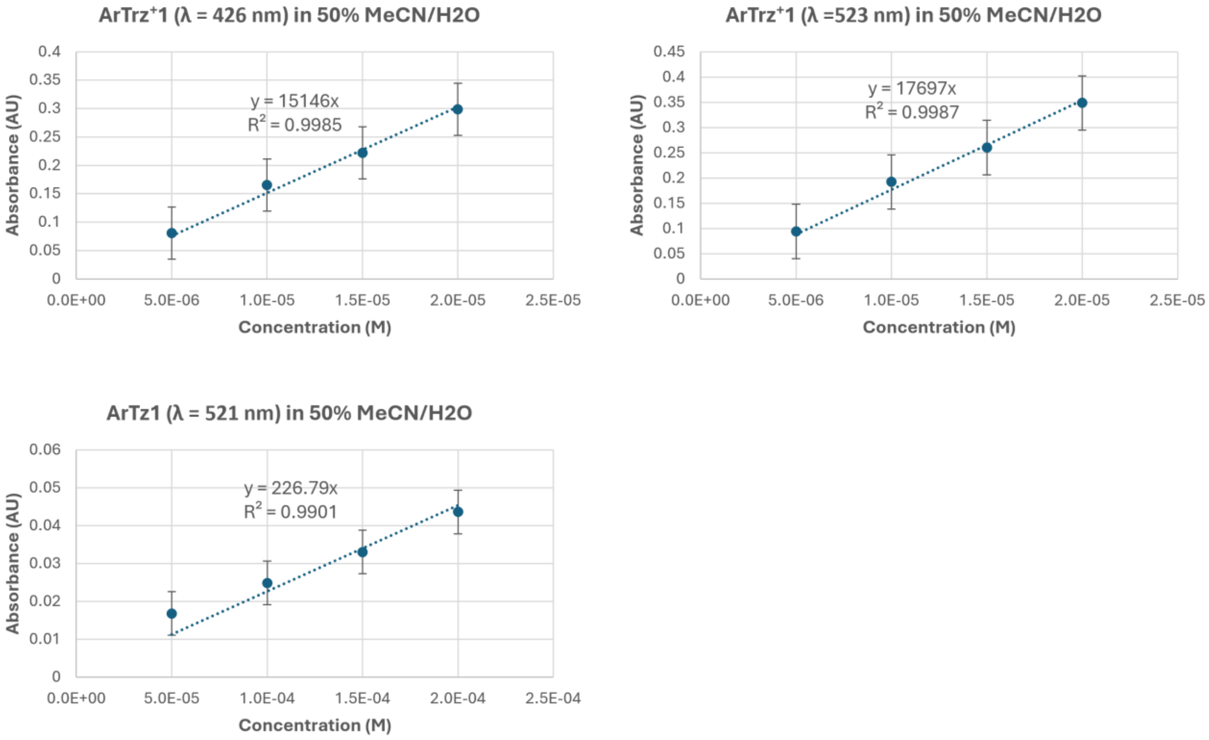

Graph S1: Graphs from determination of extinction coefficient of T(r)z$^{(+)}$. **ArTrz$^+$1** = **Trz$^+$1**.

Fluorescence turn-on measurements

All triaziniums and tetrazines were purified by analytical HPLC prior to turn-on fluorescence measurements since traces of fluorescent impurities can significantly influence the maximum observable ratio.[8] Analytically pure samples were used directly for the measurements and were stored in the dark on ice before analysis. The impurities can be best seen using fluorescence detection on the HPLC. Representative HPLC analysis before and after purification of the dyes is depicted in Figure S4 (showing compound **Trz$^+$Coum** as an example).

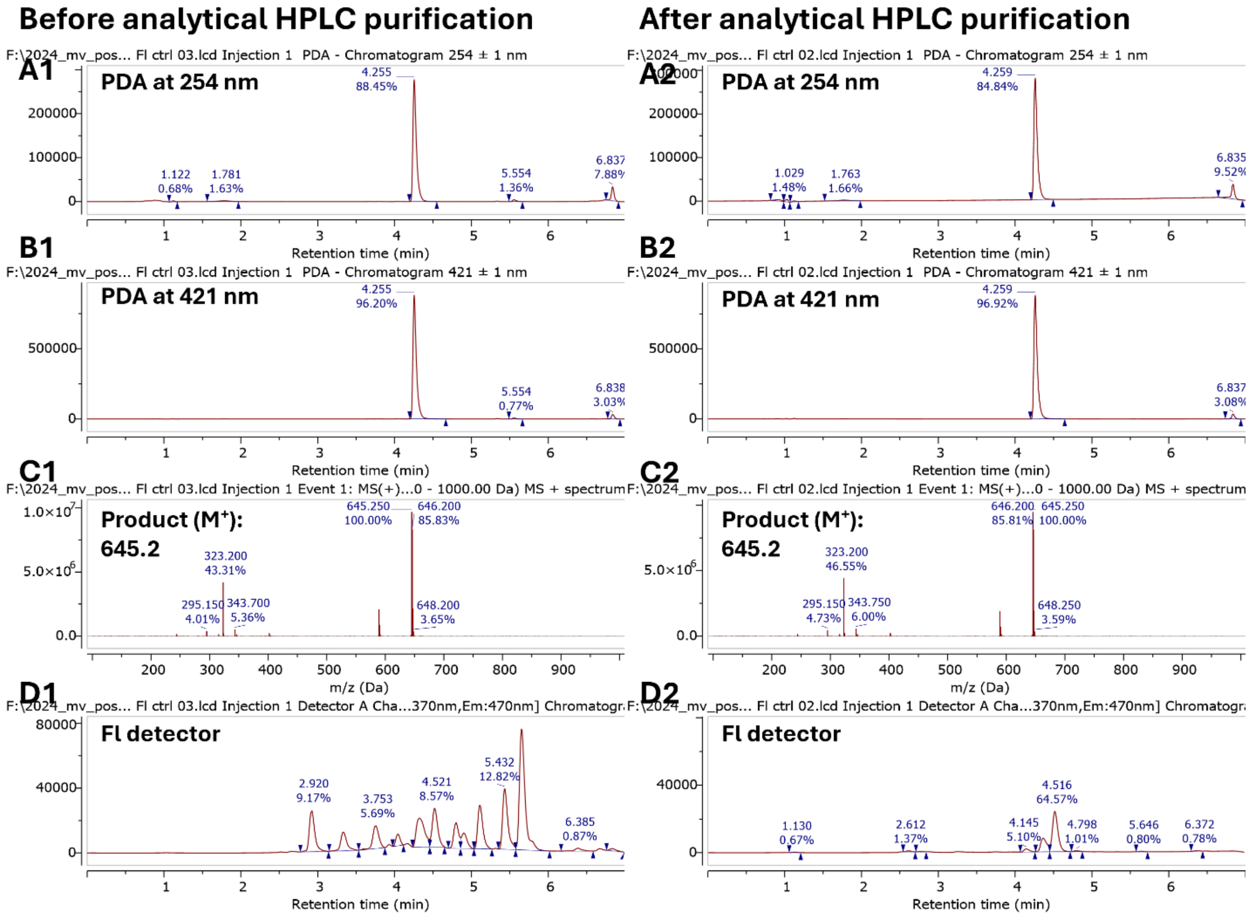

Figure S4: Exported HPLC-MS chromatograms of the selected **Trz⁺Coum** before (left) and after (right) the analytical HPLC purification recorded at (A) λ = 254 nm or (B) at characteristic absorption λ =421 nm with (C) the corresponding MS chromatogram including (D) the corresponding analysis from fluorescence detector.

The turn-on fluorescence measurements were performed as follows: 5 µL of the 500 µM sample collected from the analytical HPLC (typical final concentration approximately 50 µM) was pre-mixed in 100 µL 20% DMSO in PBS buffer (1×, pH = 7.4) or MeCN/H_2O (1:1) and solution of blank or ***endo*-BCN** (5 µL from 100 mM fresh stock in MeCN/H_2O (1:1)) was added. The mixture was then incubated ca. 30 min to ensure complete conversion and 70 µL diluted in 2.0 mL 20% DMSO in PBS buffer (1×, pH = 7.4) or MeCN/H_2O (1:1). The cuvette was inserted into fluorescence spectrophotometer and the measurement was started. After the control absorbance measurement, fluorescence emission was measured using the following setup. All probes were excited in accordance with their characteristic absorption (Table S1) as follows: 515 nm for **TAMRA**, 470 nm for **ATTO495, OG,** and **BODIPY,** 520 nm for **(Sulfo)Cy3**, 630 nm for **SiRho** and 400 nm for **Coum** dyes, respectively. The fluorescence was collected from 535 nm for **TAMRA**, 490 nm for **ATTO495, OG** and **BODIPY,** 540 nm for **(Sulfo)Cy3**, 640 nm for **SiRho,** and 420 nm for **Coum** dyes, up to 750 or 800 nm. All spectra were recorded in three independent measurements for each compound. The data were processed using OriginPro 9.1 software and Microsoft® Excel® pro Microsoft 365 MSO (Version 2408 Build 16.0.17928.20114) 32 bites. All spectra were subtracted from the baseline (20% DMSO in PBS buffer or MeCN/H_2O (1:1) as the blank). The turn-on values were finally calculated (A) by dividing the fluorescence intensity of the click product at the emission maximum by the average of the residual fluorescence of the quenched

Trz⁺Fluorophor before the reaction, (B) from the ratio of the corresponding integrated areas under the respective curve of fluorescence emission of the click product or the quenched **Trz⁺Fluorophor**, (C) by dividing the quantum yield of the fluorescence of the click product by the quantum yield of the quenched **Trz⁺Fluorophor**. Finally, a graph from the averaged values was plotted, and an average turn-on value was calculated from the three independent measurements. The plot is depicted in Figure S5, and data is summarized in Table S1 and S2. The highest fluorescence signal intensities for reaction with the ***endo*-BCN** are listed. Also, the energy of 0-0 transition was determined by plotting normalized absorption and emission spectra.

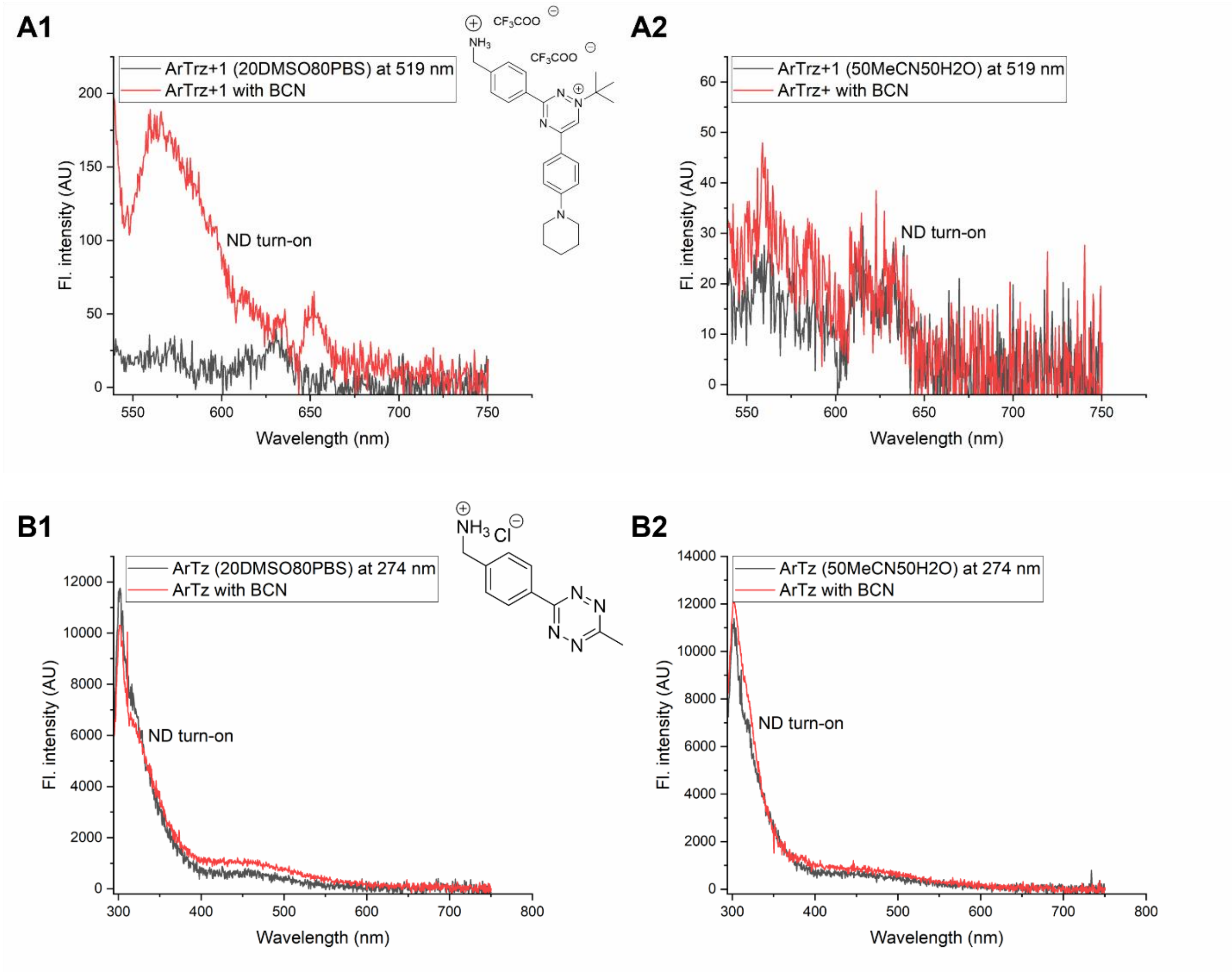

C1

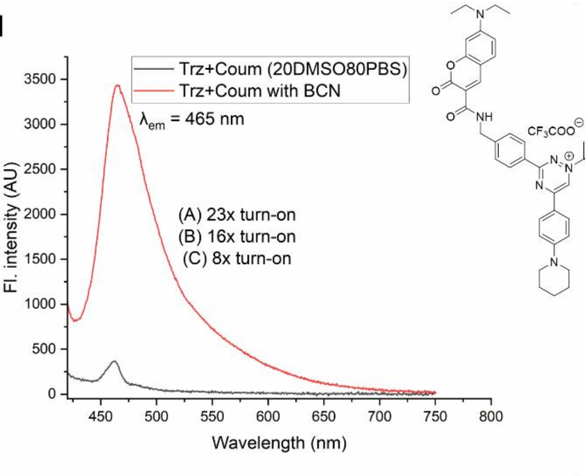

C2

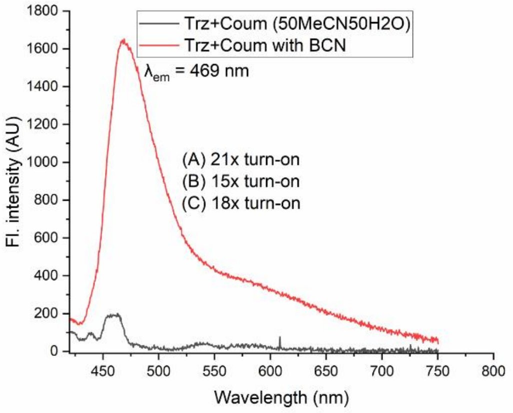

D1

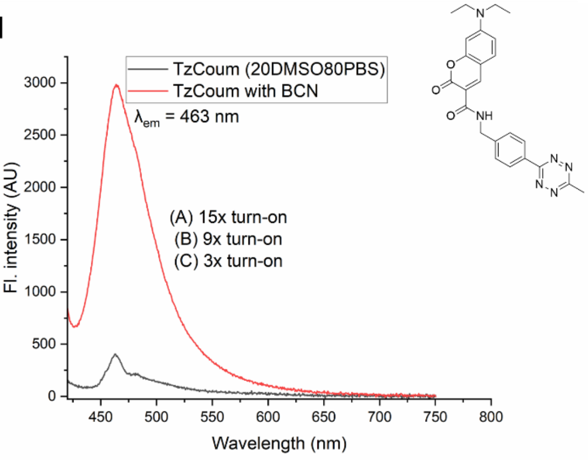

D2

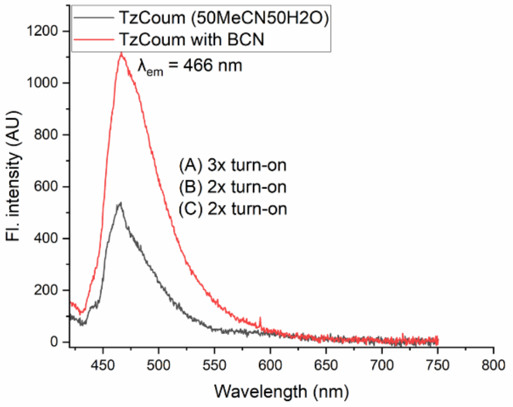

E1

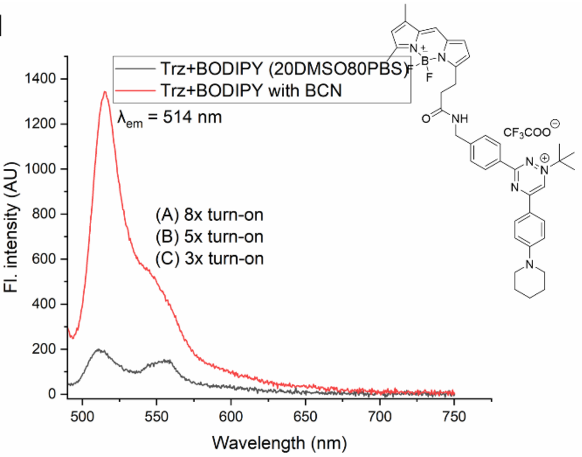

E2

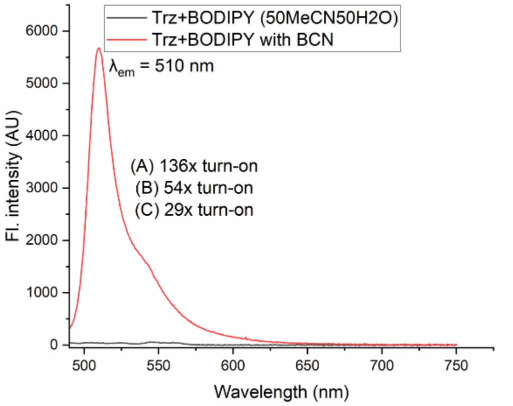

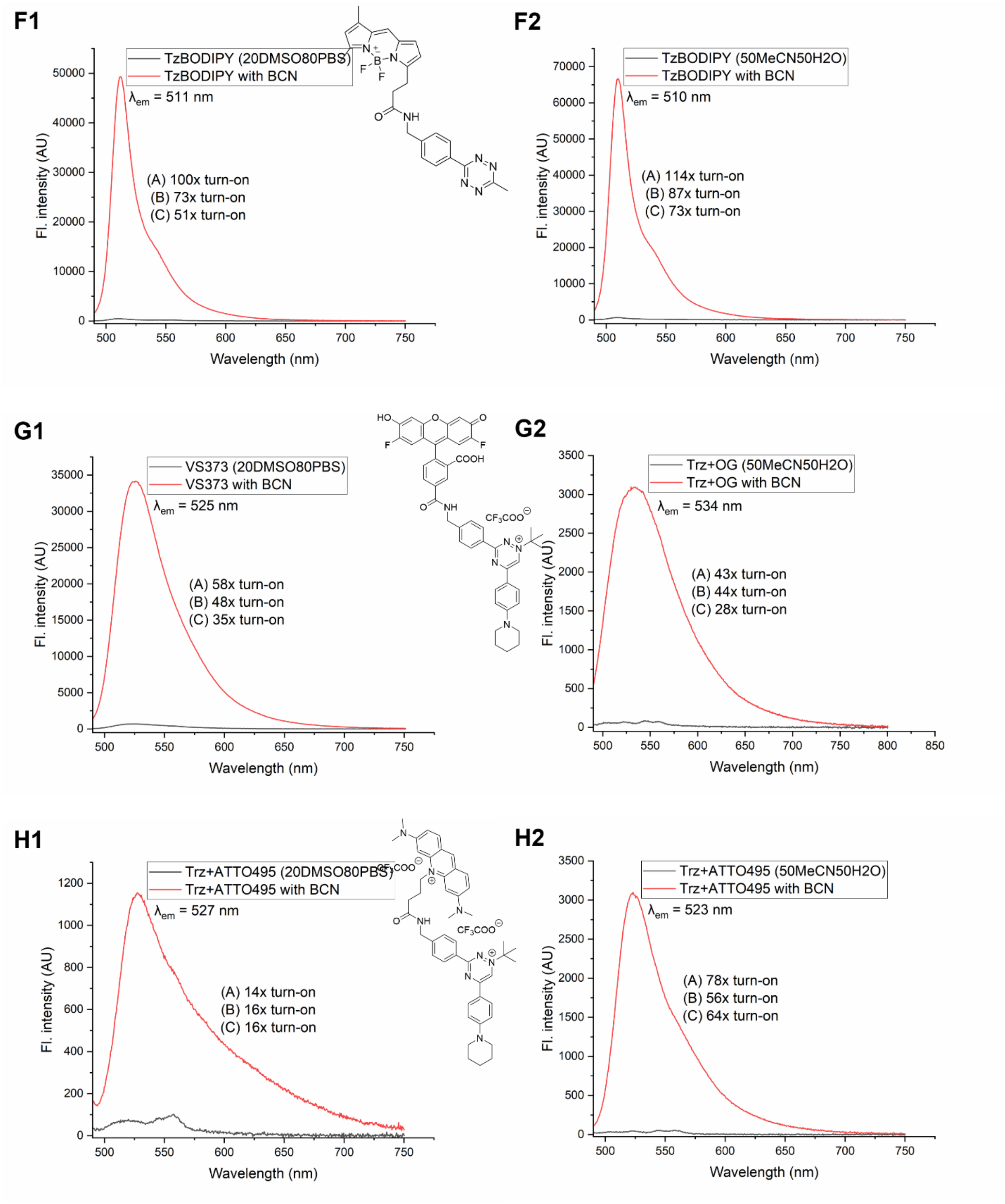
F1
TzBODIPY (20DMSO80PBS)
TzBODIPY with BCN
λem = 511 nm
(A) 100x turn-on
(B) 73x turn-on
(C) 51x turn-on
Fl. intensity (AU)
Wavelength (nm)
F2
TzBODIPY (50MeCN50H2O)
TzBODIPY with BCN
λem = 510 nm
(A) 114x turn-on
(B) 87x turn-on
(C) 73x turn-on
Fl. intensity (AU)
Wavelength (nm)
G1
VS373 (20DMSO80PBS)
VS373 with BCN
λem = 525 nm
(A) 58x turn-on
(B) 48x turn-on
(C) 35x turn-on
Fl. intensity (AU)
Wavelength (nm)
G2
Trz+OG (50MeCN50H2O)
Trz+OG with BCN
λem = 534 nm
(A) 43x turn-on
(B) 44x turn-on
(C) 28x turn-on
Fl. intensity (AU)
Wavelength (nm)
H1
Trz+ATTO495 (20DMSO80PBS)
Trz+ATTO495 with BCN
λem = 527 nm
(A) 14x turn-on
(B) 16x turn-on
(C) 16x turn-on
Fl. intensity (AU)
Wavelength (nm)
H2
Trz+ATTO495 (50MeCN50H2O)
Trz+ATTO495 with BCN
λem = 523 nm
(A) 78x turn-on
(B) 56x turn-on
(C) 64x turn-on
Fl. intensity (AU)
Wavelength (nm)

I1

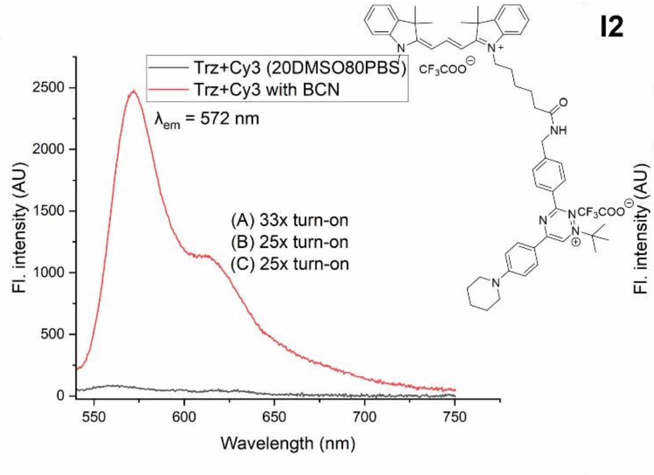

I2

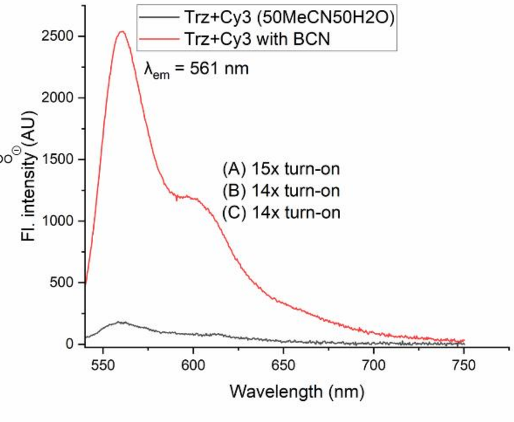

J1

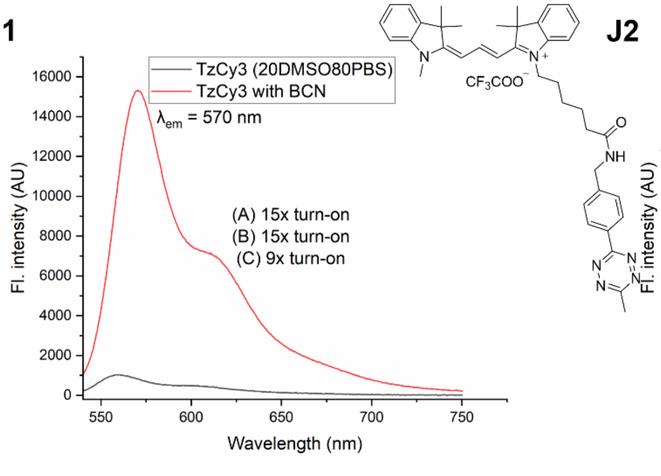

J2

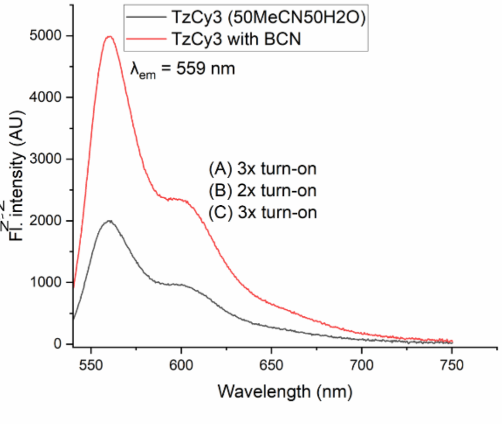

K1

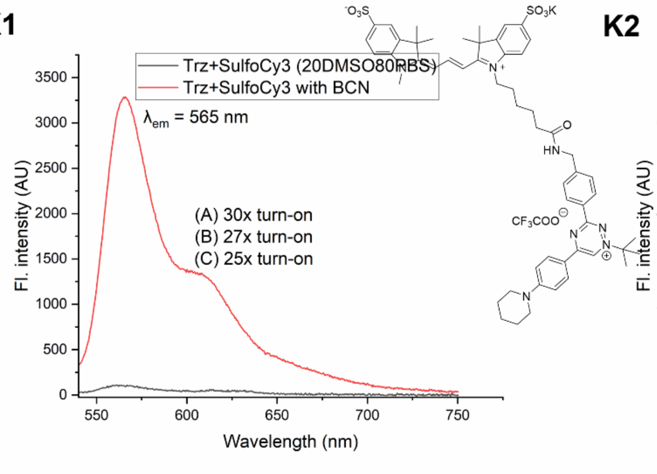

K2

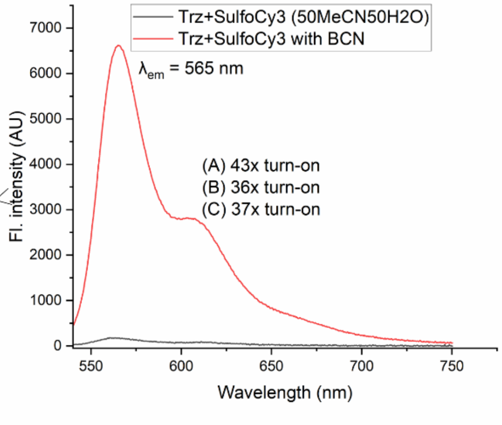

L1

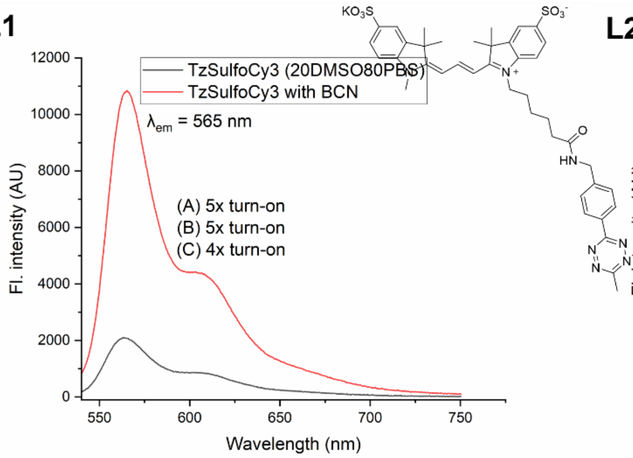

L2

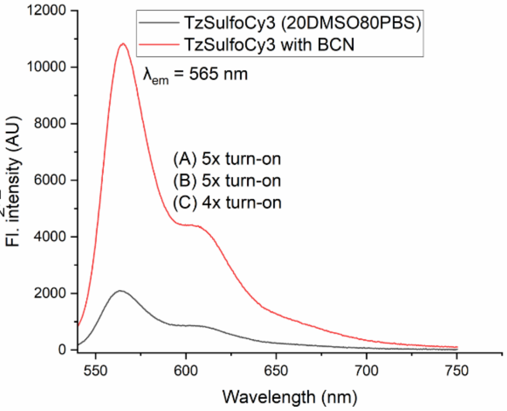

M1

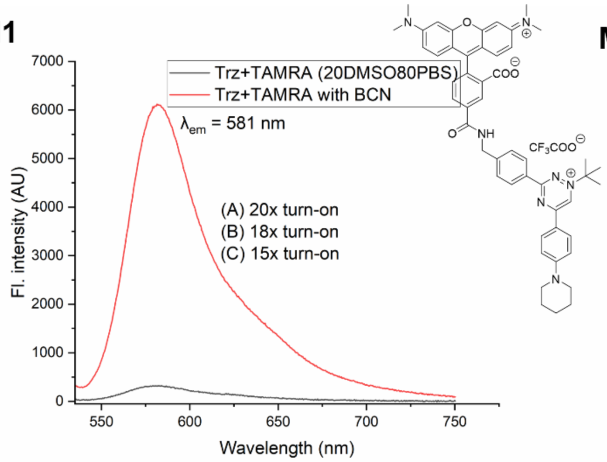

M2

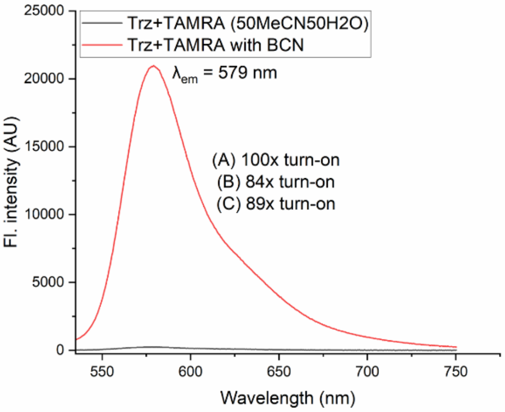

N1

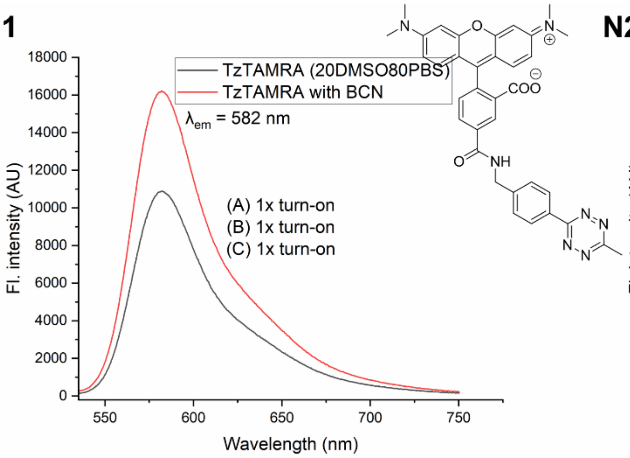

N2

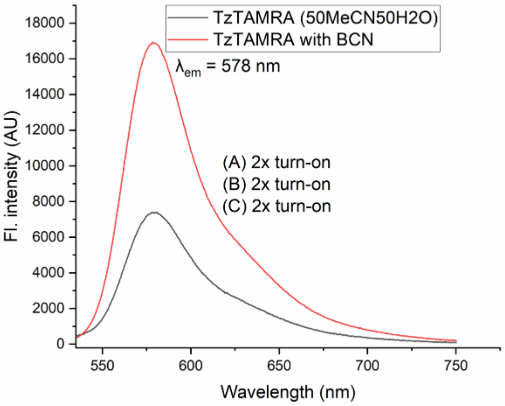

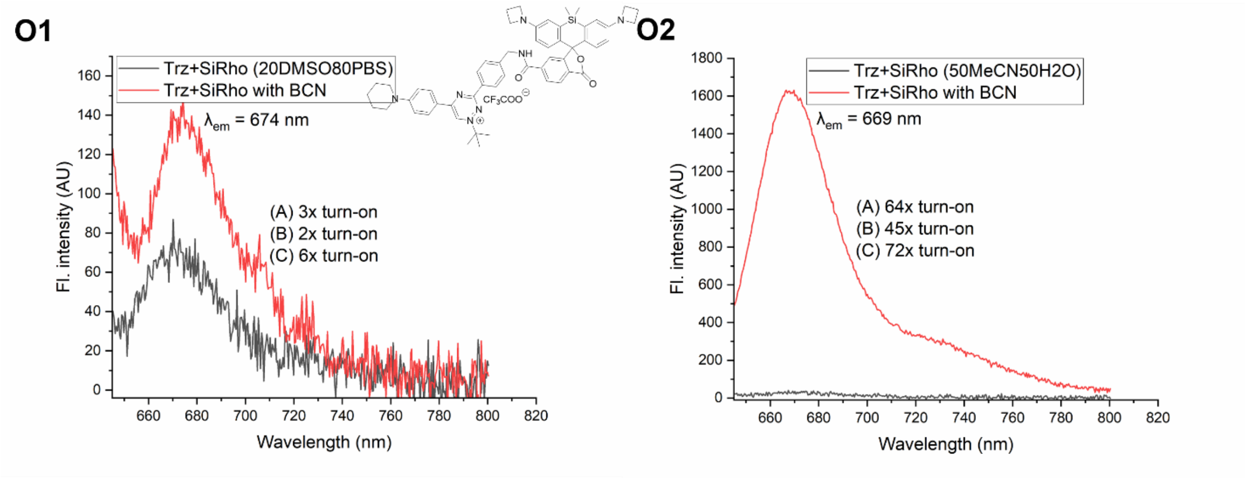

Figure S5: Fluorescence spectra of the **Tz** and **Trz⁺Fluorophores** and their click products formed in the reaction with ***endo*-BCN** (indicated as number-x = x-fold) in PBS buffer or MeCN/H_2O (1:1). **ArTrz⁺1** = **Trz⁺1**.

Table S1: Summary of the Abs. and Em. maxima and quantum yields of the T(r)z$^{(+)}$Fl and their click products in DMSO/PBS (1:4) or MeCN/H_2O (1:1)

T(r)z$^{(+)}$Fl	λ_{Abs} (nm) T(r)z$^{(+)}$		λ_{Abs} (nm) T(r)z$^{(+)}$+BCN		λ_{Em} (nm)		Φ_f^a T(r)z$^{(+)}$		Φ_f^a T(r)z$^{(+)}$+BCN	
**ArTrz⁺1**	272<br>425<br>523	275<br>426<br>523	330<br>421<br>517	310<br>409<br>530	-	-	ND	ND	ND	ND
**ArTz1**	261<br>320<br>532	262<br>320<br>521	276	276	-	-	ND	ND	ND	ND
**Trz⁺Coum**	425	425	417	423	465	469	0.0029	0.0008	0.024	0.0109
**TzCoum**	430	424	432	424	463	466	0.0191	0.0107	0.053	0.022
**Trz⁺BODIPY**	508	504	506	504	514	510	0.0190	0.0053	0.064	0.151
**TzBODIPY**	506	504	506	504	511	510	0.0139	0.0064	0.713	0.469
**Trz⁺OG**	500	498	500	500	525	534	0.0194	0.0128	0.680	0.362
**Trz⁺ATTO495**	503	500	503	501	527	523	0.0084	0.0013	0.131	0.083
**Trz⁺Cy3**	548	545	548	545	572	561	0.0014	0.0103	0.0351	0.1424
**Tz Cy3**	545	545	548	545	570	559	0.0143	0.0733	0.1278	0.1884
**Trz⁺SulfoCy3**	552	553	552	553	565	565	0.0011	0.0075	0.0269	0.2743
**Tz SulfoCy3**	553	552	553	549	565	565	0.0253	0.1698	0.0975	0.3478
**Trz⁺TAMRA**	553	549	553	549	581	579	0.0018	0.0011	0.026	0.098
**TzTAMRA**	553	549	553	549	582	578	0.0851	0.055	0.098	0.112
**Trz⁺SiRho**	631	637	631	648	674	669	0.1267	0.0085	0.726	0.614

[a]Determined from the indicated emission spectra using 50 nM solution of the corresponding commercial dye as the standard.

Table S2: Summary of the various turn-on values of the T(r)z[(+)]Fl and their click products in DMSO/PBS (1:4) or $MeCN/H_2O$ (1:1)

T(r)z[(+)]Fl	Fluorescence turn-on[a]		Fluorescence turn-on[b]		Fluorescence turn-on[c]		0-0 Transition energy T(r)z[(+)]		0-0 Transition energy T(r)z[(+)]+BCN	
**Trz[+]Coum**	23	21	16	15	8	18	453	448	448	447
**TzCoum**	15	3	9	2	3	2	453	449	449	448
**Trz[+]BODIPY**	8	136	5	54	3	29	511	511	510	507
**TzBODIPY**	100	114	73	87	51	73	508	506	509	507
**Trz[+]OG**	58	43	48	44	35	28	510	517	510	508
**Trz[+]ATTO495**	14	78	16	56	16	64	516	514	514	510
**Trz[+]Cy3**	33	15	25	14	25	14	553	552	559	552
**Tz Cy3**	15	3	15	2	9	3	554	552	558	552
**Trz[+]SulfoCy3**	30	43	27	36	25	37	559	558	559	557
**Tz SulfoCy3**	5	2	5	2	4	2	557	558	558	558
**Trz[+]TAMRA**	20	100	18	84	15	89	567	562	567	563
**TzTAMRA**	1	2	1	2	1	2	566	563	566	562
**Trz[+]SiRho**	3	64	2	45	6	72	660	662	664	660

[a]Turn-on values determined by dividing the fluorescence intensity of the click product at the emission maximum by the average of the residual fluorescence of the quenched fluorophore before the reaction. [b]Turn-on values determined by dividing the integrated area of the fluorescence of the click product by the integrated area of the quenched fluorophore. [c]Turn-on values determined by dividing the quantum yield of the fluorescence of the click product by the quantum yield of the quenched fluorophore.

Fluorescence time-lapse measurements

The turn-on fluorescence measurements were performed as follows: 2.5 µL of the approximately 50 µM sample collected from the analytical HPLC (typical final concentration approximately 5 µM) was diluted in 1.0 mL of 20% DMSO in PBS buffer (1×, pH = 7.4) or $MeCN/H_2O$ (1:1). The cuvette was immediately inserted into fluorescence spectrophotometer and the measurement was started. All probes were excited in accordance with their characteristic absorption (Table S1) as follows: 515 nm for **TAMRA**, 470 nm for **ATTO495, OG,** and **BODIPY,** 520 nm for **(Sulfo)Cy3**, 630 nm for **SiRho,** and 400 nm for **Coum** dyes, respectively. The fluorescence was collected every 20 seconds at the corresponding λ_{em} for 15 or 30 min in three independent measurements for each compound. After 1 min, the cuvette was taken out for ca. 40 s and a solution of ***endo*-BCN** (5 µL from 100 mM fresh stock in $MeCN/H_2O$ (1:1)) was added and measurement continued. (FigureS6).

A1

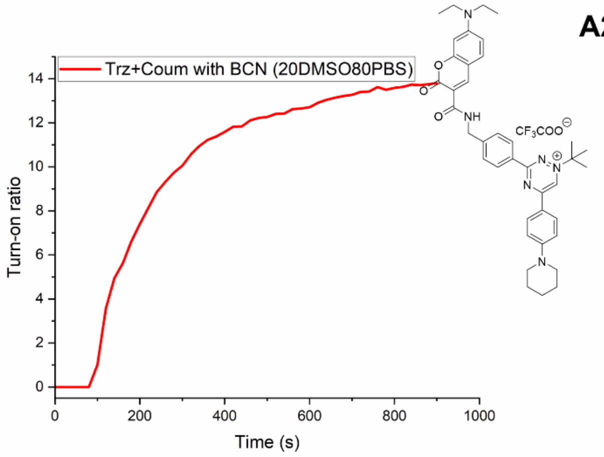

A2

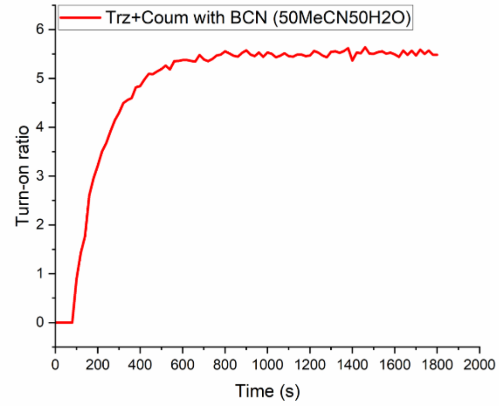

B1

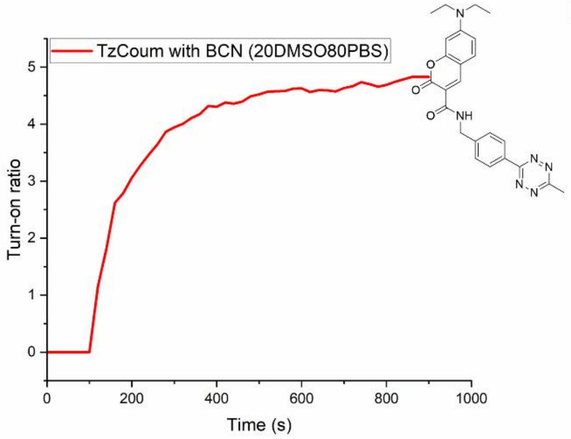

B2

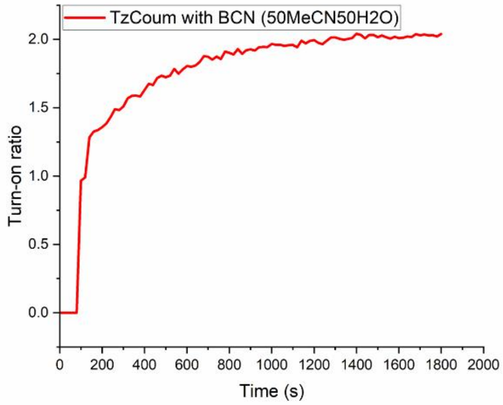

C1

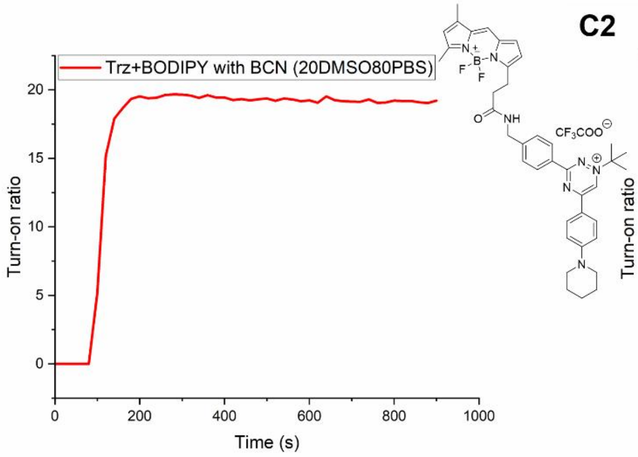

C2

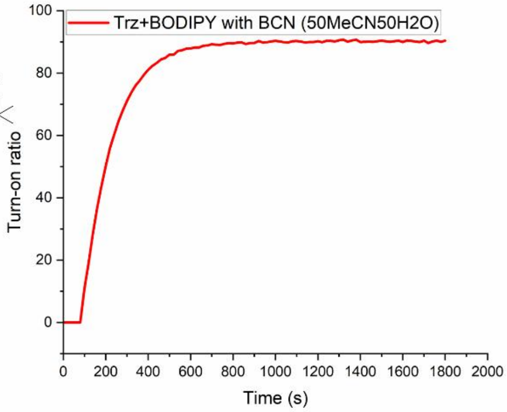

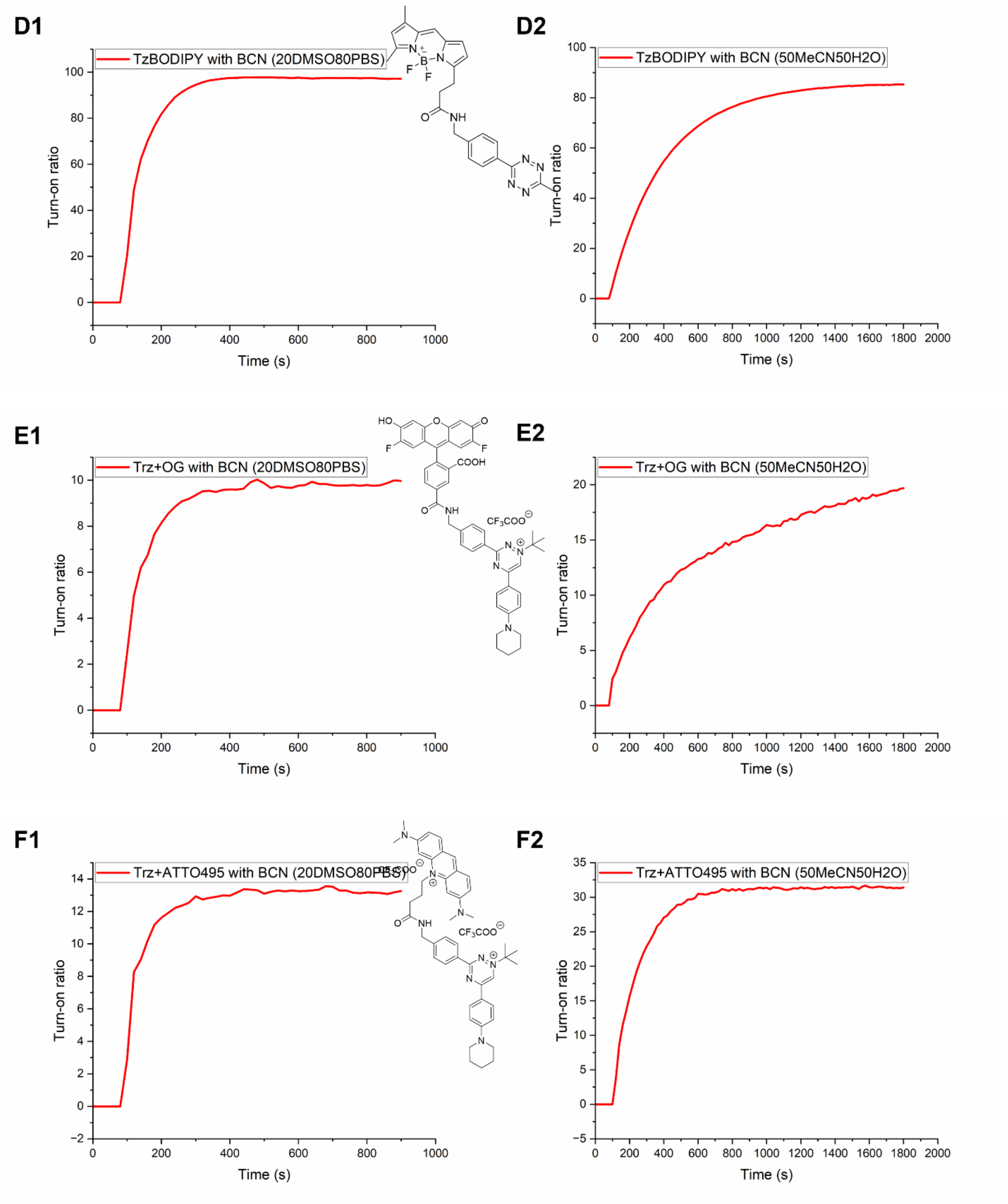
D1
TzBODIPY with BCN (20DMSO80PBS)
Turn-on ratio
Time (s)
D2
TzBODIPY with BCN (50MeCN50H2O)
Turn-on ratio
Time (s)
E1
Trz+OG with BCN (20DMSO80PBS)
Turn-on ratio
Time (s)
E2
Trz+OG with BCN (50MeCN50H2O)
Turn-on ratio
Time (s)
F1
Trz+ATTO495 with BCN (20DMSO80PBS)
Turn-on ratio
Time (s)
F2
Trz+ATTO495 with BCN (50MeCN50H2O)
Turn-on ratio
Time (s)

G1

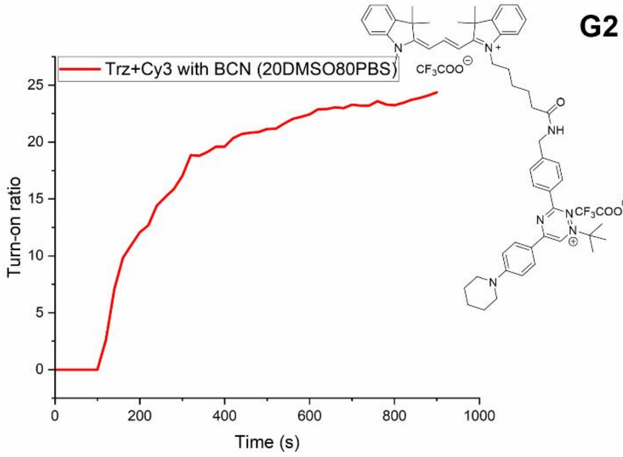

G2

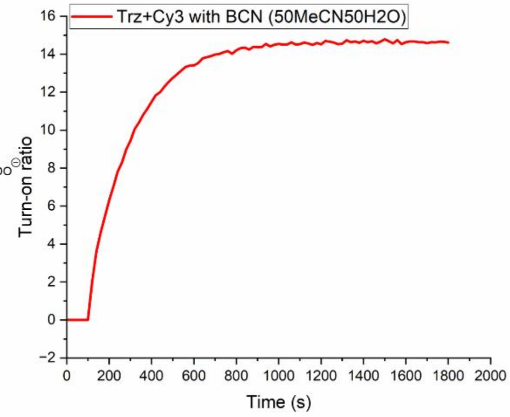

H1

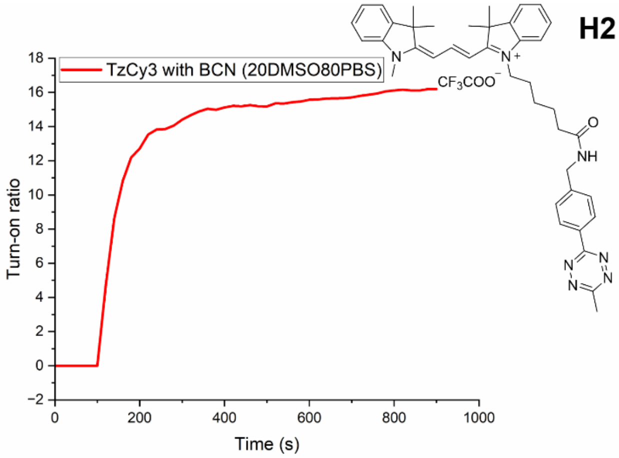

H2

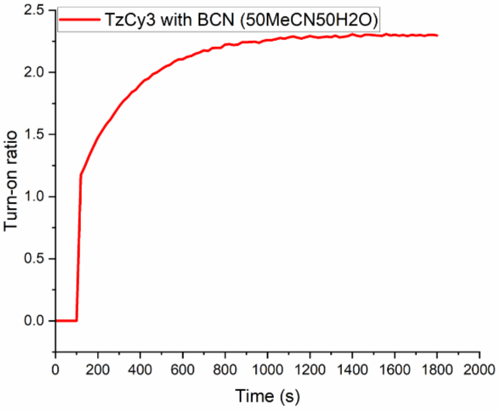

I1

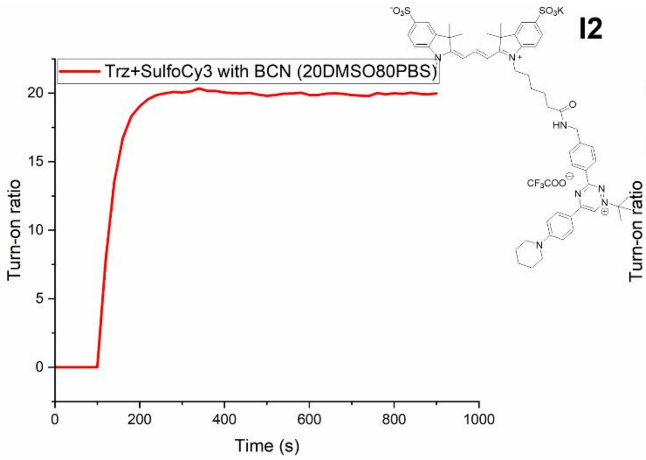

I2

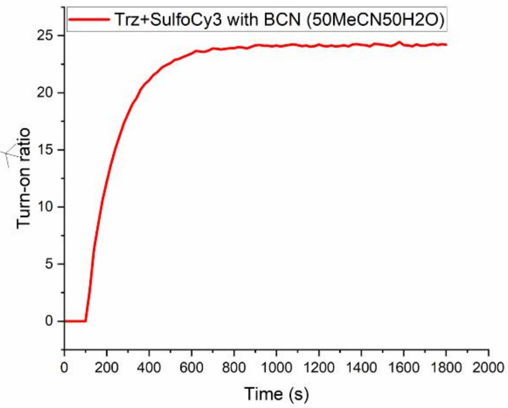

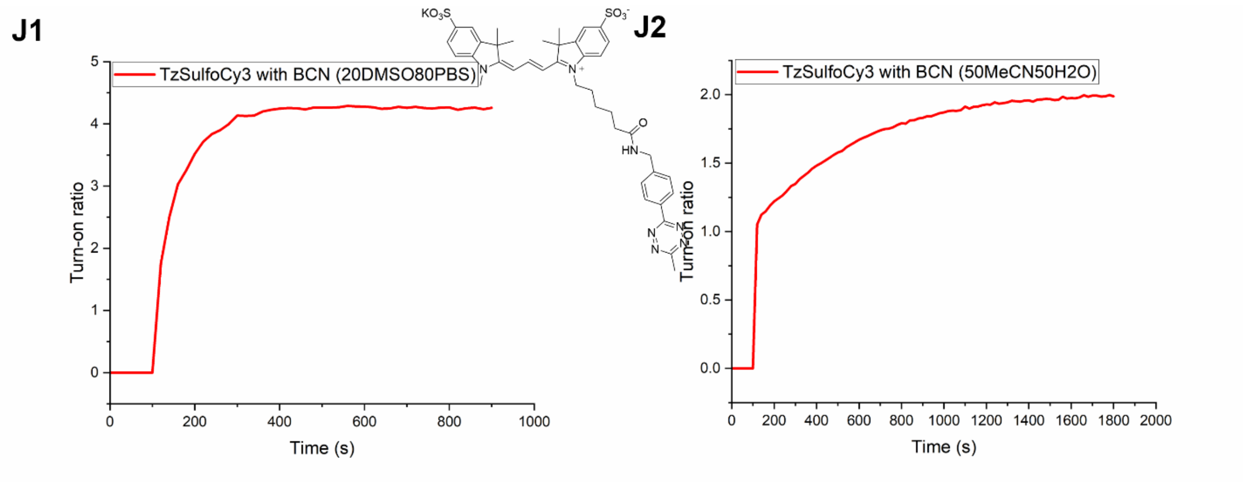
J1
J2
TzSulfoCy3 with BCN (20DMSO80PBS)
TzSulfoCy3 with BCN (50MeCN50H2O)
Turn-on ratio
Time (s)

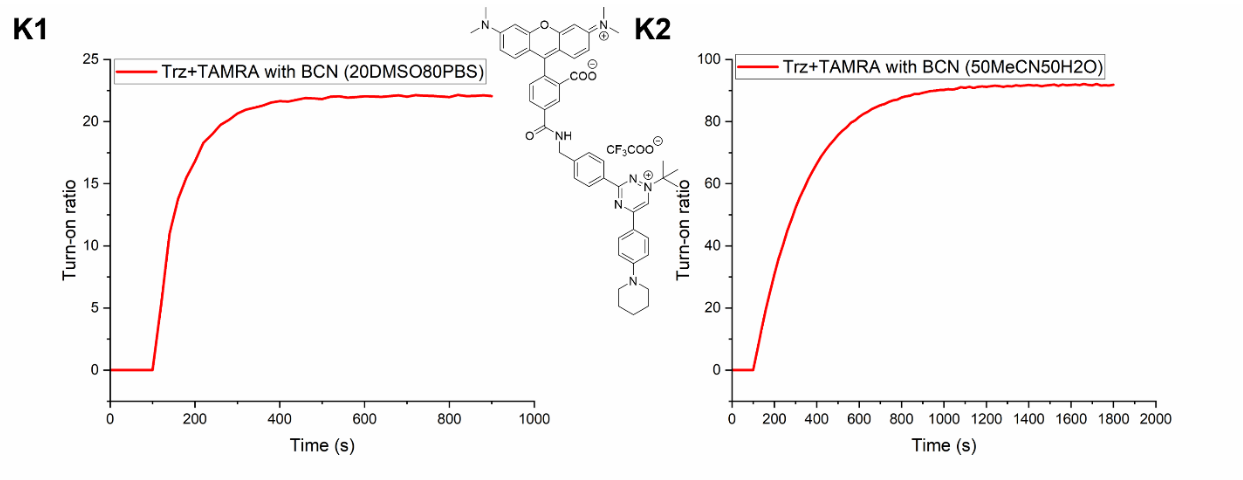
K1
K2
Trz+TAMRA with BCN (20DMSO80PBS)
Trz+TAMRA with BCN (50MeCN50H2O)
Turn-on ratio
Time (s)

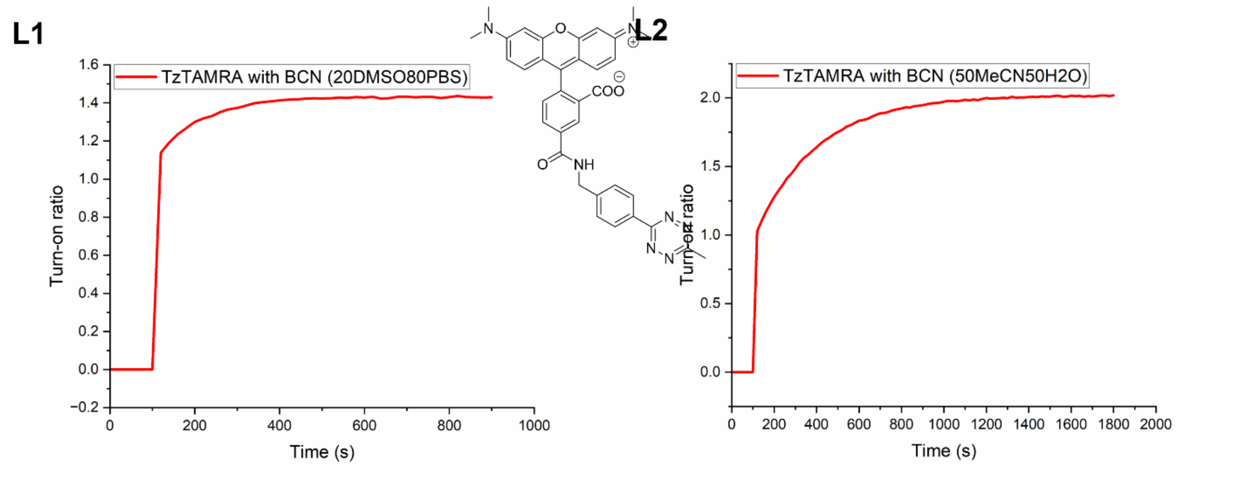
L1
L2
TzTAMRA with BCN (20DMSO80PBS)
TzTAMRA with BCN (50MeCN50H2O)
Turn-on ratio
Time (s)

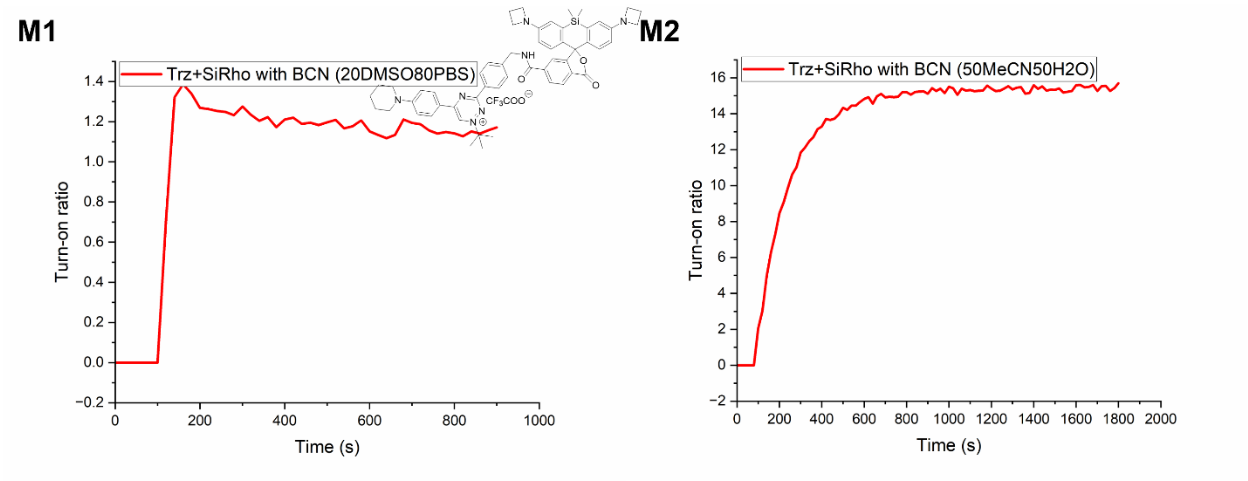

Figure S6: Fluorescence time-lapse measurements showing changes in fluorescence signal for the reaction of fluorogenic T(r)z+ with the ***endo*-BCN** dienophile in time are presented as turn-on values.

Fluorescence quantum yield determination

Quantum yields (QY) were determined using the corresponding commercial fluorophores (carboxylic acids) with known photo-physical properties as the standards (Cy3, BODIPY FL, TAMRA, SiRho, 7-(DiEtAmino)Coum-3-carboxylic acid) available in literature[9] or online (https://www.aatbio.com/catalog/chemical-reagents-classic-dyes). A similar solution described in the preceding "Fluorescence turn-on measurements" chapter was used. All probes were excited, and their fluorescence was collected as indicated in the preceding "Fluorescence turn-on measurements" chapter. The standard 50 nM solutions of the commercial fluorophores were prepared from 1.5 µL of 100 µM fresh stock in DMSO (100%), which was diluted into 3.0 mL of the corresponding solvent system.[10]

Spectroscopy experiments

Methods and experimental setup

Absorption spectra and the molar absorption coefficients were obtained on a UV-vis spectrometer with matched 1.0-cm quartz cells. Molar absorption coefficients were determined from the absorption spectra of a dilution series of four concentrations *c* ~1–10 mM (the average values were obtained from three independent measurements). Fluorescence spectra were recorded on an automated luminescence spectrometer in 1.0 cm quartz fluorescence cuvettes at 25 ± 1 °C; the sample concentration was set to keep the absorbance below 0.1 at λ_{max}; each sample was measured five times, and the spectra were averaged. Emission spectra are normalized and corrected using standard correction files. Spectral overlap was measured by integrating the absorption spectra of the quenchers (*c* = 10 mM) and the normalized emission spectra of the fluorophore. The time correlated single photon counting (TCSPC) with a nanosecond pulse LED source (λ_{em} = 572 nm) was used to determine the fluorescence lifetimes. The data obtained were deconvoluted from the measured decay curves of the sample and the instrumental response function. The quantum yields of

fluorescence were obtained by measuring fluorescence intensity relative to the unsubstituted chromophores with tabulated quantum yields as standards. The photostability of **Trz⁺TAMRA** was performed by irradiating the solution (10 mM in MeCN/water 1/1, *v*/*v*) by a 525-nm 16-LED module for 600 s. No changes in the absorption spectra were detected.

Measured spectra

The conjugate **Trz⁺TAMRA** has absorption spectrum (Figure S7) that is equal to the sum of **Trz⁺1** and **TAMRA** in the region 400-700 nm. Conjugate's emission completely overlaps with **TAMRA** but is much weaker (i.e. quenched by triazinium). **Trz⁺1** has broad absorption in the whole visible range (that is why it is black in color) and weak emission that is close to **TAMRA**'s emission. The triazinium quencher **Trz⁺1** absorbs excitation light and therefore the optical density had to be corrected for internal filter effect. Both compounds therefore have their 0-0 transitions very close to each other. This means that FRET or similar energy transfer between singlet excited states is almost isoenergetic.

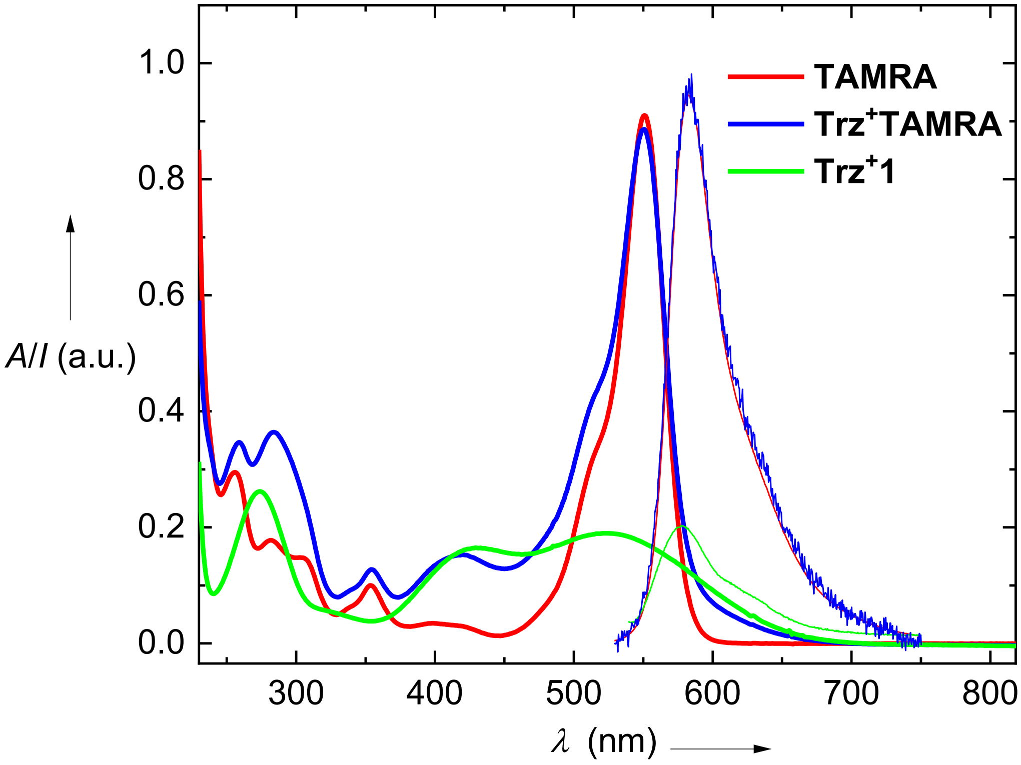

Figure S7: Absorption (thick lines) and normalized emission spectra (thin lines, λ_{exc} = 500 nm) of solutions (*c* = 10 μM) of **TAMRA** (red), **Trz⁺1** (green) and **Trz⁺TAMRA** (blue) in MeCN/water (1:1, *v*/v).

At low concentrations (1 eq. of **Trz⁺1**), the absorption spectrum corresponds to the sum of the absorption spectra of the individual components (Figure S8). At high concentrations (10 eq. of **Trz⁺1**), the absorption of **TAMRA** is suppressed meaning that it forms a complex with **Trz⁺1** that has a lower molar exctinction coefficient by ~50%.

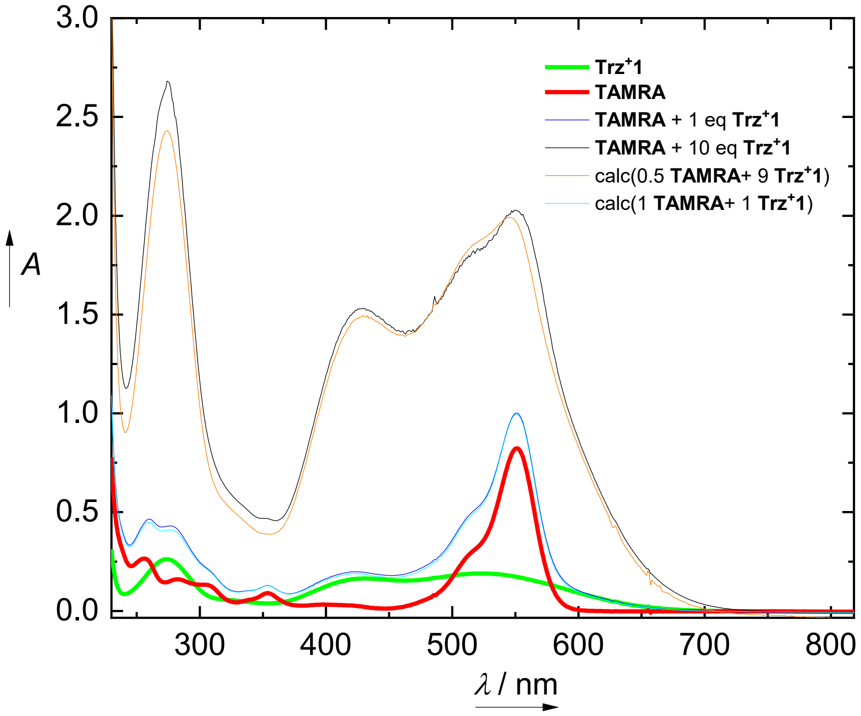

Figure S8: Absorption spectra of solutions (c = 10 µM) of **TAMRA** (red), **Trz⁺1** (green) and their mixtures (equimolar, blue; 10-fold excess of **Trz⁺1**, black) in MeCN/water (1:1, *v*/v). Calculated sum spectra (orange and cyan) are shown for comparison.

The Starn-Volmer analysis (Figure S9) was performed by measuring both emission intensities and lifetimes of **TAMRA** in presence of **Trz⁺1** (λ_{exc} = 500 nm, c(**TAMRA**) = 10 mM in MeCN/water (1:1, *v*/v). Fluorescence intensities show upward curvature in the Stern-Volmer plot that means a combination of dynamic and static quenching. Fluorescence lifetimes of **TAMRA** (Figure S10) are not dramatically influenced by the presence of **Trz⁺1** (but still are partially quenched from 2 ns to 1.9 ns, lifetime of 2 ns corresponds to the literature)[11] which means that static quenching prevails.

Spectral analysis of **Trz⁺TAMRA** showed a combination of static and dynamic quenching with weakly emissive state (τ_{fl} = 0.17 ns) and therefore two emitting species are interconverted between each other (which corresponds to the curvature of the lifetime Stern Volmer analysis. At 1 eq., dynamic quenching prevails, at 10 eq. static quenching prevails. The conjugate (**Trz⁺TAMRA**) fluorescent lifetime has biexponential decay, 85% with short 0.17 ns lifetime (corresponding to the partially quenched state), and the residual 15% with long 1.7 ns lifetime (probably a non-stacked or weakly stacked conformer). The fluorescence of the fluorophore is not quenched completely probably due to incomplete stacking of the quencher to the dye (or existence of equilibrium with a weakly/non-stacked conformer(s)).

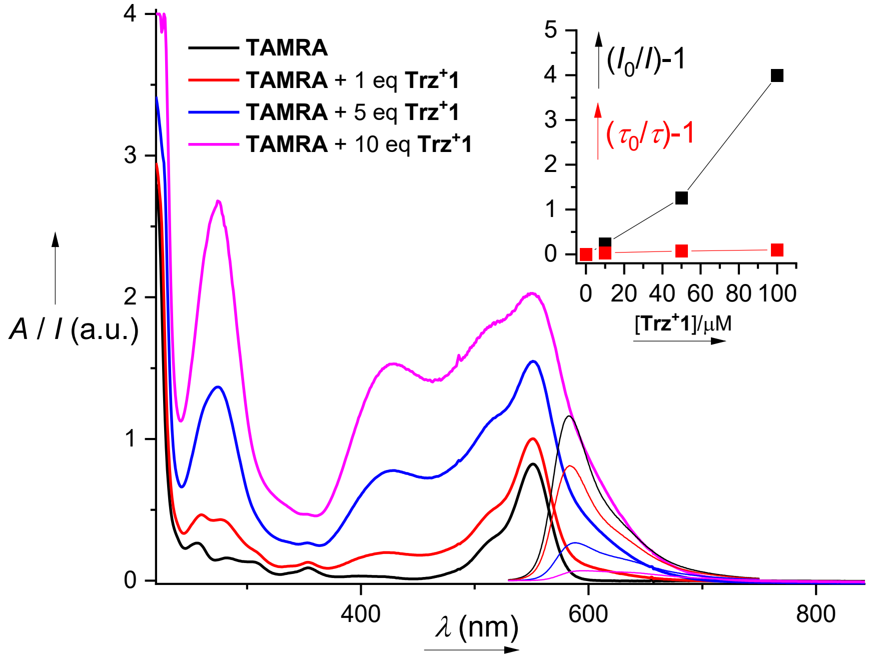

Figure S9: Absorption (thick lines) and normalized emission spectra (thin lines, λ_{exc} = 500 nm) of solutions (c = 10 μM) of **TAMRA** in the absence (black) and in the presence of 1–10 eq. of **Trz⁺1** (red, blue, magenta lines, respectively) in MeCN/water (1:1, v/v). Inset: Stern-Volmer analysis of maximum emission intensity (black dataset) and lifetimes (red dataset).

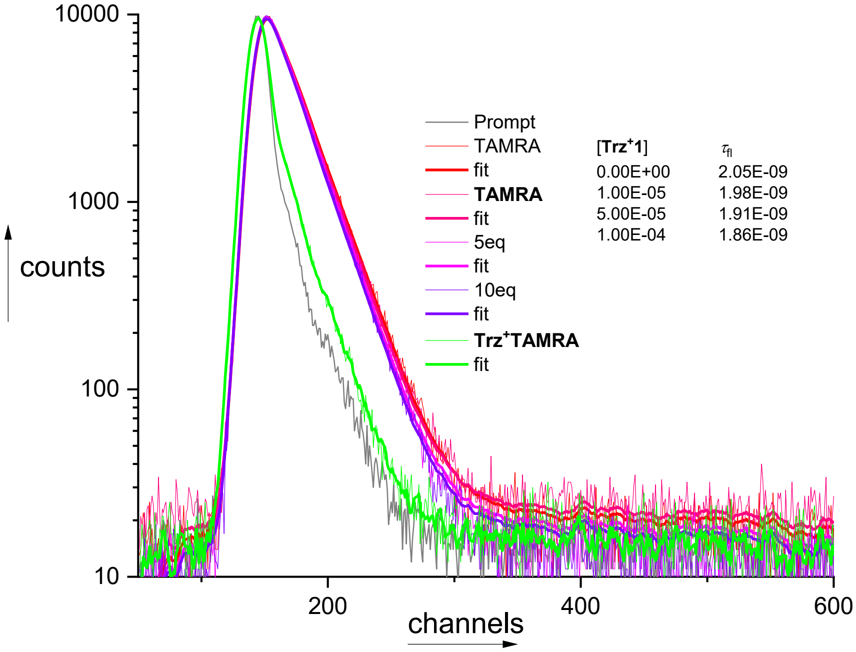

Figure S10: TC SPC decay traces of fluorescence lifetime measurements (thin lines, λ_{exc} = 572 nm) of solutions (c = 10 mM) of **TAMRA** in the absence (red) and in the presence of 1–10 eq. of **Trz⁺1** (pink, magenta, violet lines, respectively) in MeCN/water (1:1, v/v) and of **Trz⁺TAMRA** (c = 10 mM, green) together with the corresponding decay fit (thick lines). 1 channel = 0.11 ns. Inset: table with fitted lifetimes.

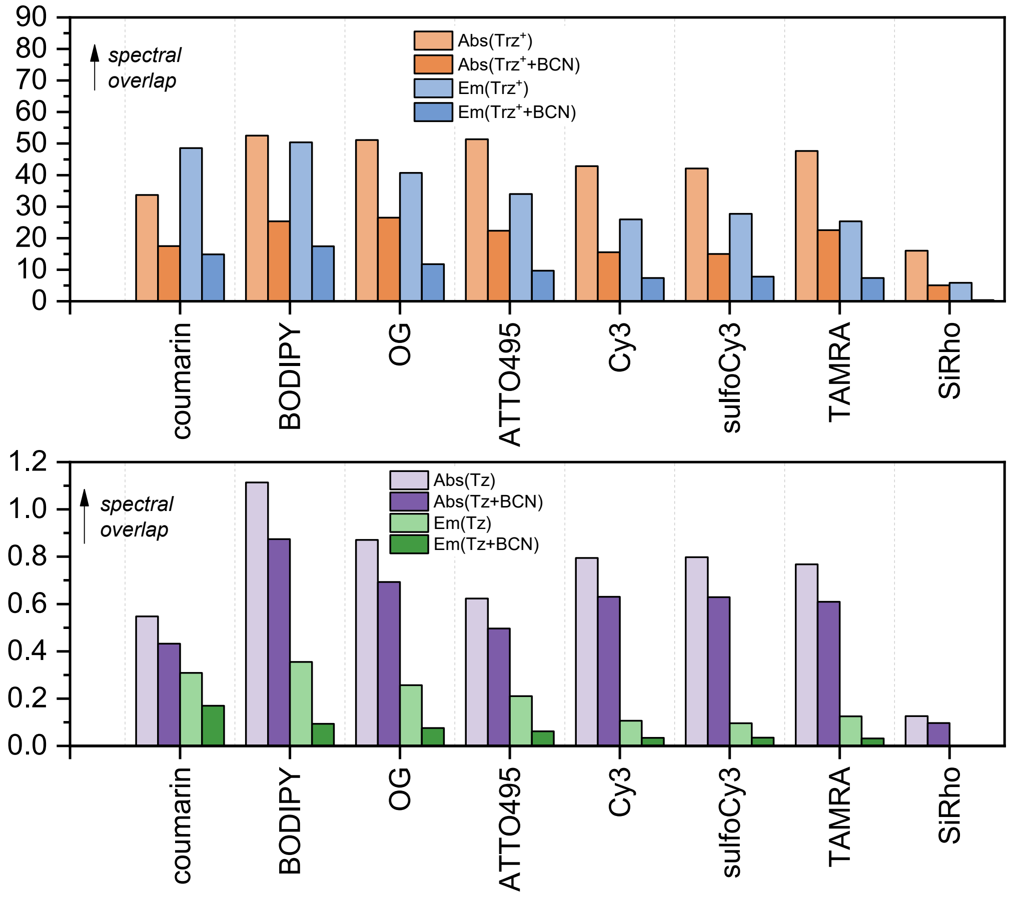

Figure S11: Spectral overlap of studied fluorophores with quenchers (**Trz^{+}1** (top) and **$MeTzNH_2$** (bottom)) in 20% DMSO in PBS (pH = 7.4) before and after IEDDA with BCN. Overlap of absorption spectra of the quencher with absorption spectra (Abs) and emission spectra (Em) of the fluorophore are shown.

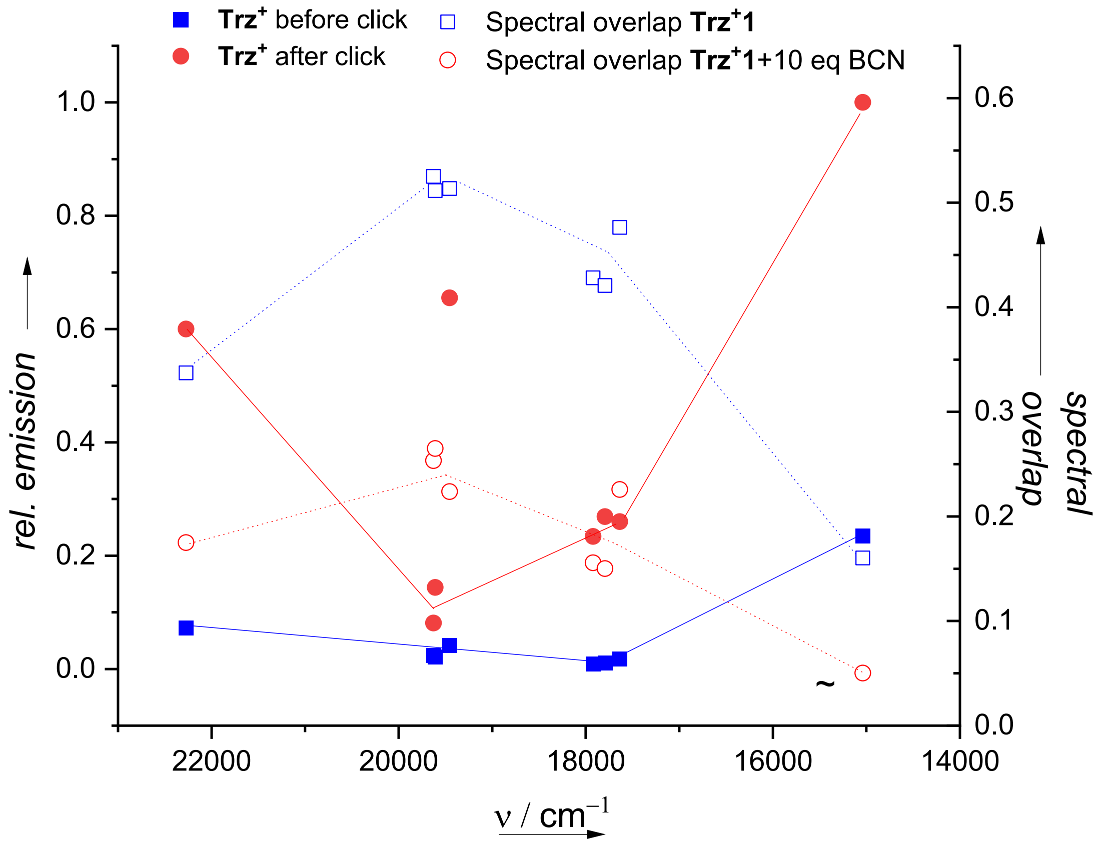

Figure S12: Correlation of normalized fluorescence intensity of Trz^+-substituted derivatives (closed symbols) with spectral overlap of **Trz^+1** with the respective fluorophore (open symbols) before (blue dataset) and after (red dataset) IEDDA with BCN, against the wavenumber of the fluorophore's 0-0 transition.

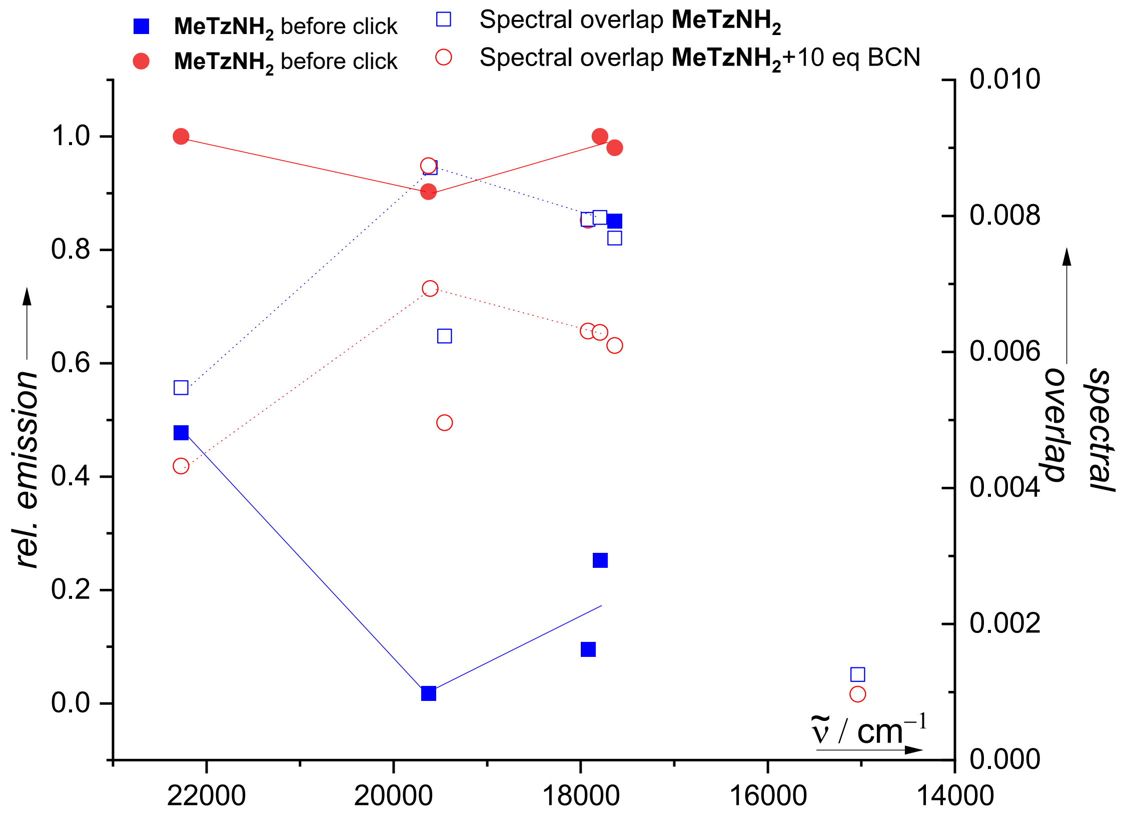

Figure S13: Correlation of normalized fluorescence intensity of Tz-substituted derivatives (closed symbols) with spectral overlap of **MeTzNH₂** with the respective fluorophore (open symbols) before (blue dataset) and after (red dataset) IEDDA with BCN, against the wavenumber of the fluorophore's 0-0 transition.

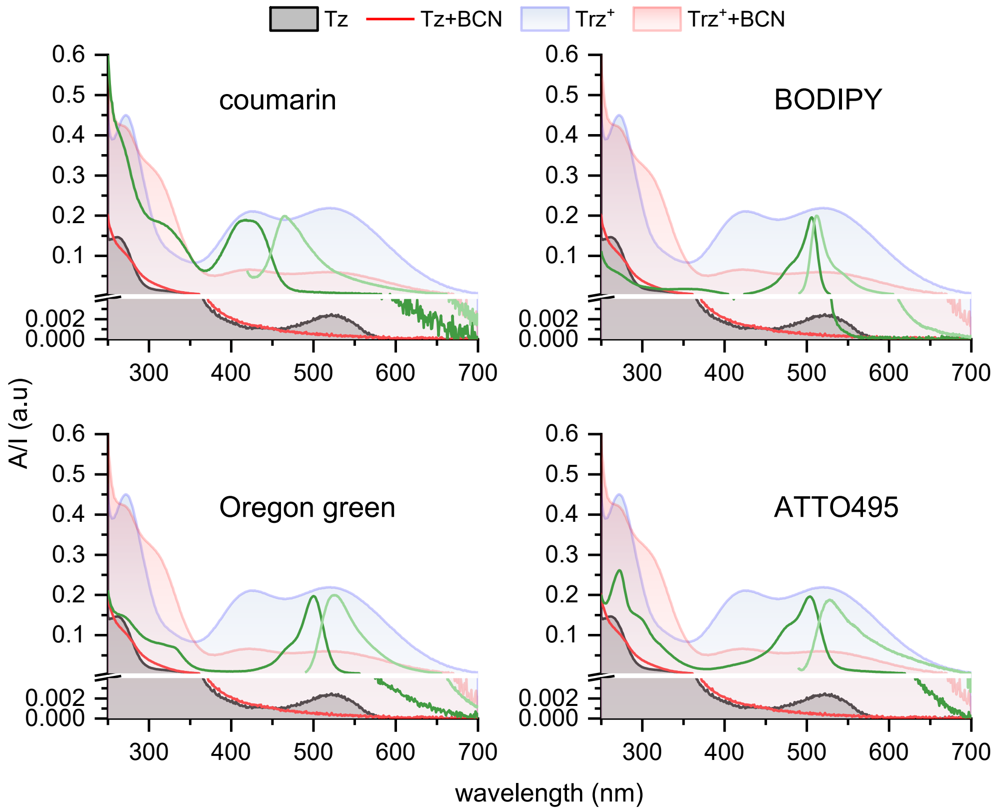

Figure S14: Spectral overlap of **MeTzNH$_2$** (Tz) and **Trz$^+$1** (Trz$^+$) with absorption and emission spectra of studied fluorophores in 20% DMSO in PBS (pH = 7.4), part 1.

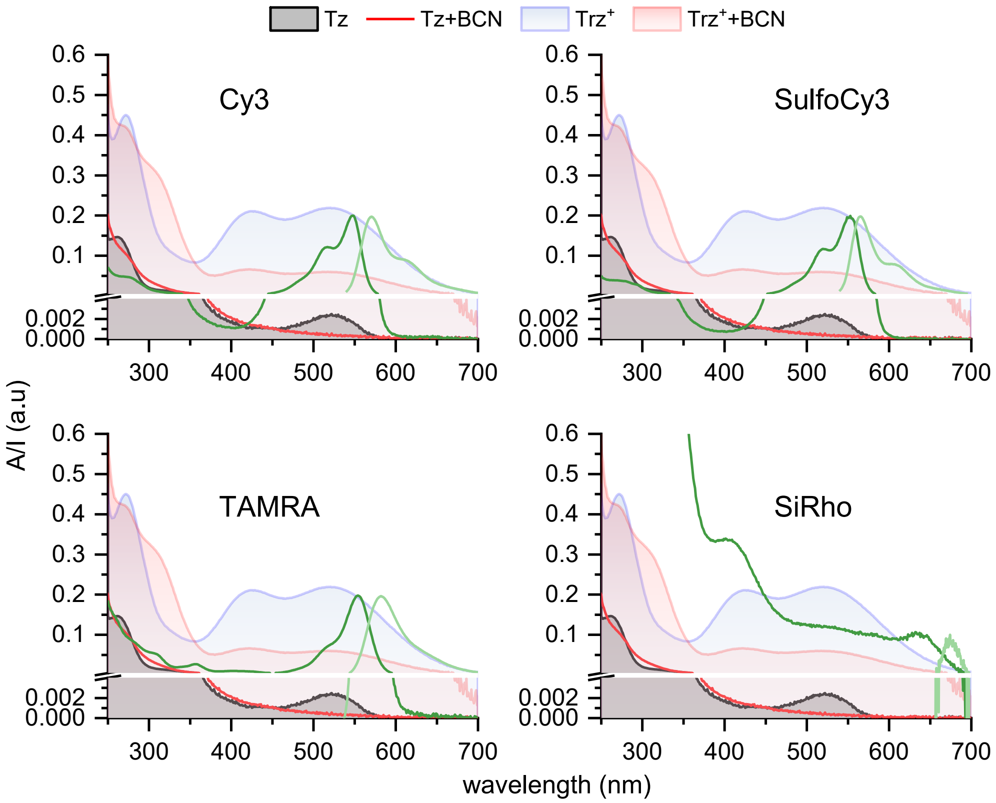

Figure S15: Spectral overlap of **$MeTzNH_2$** (Tz) and **Trz^+1** (Trz^+) with absorption and emission spectra of studied fluorophores in 20% DMSO in PBS (pH = 7.4), part 2.

Computational Details

Conformational sampling and density functional theory calculations

Conformational sampling was performed for all Trz^+/Tz-fluorophore conjugate pairs, separated Trz^+/Tz quenchers and fluorophores, and their corresponding intermolecular complexes. The CREST program (version 2.12)[12] was used with its default conformational search algorithm. Energy and RMSD thresholds were set to 12 kcal/mol (ewin keyword) and 1.0 Å (rthr keyword), respectively. All resulting geometries were optimized at the GFN2-xTB level in water, modeled via the GBSA solvation model.[13] For intermolecular complexes, the non-covalent interaction (NCI) mode of CREST was employed.[12]

Following conformational sampling, density functional theory (DFT) single-point energy calculations were performed using ORCA 5.0.3.[14] These calculations employed the BP86 functional,[15-16] the def2-TZVP basis set,[17] the D3BJ dispersion correction,[18-19] and the conductor-like polarizable continuum model (CPCM)[20-21] for water. The resolution-of-identity approximation (RIJCOSX) was used to accelerate the calculations.[22] Geometries with relative energies below 6 kcal/mol underwent further DFT optimization using the same computational setup as above to identify the lowest-energy structures.

High-level energy calculations for evaluation of interaction energies and reduction potentials were conducted using the PBE0 functional.[23] Electronic absorption spectra were obtained from the excited-state TD-DFT calculations at the PBE0/def2-TZVP level with CPCM solvation. Excited-state analysis was then performed with the TheoDORE package,[24] using default parameters. The transition density matrix was obtained from ORCA output together with molecular orbitals in Molden format. The transition density was decomposed into natural transition orbitals (NTOs), and the molecular complex was divided into two fragments corresponding to fluorophore and quencher units. Electron and hole densities were then projected onto these fragments using Löwdin population analysis and electron-hole correlation plots were generated with the frag_decomp routine.

Gibbs free energy calculations

The terms contributing to Gibbs free energy were calculated as follows:

$$G = E_{el} + G_{solv} + [E_{ZPVE} + RT - RT lnQ], \qquad \text{(Eq1)}$$

where:

i) E_{el} is the *in vacuo* electronic energy, calculated using the methodology described above,

ii) G_{solv} is the free energy of solvation; calculated using the conductor-like polarizable continuum model (CPCM),

iii) $[E_{ZPVE} + RT – RT \ln Q]$ corresponds to the thermal enthalpic and entropic contributions to the solute energy with E_{ZPVE} and Q being the zero-point vibrational energy and the molecular partition function, respectively; obtained from frequency calculations with the rigid rotor/harmonic oscillator approximation (for p = 1 bar, T = 298 K).

Reduction potentials calculations

The standard one-electron reduction potentials ($E°$ in V) were calculated from the change of the Gibbs free energy from Eq1 upon 1e$^-$ reduction of the solute, Ox (aq) + e^- → Red (aq):

$$E° \text{ [V]} = G_{\text{Ox}} \text{ [eV]} - G_{\text{Red}} \text{ [eV]} + \Delta E°_{\text{abs}}(\text{reference}) \text{ [eV]}, \quad \text{(Eq2)}$$

where G_{Ox}/G_{Red} are the Gibbs free energies of the oxidized/reduced state of a solute and $\Delta E°_{abs}$(reference) is the absolute potential of a reference electrode. Potentials were referenced to the saturated calomel electrode (SCE), with the absolute potential of -4.68 eV.[25]

Example ORCA inputs

Geometry Optimization

```
! PBE0 def2-TZVP def2/J RIJCOSX D3BJ Opt Freq CPCM(water)
```

TD-DFT Calculation

```
! PBE0 def2-TZVP def2/J RIJCOSX D3BJ CPCM(water) Normalprint

%tddft

 maxdim 5

 nroots 20

 end
```

Optimized structures

Optimized geometries are provided as XYZ coordinate files in a separate zip archive.

Calculated properties of Trz^+/Tz-fluorophore conjugates

Geometry of the Trz^+/Tz-fluorophore conjugates

Low-energy structures obtained from CREST sampling and subsequent DFT optimizations, as described above, showed a strong preference for stacked configurations in all of the Trz^+-fluorophore conjugates (Figure S16 and Table S3). However, Tz-fluorophore conjugates with OG and TAMRA fluorophores deviated from this trend, favoring open configurations. We attribute this behavior to their short, rigid linkers, which limit the flexibility required for stacking configuration. In case of Tz-fluorophore derivatives, the effect is more pronounced due to their less favorable interaction energies (see below).

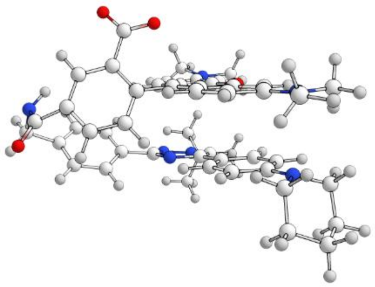

Trz⁺-TAMRA (stacked)

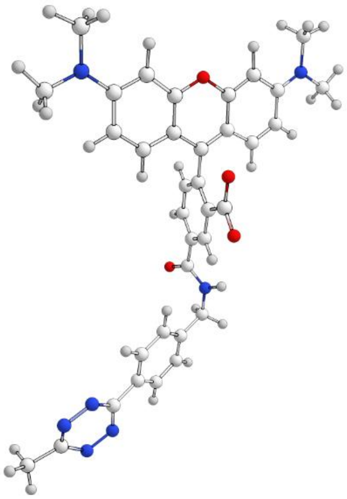

Tz-TAMRA (open)

Figure S16: Comparison of the stacked vs. open configuration of Trz^{+}/Tz-TAMRA conjugates.

Table S3. Computed geometry of the lowest-energy conformer of Trz^{+}/Tz-fluorophore conjugate.

	Trz⁺	*Tz*
ATTO495	Stacked	Stacked
Sulfocy3	Stacked	Stacked
OG	Stacked	Open
BODIPY	Stacked	Stacked
Cy3	Stacked	Stacked
Coum	Stacked	Stacked
TAMRA	Stacked	Open

Interaction energies

To isolate the energetic contribution of the linker, intermolecular complexes were created by truncating Trz^{+}/Tz-fluorophore conjugates into separated fragments and capping the linkers with hydrogen atoms. These complexes underwent NCI-mode CREST sampling and energy evaluation as described above, and interaction energies were computed from the electronic energies of the intermolecular complexes and the separated forms (Figure S17). Calculated interaction energies ranged from ~ –15 to –21 kcal/mol for Trz^{+}-fluorophore complexes and ~ –9 to –14 kcal/mol for Tz-fluorophore complexes (Table S4), averaging to a ~6.5 kcal/mol difference favoring Trz^{+}-fluorophore interactions, likely due to enhanced cation-π interactions in positively charged quencher.

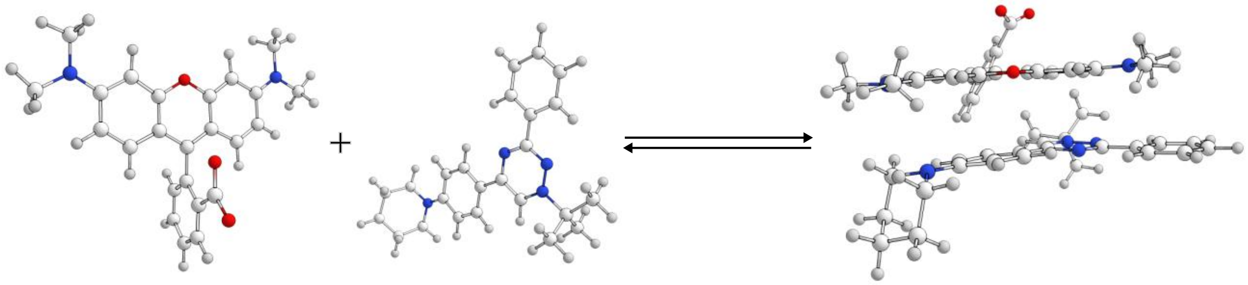

Figure S17: Schematic representation of interaction energy calculations, illustrating the energy difference between separated fragments (fluorophore + quencher) and their corresponding intermolecular complex.

Table S4. Interaction energies of Trz^{+}/Tz + fluorophore intermolecular complexes (kcal/mol).

	Trz⁺	*Tz*
ATTO495	-19.8	-13.2
Sulfocy3	-16.2	-10.2
OG	-19.1	-10.2
BODIPY	-15.5	-9.9
Cy3	-15.8	-11.5
Coum	-15.0	-8.7
TAMRA	-21.4	-13.5

Reduction potentials and vertical excitation energies

Table S5. Computed reduction potentials (V vs. SCE) and vertical excitation wavelengths of individual fluorophore and quenchers.

	$E°$ [V] vs. SCE	*$E°$ [V] vs. SCE[a]	V_{exc} [nm]
Fluorophores			
ATTO495	0.77	-2.20	417
Sulfocy3	0.61	-2.16	447
OG	0.09	-3.12	387
BODIPY	0.81	-2.34	394
Cy3	0.66	-2.18	438
Coum	0.56	-3.02	347
TAMRA	0.41	-2.48	429
SiRho	0.08	-2.50	481
Quenchers			
Tz	-1.12	-	269
Trz+	-0.94	-	493
Tz-BCN	-2.75	-	255
Trz-BCN	-2.92	-	329

[a]Fluorophores excited-state potentials were obtained by shifting the ground-state reduction potentials by vertical excitation energies from TD-DFT calculations, following the Rehm-Weller approximation.[26]

Table S6. Comparison of the reduction potentials and vertical excitation wavelengths for selected fluorophores (FL) and their corresponding Trz^+-conjugates obtained at the DFT/TD-DFT level with PBE0/def2-TZVP method.

	FL $E°$ (V)	***Trz⁺FL*** $E°$ (V)	***FL*** V_{exc} (nm)	***Trz⁺FL*** V_{exc} (nm)
Coum	0.56	0.61	347	349
BODIPY	0.81	0.68	394	390
ATTO495	0.77	0.80	417	419
OG	0.09	0.23	387	382
Cy3	0.66	0.69	438	441
SulfoCy3	0.61	0.63	447	444
TAMRA	0.41	0.51	429	453

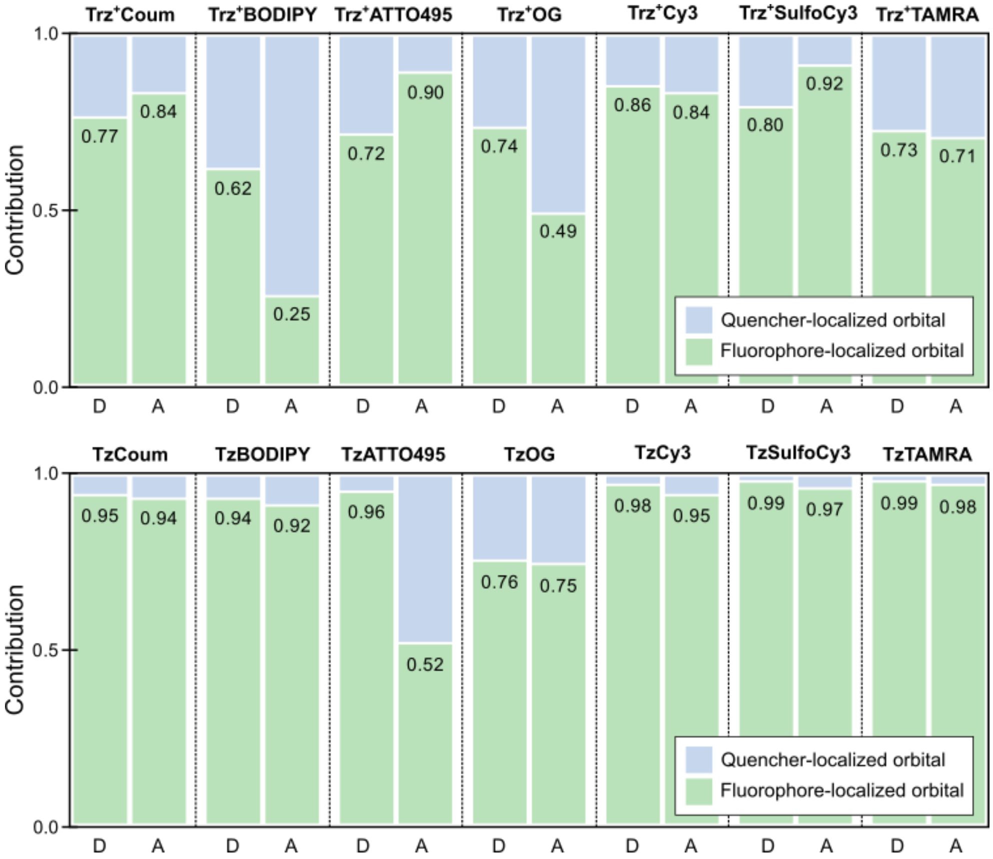

Figure S18: Bar plots showing the relative contributions of the fluorophore (green) and quencher (blue) fragments to the hole (H) and electron (E) populations involved in the main excited-state transition (as listed in Table S6). The increased quencher fraction (blue) in Trz^+ conjugates (top row) illustrates stronger participation of the quencher fragment in both electron and hole densities compared to the Tz conjugates (bottom row). Excited-state analysis was performed with the TheoDORE package, based on electronic absorption spectra obtained at the TD-DFT level with the PBE0/def2-TZVP method.

Quenching mechanism – Photoinduced electron transfer

To evaluate the feasibility of the photoinduced electron transfer quenching mechanism, we computationally assessed its principal thermodynamic factors. The reduction potentials and vertical excitation energies are only minimally affected by the intramolecular stacking interactions between the quencher and fluorophore. This conclusion is supported by the superimposability of the absorption spectrum of the conjugate with the sum spectrum of the individual components. However, the nature of the excited states, specifically the composition of the donor and acceptor orbitals of the fluorophores, shows a significant contribution from the quencher orbitals. Since this mixing is particularly pronounced in the Trz^+-based conjugates, we propose that enhanced electronic coupling may be the contributing factor to their greater quenching efficiency.

Density functional theory (DFT) calculations reveal that the reduction potential of the Trz^+ quencher ($E°_{Trz+}$ = –0.94 V vs. SCE) is approximately 0.2 V less negative than that of the Tz quencher ($E°_{Tz}$ = –1.12 V vs. SCE). Since the computed reduction potentials of all fluorophore dyes are positive ($E°$ = +0.1 to +0.8 V vs. SCE), ground-state electron transfer from the fluorophore to the quencher is thermodynamically prohibited (Table S5). However, based on the experimental E_{0-0} energy and the Rehm-Weller approximation,[26] the excited-state reduction potentials of the fluorophores shift negatively by ~2.0–2.5 V, making the fluorophores' oxidation highly favourable for both Trz^+- and Tz-based quenchers. Importantly, the click reaction products with *endo*-BCN induce a significant negative shift in the reduction potentials of the Trz^+ and Tz cores, resulting in $E°_{Trz+BCN}$ = –2.92 V vs. SCE and $E°_{TzBCN}$ = –2.75 V vs. SCE. Consequently, fluorescence quenching in the click products is unlikely, even in the excited state.

To further examine the PeT mechanism within triazinium conjugates, we tested the photostability of **Trz^+TAMRA** under irradiation (MeCN/water 1/1, 2 h, λ_{irr} = 535 nm). Notably, no detectable photodegradation of **Trz^+TAMRA** was observed, indicating that Trz^+ remains intact and does not decompose during the quenching process.

Calculation of FRET Efficiency

FRET Efficiency was calculated according to the following equation:

$$E(R) = \frac{R_0^6}{R_0^6+R_0} \quad \text{(Eq. 3)}$$

Where R is the distance between the donor fluorophore and the quencher, and R_0 is the Förster radius defined via:[27]

$$R_0^6 = \frac{9\ln(10)\kappa^2\Phi_D^0}{128\pi^5 N_A n^4} J \qquad \text{(Eq. 4)}$$

Where κ^2 is orientation factor ($\kappa^2 = \frac{2}{3}$ for randomly oriented dipoles of donor fluorophore and the quencher), Φ_D^0 is the fluorescence quantum yield of the donor fluorophore, N_A is the Avogadro constant, n is the refractive index of the solvent (n = 1.3478 for MeCN/water 1:1)[28] and J is the spectral overlap integral defined as:

$$J = \int_\lambda \bar{I}_\lambda^{D^*} \varepsilon_A(\lambda)\lambda^4 d\lambda \qquad \text{(Eq. 5)}$$

Where $\bar{I}_\lambda^{D^*}$ is the normalized spectral radiant intensity of the fluorophore donor ($\int_\lambda \bar{I}_\lambda^{D^*} d\lambda = 1$), $\varepsilon_A(\lambda)$ is the absorption spectrum of the quencher entered explicitly by way of the molar absorption coefficient ε_A.

Collecting the numerical constants and length conversion factors, the following practical expression for R_0 can be formulated as:

$$\frac{R_0}{nm} = 0.02108\left(\frac{\kappa^2\Phi_D^0}{n^4}\frac{J}{mol^{-1}dm^3cm^{-1}nm^4}\right)^{\frac{1}{6}} \qquad \text{(Eq. 6)}$$

The overlap integrals have been calculated from Eq. 4 using emission spectra and quantum yields of unsubstituted model fluorophores and absorption spectra of **Trz⁺1** and **$MeTzNH_2$** before and after click reaction with *endo*-BCN (Figure 3A,B). The values of *J*, together with the values of R_0, are summarized in Table S7.

Table S7: Overlap integral *J* and Förster radius R_0 for model fluorophores and **Trz^+1** and **$MeTzH_2$** before and after click reaction with *endo*-BCN in $MeCN/H_2O$ (1:1)

Dye	Φ_D^0	Quencher	*J*/$mol^{-1}dm^3cm^{-1}nm^4$	R_0/nm
**Coum**	0.04	**$MeTzNH_2$**	7.20E+12	**1.31**
		**$MeTzNH_2$+BCN**	3.04E+12	**1.14**
		**Trz^+1**	9.35E+14	**2.95**
		**Trz^+1+BCN**	2.62E+14	**2.39**
**BODIPY**	0.79	**$MeTzNH_2$**	1.30E+13	**2.38**
		**Trz^+1+BCN**	2.62E+12	**1.82**
		**$MeTzNH_2$+BCN**	1.28E+15	**5.12**
		**Trz^+1+BCN**	3.49E+14	**4.12**
**OG**	0.91	**$MeTzNH_2$**	1.12E+13	**2.38**
		**$MeTzNH_2$+BCN**	2.35E+12	**1.83**
		**Trz^+1**	1.31E+15	**5.26**
		**Trz^+1+BCN**	3.55E+14	**4.23**
**ATTO495**	0.2	**$MeTzNH_2$**	8.70E+12	**1.77**
		**$MeTzNH_2$+BCN**	2.06E+12	**1.39**
		**Trz^+1**	1.24E+15	**4.04**
		**Trz^+1+BCN**	3.34E+14	**3.25**
**Cy3**	0.15	**$MeTzNH_2$**	2.36E+12	**1.36**
		**$MeTzNH_2$+BCN**	1.47E+12	**1.26**
		**Trz^+1**	1.13E+15	**3.80**
		**Trz^+1+BCN**	3.05E+14	**3.05**
**SulfoCy3**	0.1	**$MeTzNH_2$**	3.16E+12	**1.33**
		**$MeTzNH_2$+BCN**	1.58E+12	**1.19**
		**Trz^+1**	1.19E+15	**3.58**
		**Trz^+1+BCN**	3.20E+14	**2.88**
**TAMRA**	0.1	**$MeTzNH_2$**	1.26E+12	**1.14**
		**$MeTzNH_2$+BCN**	1.35E+12	**1.16**
		**Trz^+1**	1.09E+15	**3.53**

		**Trz^+1+BCN**	2.94E+14	**2.84**
**SiRho**	0.54	**$MeTzNH_2$**	5.88E+10	**0.91**
		**$MeTzNH_2$+BCN**	2.23E+11	**1.13**
		**Trz^+1**	2.66E+14	**3.69**
		**Trz^+1+BCN**	6.81E+13	**2.94**

The quenching efficiency E depends on the fluorophore quencher distance R and the mutual orientation of the transition dipole moments κ (Eq. 4). Figure xy shows the dependence of E on R for TAMRA-based quencher fluorophore conjugates. While the stacked conformer has almost 100% efficiency of FRET quenching, in the open conformer, the fluorophore and quencher are further apart, which leads to dramatically decreased quenching for tetrazine conjugates, despite the non-zero spectral overlap. This correlates with a very low quenching turn-on value for **TzTAMRA** (Figure 4A, Table 1). In the case of Trz^+, the fluorophore is completely quenched in the stacked conformer before the click reaction (Φ_f (**Trz^+TAMRA**) ~0.002), while some emission (Φ_f (**Trz^+TAMRA**+BCN) ~0.026) is observed after the click with BCN as the conjugate populates the open conformer due to the lack of electrostatic attractive interactions between the fluorophore and the quencher. This model is, however, based on a random orientation of the fluorophore-quencher pair. In reality, the linker limits the system's entropy and thus deviates the orientation factor κ from the value 2/3. A full theoretical analysis of the orientation factor would require simulations of the (excited state) dynamics, which are beyond the scope of this study.

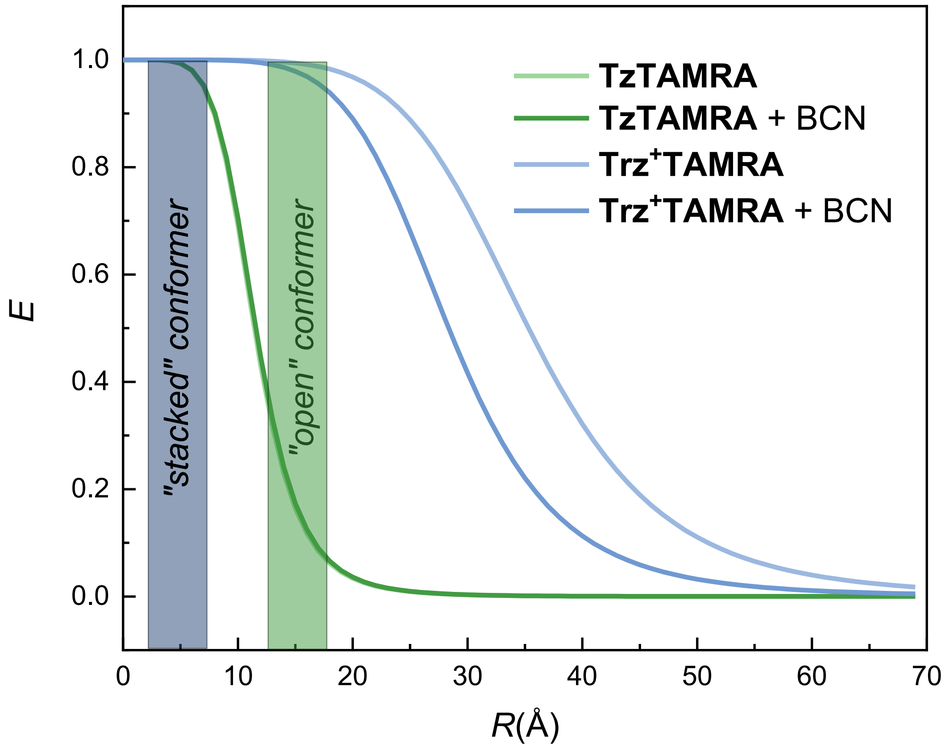

Figure S19: Dependence of FRET quenching efficiency E on fluorophore-quencher distance R for TAMRA-based conjugates in MeCN/H_2O (1:1). Distance ranges for "stacked" and "open" conformers are shown by coloured rectangles.

Reaction kinetics of Trz$^+$1 and Tz1 with *endo*-BCN-OH

Procedure for the kinetic study by UV/VIS: Reaction kinetics was monitored by following the decrease in the concentration (absorbance) of the starting **Trz$^+$1** or **Tz1** in time after the addition of excess dienophile under pseudo-first-order conditions. A typical procedure: a 10 mM solution of the triazinium in (100%) DMSO and 100 mM solution of ***endo*-BCN-OH** in DMSO (100%) was added to MeCN/PBS (1:9) mixture in the cuvette inserted in the spectrophotometer to give a final volume of 3 mL and the measurement was immediately started. The solvent system for **Tz1** contained SDS (1% w/v) to ensure a proper solubility. The final concentration of **Trz$^+$1** or **Tz1** was 10 µM using 10-20 equiv. of ***endo*-BCN-OH**. The decrease in the absorption of triazinium was followed over 20 min in intervals of 0.1, 1.0 or 2.5 min. The time-dependent measurement was performed at the corresponding characteristic absorption wavelengths (λ = 520 nm) of **Trz$^+$1** or (λ = 260 nm) of **Tz1**, which was determined by UV/VIS spectroscopy before each measurement. The measured intensity of the absorption was plotted against time. Fitting the curves with a single exponential equation ($y = y_0 + Ae^{-k/t}$) provided the observed rate constants. The second-order rate constants were obtained by plotting the observed rate constants *vs* concentration of the dienophile and fitting the data with a linear

function, which provided the second-order rate constants from the slope of the plot. All data were processed using OriginPro 9.1 or MS Excel 365 and are presented in Figure S20. °

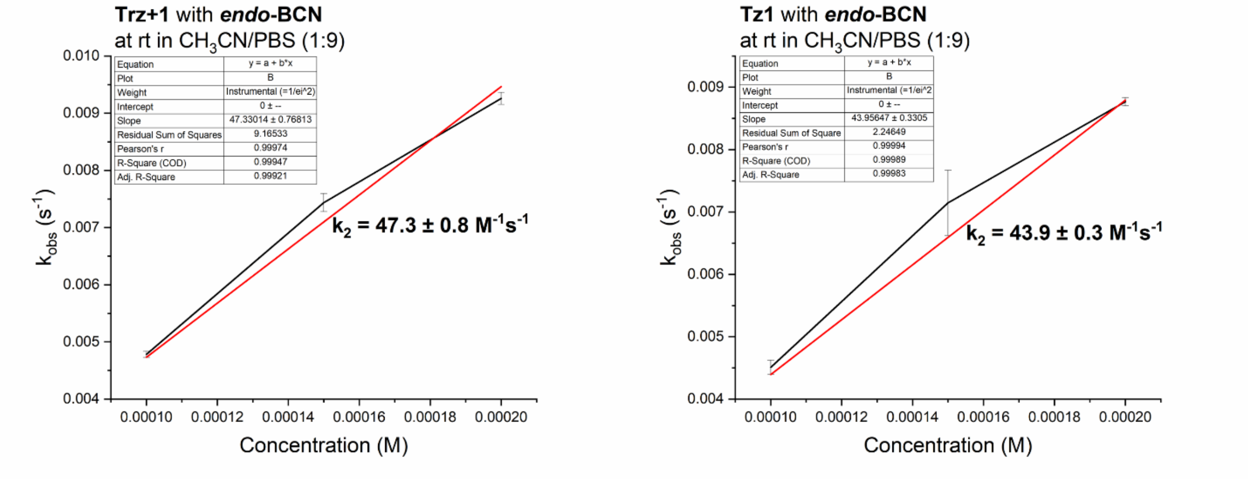

Figure S20: Data from kinetic measurements. Shown are the fitted data, reagents used, conditions and the respective second-order rate constants of **Trz$^+$1** and **Tz1**. Measured at room temperature (22°C).

HPLC-MS analysis of Trz$^+$1 stability under biologically relevant conditions

Stability of **Trz$^+$1** was examined in DPBS by monitoring the solution by HPLC-MS. Typical procedure: 5 µL of a 10 mM solution of **Trz$^+$1** in DMSO (100%) was diluted with 50 µL of DPBS. The final concentration of **Trz$^+$1** was 1 mM. This solution was incubated at 37 °C in a HPLC vial, and samples were analyzed after the indicated time of incubation. The integral of the absorption of **Trz$^+$1** at 525 nm was measured, and the value at the beginning was set to 100% (Table S8 and S9, Figure S23). The measurements were performed in duplicate on CORTECS C18 column (2.7 µm, 50 × 4.6 mm) using solvent A: H_2O + 0.05% HCOOH; solvent B: MeCN + 0.05% HCOOH forming the following gradient 5% B → 95% B (4.0 min), then 95% B (0.5 min), then 95% B → 5% B (0.5 min) and 5% B (2.0 min) at a flow rate of 1.50 mL/min. The samples for HPLC-MS measurements were diluted in a vial at a ratio of 1:1 with MeCN to get 100 µL of a clear solution, and 5 µL of the resulting samples were injected (Figure S21 and S22). The MS device was disconnected from the HPLC during measurements.

In addition, **Trz$^+$1** was incubated in the presence of 10mM reduced glutathione (GSH).

Table S8: Stability of **Trz$^+$1** represented as integrated area of PDA signal at λ = 525 nm from analytical HPLC-MS.

Entry	Additive (equiv.)	Trz$^+$1 (integral)[a] (0 h)	Trz$^+$1 (integral)[a] (12 h)	Trz$^+$1 (integral)[a] (24 h)
**1**	X	5831571	5715920	5677132
**2**	X	5735896	5905320	6184092
**3**	GSH (10)	5632582	5637531	5738116
**4**	GSH (10)	6428892	5600932	5355498

[a]Integrated areas of **Trz$^+$1** from HPLC-MS chromatogram detected at λ = 525 nm.

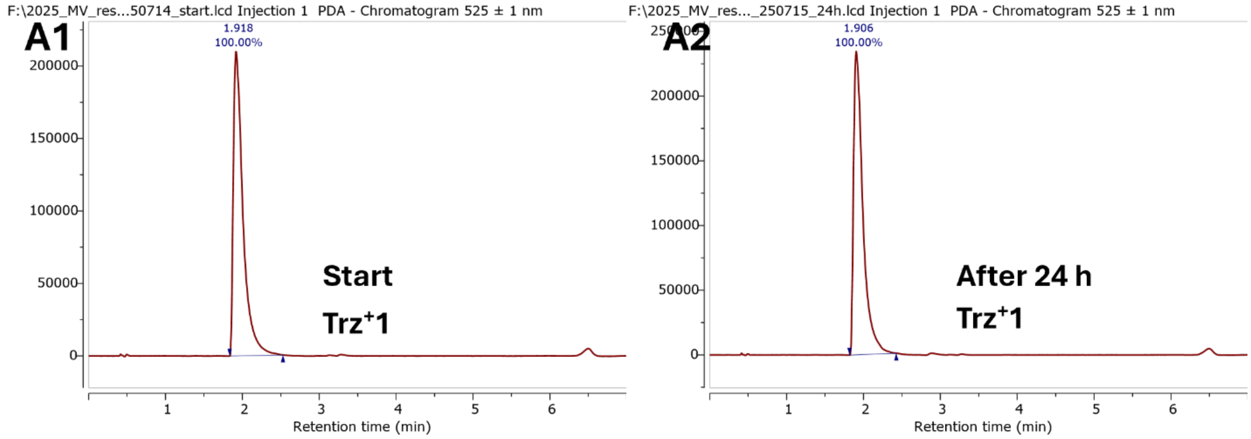

Figure S21: Exported HPLC-MS chromatogram of **Trz$^+$1** before (A1) and after (A2) incubated in PBS (λ = 525 nm).

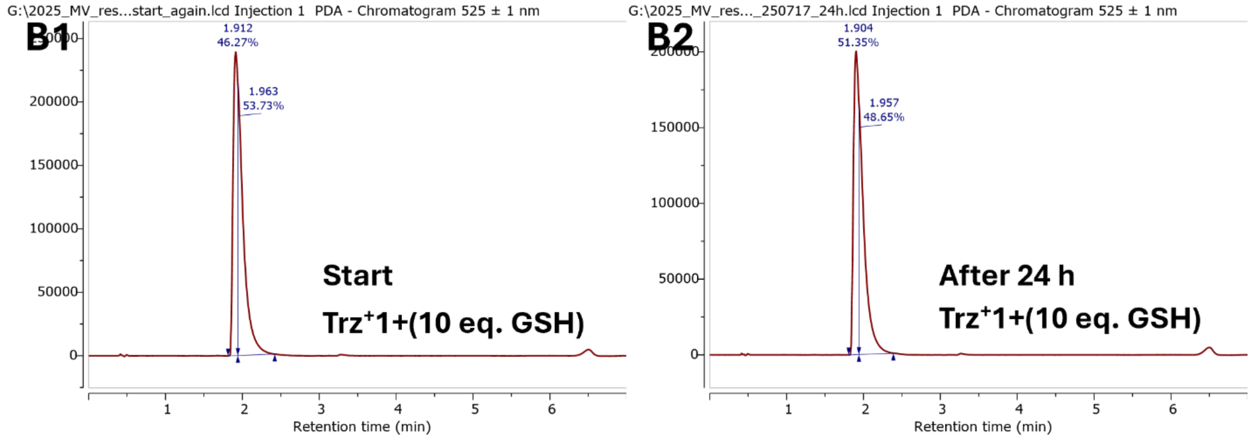

Figure S22: Exported HPLC-MS chromatogram of **Trz$^+$1** before (B1) and after (B2) incubated with 10.0 equiv. of GSH in PBS (λ = 525 nm).

Table S9: Stability of **Trz$^+$1** represented as remaining percentual residue (based on integrated areas from HPLC-MS chromatogram detected at λ = 525 nm).

**Entry**	**Additive (equiv.)**	**Trz$^+$1 (%)[b] (0 h)**	**Trz$^+$1 (%)[b] (12 h)**	**Trz$^+$1 (%)[b] (24 h)**
**1**	X	100	99	99
**2**	X	100	101	106
**3**	GSH (5)	100	88	89
**4**	GSH (5)	100	99	95

[b]Remaining **Trz$^+$1** percentual residue determined by HPLC-MS (starting integral of **Trz$^+$1** absorption (λ = 525 nm) set to 100 %). Calculation based on integrated areas from HPLC-MS chromatogram detected at λ = 525 nm.

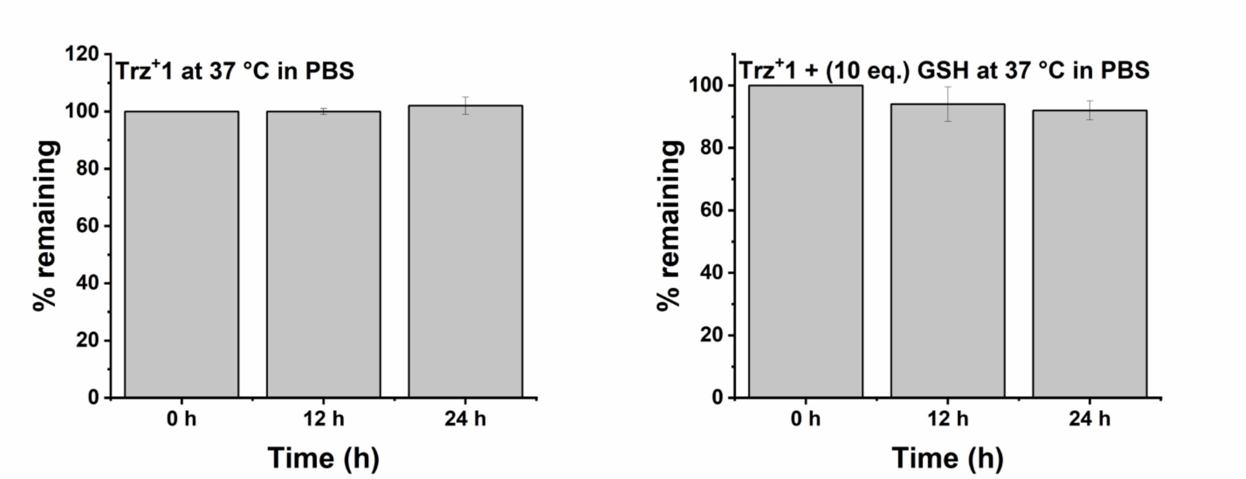

Figure S23: Stability of **Trz⁺1** in PBS (with 10.0 equiv. of GSH) as determined by HPLC-MS after 24 h incubation at 37 °C.

BCN-TPP mitochondrial labeling

U2OS cells were seeded on a 96-well plate at a density of 17500 cells per well in complete DMEM medium. After 48h incubation, the cells were incubated with 5 μM **BCN-TPP** complete DMEM medium for 15 min.[29] The cells were 3 times washed with a complete L-15 medium. After that, 1 μM of each cell-permeable **T(r)z$^{(+)}$Fl** was added to the cells in a complete L-15 medium with DRAQ5 (5 μg/mL) or Hoechst 33342 (1 μg/mL) for 30 min. Then the cells were imaged on a confocal microscope without any washing (Figure S24).

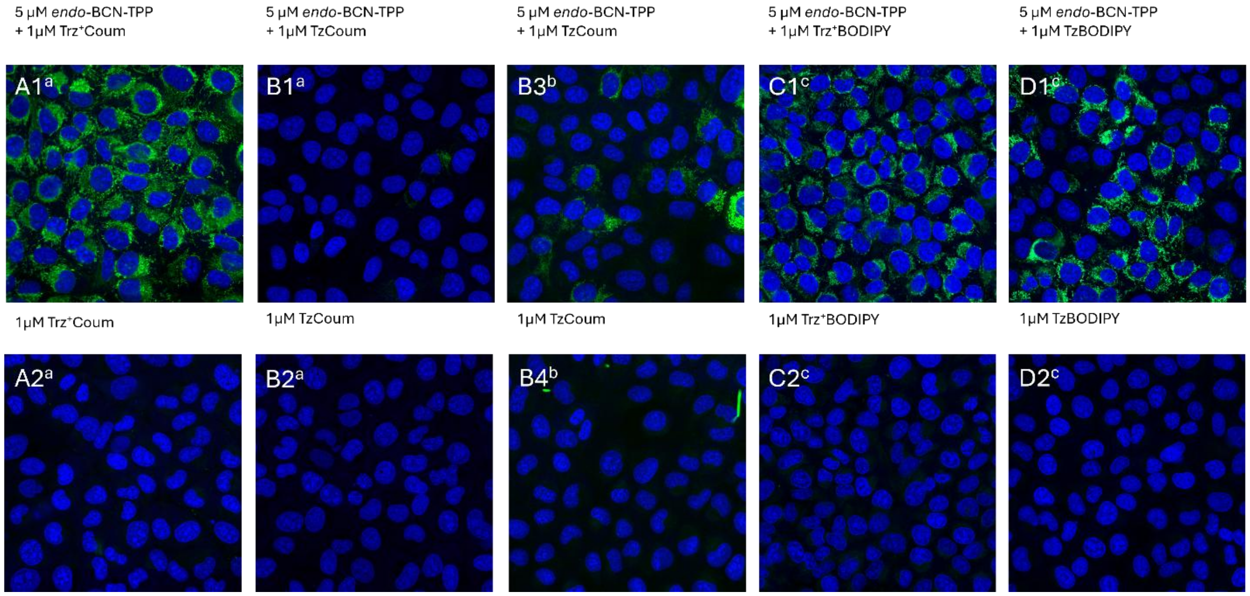

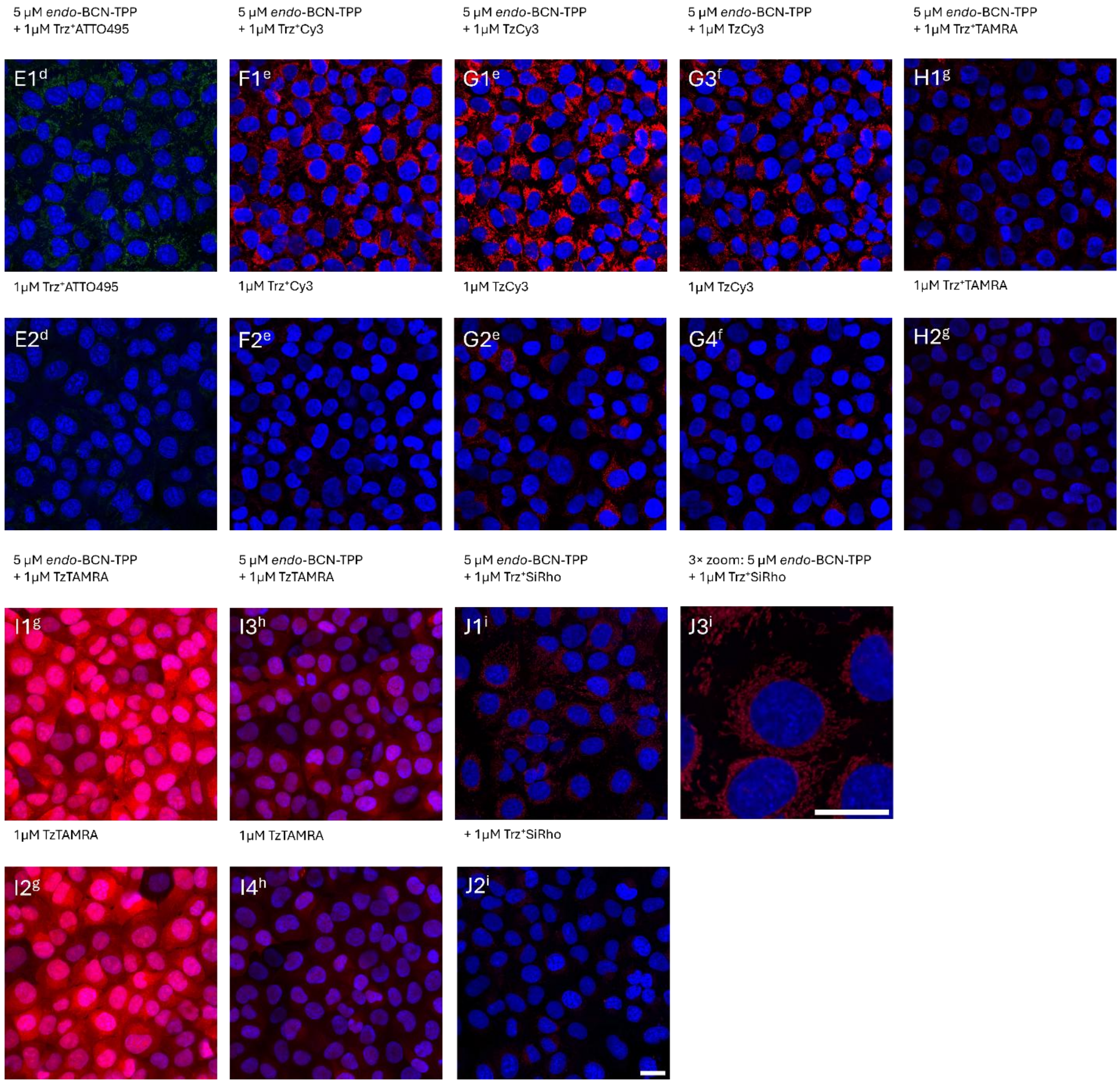

Figure S24: Confocal microscope images of U2OS cells treated with ***endo*-BCN-TPP** conjugate and labeled with A1) **Trz⁺Coum**, B1) B3) **TzCoum**, C1) **Trz⁺BODIPY**, D1) **TzBODIPY**, E1) **Trz⁺ATTO495**, F1) **Trz⁺Cy3**, G1) G3) **TzCy3**, H1) **Trz⁺TAMRA**, I1) I3) **TzTAMRA,** J1) J3) **Trz⁺SiRho** (3×zoomed). A2) B2) B4) C2) D2) E2) F2) G2) G4) H2) I2) I4) J2) are the corresponding controls (cells treated only with the probe). Click labeling is shown in red or green. Nuclei were stained with Hoechst 33342 or DRAQ5 and are shown in blue. [a,b]Microscope set-up: Coum (405 nm) laser intensity: 2.0[a] or 3.0[b] %, and emission (410 – 676 nm) detector: 600[a] or 650[b] V. [c]Microscope set-up: BODIPY (488 nm) laser intensity: 2.0 %, and emission (490 – 658 nm) detector: 600 V. [d]Microscope set-up: ATTO495 (488 nm) laser intensity: 3.0 %, and emission (490 – 667 nm) detector: 700 V. [e,f]Microscope set-up: Cy3 (561 nm) laser intensity: 1.0[e,f] %, and emission (410 – 694 nm) detector: 600[e] or 550[f] V. [g,h]Microscope set-up: TAMRA (561 nm) laser intensity: 1.0[g,h] %, and emission (410 – 694 nm) detector: 600[g] or 550[h] V. [i]Microscope set-up: Cy5 (639 nm) laser intensity: 3.5 %, and emission (641 – 694 nm) detector: 700 V. Hoechst 33342 – laser 405 nm. DRAQ5 – laser 639 nm. Scale bar is 25 µm.

Then the cells were briefly washed 3 times with 100 µL of PBS, detached from the dish using 50 µL of accutase. The wells were briefly washed 2 times with 50 µL of PBS to collect the cells for flow cytometry analysis (Figure S25).

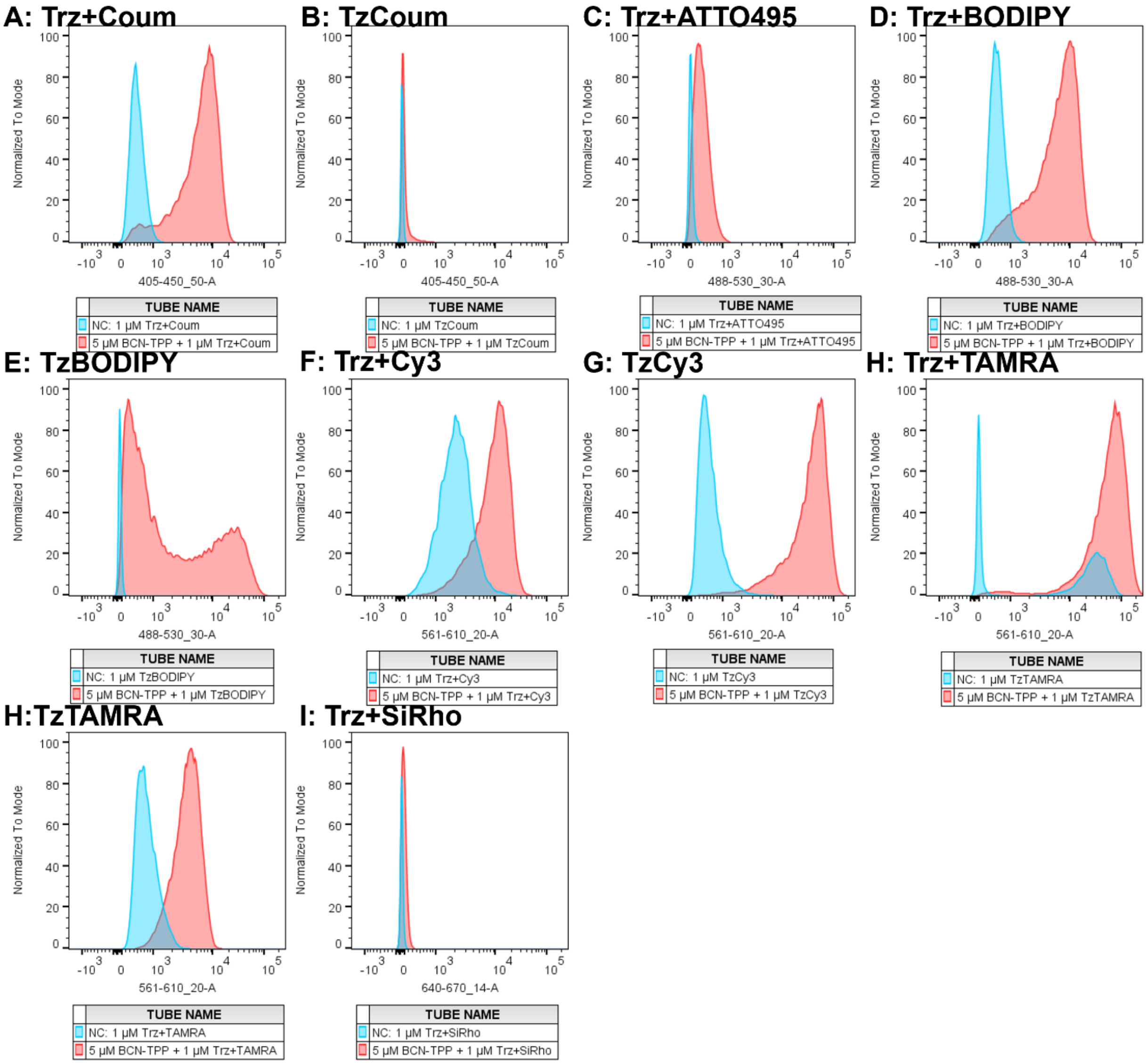

Figure S25: Corresponding histograms from flow cytometry analysis of U2OS cells treated with ***endo*-BCN-TPP** conjugate and labeled with **T(r)z+Fluorophor** are shown in red. The corresponding negative controls (cells treated only with the probe) are in blue.

Fluorescence intensity from BCN-TPP mitochondrial labeling

Fluorescence intensity was quantified using the Fiji distribution of ImageJ. Multi-channel images were first split into individual fluorescence channels using Image > Color > Split Channels. Only the relevant resulting grayscale image (with mitochondrial labeling) was processed independently.

Quantification was performed by automatically defining the whole image as a region of interest (ROI). For each ROI, the Analyze > Measure function was applied to obtain the mean gray value and integrated density (area × mean gray value). All analysis steps were performed using identical

acquisition and processing parameters to maintain comparability between conditions (Tables S10, S13 and S14).

Table S10: Quantified fluorescence (ImageJ) from BCN-TPP mitochondrial labeling

Value	Mean gray value		Turn-on	Integrated density		Turn-on
**$T(r)z^{(+)}Fl$**	**$T(r)z^{(+)}$**	**$T(r)z^{(+)}$+BCN**	**-**	**$T(r)z^{(+)}$**	**$T(r)z^{(+)}$+BCN**	**-**
**Trz^{+}Coum**	2.6	28.8	11	120	1344	11
**TzCoum**	0.2	0.7	3.5	11	34	3.1
**Trz^{+}BODIPY**	4.4	20.1	4.6	204	936	4.6
**TzBODIPY**	1.6	18.2	11	73	849	12
**Trz^{+}ATTO**	0.2	7.4	37	8	344	43
**Trz^{+}Cy3**	4.2	21.0	5	313	1547	5
**TzCy3**	9.4	38.0	4	437	1770	4
**Trz^{+}TAMRA**	14.4	15.7	1.1	672	730	1.1
**TzTAMRA**	153.8	180.4	1.2	7168	8409	1.2
**Trz^{+}SiRho**	1.8	4.8	2.7	131	225	1.7

Additional BCN-TPP mitochondrial labeling

U2OS cells were seeded on a 96-well plate at a density of 25000 cells per well in a complete DMEM medium. After 24h incubation, the cells were incubated with 5 µM of **BCN-TPP**[27] in a complete L-15 medium for 15 min. The cells were washed 3 times with a complete L-15 medium. After that, 1 µM of **$T(r)z^{+}$Coum** was added to the cells in a complete L-15 medium with DRAQ5 (5 µg/mL) for 30 min. Then the cells were imaged on a confocal microscope (Figure S26).

Alternatively, 5 µM of **BCN-TPP** was incubated with 1 µM of **$T(r)z^{+}$Coum** in a complete L-15 medium for 45 min. After that, the resulting click product was added to the cells in a complete L-15 medium with DRAQ5 (5 µg/mL) for 30 min. Then the cells were imaged on a confocal microscope (Figure S26).

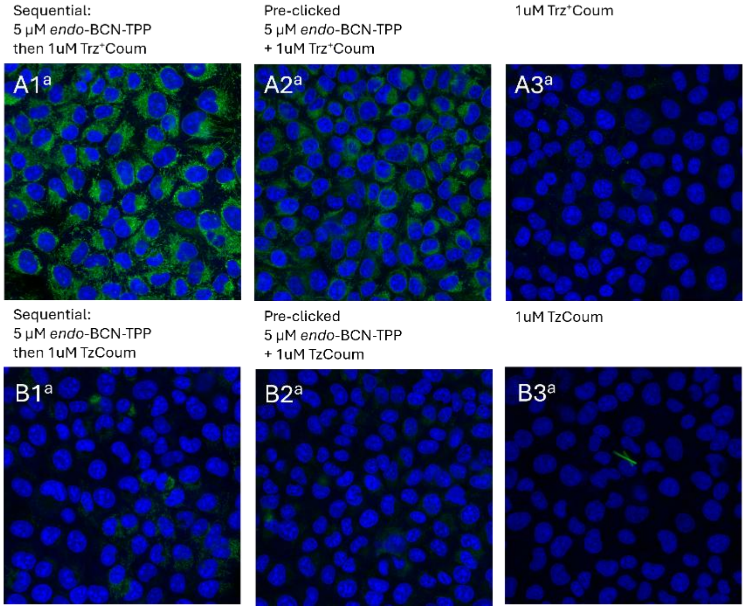

Figure S26: Confocal microscope images of U2OS cells treated with ***endo*-BCN-TPP** conjugate and labeled with A1) **Trz^{+}Coum**, B1) **TzCoum** or A2) B2) pre-clicked ***endo*-BCN-TPP+T(r)z$^{(+)}$Coum** conjugate. A3) B3) are the corresponding controls (cells treated only with the probe). Click labeling is shown in green. Nuclei were stained with DRAQ5 and are shown in blue. [a]Microscope set-up: Coum (405 nm) laser intensity: 2.0 %, and emission (410 – 676 nm) detector: 600 V.

SKBR3 cells Herceptin labeling

Rebridging of Herceptin with BrPD-PEG3-BCN **(Herceptin-BrPD4)**

35 µL of 15 mg/mL solution of Therapeutic Herceptin monoclonal antibody was diluted into buffer containing: 25 mM NaCl, 50 mM HEPES-NaOH (pH 8.3), 0.5 mM EDTA, (2%) DMSO, to the 5 mg/mL final concentration. It was buffer exchanged by (0.5 mL) Zeba (Thermo®) spin column into the indicated 50 mM HEPES-NaOH (pH 8.3) buffer. TCEP.HCl (3.6 µL,10 mM in deionized H_2O, 10.0 equiv.) was added to the Herceptin solution and the mixture was incubated at rt for 90 min. After this time, the solution was buffer exchanged by (0.5 mL) Zeba (Thermo®) spin column into the indicated 50 mM HEPES-NaOH (pH 8.3) buffer. **BrPD4** (7.1 µL,10 mM in DMSO, 20. 0 equiv.) was added to the solution of reduced antibody. The reaction was incubated at rt for 15 h. Excess reagent was removed by desalting using (0.5 mL) Zeba (Thermo®) spin column into PBS (pH = 7.4).

Modification of Herceptin with NHS-PEG3-BCN

35 µL of 15 mg/mL solution of Therapeutic Herceptin monoclonal antibody was diluted into buffer containing: 25 mM NaCl, 50 mM HEPES-NaOH (pH 8.3), 0.5 mM EDTA, (2%) DMSO, to the 5 mg/mL final concentration. It was buffer exchanged by (0.5 mL) Zeba (Thermo®) spin column into the indicated 50 mM HEPES-NaOH (pH 8.3) buffer. Commercial (freshly dissolved) ***endo*-BCN PEG3 NHS** (0.52 µL,10 mM in DMSO, 1.5 equiv.) was added to the solution of antibody. The reaction was incubated at rt for 1 h. After this time, the second portion of ***endo*-BCN PEG3 NHS** (0.52 µL,10 mM in DMSO, 1.5 equiv.) was added to the mixture and the incubation continued for 1 additional hour.

Excess reagent was removed by desalting using (0.5 mL) Zeba (Thermo®) spin column into PBS (pH = 7.4).

SDS-PAGE analysis of Herceptin modification

Non-reducing glycine-SDS-PAGE at 4-15% precast acrylamide gel (BIO RAD: cat. #456-1085) was performed following standard lab procedures. A broad-range MW marker Precision Plus Protein™ Kaleidoscope™ Standards (10-250 kDa, BIO RAD: cat #1610375) was co-run to estimate protein weights. Click reaction was firstly performed on 10 µL of diluted mAb (final ~17 µM concentration in PBS), which was treated by **T(r)z$^{(+)}$Sulfo Cy3** reagent (1.75 µL of 1 mM in DMSO (100%); 20 equiv.) for 1 h at rt. The resulting samples (2.0 µL at ~17 µM in total mAb) were mixed with 2.5 µL of 4 × Laemmli SDS sample loading buffer (BIO RAD: cat. #1610747) and diluted with H_2O up to 10 µL. Dithiotreitol (DTT) was used as a reducing agent in reduced samples (1 µL of 1 M; excess). The gel was run at constant voltage (300 V) for 20 min in 1× Tris/Glycine/SDS running buffer. The resulting gel was imaged using Typhoon™ scanner using Cy3 channel (λ_{ex} = 532 nm) and then stained by the Coomassie G-250 dye (BIO RAD: cat. #1610786) following a standard manufacturer's protocol (Figure S27).

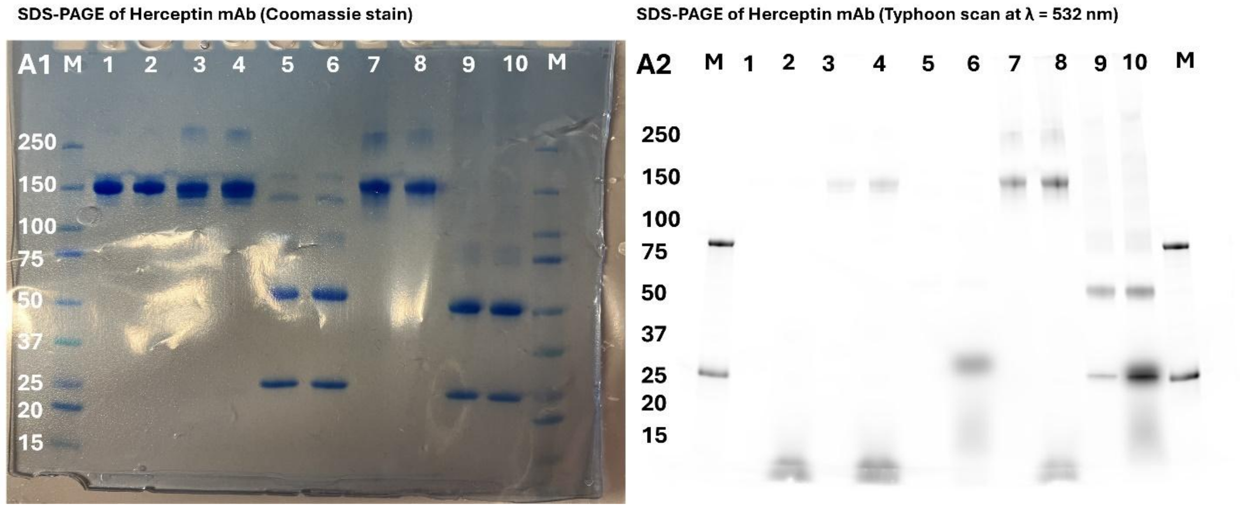

Figure S27: SDS-PAGE analysis of Herceptin mAb 1) 2) before and after 3) 4) 5) 6) rebridging or 7) 8) 9) 10) NHS conjugation with *endo*-BCN-linker and click reaction with T(r)z$^{(+)}$SulfoCy3 dyes. M) protein ladder. 1) Herceptin + Trz^{+}SulfoCy3. 2) Herceptin + TzSulfoCy3. 3) Herceptin-BrPD4 + Trz^{+}SulfoCy3. 4) Herceptin-BrPD4 + TzSulfoCy3. 5) Reduced Herceptin-PD4 + Trz^{+}SulfoCy3. 6) Reduced Herceptin-PD4 + TzSulfoCy3. 7) Herceptin-PEG3-BCN + Trz^{+}SulfoCy3. 8) Herceptin-PEG3-BCN + TzSulfoCy3. 9) Reduced Herceptin-PEG3-BCN + Trz^{+}SulfoCy3. 10) Reduced Herceptin-PEG3-BCN + TzSulfoCy3.

Table S11: Summary of SDS-PAGE bands quantification (integrated areas) by densitometry from modified Herceptin mAb under Coomassie or fluorescent (λ = 532 nm) visualization

Coomassie SDS-PAGE				Typhoon fluorescent scan (at λ = 532 nm)			
Entry	Native mAb	Reduced mAb LC	Reduced mAb HC	Entry	Native mAb	Reduced mAb LC	Reduced mAb HC
Well 1	6541.326	-	-	Well 1	0	-	-
Well 2	7015.861	-	-	Well 2	0	-	-
Well 3	8948.569	-	-	Well 3	5213.276	-	-
Well 4	7814.983	-	-	Well 4	10554.309	-	-
Well 5	-	3464.234	5092.79	Well 5	-	0	0
Well 6	-	2969.598	5248.255	Well 6	-	11393.56	0
Well 7	7509.154	-	-	Well 7	30737.525		-
Well 8	7722.740	-	-	Well 8	48929.538		-
Well 9	-	3590.891	5702.619	Well 9	-	3973.418	30666.484
Well 10	-	3447.477	5917.033	Well 10	-	42178.337	21283.099

Table S12: Summary of SDS-PAGE bands quantification (normalized values) by densitometry from modified Herceptin mAb under Coomassie or fluorescent (λ = 532 nm) visualization

Coomassie SDS-PAGE[a]				Typhoon fluorescent scan (at λ = 532 nm)[a]			
Entry	Native mAb	Reduced mAb LC	Reduced mAb HC	Entry	Native mAb	Reduced mAb LC	Reduced mAb HC
Well 1	69 %	-	-	Well 1	0	-	-
Well 2	74 %	-	-	Well 2	0	-	-
Well 3	95 %	-	-	Well 3	8 %	-	-
Well 4	83 %	-	-	Well 4	16 %	-	-
Well 5	-	36 %	54 %	Well 5	-	0	0
Well 6	-	31 %	56 %	Well 6	-	17 %	0
Well 7	80 %	-	-	Well 7	48 %		-
Well 8	82 %	-	-	Well 8	77 %		-
Well 9	-	38 %	60 %	Well 9	-	6 %	48 %
Well 10	-	36 %	63 %	Well 10	-	66 %	33 %

[a]Values of integrated areas normalized to the highest signal (from Well 10) set as 100 %.

Note: Herceptin-BrPD4 conjugate was not further used due to instability of the solid BrPD4 and the conjugate. Therefore, all the following experiments were performed only with Herceptin-PEG3-BCN.

Intact mass analysis of mAb Herpectin-PEG3-BCN conjugate

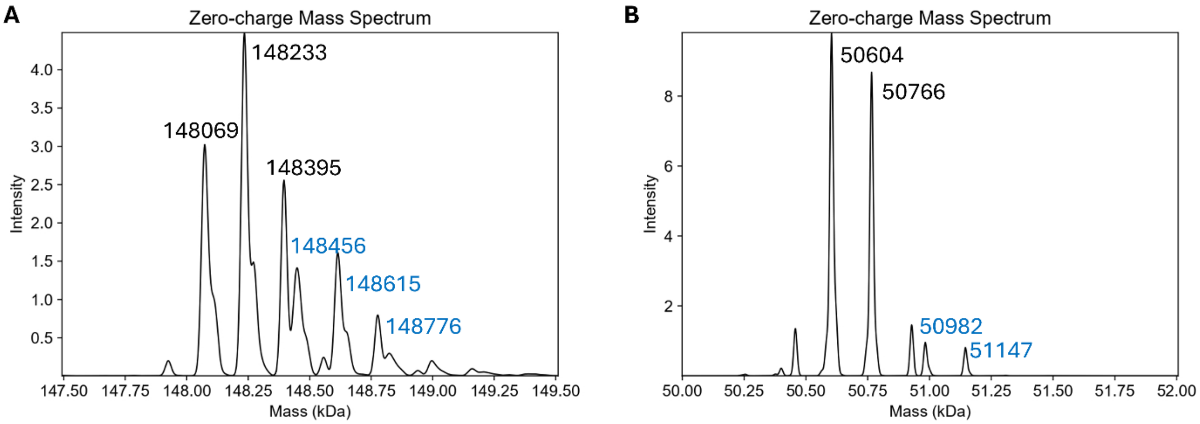

Figure S28: Intact mass analysis of A) native and B) reduced mAb (HC) Herceptin-PEG3-BCN conjugate. Depicted is a deconvoluted zoomed spectral region of interest with main peaks. Parent Herceptin mass is assigned in black and modified Herceptin-PEG3-BCN mass is in blue. Method details: MS Range: 500-3800. Source parameters: ESI Voltage 3100V, Source Temp: 120°C, Desolvation Temp: 450°C, Desolvation Gas: 800 L/hour, Cone Voltage: 60V, Source Offset: 20V. Gradient: Solvent A: 0.1% FA in H_2O. Solvent B: 0.1% FA in MeCN 0-0.5min - 400 ul/min 5% B (desalting step) 0.5-3min - 100 ul/min 10-60% B gradient 3-7min - Alternating 5% B and 95% B for column washing.

Herceptin cell labeling microscopy

SKBR3 cells were seeded on a 96-well plate at a density of 10 000 cells per well in complete DMEM medium. After 72h incubation, the cells were treated with 100 µL of 10 µg/mL Herceptin-PEG_3-BCN in serum-free DMEM for 1 h. The cells were washed 3 times with serum-free L-15 medium. After that, 2.5 µM **SulfoCy3Trz**$^+$ or **SulfoCy3Tz** were added to the cells in serum-free L-15 medium with Hoechst 33342 (diluted 1:10000) for 30 min. Then the cells were imaged on a confocal microscope without any washing (Figure S29). **Note: Herceptin-BrPD4 conjugate was not formed due to the instability of BrPD4.**

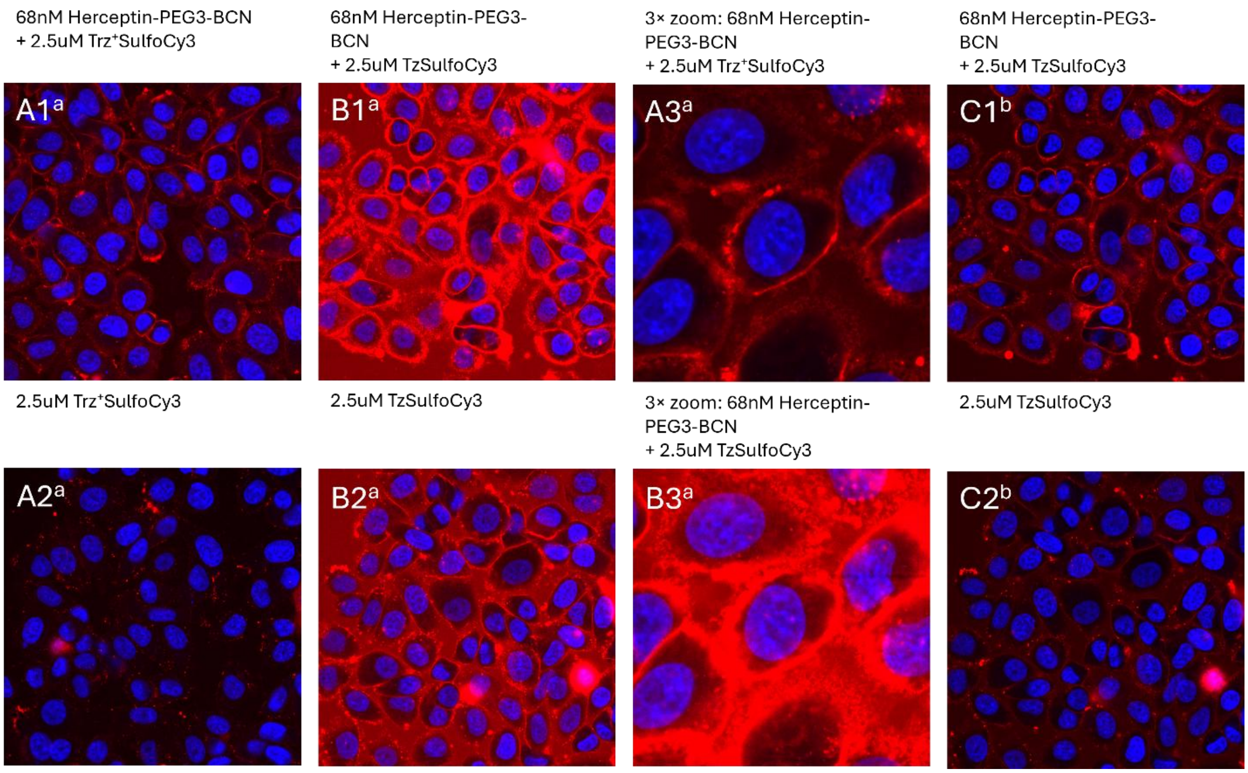

Figure S29: Confocal microscope images of SKBR3 cells treated with **Herceptin-PEG3-BCN** mAb conjugate and labeled with A1) A3) **Trz^{+}SulfoCy3** (3×zoomed), B1) B3) C1) **TzSulfoCy3** (3×zoomed). A2) B2) C2) are the corresponding controls (cells treated only with the probe). Click labeling is shown in red. Nuclei were stained with Hoechst 33342 and are shown in blue. [a]Microscope set-up: Cy3 (561 nm) laser intensity: 4%, and emission (410 – 694 nm) detector: 750 V. [b]Microscope set-up: Cy3 (561 nm) laser intensity: 3%, and emission (410 – 694 nm) detector: 700 V. Hoechst 33342 – laser 405 nm. Scale bar is 25 µm.

BCN-ConA cellular labeling

Concanavalin A was conjugated with BCN according to the standard protocol.[6] U2OS cells were seeded on a 96-well plate at a density of 25 000 cells per well in complete DMEM medium. The next day, the cells were incubated with 100 µL of 10 µg/mL BCN-ConA in PBS with 1mg/mL of BSA, Ca^{2+}, Mg^{2+} for 15 min. The cells were 3 times washed with a complete L-15 medium. After that, 2.5 µM **T(r)z$^{(+)}$SulfoCy3** was added to the cells in a complete L-15 medium with Hoechst 33342 (1 µg/mL) or DRAQ5 (5 µg/mL) for 30 min. Then the cells were imaged on a confocal microscope without washing (Figure S30).

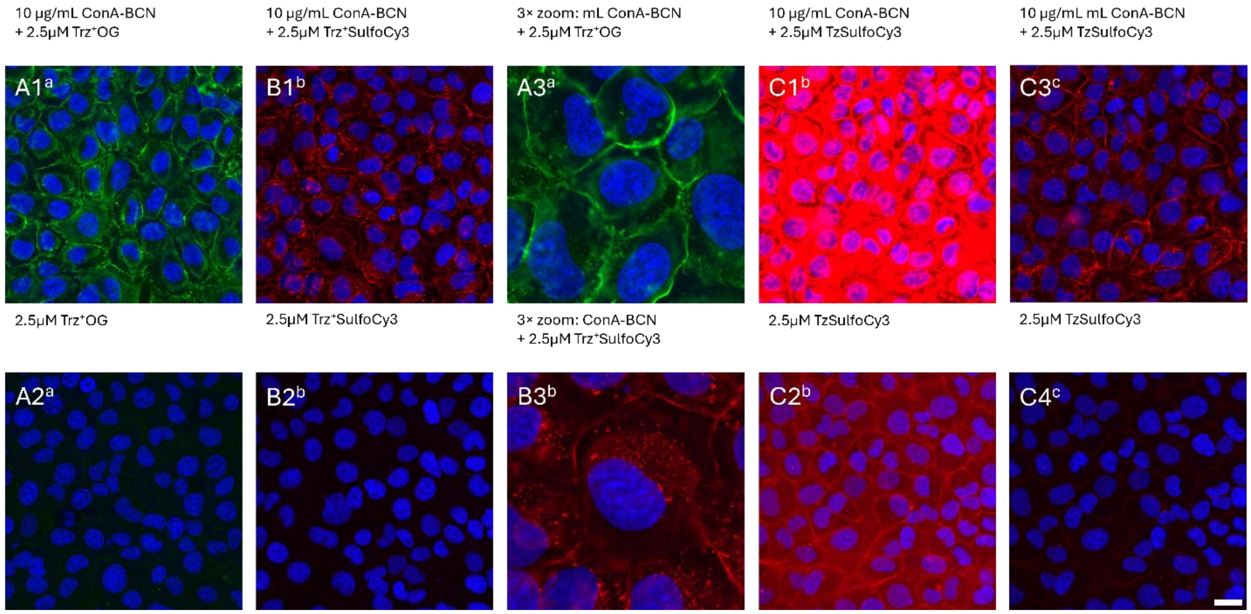

Figure S30: Confocal microscope images of U2OS cells treated with **BCN-ConA** protein conjugate and labeled with A1) A3) **Trz⁺OG** (3×zoomed), B1) B3) **Trz⁺SulfoCy3** (3×zoomed) C1) C3) **TzSulfoCy3**. A2) B2) C2) C4) are the corresponding controls (cells treated only with the probe). Click labeling is shown in red or green. Nuclei were stained with Hoechst 33342 or DRAQ5 and are shown in blue. [a]Microscope set-up: OG (488 nm) laser intensity: 3.5 %, and emission (490 – 658 nm) detector: 750 V. [b,c]Microscope set-up: Cy3 (561 nm) laser intensity: 4[b] or 2.5[c] %, and emission (410 – 694 nm) detector: 700[b] or 650[c] V. Hoechst 33342 – laser 405 nm. DRAQ5 – laser 639 nm. Scale bar is 25 µm.

Model drug's cellular labeling

U2OS cells were seeded on a 96-well plate at a density of 17 500 cells per well in complete DMEM medium. After 48h incubation, the cells were incubated with 2 µM Dasatinib-PEG3-BCN (**Dasatinib-BCN**) or Geldanamycin-PEG3-BCN (**Geldanamycin-BCN**) in complete DMEM for 2 h. The cells were washed 3 times with a complete L-15 medium. After that, 1 µM of each cell-permeable **T(r)z$^{(+)}$Fl** was added to the cells in a complete L-15 medium with DRAQ5 (5 µg/mL) or Hoechst 33342 (1 µg/mL) for 30 min. Then the cells were imaged on a confocal microscope without washing (Figure S31, S32).

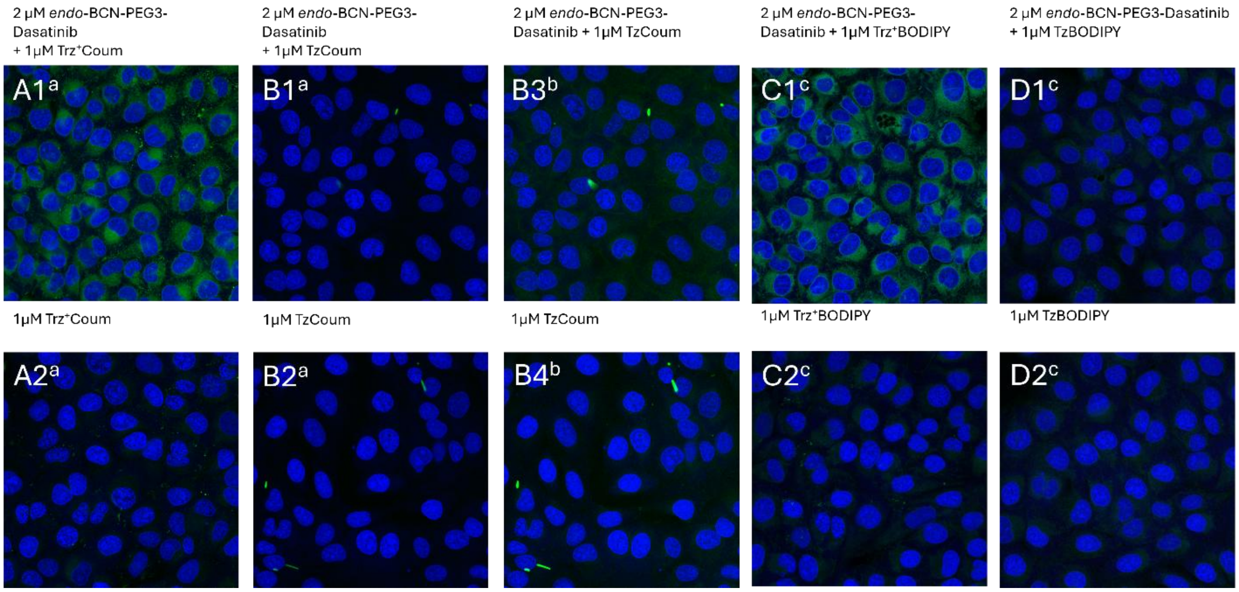
2 µM *endo*-BCN-PEG3-Dasatinib + 1µM Trz+Coum
2 µM *endo*-BCN-PEG3-Dasatinib + 1µM TzCoum
2 µM *endo*-BCN-PEG3-Dasatinib + 1µM TzCoum
2 µM *endo*-BCN-PEG3-Dasatinib + 1µM Trz+BODIPY
2 µM *endo*-BCN-PEG3-Dasatinib + 1µM TzBODIPY
A1a
B1a
B3b
C1c
D1c
1µM Trz+Coum
1µM TzCoum
1µM TzCoum
1µM Trz+BODIPY
1µM TzBODIPY
A2a
B2a
B4b
C2c
D2c

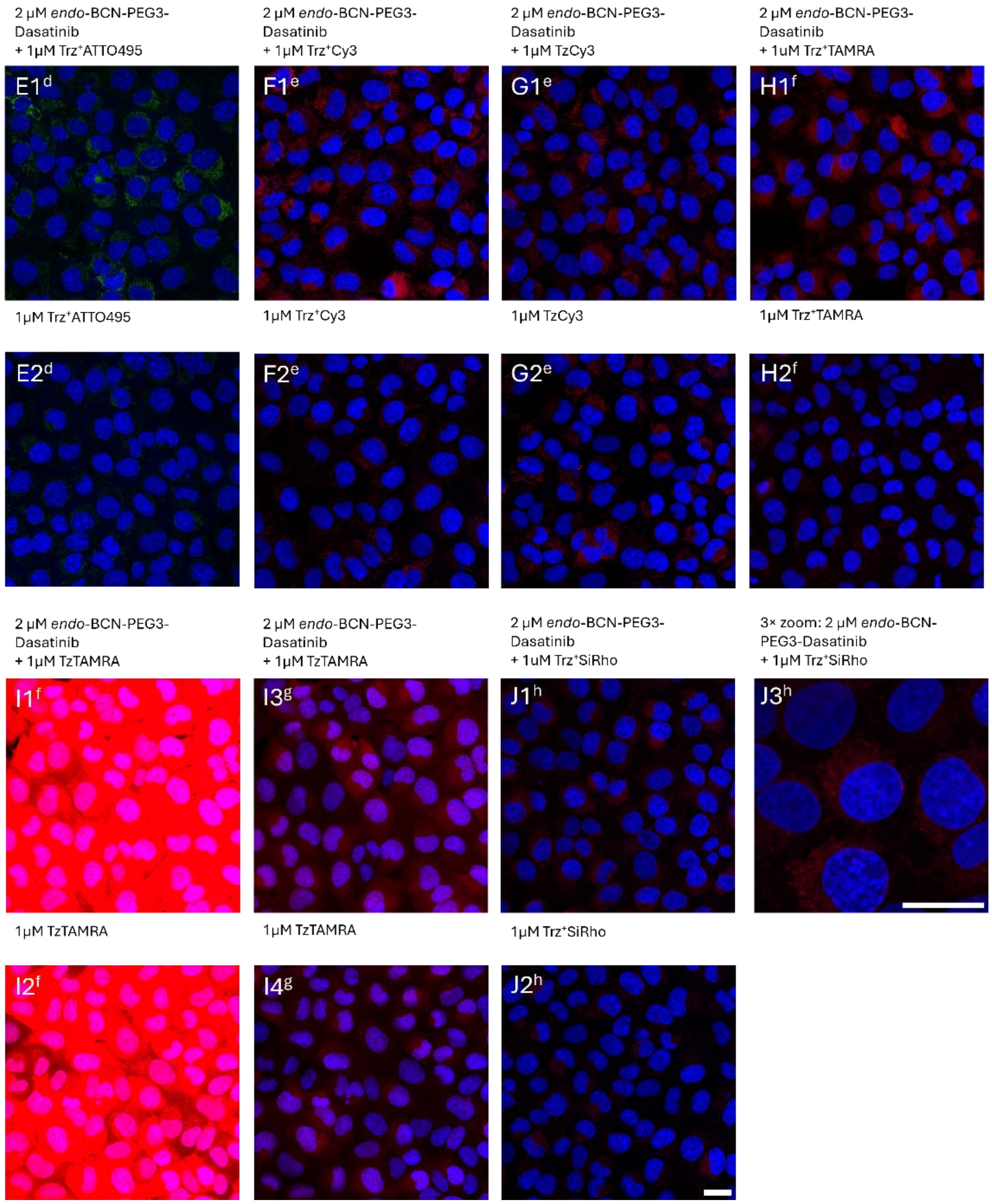

Figure S31: Confocal microscope images of U2OS cells treated with ***endo*-BCN-PEG3-Dasatinib** drug conjugate and labeled with A1) **Trz$^+$Coum**, B1) B3) **TzCoum**, C1) **Trz$^+$BODIPY**, D1) **TzBODIPY**, E1) **Trz$^+$ATTO495**, F1) **Trz$^+$Cy3**, G1) **TzCy3**, H1) **Trz$^+$TAMRA,** I1) I3) **TzTAMRA** J1) J3) **Trz$^+$SiRho** (3×zoomed). A2) B2) B4) C2) D2) E2) F2) G2) H2) I2) I4) J2) are the corresponding controls (cells treated only with the probe). Click labeling is shown in red or green. Nuclei were stained with Hoechst 33342 or DRAQ5 and are shown in blue. [a,b]Microscope set-up: Coum (405 nm) laser intensity: 2.5[a] or 3.5[b] %, and emission (410 – 676 nm) detector: 600[a] or 700[b] V. [c]Microscope set-up: BODIPY (488 nm) laser intensity: 2.5 %, and emission (490 – 658 nm) detector: 600 V. [d]Microscope set-up: ATTO495 (488 nm) laser intensity: 3.0 %, and emission (490 – 667 nm) detector: 700 V. [e]Microscope set-up: Cy3 (561 nm) laser intensity: 1.5 %, and emission (410 – 694 nm) detector: 550 V. [f,g]Microscope set-up: TAMRA (561 nm) laser intensity: 1.0[f] or 0.5[g] %, and emission (410 – 694 nm) detector: 600[f] or 550[g] V. [h]Microscope set-up: Cy5 (639 nm) laser intensity: 3.5 %, and emission (641 – 694 nm) detector: Hoechst 33342 – laser 405 nm. DRAQ5 – laser 639 nm. Scale bar is 25 µm.

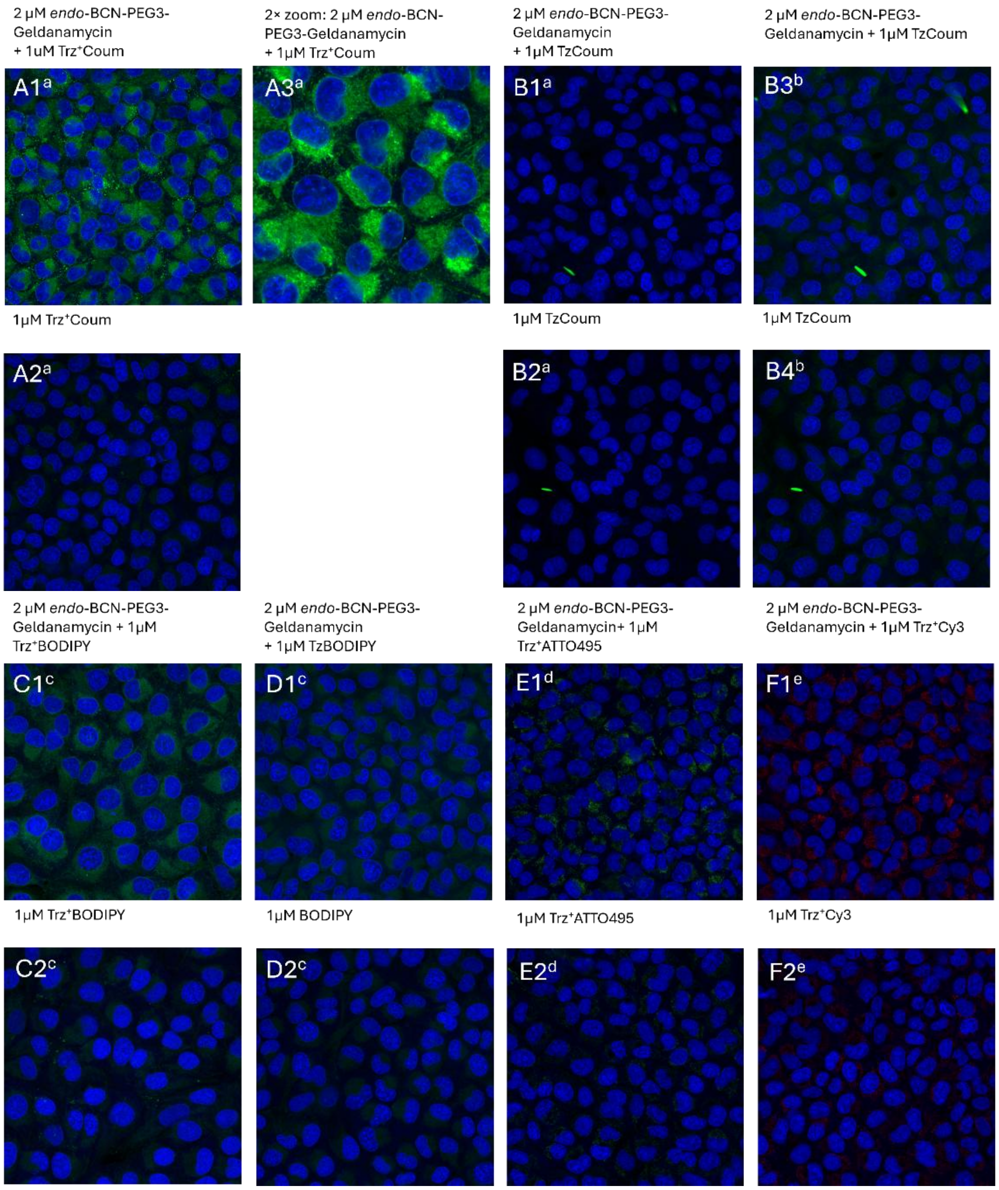

2 µM endo-BCN-PEG3-Geldanamycin + 1uM Trz⁺Coum
2× zoom: 2 µM endo-BCN-PEG3-Geldanamycin + 1µM Trz⁺Coum
2 µM endo-BCN-PEG3-Geldanamycin + 1µM TzCoum
2 µM endo-BCN-PEG3-Geldanamycin + 1µM TzCoum
A1a
A3a
B1a
B3b
1µM Trz⁺Coum
1µM TzCoum
1µM TzCoum
A2a
B2a
B4b
2 µM endo-BCN-PEG3-Geldanamycin + 1µM Trz⁺BODIPY
2 µM endo-BCN-PEG3-Geldanamycin + 1µM TzBODIPY
2 µM endo-BCN-PEG3-Geldanamycin+ 1µM Trz⁺ATTO495
2 µM endo-BCN-PEG3-Geldanamycin + 1µM Trz⁺Cy3
C1c
D1c
E1d
F1e
1µM Trz⁺BODIPY
1µM BODIPY
1µM Trz⁺ATTO495
1µM Trz⁺Cy3
C2c
D2c
E2d
F2e

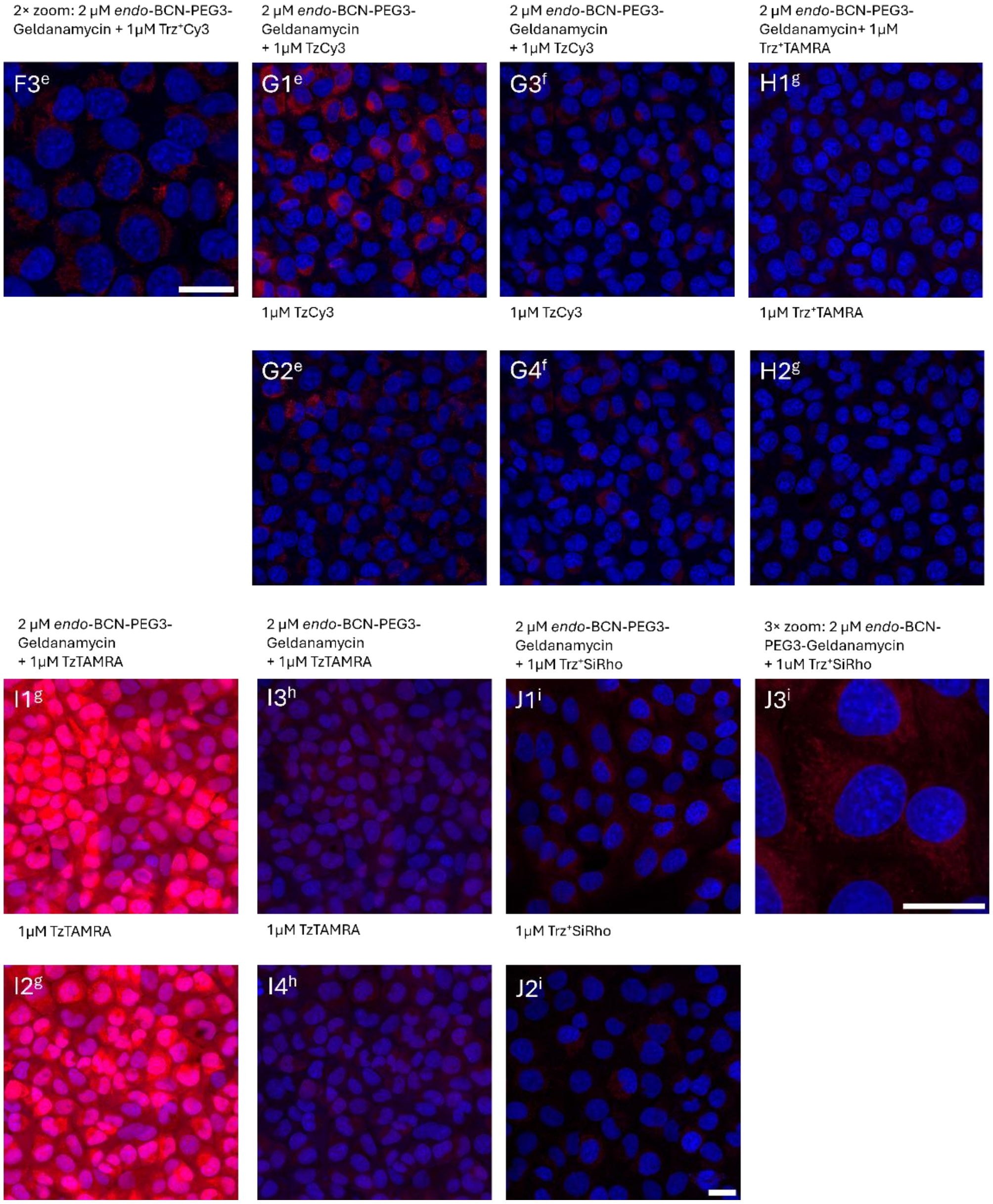

Figure S32: Confocal microscope images of U2OS cells treated with ***endo*-BCN-PEG3-Geldanamycin** drug conjugate and labeled with A1) A3) **Trz^+Coum** (2×zoomed), B1) B3) **TzCoum**, C1) **Trz^+BODIPY**, D1) **TzBODIPY**, E1) **Trz^+ATTO495**, F1) F3) **Trz^+Cy3** (2×zoomed), G1) G3) **TzCy3**, H1) **Trz^+TAMRA,** I1) I3) **TzTAMRA,** J1) J3) **Trz^+SiRho** (3×zoomed). A2) B2) B4) C2) D2) E2) F2) G2) G4) H2) I2) I4) J2) are the corresponding controls (cells treated only with the probe). Click labeling is shown in red or green. Nuclei were stained with Hoechst 33342 or DRAQ5 and are shown in blue. [a,b]Microscope set-up: Coum (405 nm) laser intensity: 2.0[a] or 3.0[b] %, and emission (410 – 676 nm) detector: 650[a] or 700[b] V. [c]Microscope set-up: BODIPY (488 nm) laser intensity: 3.0 %, and emission (490 – 658 nm) detector: 600 V. [d]Microscope set-up: ATTO495 (488 nm) laser intensity: 2.5 %, and emission (490 – 667 nm) detector: 700 V. [e,f]Microscope set-up: Cy3 (561 nm) laser intensity: 1.5[e] or 1.0[f] %, and emission (410 – 694 nm) detector: 550[e] or 500[f] V. [g,h]Microscope set-up: TAMRA (561 nm) laser intensity: 1.5[g] or 0.5[h] %, and emission (410 – 694 nm) detector: 550[g] or 500[h] V. [i]Microscope set-up: Cy5 (639 nm) laser intensity: 4.0 %, and emission (641 – 694 nm) detector: 750 V. Hoechst 33342 – laser 405 nm. DRAQ5 – laser 639 nm. Scale bar is 25 µm.

Fluorescence intensity from BCN-drug labeling

Table S13: Quantified fluorescence (ImageJ) from BCN-Dasatinib drug labeling

Value	Mean gray value		Turn-on	Integrated density		Turn-on
**$T(r)z^{(+)}Fl$**	**$T(r)z^{(+)}$**	**$T(r)z^{(+)}$+BCN**	**-**	**$T(r)z^{(+)}$**	**$T(r)z^{(+)}$+BCN**	**-**
**Trz^{+}Coum**	5.5	22.7	4	258.4	1057.4	4
**TzCoum**	2.9	3.3	1.1	135.8	154.5	1.1
**Trz^{+}BODIPY**	6.6	57.1	9	305.3	2662.4	9
**TzBODIPY**	4.5	4.4	0	210.3	203.1	0
**Trz^{+}Cy3**	4.2	21.0	5	312.9	1547.0	5
**TzCy3**	5.4	8.0	1.5	397.2	591.2	1.5

Table S14: Quantified fluorescence (ImageJ) from BCN-Geldanamycin drug labeling

Value	Mean gray value		Turn-on	Integrated density		Turn-on
**$T(r)z^{(+)}Fl$**	**$T(r)z^{(+)}$**	**$T(r)z^{(+)}$+BCN**	**-**	**$T(r)z^{(+)}$**	**$T(r)z^{(+)}$+BCN**	**-**
**Trz^{+}Coum**	4.9	24.1	5	226.9	1121.4	5
**TzCoum**	1.1	1.8	1,6	52.4	85.7	1.6
**Trz^{+}BODIPY**	6.6	15.4	2.3	305.3	716.9	2.3
**TzBODIPY**	5.0	10.9	2.2	234.4	509.9	2.2
**Trz^{+}Cy3**	7.8	11.7	1.5	365.4	543.1	1.5
**TzCy3**	5.0	5.9	1.2	233.9	273.8	1.2

Additional model drug's cellular labeling

U2OS cells were seeded on a 96-well plate at a density of 25000 cells per well. After 48h incubation, the cells were incubated with 2 μM Dasatinib-PEG3-BCN (**Dasatinib-BCN**) or Geldanamycin-PEG3-BCN (**Geldanamycin-BCN**) in complete DMEM for 2 h. The cells were washed 3 times with a complete L-15 medium. After that, 1 μM of each cell-permeable **$T(r)z^{(+)}Fl$** was added to the cells in a complete L-15 medium with DRAQ5 (5 μg/mL) for 30 min. Then the cells were imaged on a confocal microscope without washing (Figure S33 and S34).

Alternatively, 2 μM of **BCN-Drug** was incubated with 1 μM of **$T(r)z^{+}$Coum** in a complete L-15 medium for 45 min. After that, the resulting click product was added to the cells in a complete L-15 medium with DRAQ5 (5 μg/mL) for 30 min. Then the cells were imaged on a confocal microscope (Figure S33 and S34).

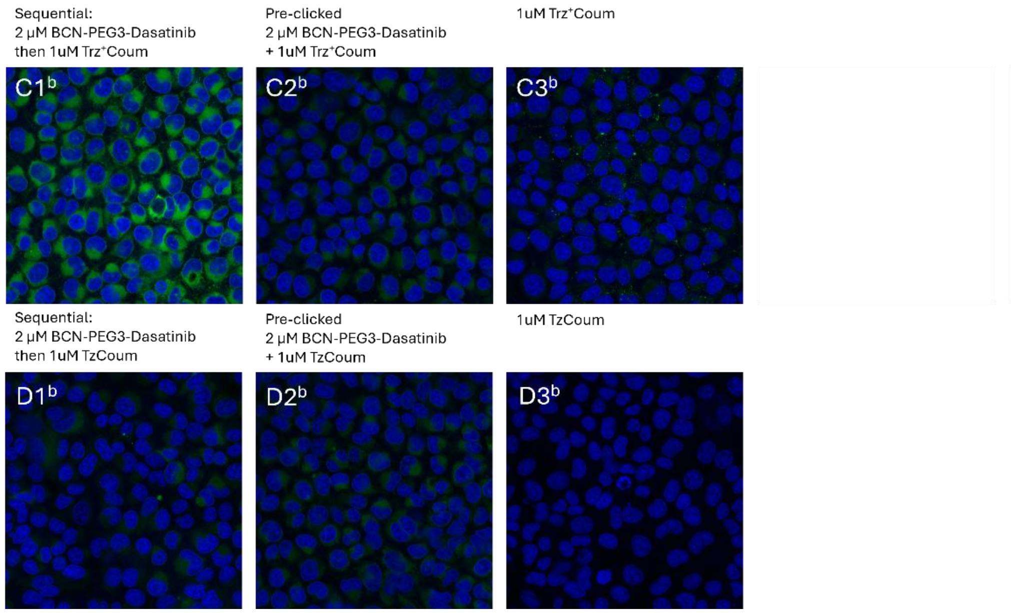

Figure S33: Confocal microscope images of U2OS cells treated with ***endo*-BCN-PEG3-Dasatinib** conjugate and labeled with C1) **Trz$^+$Coum**, D1) **TzCoum** or C2) D2) pre-clicked ***endo*-BCN-PEG3-Dasatinib+T(r)z$^{(+)}$Coum** conjugate. C3) D3) are the corresponding controls (cells treated only with the probe). Click labeling is shown in green. Nuclei were stained with DRAQ5 and are shown in blue. [b]Microscope set-up: Coum (405 nm) laser intensity: 2.5 %, and emission (410 – 676 nm) detector: 600 V.

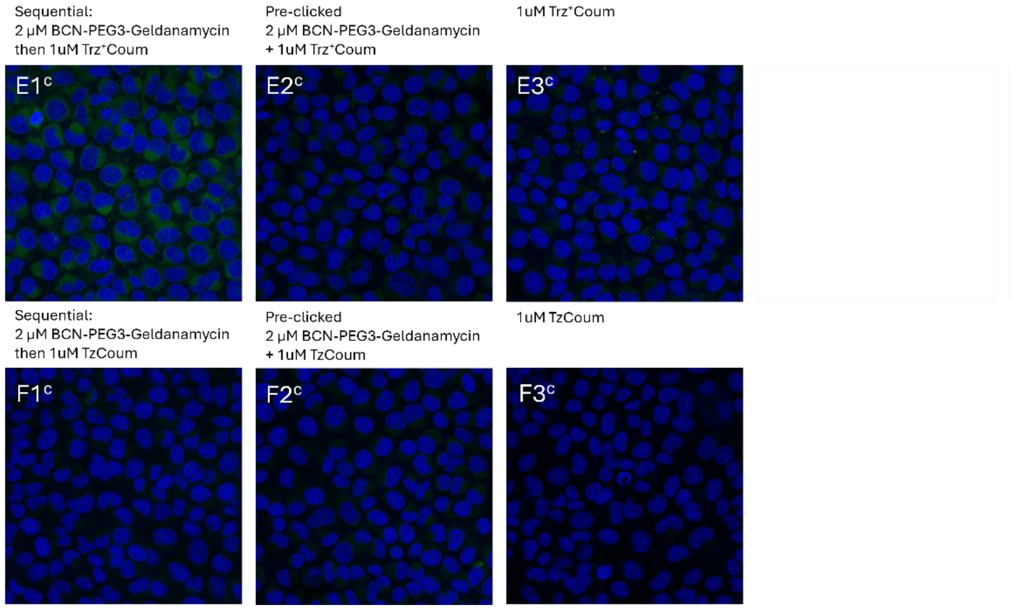

Figure S34: Confocal microscope images of U2OS cells treated with ***endo*-BCN-PEG3-Geldanamycin** conjugate and labeled with E1) **Trz$^+$Coum**, F1) **TzCoum** or E2) F2) pre-clicked ***endo*-BCN-PEG3-Geldanamycin+T(r)z$^{(+)}$Coum** conjugate. E3) F3) are the corresponding controls (cells treated only with the probe). Click labeling is shown in green. Nuclei were stained with DRAQ5 and are shown in blue. [c]Microscope set-up: Coum (405 nm) laser intensity: 2.0 %, and emission (410 – 676 nm) detector: 650 V.

ER HaloTag cellular labeling

ER HaloTag plasmid production

The cDNA of HaloTag® was amplified from vector pFN21A-GAP43 (Promega) using PCR and cloned into pCDH-CMV-MCS-EF1α-Puro (SBI Systems Biosiences) between BamHI/NotI restriction sites. KDEL peptide causing the retention of proteins in the endoplasmic reticulum (ER) was introduced at the C-terminus of the HaloTag by digesting the resulting plasmid with XhoI and NotI and ligating the annealed phosphorylated oligos encoding the KDEL peptide. Next, the N-terminal leader sequence of human IgK signal peptide (MDMRVLAQLLGLLLLCFPGARC) was added to the HaloTag-KDEL construct by digesting the vector with BamHI/EcoRI and ligating in the DNA oligos encoding the desired protein sequence. The final plasmid pCDH-ER-HALO-KDEL was used to prepare lentiviral particles. U2OS cells were infected with lentiviral particles, and 2 days post-infection positive transductants were selected using 2µg/mL of Puromycin.

Preparation of recombinant lentiviral particles

HEK293T cells growing on the 10 cm dish were transfected with a mixture of pMD2G psPAX2 and the lentiviral transfer vector pCDH-ER-HALO-KDEL (4/7/9 µg) using Lipofectamine 3000 (ThermoFisher). 24h post-transfection the media was replaced with DMEM containing 20% FBS and cells were cultivated for another 72 hours. Medium containing the lentiviral particles was collected and filtered using a 45 µm syringe filter (Millipore).

Oligonucleotide sequences

Amplification of HaloTag coding sequence: Halo_trans_fw: ccGGATCCaATGGCAGAAATCGGTA containing BamHI site. Halo_trans_rv: ttgcggccgcttaGCCGGAAATCTC containing NotI restriction site.

Insertion of KDEL peptide coding sequence: KDEL_fw: TCGAaATTTCCGGCaaagatgaactgtaagc. KDEL_rv: ggccgcttacagttcatctttGCCGGAAATt.

Insertion of ER targeting sequence: Human IgKVIII_FW: AATTCatggatatgcgcgtgctggcgcagctgctgggcctgctgctgctgtgctttccgggcgcgcgctgcAAG. Human IgKVIII_rv: GATCCttgcagcgcgcgcccggaaagcacagcagcagcaggcccagcagctgcgccagcacgcgcatatccatG.

Modification of U2OS cells with ER HaloTag plasmid

(A) Transduction

500 000 U2OS cells were seeded on a 6-well plate. After 24 hours of cultivation media was aspired and replaced with 1 mL of fresh DMEM containing 10% FBS. Cells were transduced with 500 µL of the filtered supernatant containing the viral particles. 2 days later, the medium was replaced with 2 mL of fresh DMEM containing 10% FBS containing 2µg/mL of Puromycin (Sigma-Aldrich) and cells were selected for another 2 days. The population of ER HaloTag-positive cells was sorted out by standard protocol against **HaloTag-BODIPY**.

(B) Transfection

For a proper comparison of the transduced stable U2OS cell line with the temporary expression of ER HaloTag, we also transfected U2OS cells according to the following procedure. U2OS cells were maintained in growth media containing Dulbecco's modified eagle medium (DMEM, Biowest: L0103-500) supplemented with 10% (v:v) FBS (biowest: L0103-500) in a humidified incubator set at 37 °C/5% CO_2. Transfection mixture was prepared from 2.00 µL of DNA (1.00 µg/µL) pre-mixed with 7.5 µL of Lipofectamine 2000 (Invitrogen: 11668019) in 1.0 mL of antibiotic-free DMEM/10% FBS. After 15 min uof incubation at rt, U2OS cells (500 000 in 2.00 mL) were combined with the transfection mixture in accordance with the manufacturer's protocol in antibiotic-free DMEM/10% FBS at 80–90% confluence. U2OS cells were seeded on a 96-well plate at a density of 25,000 cells per well 20 h before HaloTag labeling experiment.

ER HaloTag cell labeling microscopy

Modified (transduced or transfected) U2OS cells were seeded on a 96-well plate at a density of 25 000 cells per well in complete DMEM medium. After 24h incubation, the cells were incubated with 10 µM HaloTag ligand-BCN conjugate (**HaloTag-BCN**) in serum-free DMEM for 30 min. The cells were 3 times quicky washed with complete L-15 medium and incubated in 100 µL of new media for 1 h with one media change to remove unbound HaloTag ligands. After that, 1 µM of **T(r)z$^{(+)}$TAMRA** was added to the cells in a complete L-15 medium with DRAQ5 (5 µg/mL) for 15 min. Then the cells were imaged on a confocal microscope without further washing (Figure S35, S36).

Alternatively, the same cells were incubated with 2 µM HaloTag ligand-Trz^{+}Coum conjugate (**HaloTrz^{+}Coum**) in serum-free DMEM for 30 min. After the same washing as indicated previously, 5 µM of the ***endo*-BCN-PEG3-NH2** was added to the cells in a complete L-15 medium with DRAQ5 (diluted 1:1000) for 15 min, followed by a confocal microscopy imaging.

Finally, the resulting signal was colocalized with ER-Tracker™ Red, which was added (instead of the DRAQ5) as 1 µM solution in PBS for 15 min. After this staining, medium was exchanged for a complete L-15 medium before colocalization imaging. The proper expression of ER HaloTag protein was always verified by applying 1 µM **HaloTag-BODIPY** in serum-free DMEM for 30 min and washing.

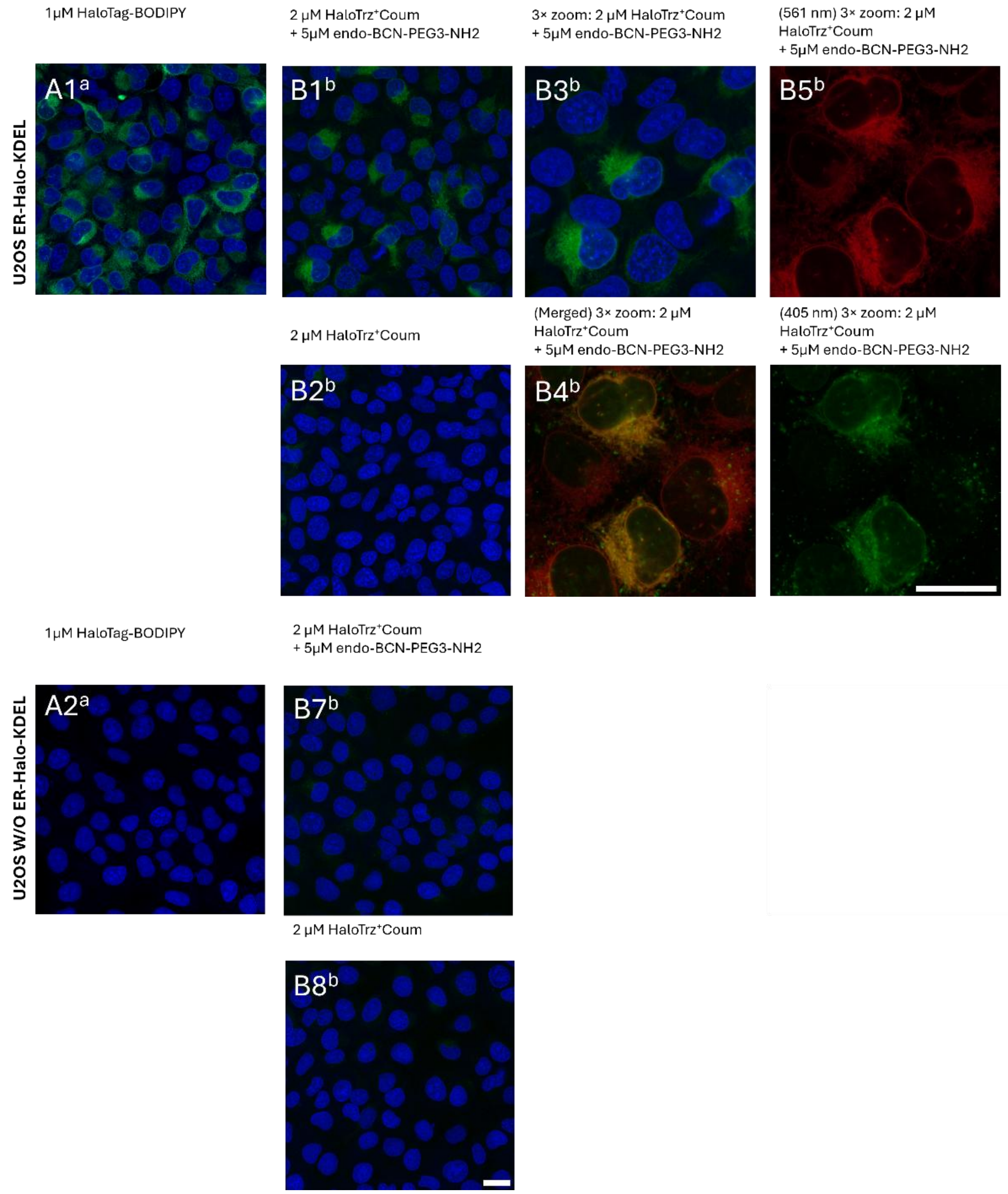

Figure S35: Confocal microscope images of transduced (ER HaloTag KDEL plasmid) U2OS or U2OS cells treated with A1) A2) **HaloTag-BODIPY**, B1) B3) B4) B5) B6) B7) **HaloTrz⁺Coum** and labeled with B1) B3) B4) B5) B6) B7) ***endo*-BCN-PEG3-NH2** (3×zoomed). B2) B8) are the corresponding controls (cells treated only with the probe). Click labeling is shown in red or green. Nuclei were stained with DRAQ5 and are shown in blue. [a]Microscope set-up: BODIPY (488 nm) laser intensity: 1.5 %, and emission (490 – 658 nm) detector: 650 V. [b]Microscope set-up: Coum (405 nm) laser intensity: 2.0 %, and emission (410 – 676 nm) detector: 650 V. DRAQ5 – laser 639 nm. mCherry – laser 561 nm. Scale bar is 25 μm.

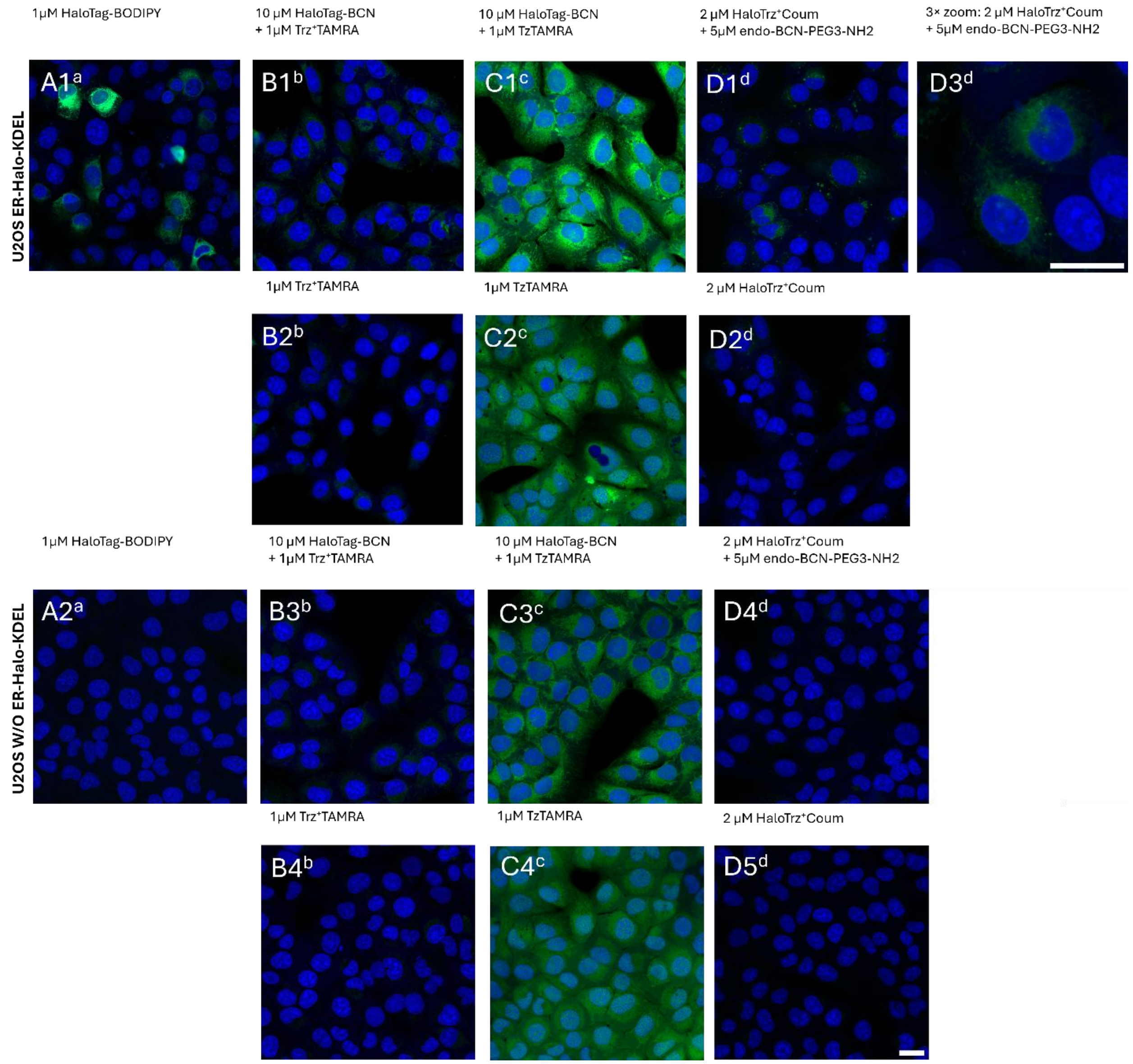

Figure S36: Confocal microscope images of transfected (ER HaloTag KDEL plasmid) U2OS or U2OS cells treated with A1) A2) **HaloTag-BODIPY**, B1) C1) B3) C3) **HaloTag-BCN**, D1) D3) D4) **HaloTrz$^+$Coum** and labeled with B1) B3) **Trz$^+$TAMRA,** C1) C3) **TzTAMRA,** D1) D3) D4) ***endo*-BCN-PEG3-NH2** (3×zoomed). B2) B4) C2) C4) D2) D5) are the corresponding controls (cells treated only with the probe). Click labeling is shown in green. Nuclei were stained with DRAQ5 and are shown in blue. [a]Microscope set-up: BODIPY (488 nm) laser intensity: 3.0 %, and emission (490 – 658 nm) detector: 750 V. [b]Microscope set-up: TAMRA (561 nm) laser intensity: 1.5 %, and emission (410 – 694 nm) detector: 550 V. [c]Microscope set-up: Coum (405 nm) laser intensity: 2.5 %, and emission (410 – 676 nm) detector: 650 V. DRAQ5 – laser 639 nm. Scale bar is 25 μm.

Amber suppression

Cell culture

HEK293T (ATCC CRL-3216) cells were maintained in Dulbecco's modified Eagle's medium (DMEM, Gibco 41965-039) supplemented with 10% FBS (Gibco A5256801), 1% penicillin-streptomycin (Gibco 15140-122), 1% sodium pyruvate (Gibco 11360-070) and 1% Glutamax (Gibco 35050-061).

The cells were cultured at 37°C in a 5% CO_2 atmosphere and passaged - using trypsin (0.05%; Gibco 25300-054) - every 3–4 days up to 20 passages.

Bioorthogonal labeling of live cells

HEK293T (40,000 cells/well) were transferred into µ-Slide 8 well plates (Ibidi 80827) and were incubated for 40 h at 37°C in a 5% CO_2 atmosphere. Ibidi plates were pre-treated with 0.02 mg/mL Poly-D-lysine (Gibco A3890401) for 4 hours at room temperature and washed afterward. Bioorthogonally reactive chemical reporter BCN was administered in non-canonical amino acid (ncAA). Therefore, cells were co-transfected with 0.25 µg $IR^{K676TAG}$-miRFP plasmid for extracellular or LaminA-$Linker^{TAG}$-miRFP[30] for intracellular labeling in combination with 0.25 µg $tRNA^{Pyl}$/NES-$PylRS^{AF}$ plasmid (obtained from Ivana Nikic-Spiegel within Material Transfer Agreement)[31] using Lipofectamine 3000 (Thermo Scientific L3000001) transfection agent for four hours according to the manufacturer's protocol in the presence of 250 µM BCN^{endo}-lysine (Sichem SC-8014) during the transfection. Subsequently, the supernatant was replaced with ncAA free medium overnight. One day after transfection, cells were labeled with the fluorescent dyes **TzSulfoCy3, Trz^+SulfoCy3, TzTAMRA,** and **Trz^+TAMRA** at a concentration of 0.1 µM (in complete DMEM (Gibco 21063-029) for 30 min for EC and 2 h for IC labeling at 37°C in the dark. In case of no-wash condition, the samples were imaged immediately after labeling -we extended the labeling time by another 2 hours in case of IC labeling - without a washing step (Figure S37).

Confocal imaging and analysis

Confocal images were acquired on a Leica TCS SP8 STED 3X microscope using the 638 nm for excitation of the reporter fluorescent protein and 552 nm laser for the probes. The images were taken using a Leica HC PL APO 40x/1.30 oil immersion objective using the Leica HyD detectors. Spectral detection parameters were: exc.: 638 nm/ em. range: 650-800 nm for reporter protein mi-RFP; exc.: 552 nm/ em. range: 565-620 nm for compounds **TzSulfoCy3, Trz^+SulfoCy3, TzTAMRA** and **Trz^+TAMRA**.

Images were analyzed using Leica Application Suite X and Fiji for ImageJ software (NIH).

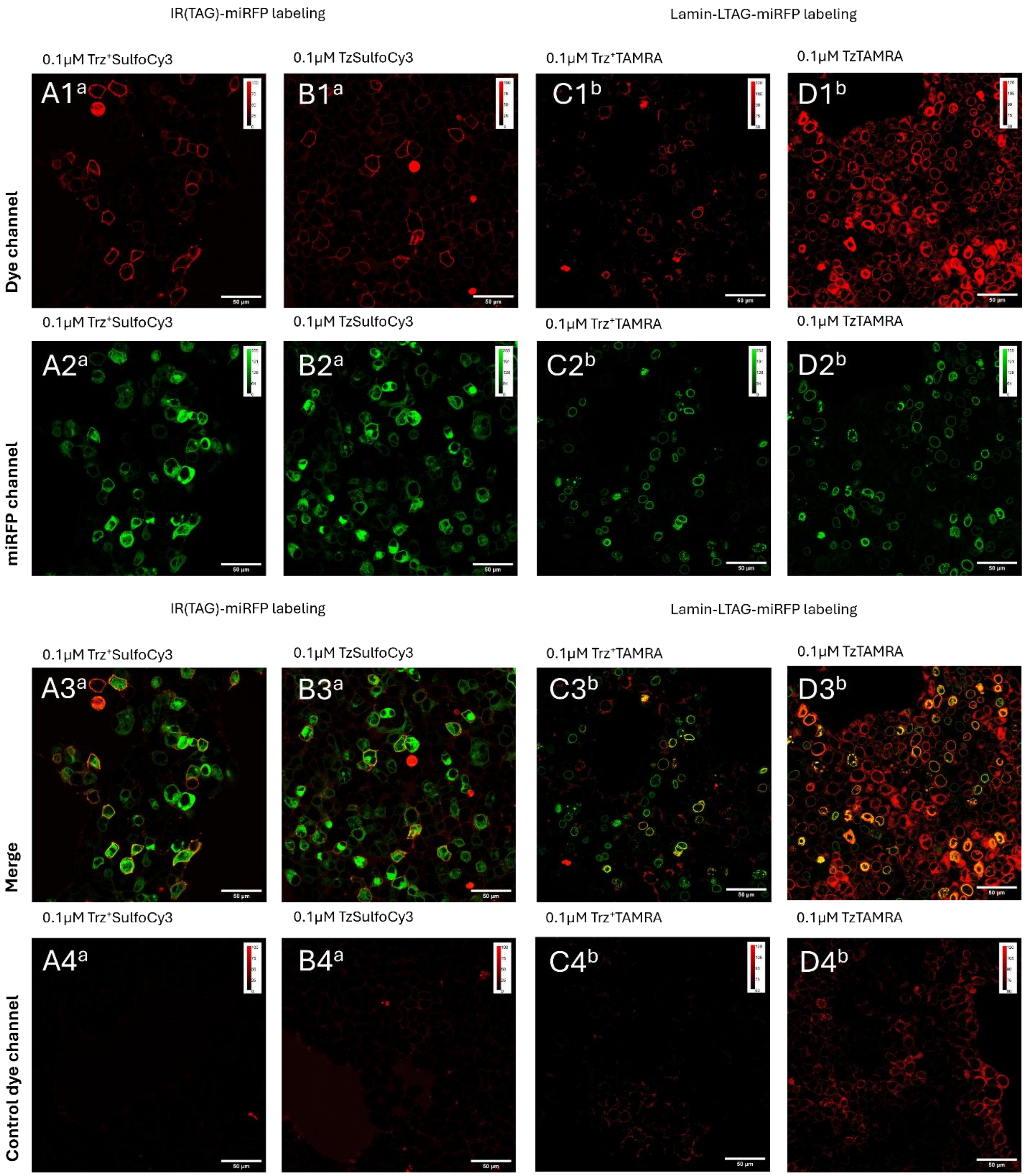
IR(TAG)-miRFP labeling
Lamin-LTAG-miRFP labeling
Dye channel
miRFP channel
0.1μM Trz⁺SulfoCy3
0.1μM TzSulfoCy3
0.1μM Trz⁺TAMRA
0.1μM TzTAMRA
A1a
B1a
C1b
D1b
A2a
B2a
C2b
D2b
IR(TAG)-miRFP labeling
Lamin-LTAG-miRFP labeling
Merge
Control dye channel
A3a
B3a
C3b
D3b
A4a
B4a
C4b
D4b

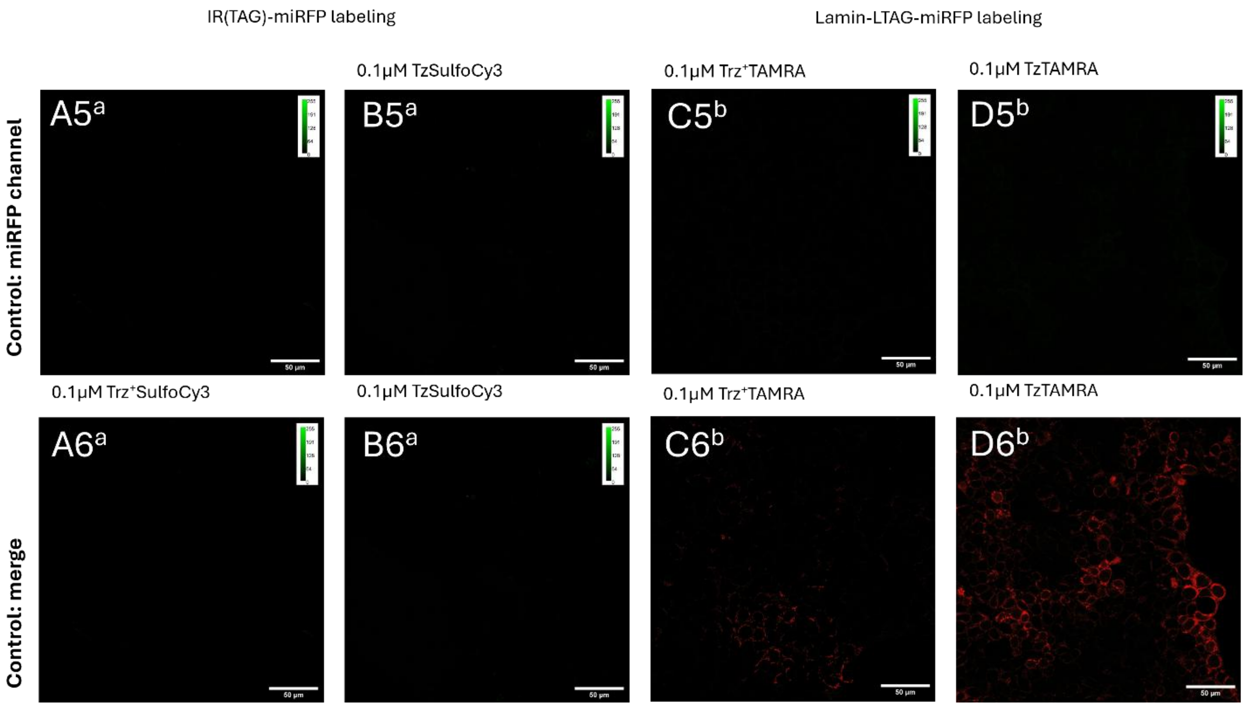

Figure S37: Confocal microscope images of labeling of **IR(TAG)-miRFP** or **Lamin-LTAG-miRFP** containing **BCN-Lys** on live HEK293T cells with A1) A2) A3) **Trz$^+$SulfoCy3**, B1) B2) B3) **TzSulfoCy3**, C1) C2) C3) **Trz$^+$TAMRA**, D1) D2) D3) **TzTAMRA**. A4) A5) A6) B4) B5) B6) C4) C5) C6) D4) D5) D6) are the corresponding controls (cells treated only with the probe). Dye cchannel is shown in red and miRFP in green. [a,b]Microscope set-up: dye channel: λ_{ex} = 552 nm and λ_{em} = 565 – 600 nm; reporter protein miRFP channel: λ_{ex} = 638 nm and λ_{em} = 650 – 800 nm. Scale bar is 50 µm.

Double labeling of BCN-Sia after metabolic incorporation

U2OS cells were seeded on a 96-well plate at a density of 17 500 cells per well in complete DMEM medium. After 24h incubation, the cells were incubated with 1 mM Sialic acid-BCN conjugate (**BCN-Sia**)[5] in complete DMEM for 48 h. The cells were washed 3 times with complete L-15 medium. After that, 2.5 µM of the selected cell-impermeable **T(r)z$^{(+)}$Fl** was added to the cells in complete L-15 medium for 30 min. Finally, it was exchanged for 1 µM of the selected cell-permeable **T(r)z$^{(+)}$Fl** with DRAQ5 (5 µg/mL) for 15 min. Then the cells were imaged on a confocal microscope without any washing (Figure S38).

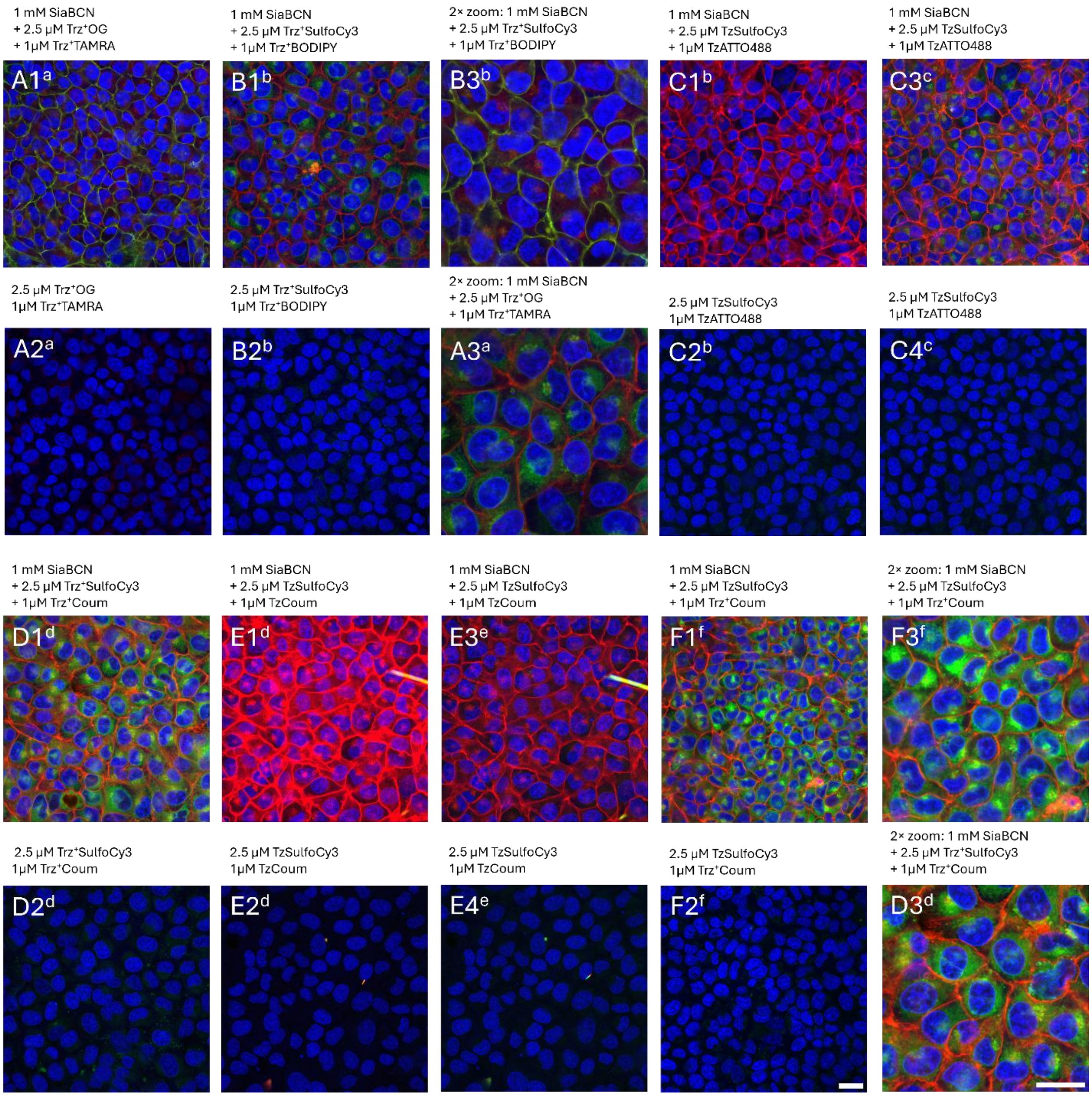

Figure S38: Confocal microscope images of U2OS cells treated with ***endo*-BCN-Sia** sugar conjugate and labeled with A1) A3) **Trz^{+}OG + Trz^{+}TAMRA** (2×zoomed), B1) B3) **Trz^{+}SulfoCy3 + Trz^{+}BODIPY** (2×zoomed), C1) C3) **TzSulfoCy + TzBODIPY**, D1) D3) **Trz^{+}SulfoCy3 + Trz^{+}Coum**, E1) E3) **TzSulfoCy3 + TzCoum**, F1) F3) **TzSulfoCy3 + Trz^{+}Coum** (2×zoomed). A2) B2) C2) C4) D2) E2) E24) F2) are the corresponding controls (cells treated only with the probe). Click labeling is shown in red or green. Nuclei were stained with DRAQ5 and are shown in blue. [a]Microscope set-up: OG (488 nm) laser intensity: 2.0 %, and emission (490 – 658 nm) detector: 550 V. TAMRA (561 nm) laser intensity: 1.0 %, and emission (410 – 694 nm) detector: 600 V. [b,c]Microscope set-up: Cy3 (561 nm) laser intensity: 2.0[b,c] %, and emission (410 – 694 nm) detector: 600[b,c] V. BODIPY (488 nm) laser intensity: 3.0[b] or 4.0[c] %, and emission (490 – 658 nm) detector: 600[b] or 700[c] V. [d,e]Microscope set-up: Cy3 (561 nm) laser intensity: 3.0[d] or 2.5[e] %, and emission (410 – 694 nm) detector: 700[d] or 650[e] V. Coum (405 nm) laser intensity: 2.5[d] or 3.0[e] %, and emission (410 – 676 nm) detector: 650[d] or 700[e] V. [f]Microscope set-up: Cy3 (561 nm) laser intensity: 2.0 %, and emission (410 – 694 nm) detector: 650 V. Coum (405 nm) laser intensity: 2.5 %, and emission (410 – 676 nm) detector: 650 V. DRAQ5 – laser 639 nm. Scale bar is 25 μm.

Cytotoxicity screening

Compound cytotoxicity was evaluated in U2OS cells (obtained from ATCC, Manassas, VA, USA, and negatively tested for Mycoplasma by MycoAlert™ Mycoplasma Detection kit, Lonza). The cells were maintained in a DMEM high glucose culture medium containing 10% FBS and 1% GlutaMax without antibiotics. On the day of the experiment, cells were seeded in 384-well white plates (Thermo Fisher Scientific, Waltham, USA) at a concentration between 2,000 – 50,000 cells per well overnight. The next day indicated concentrations of the test compounds were added and the cells were incubated at 37°C, 5% CO_2 for 24 h (no wash setup). Alternatively, the whole volume of media containing the compounds was exchanged for fresh media after 1h and the plate was further incubated for 24 h (washout setup). After that, CellTiter-Glo® 2.0 detection reagent (Promega, Madison, USA) was added. The plate was left on a shaker (350 rpm) for 20 min at RT. Luminescence was then measured by a multimode plate reader. The signal of the compound-treated cells was related to the value of untreated control which was arbitrarily set to 100% viability. IC_{50} values were calculated from dose-response curves using GraphPad Prism software's non-linear regression method (Graph S2).

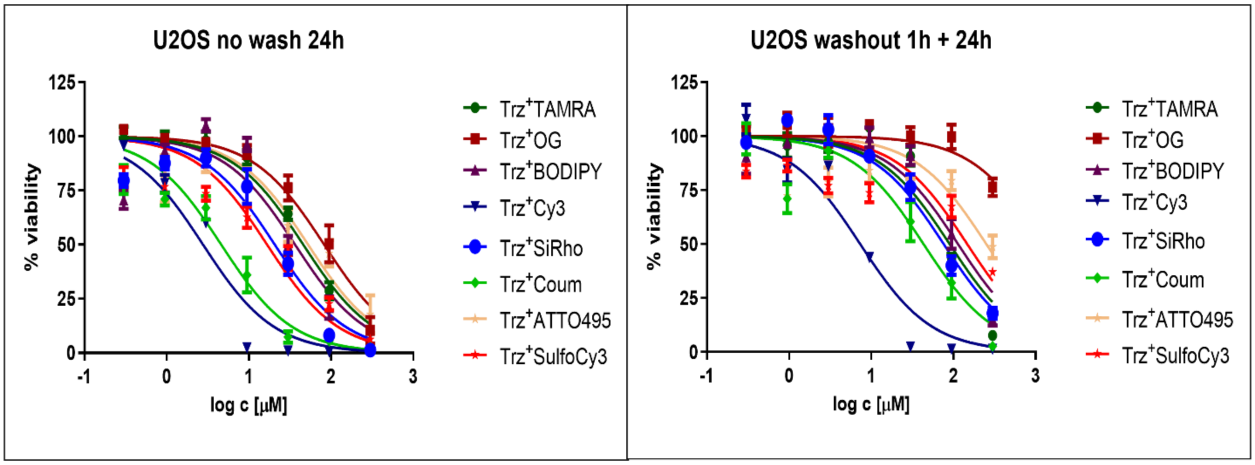

Graph S2: Cytotoxicity of fluorogenic triaziniums on U2OS cell line.

Table S15. Cytotoxicity of triazinium-dye conjugates

Compound	IC_{50} (µM) washout	IC_{50} (µM) no wash
Trz⁺ TAMRA	89.7	45.8
Trz⁺ OG	>300	82.4
Trz⁺ Bodipy	114	36.4
Trz⁺ Cy3	7.3	2.8
Trz⁺ Coum	43.2	4.6
Trz⁺ SiRho	76.0	20.6
Trz⁺ ATTO495	251	52.5
Trz⁺ SulfoCy3	147	16.6

Literature

(1) Galeta, J.; Šlachtová, V.; Dračínský, M.; Vrabel, M. Regio- and Diastereoselective 1,3-Dipolar Cycloadditions of 1,2,4-Triazin-1-ium Ylides: a Straightforward Synthetic Route to Polysubstituted Pyrrolo[2,1-f][1,2,4]triazines. *ACS Omega* **2022,** *7* (24), 21233-21238.
(2) Katoh, T.; Yoshikawa, M.; Yamamoto, T.; Arai, R.; Nii, N.; Tomata, Y.; Suzuki, S.; Koyama, R.; Negoro, N.; Yogo, T. Parallel fluorescent probe synthesis based on the large-scale preparation of BODIPY FL propionic acid. *Bioorg. Med. Chem. Lett.* **2017,** *27* (5), 1145-1148.
(3) Szatmári, Á.; Cserép, G. B.; Molnár, T. Á.; Söveges, B.; Biró, A.; Várady, G.; Szabó, E.; Németh, K.; Kele, P. A Genetically Encoded Isonitrile Lysine for Orthogonal Bioorthogonal Labeling Schemes. *Molecules* **2021,** *26* (16), 4988.
(4) Singh, V.; Wang, S.; Kool, E. T. Genetically Encoded Multispectral Labeling of Proteins with Polyfluorophores on a DNA Backbone. *J. Am. Chem. Soc.* **2013,** *135* (16), 6184-6191.
(5) Agarwal, P.; Beahm, B. J.; Shieh, P.; Bertozzi, C. R. Systemic Fluorescence Imaging of Zebrafish Glycans with Bioorthogonal Chemistry. *Angew. Chem. Int. Ed.* **2015,** *54* (39), 11504-11510.
(6) Šlachtová, V.; Bellová, S.; La-Venia, A.; Galeta, J.; Dračínský, M.; Chalupský, K.; Dvořáková, A.; Mertlíková-Kaiserová, H.; Rukovanský, P.; Dzijak, R.; Vrabel, M. Triazinium Ligation: Bioorthogonal Reaction of N1-Alkyl 1,2,4-Triazinium Salts**. *Angew. Chem. Int. Ed.* **2023,** *62* (36), e202306828.
(7) Marquard, A. N.; Carlson, J. C. T.; Weissleder, R. Expanding the Scope of Antibody Rebridging with New Pyridazinedione–TCO Constructs. *Bioconjugate Chem.* **2020,** *31* (6), 1616-1623.
(8) Šlachtová, V.; Bellová, S.; Vrabel, M. Synthesis of C3-Substituted N1-tert-Butyl 1,2,4-Triazinium Salts via the Liebeskind–Srogl Reaction for Fluorogenic Labeling of Live Cells. *J. Org. Chem.* **2024**.
(9) Maity, B.; Chatterjee, A.; Seth, D. The photophysics of 7-(diethylamino)coumarin-3-carboxylic acid N-succinimidyl ester in reverse micelle: excitation wavelength dependent dynamics. *RSC Adv* **2014,** *4* (7), 3461-3471.
(10) Würth, C.; Grabolle, M.; Pauli, J.; Spieles, M.; Resch-Genger, U. Relative and absolute determination of fluorescence quantum yields of transparent samples. *Nat Protoc* **2013,** *8* (8), 1535-1550.
(11) Dougherty, C. A.; Vaidyanathan, S.; Orr, B. G.; Banaszak Holl, M. M. Fluorophore:Dendrimer Ratio Impacts Cellular Uptake and Intracellular Fluorescence Lifetime. *Bioconjugate Chem.* **2015,** *26* (2), 304-315.
(12) Pracht, P.; Bohle, F.; Grimme, S. Automated exploration of the low-energy chemical space with fast quantum chemical methods. *Phys. Chem. Chem. Phys.* **2020,** *22* (14), 7169-7192.
(13) Bannwarth, C.; Ehlert, S.; Grimme, S. GFN2-xTB—An Accurate and Broadly Parametrized Self-Consistent Tight-Binding Quantum Chemical Method with Multipole Electrostatics and Density-Dependent Dispersion Contributions. *J. Chem.Theory Comput.* **2019,** *15* (3), 1652-1671.
(14) Neese, F. The ORCA program system. *WIREs Comput Mol Sci* **2012,** *2* (1), 73-78.
(15) Perdew, J. P. Density-functional approximation for the correlation energy of the inhomogeneous electron gas. *Phys. Rev. B* **1986,** *33* (12), 8822-8824.
(16) Becke, A. D. Density-functional exchange-energy approximation with correct asymptotic behavior. *Phys. Rev. A* **1988,** *38* (6), 3098-3100.
(17) Weigend, F.; Ahlrichs, R. Balanced basis sets of split valence, triple zeta valence and quadruple zeta valence quality for H to Rn: Design and assessment of accuracy. *Phys. Chem. Chem. Phys.* **2005,** *7* (18), 3297-3305.

(18) Grimme, S.; Antony, J.; Ehrlich, S.; Krieg, H. A consistent and accurate ab initio parametrization of density functional dispersion correction (DFT-D) for the 94 elements H-Pu. *J. Chem. Phys.* **2010,** *132* (15).
(19) Grimme, S.; Ehrlich, S.; Goerigk, L. Effect of the damping function in dispersion corrected density functional theory. *J. Comput. Chem.* **2011,** *32* (7), 1456-1465.
(20) Barone, V.; Cossi, M. Quantum Calculation of Molecular Energies and Energy Gradients in Solution by a Conductor Solvent Model. *J. Phys. Chem. A* **1998,** *102* (11), 1995-2001.
(21) Klamt, A.; Schüürmann, G. COSMO: a new approach to dielectric screening in solvents with explicit expressions for the screening energy and its gradient. *J. Chem. Soc., Perkin Trans. 2* **1993,** (5), 799-805.
(22) Helmich-Paris, B.; de Souza, B.; Neese, F.; Izsák, R. An improved chain of spheres for exchange algorithm. *J. Chem. Phys.* **2021,** *155* (10).
(23) Adamo, C.; Barone, V. Toward reliable density functional methods without adjustable parameters: The PBE0 model. *J. Chem. Phys.* **1999,** *110* (13), 6158-6170.
(24) Plasser, F. TheoDORE: A toolbox for a detailed and automated analysis of electronic excited state computations. *The Journal of Chemical Physics* **2020,** *152* (8).
(25) Trasatti, S. The absolute electrode potential: an explanatory note (Recommendations 1986). *Pure Appl. Chem.* **1986,** *58* (7), 955-966.
(26) Rehm, D.; Weller, A. Kinetics of Fluorescence Quenching by Electron and H-Atom Transfer. *Isr. J. Chem.* **1970,** *8* (2), 259-271.
(27) Klán, P.; Wirz, J., Photochemistry of organic compounds: From concepts to practice. In *Photochemistry of organic compounds: From concepts to practice.* [Online] 2009.
(28) Bertie, J. E.; Lan, Z. Liquid Water−Acetonitrile Mixtures at 25 °C: The Hydrogen-Bonded Structure Studied through Infrared Absolute Integrated Absorption Intensities. *The Journal of Physical Chemistry B* **1997,** *101* (20), 4111-4119.
(29) Siegl, S. J.; Galeta, J.; Dzijak, R.; Dračínský, M.; Vrabel, M. Bioorthogonal Fluorescence Turn-On Labeling Based on Bicyclononyne-Tetrazine Cycloaddition Reactions that Form Pyridazine Products. *Chempluschem* **2019,** *84* (5), 493-497.
(30) Albitz, E.; Kern, D.; Kormos, A.; Bojtár, M.; Török, G.; Biró, A.; Szatmári, Á.; Németh, K.; Kele, P. Bioorthogonal Ligation-Activated Fluorogenic FRET Dyads. *Angew. Chem. Int. Ed.* **2022,** *61* (6), e202111855.
(31) Arsić, A.; Hagemann, C.; Stajković, N.; Schubert, T.; Nikić-Spiegel, I. Minimal genetically encoded tags for fluorescent protein labeling in living neurons. *Nat Commun* **2022,** *13* (1), 314.
