# Supplementary material for: Fluorescence Quenching Properties and Bioimaging Applications of Readily Accessible Blue to Far-Red Fluorogenic Triazinium Salts
Source: J Am Chem Soc. 2025 Dec 28;148(1):1183–96. doi: 10.1021/jacs.5c17428 (PMC12814330; doi:10.1021/jacs.5c17428)

PipTrz1 ^1H NMR (400 MHz, $CDCl_3$)

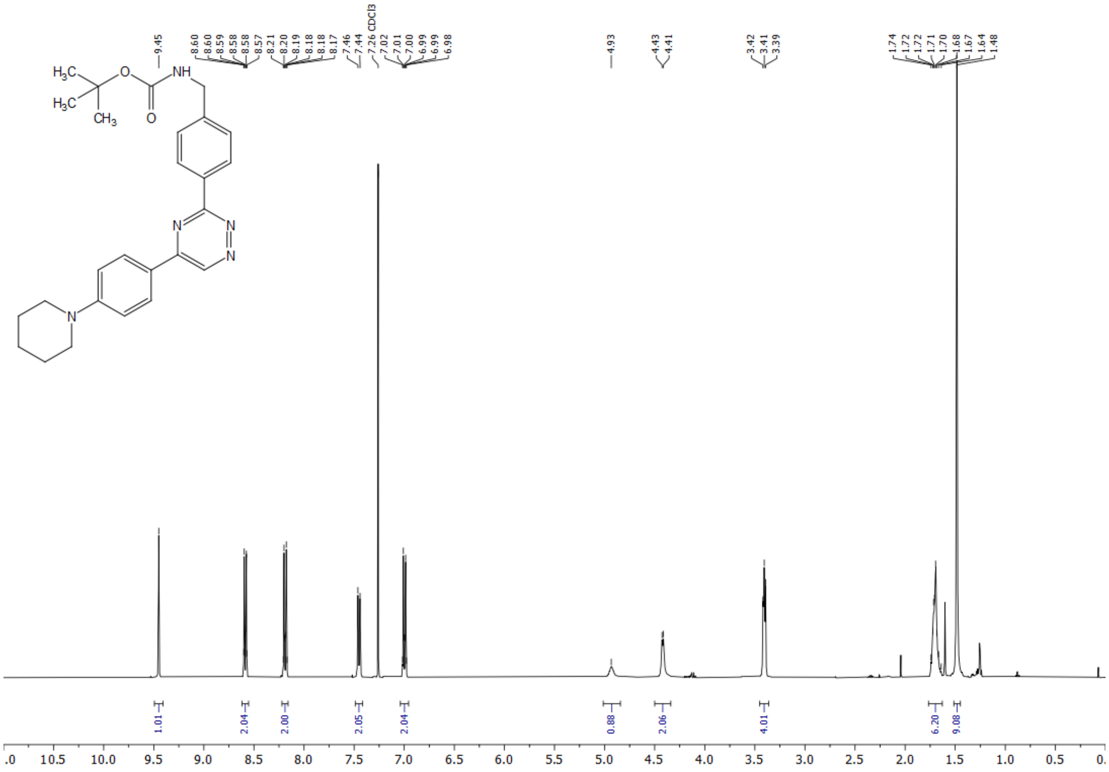

PipTrz1 ^{13}C NMR (101 MHz, $CDCl_3$)

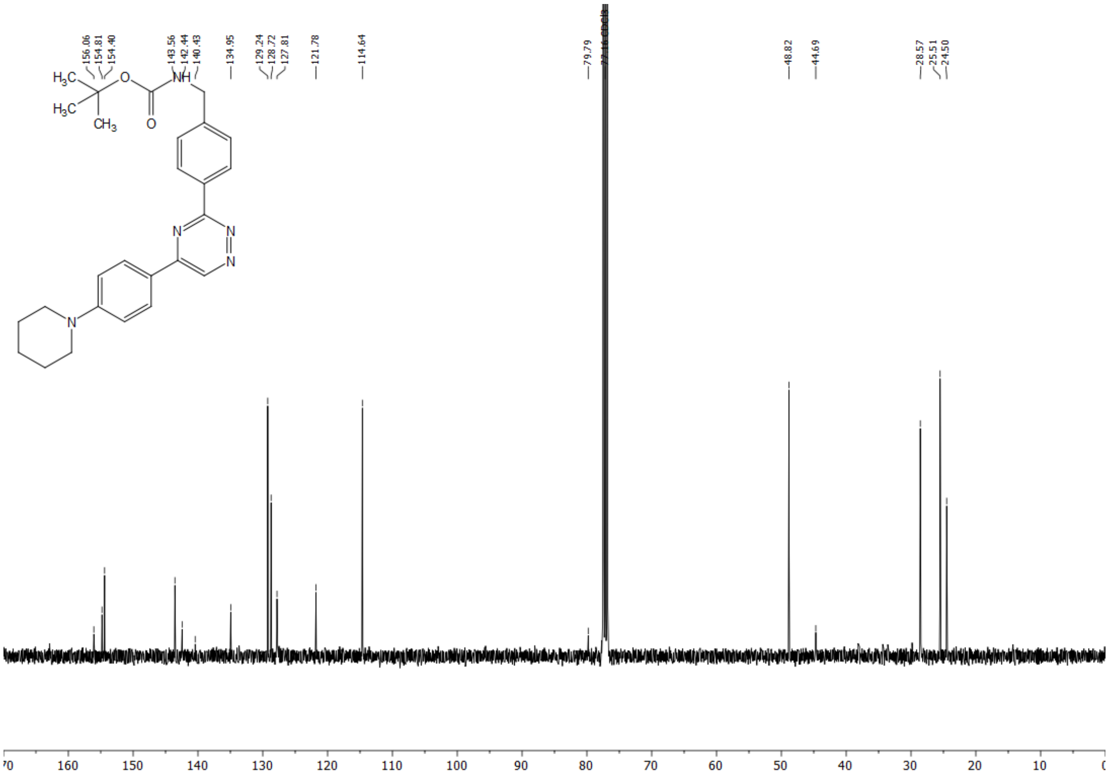

Trz⁺1 ^{1}H NMR (400 MHz, CD_3CN)

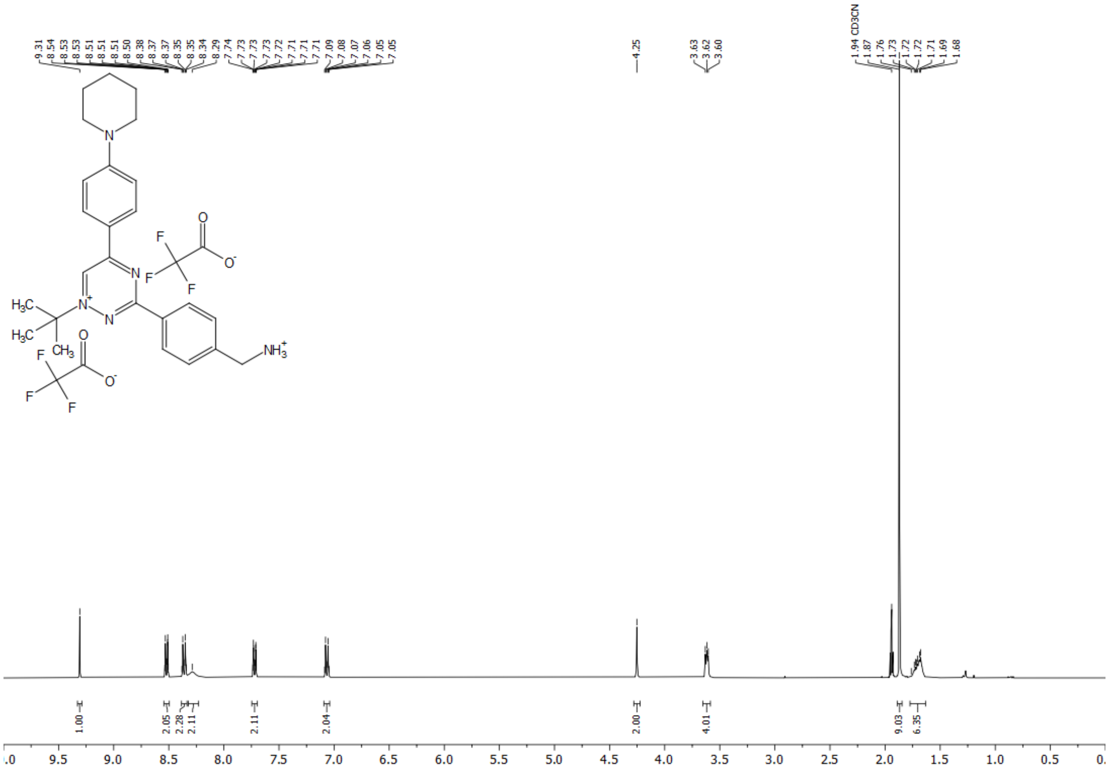

Trz⁺1 ^{13}C NMR (101 MHz, CD_3CN)

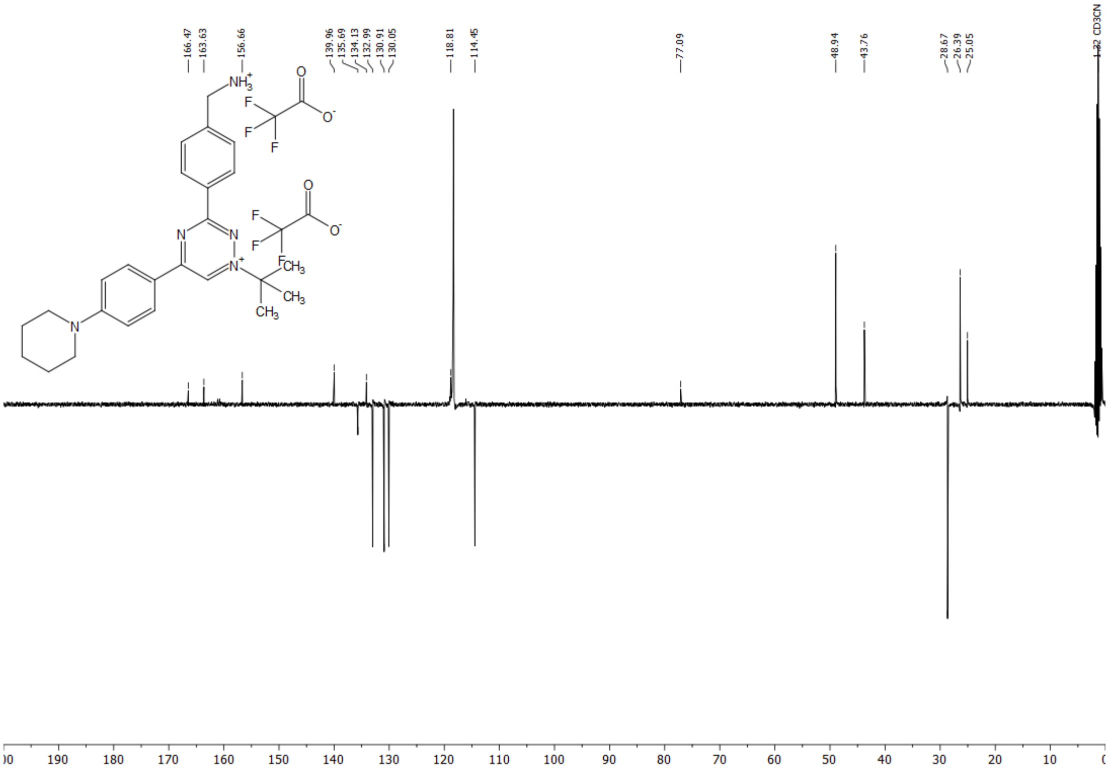

PipSMeTrz+ ^{1}H NMR (400 MHz, CD_3CN)

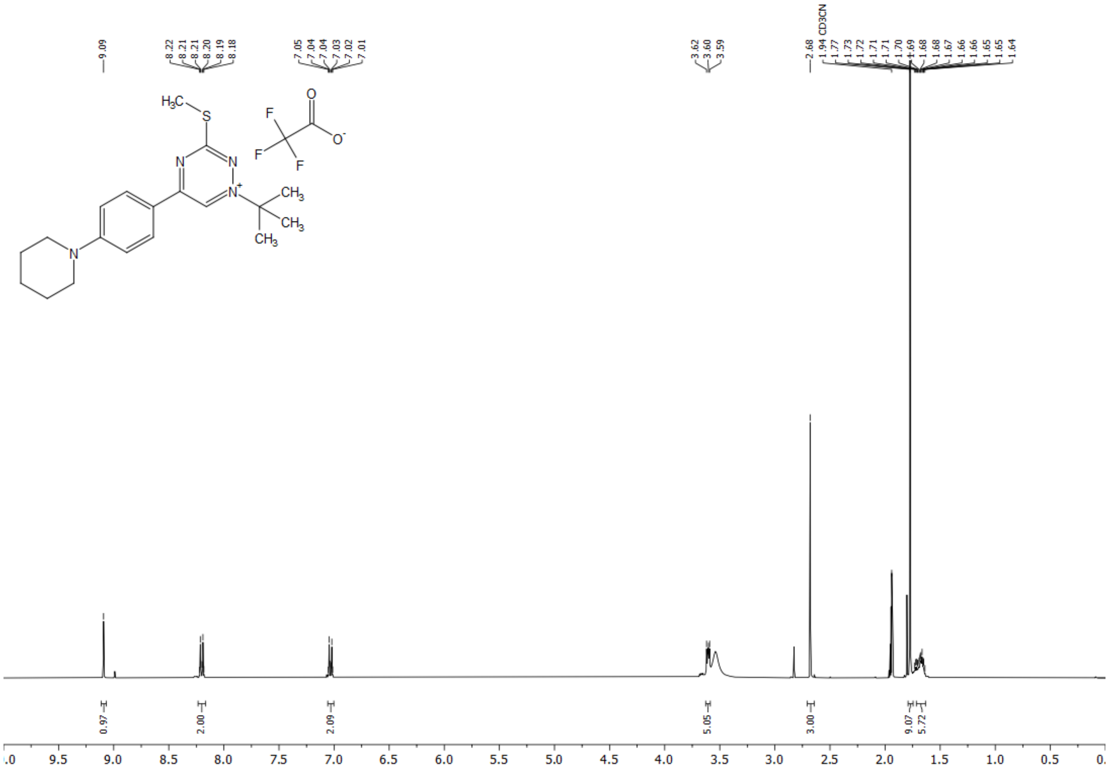

PipSMeTrz+ ^{19}F NMR (376 MHz, CD_3CN)

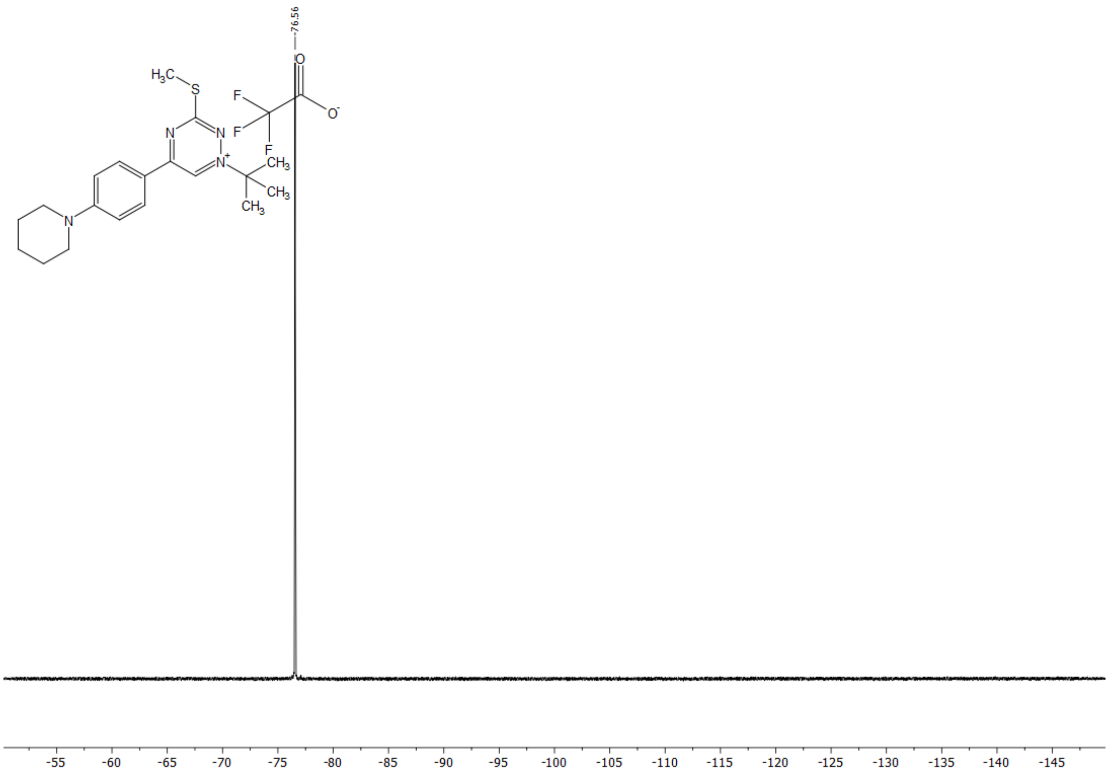

PipSMeTrz^{+} ^{13}C NMR (101 MHz, CD_3CN)

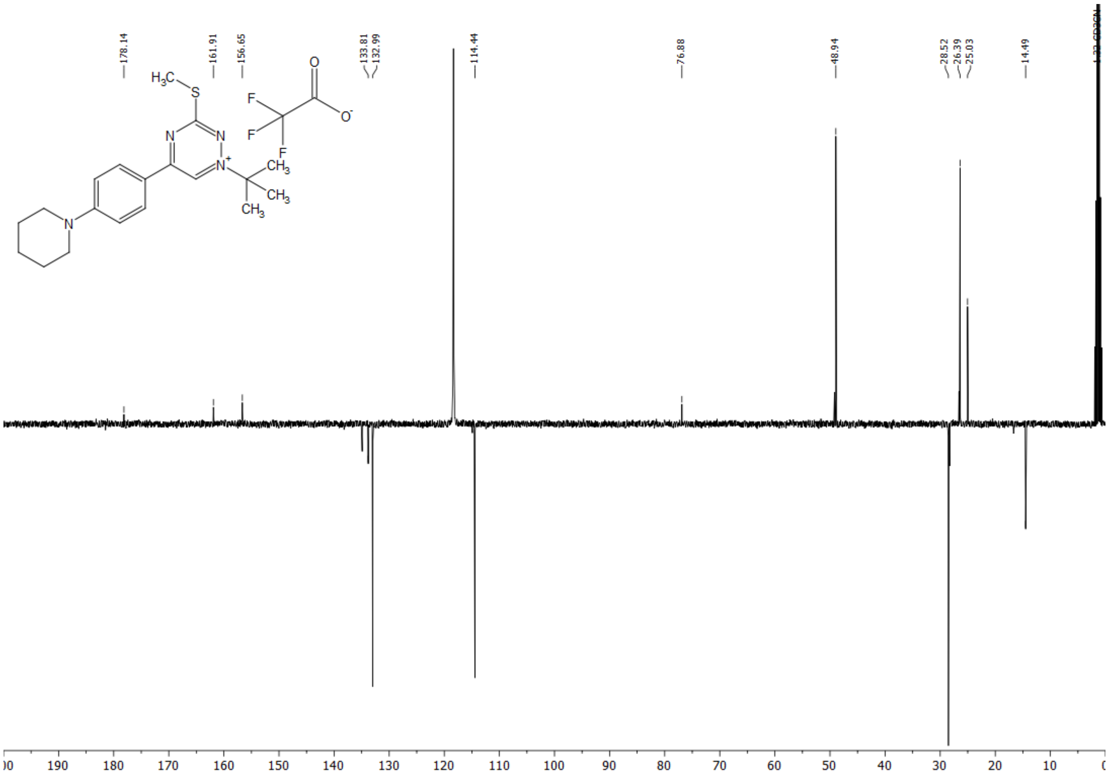

Trz^{+}Coum ^{1}H NMR (400 MHz, CD_3CN)

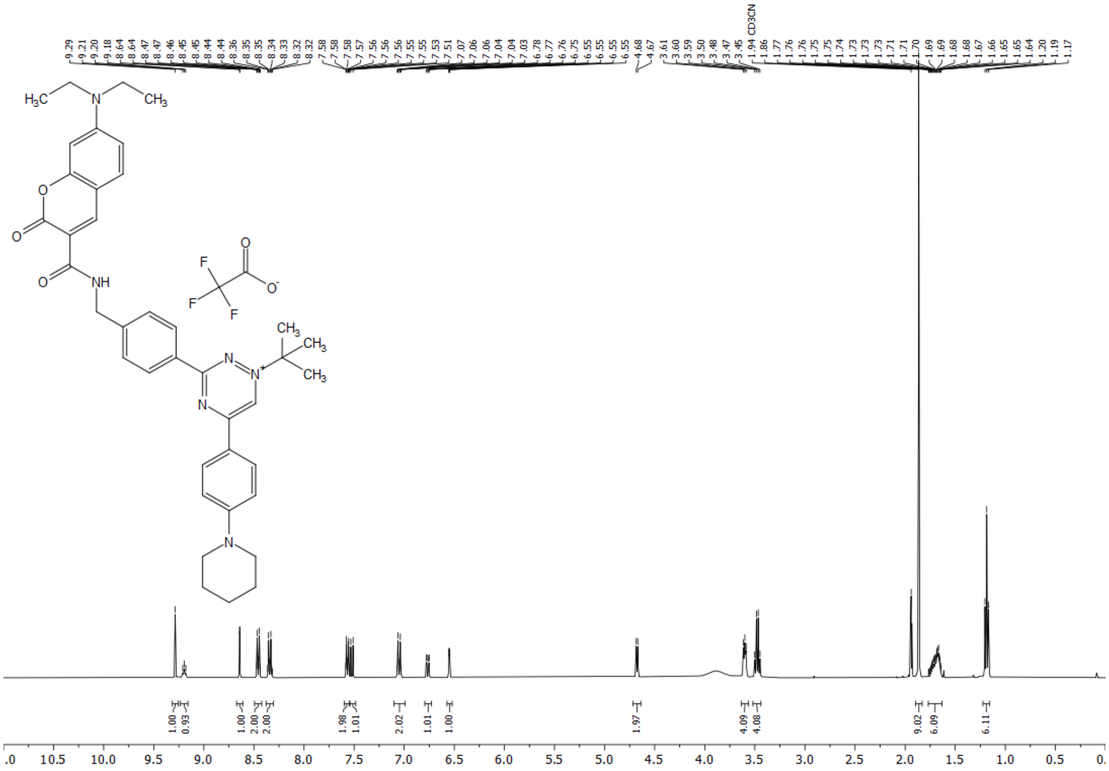

Trz$^+$Coum ^{19}F NMR (376 MHz, CD_3CN)

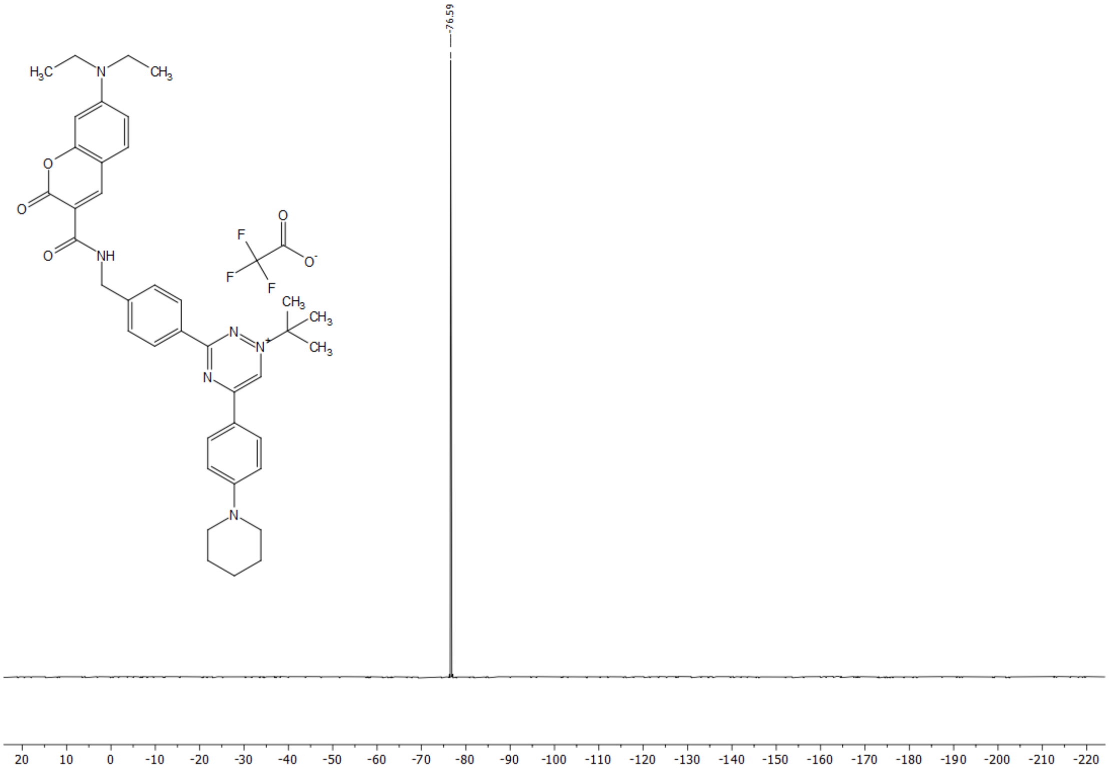

Trz$^+$Coum ^{13}C NMR (101 MHz, CD_3CN)

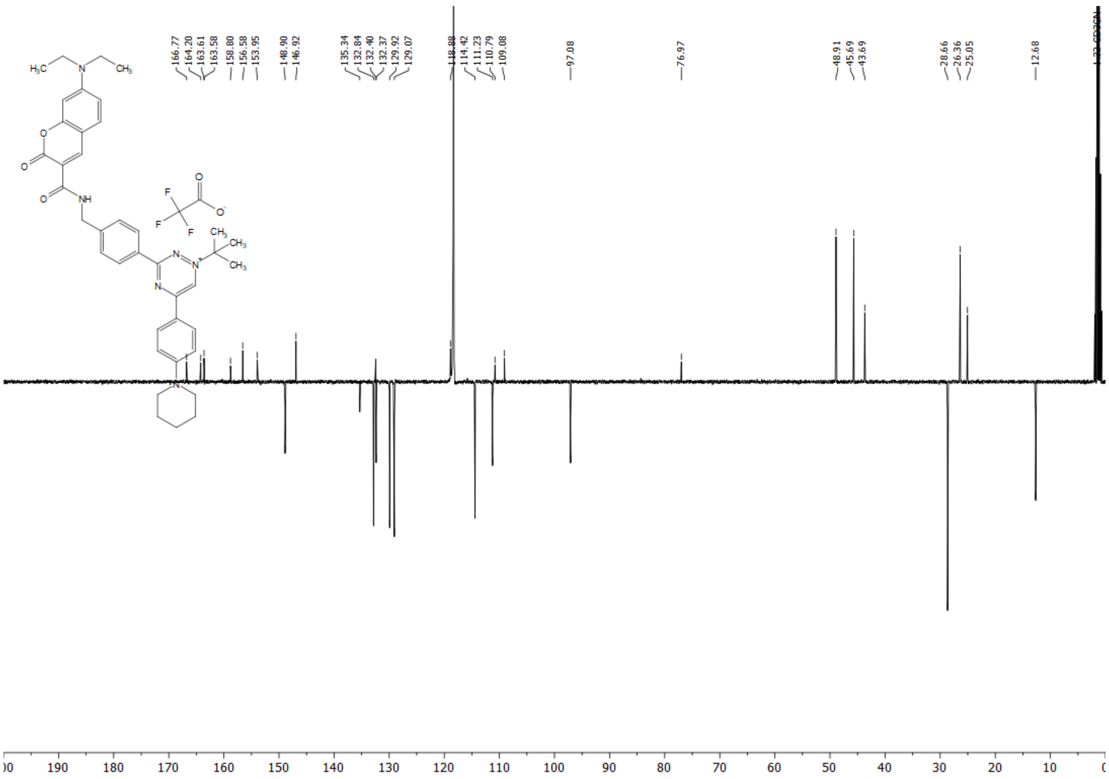

Trz⁺BODIPY ^{1}H NMR (500 MHz, DMSO-d_6)

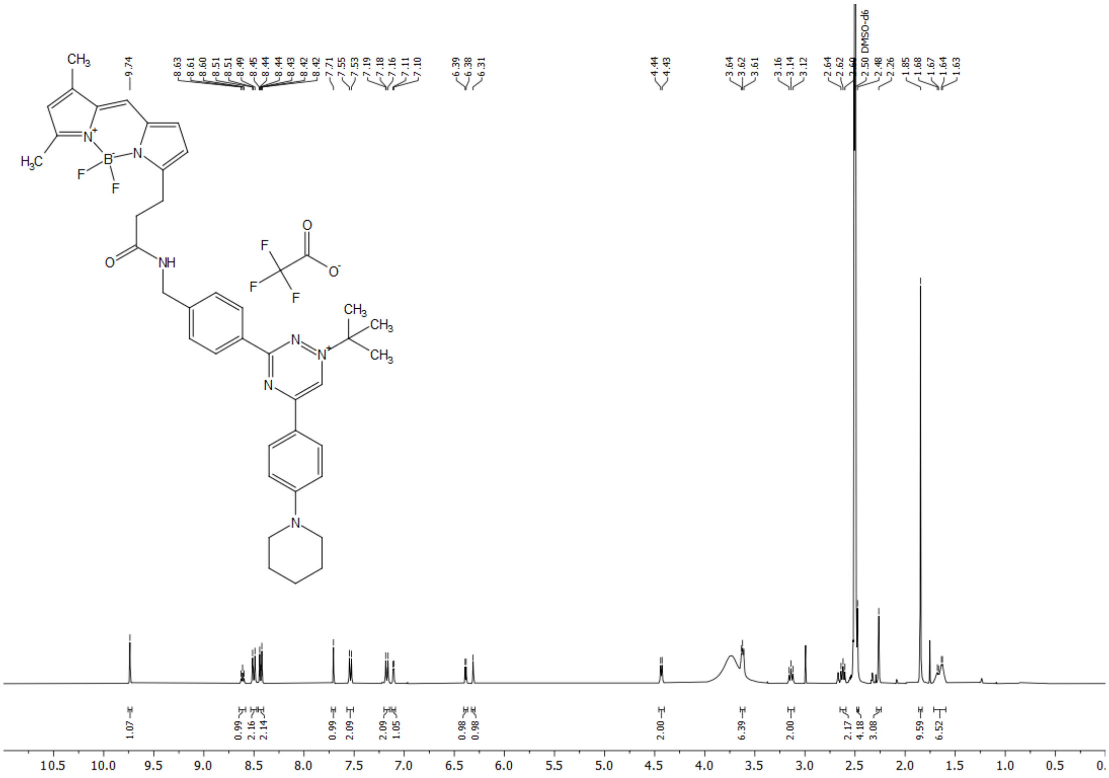

Trz⁺BODIPY ^{19}F NMR (470 MHz, DMSO-d_6)

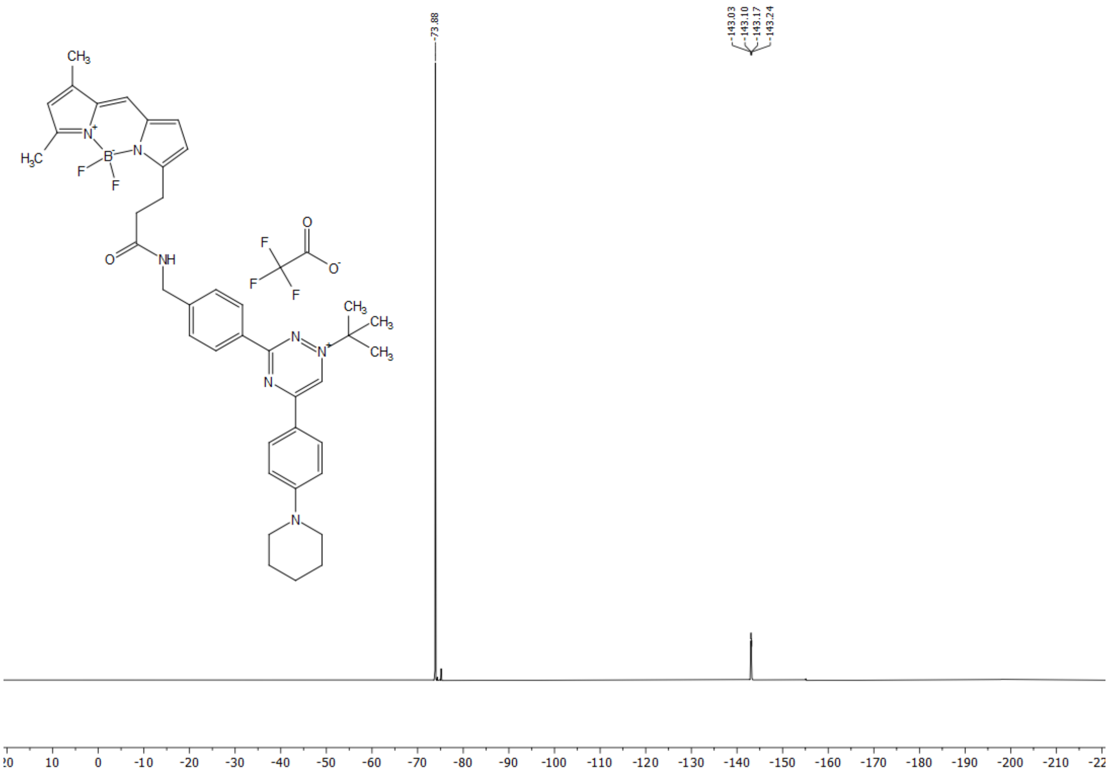

Trz⁺BODIPY ^{13}C NMR (126 MHz, DMSO-d_6)

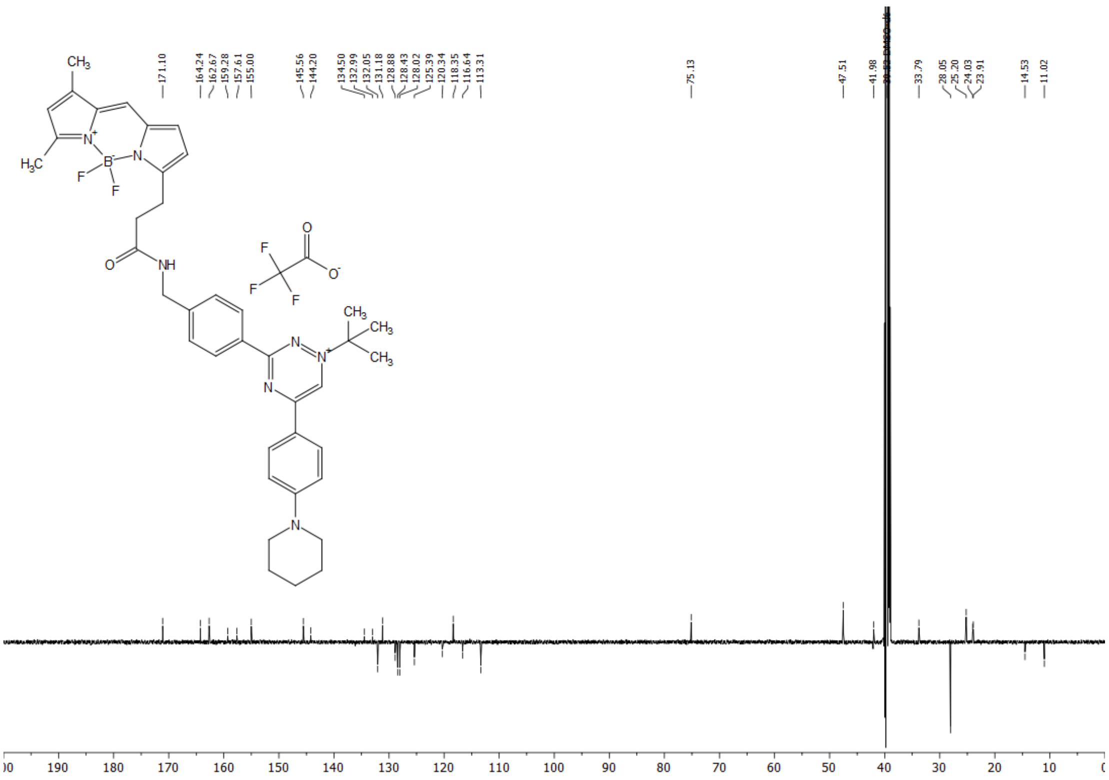

Trz⁺OG ^{1}H NMR (500 MHz, DMSO-d_6)

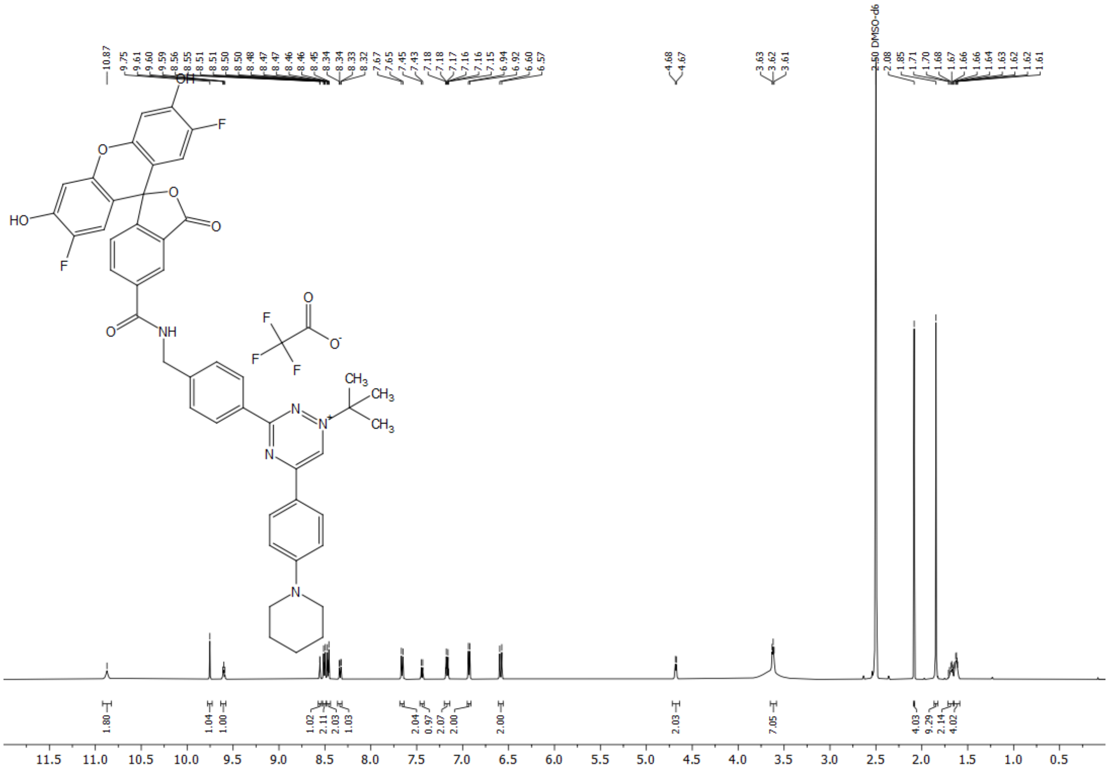

Trz^{+}OG ^{19}F NMR (470 MHz, DMSO-d_6)

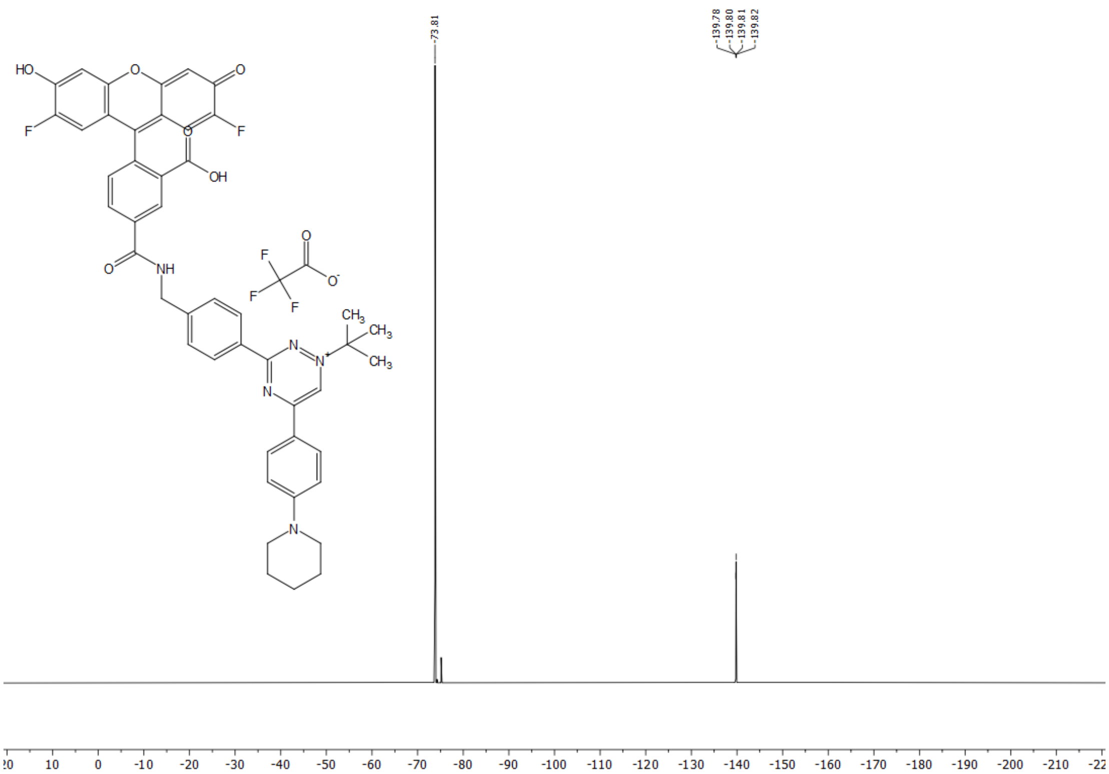

Trz^{+}OG ^{13}C NMR (126 MHz, DMSO-d_6)

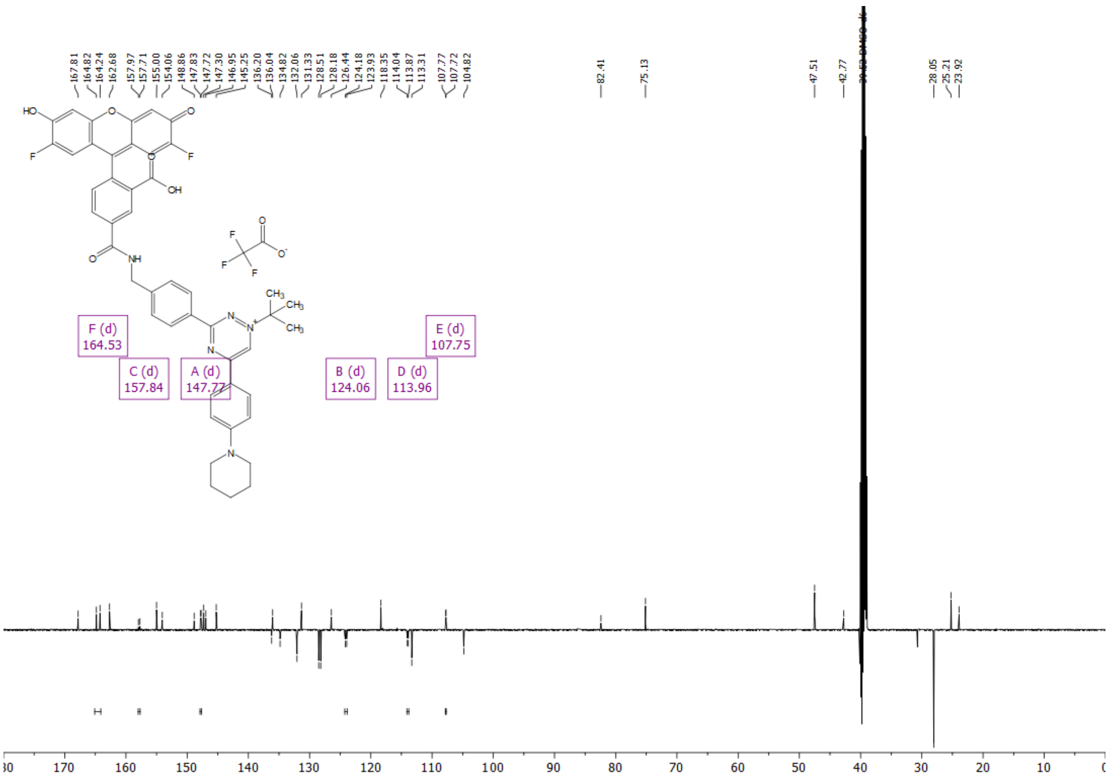

Trz⁺ATTO495 ^{1}H NMR (500 MHz, CD_3CN)

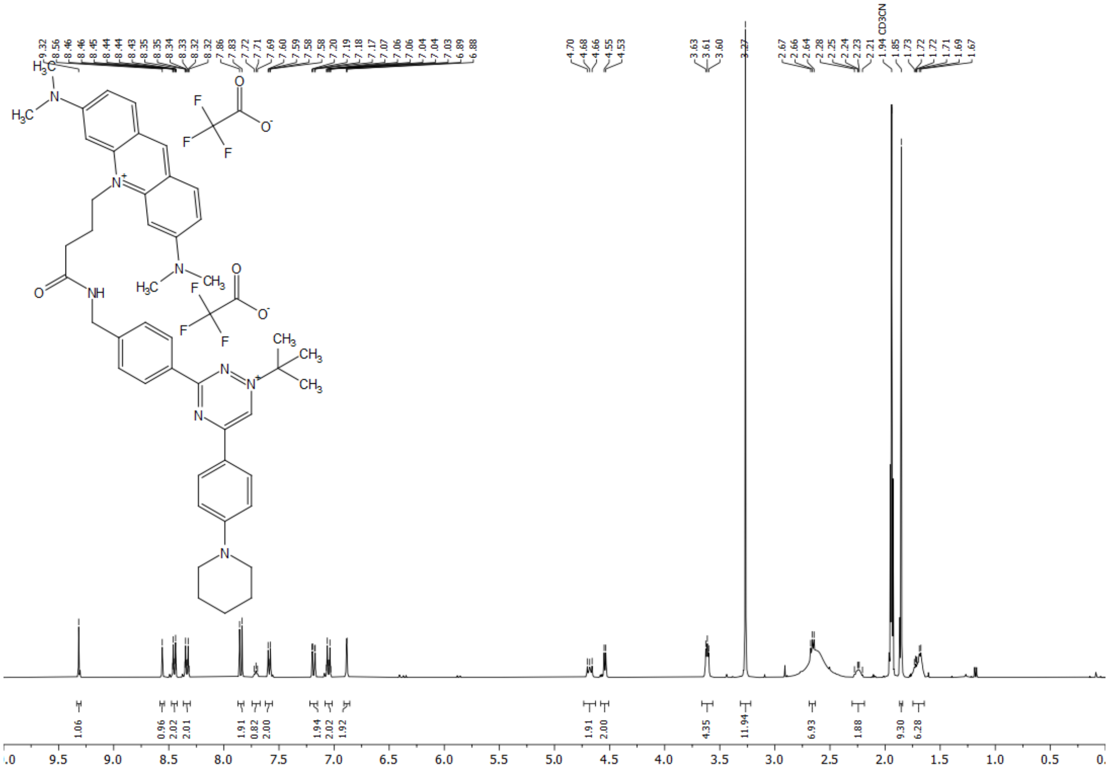

Trz⁺ATTO495 ^{19}F NMR (470 MHz, CD_3CN)

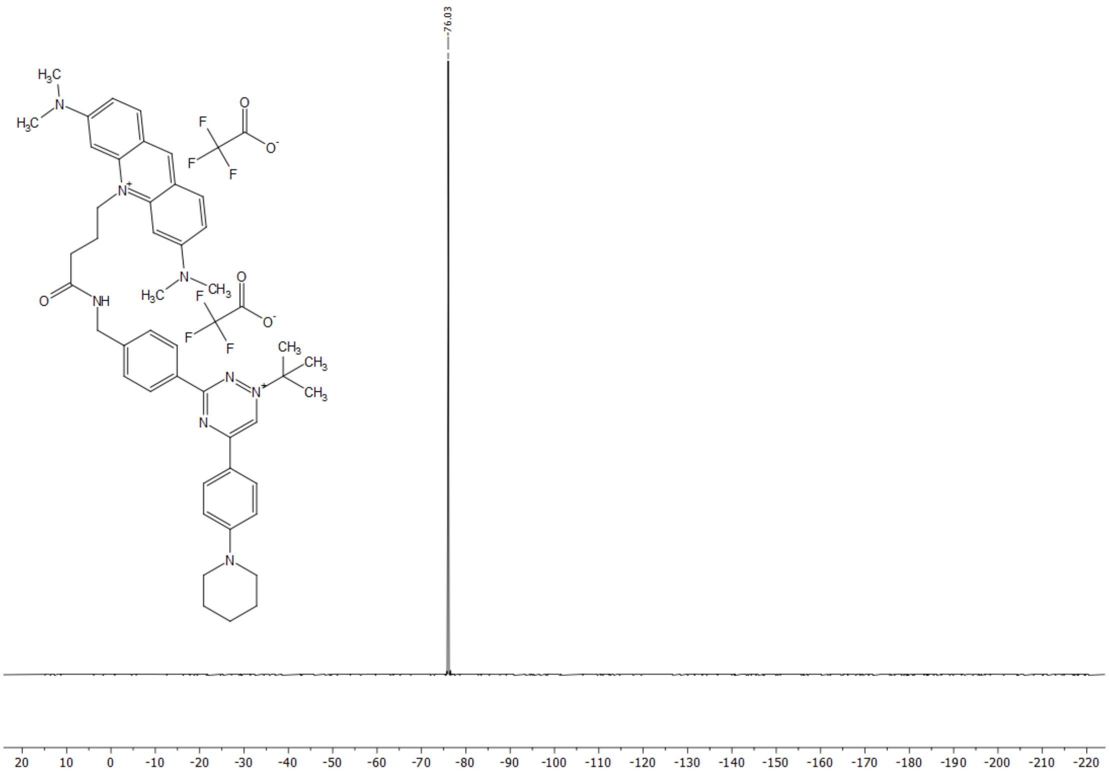

Trz⁺ATTO495 ^{13}C NMR (126 MHz, CD_3CN)

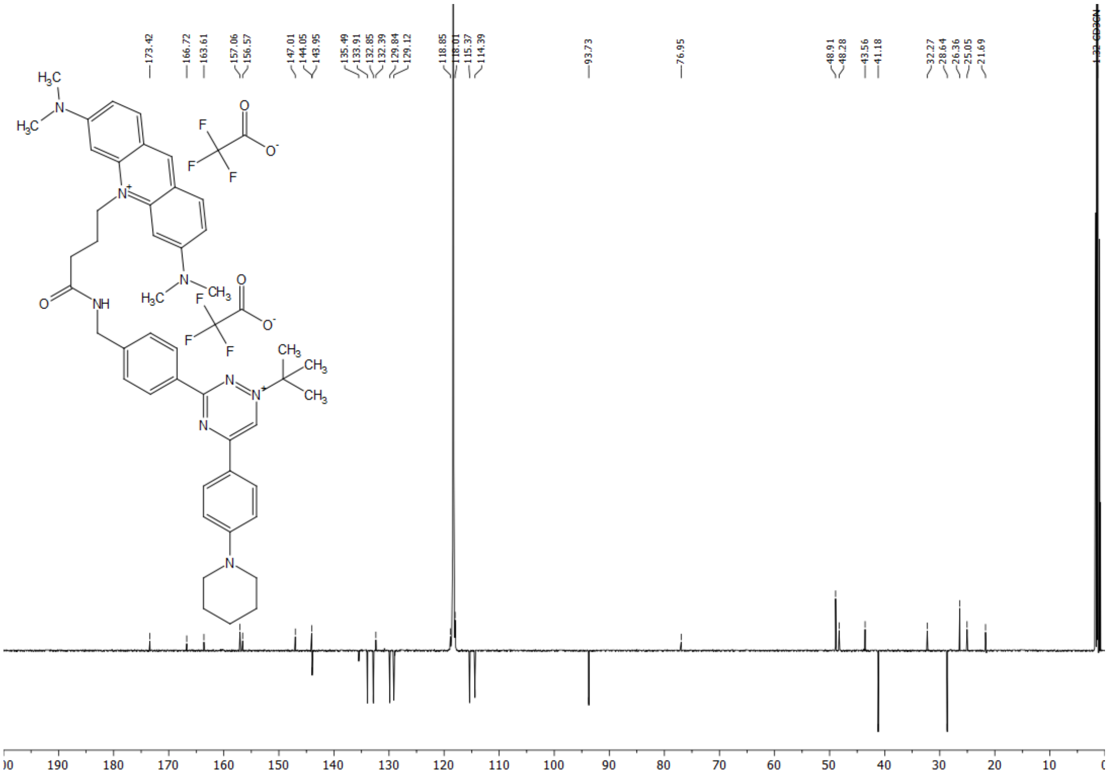

Trz⁺Cy3 ^{1}H NMR (500 MHz, DMSO-d_6)

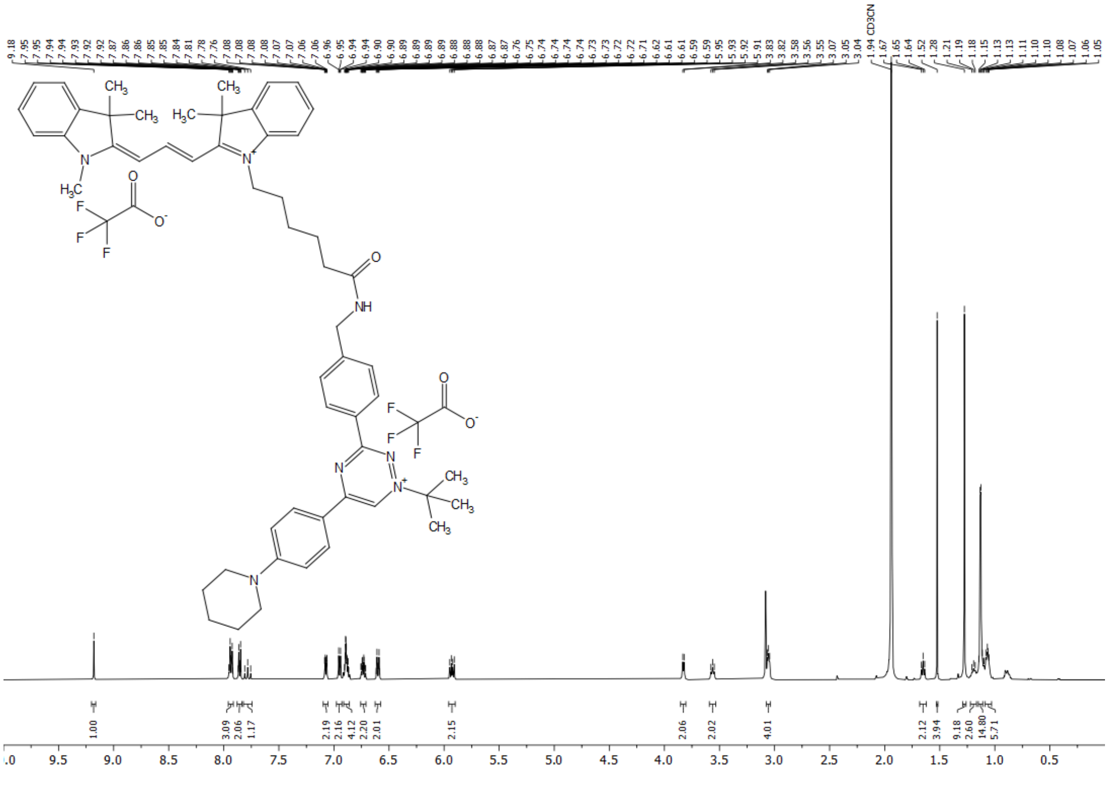

Trz⁺Cy3 ^{19}F NMR (470 MHz, DMSO-*d$_6$*)

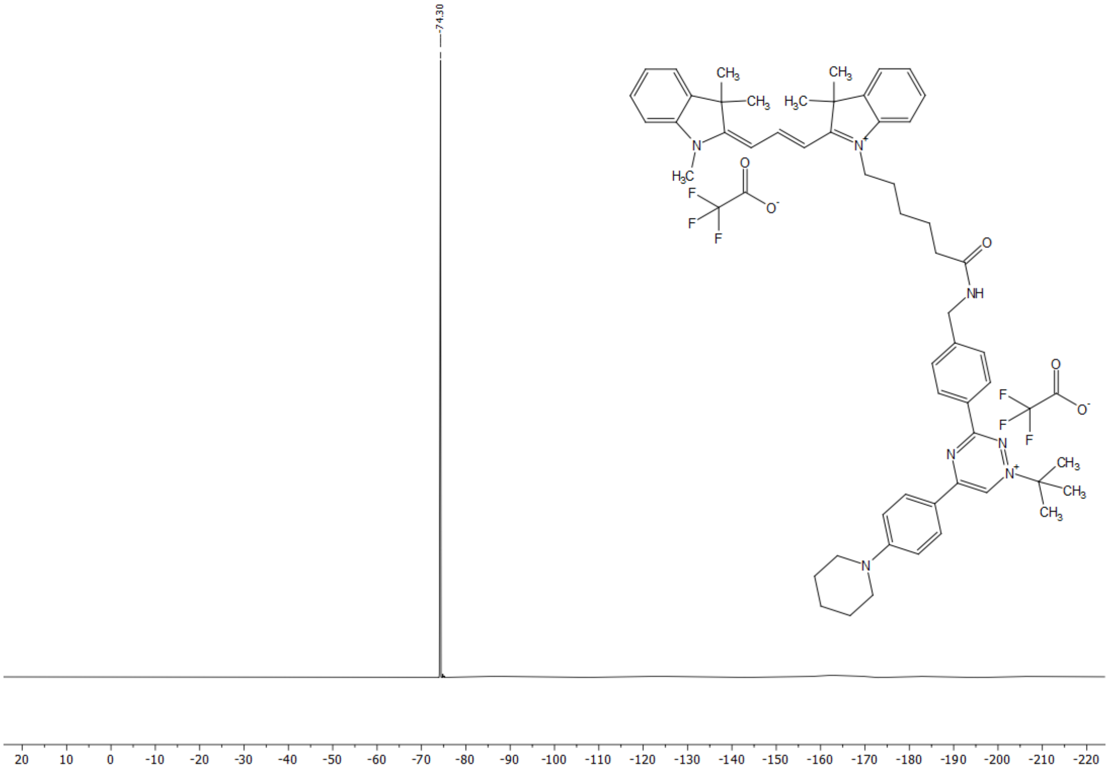

Trz⁺Cy3 ^{13}C NMR (126 MHz, DMSO-*d$_6$*)

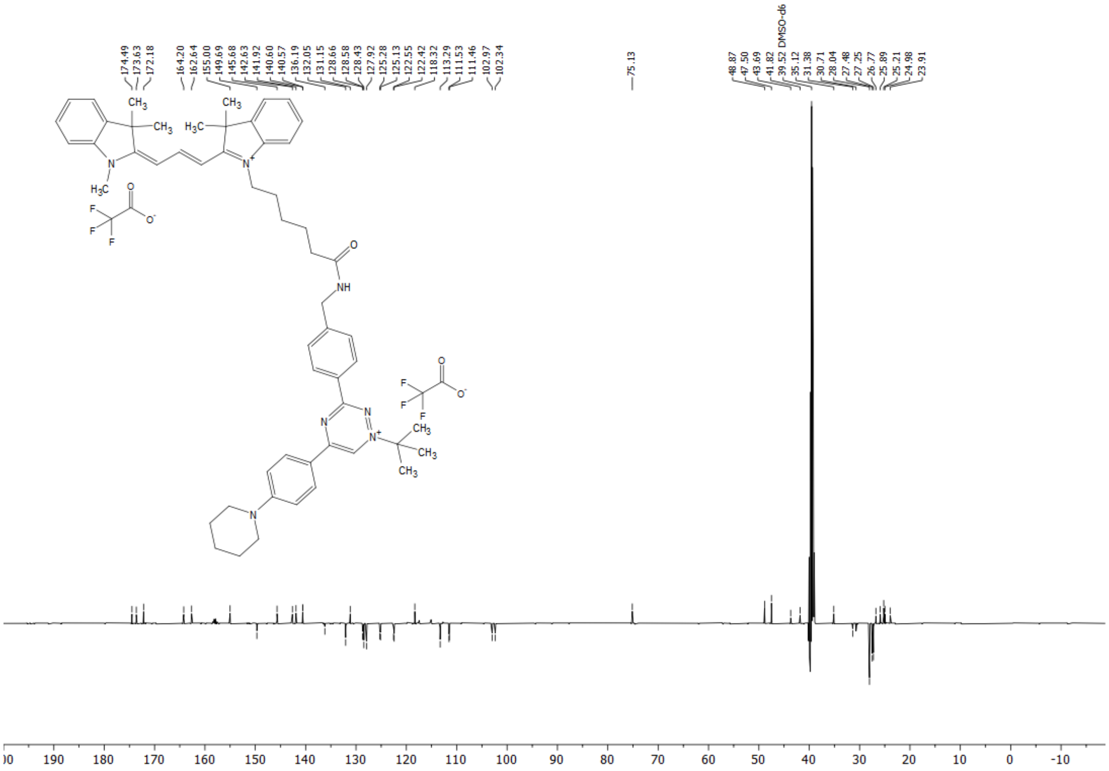

Trz⁺SulfoCy3 ¹H NMR (500 MHz, DMSO-d_6)

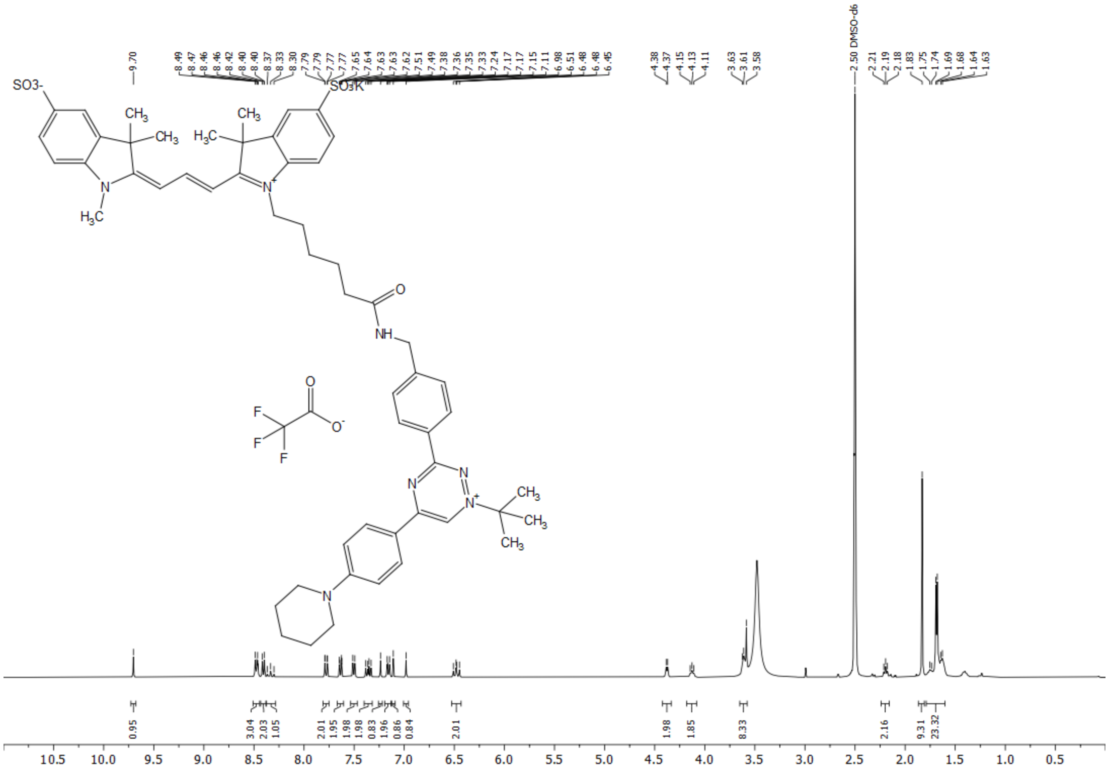

Trz⁺SulfoCy3 ¹³C NMR (126 MHz, DMSO-d_6)

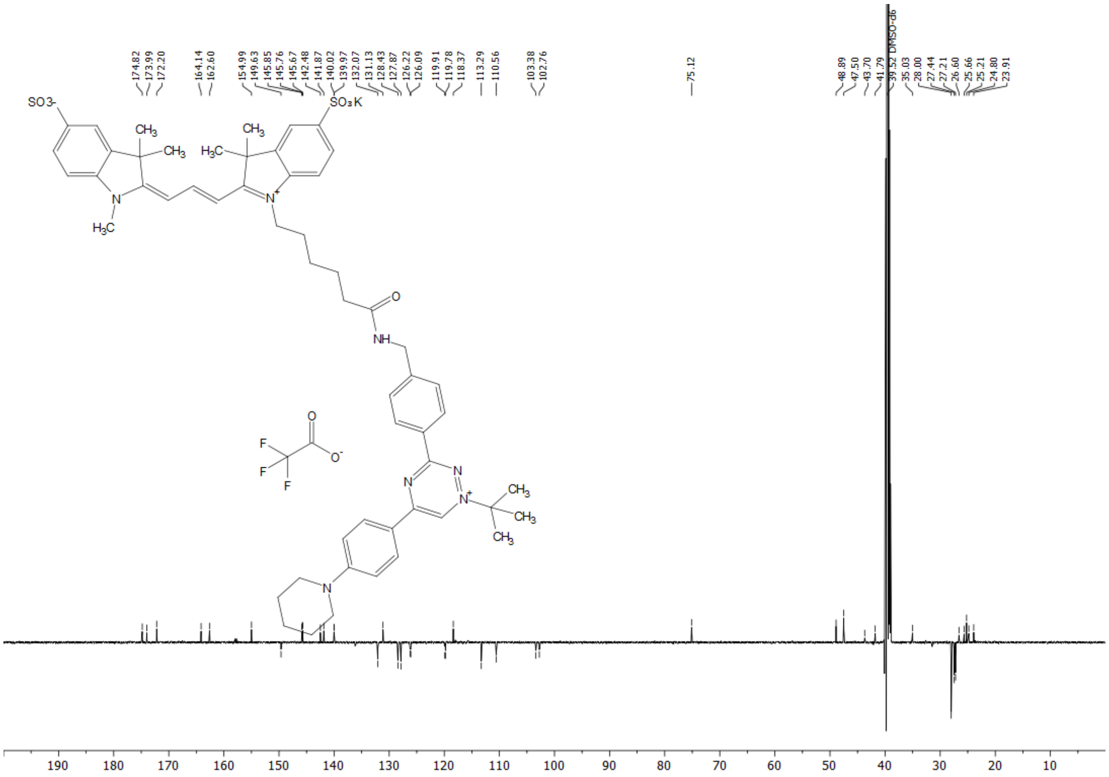

Trz⁺TAMRA ¹H NMR (500 MHz, DMSO-d_6)

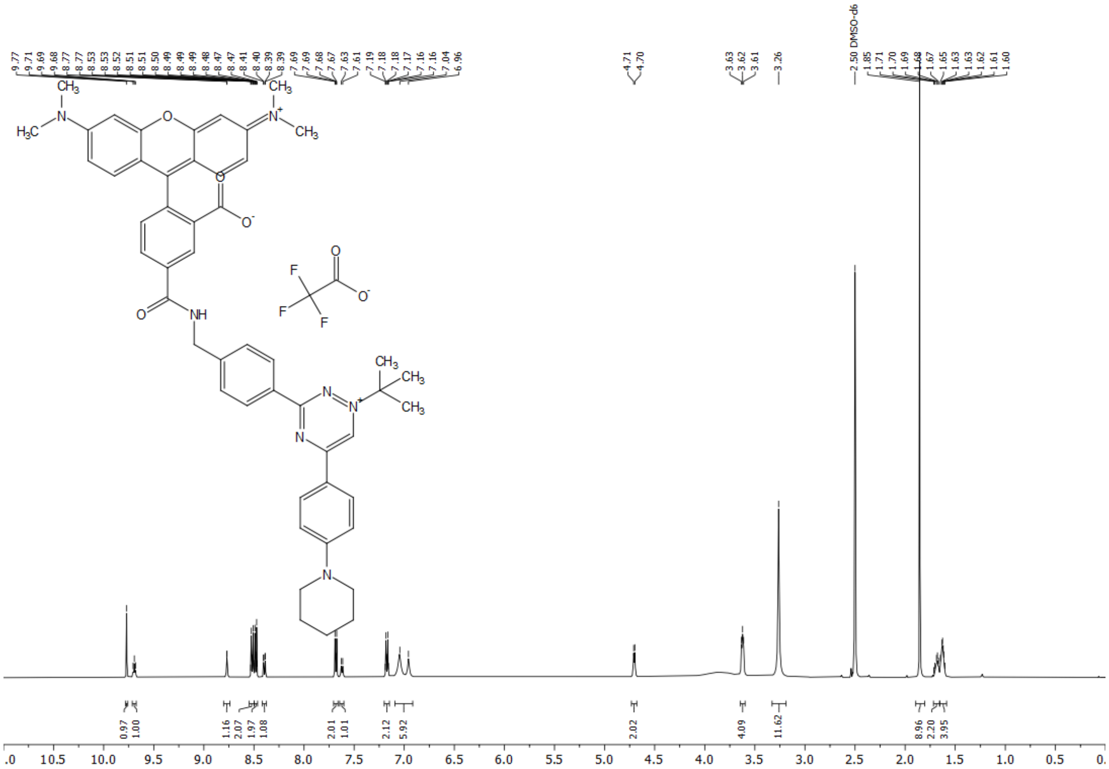

Trz⁺TAMRA ¹⁹F NMR (470 MHz, DMSO-d_6)

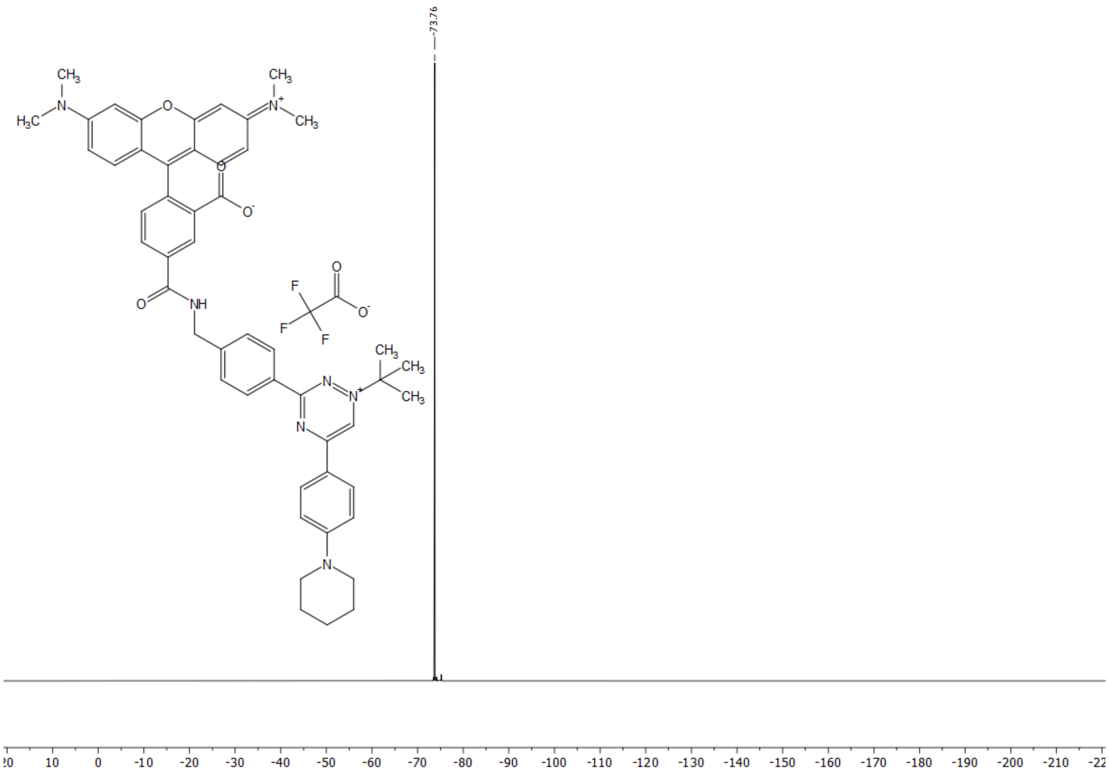

Trz⁺TAMRA ^{13}C NMR (126 MHz, DMSO-d_6)

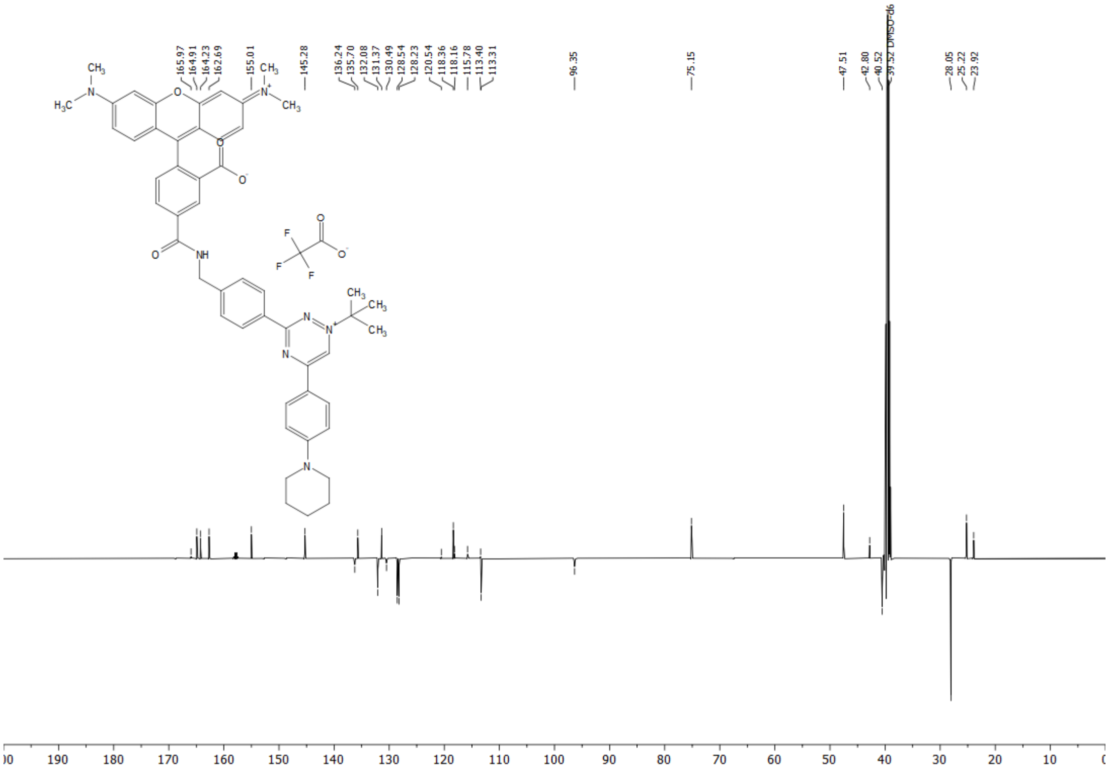

Trz⁺SiRho ^{1}H NMR (500 MHz, DMSO-d_6)

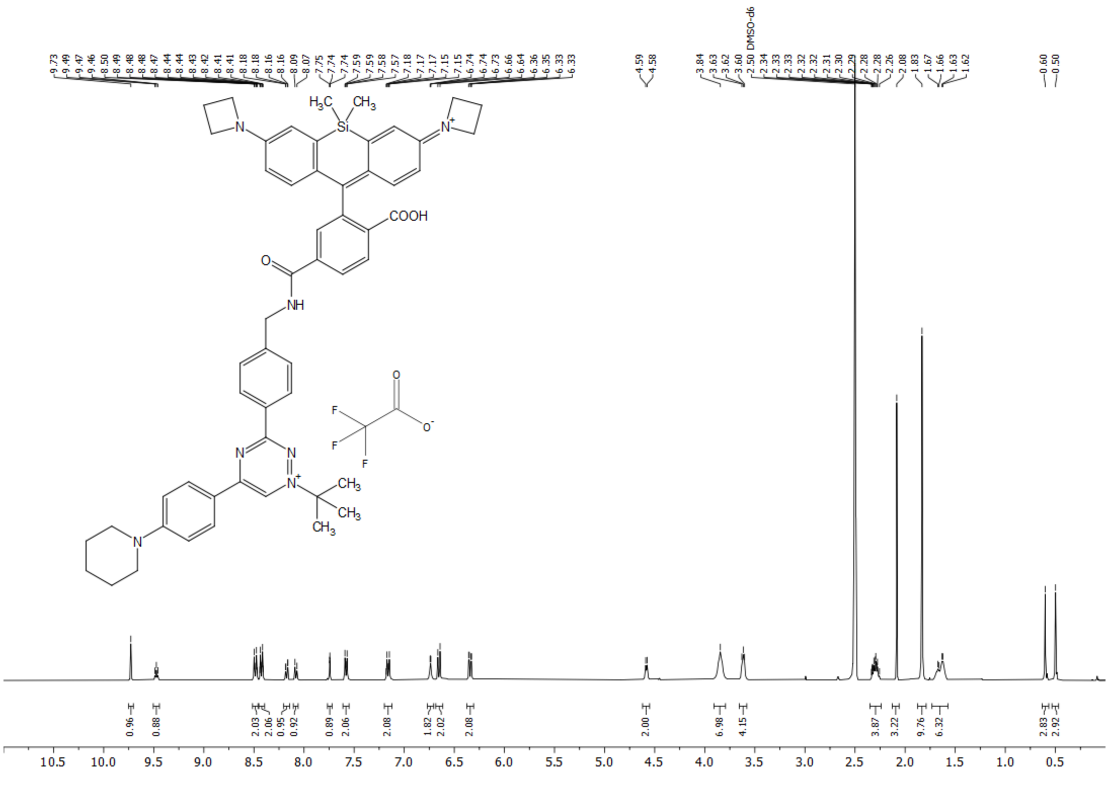

Trz^{+}SiRho ^{19}F NMR (470 MHz, DMSO-d_6)

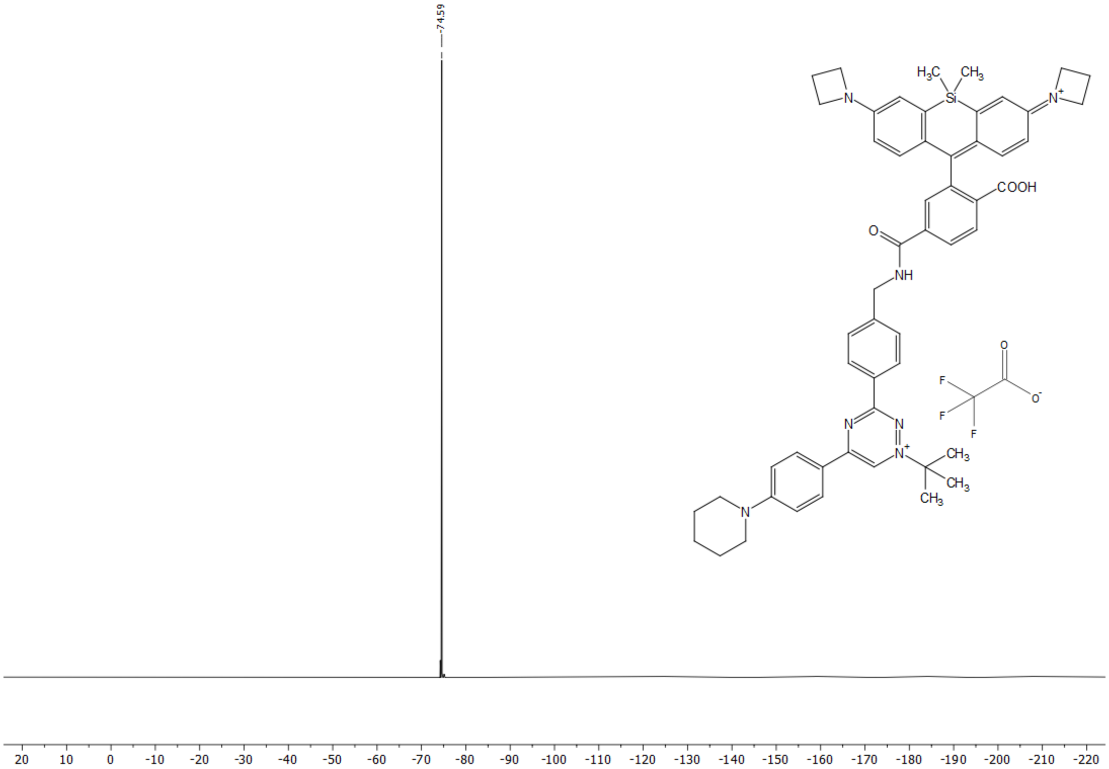

Trz^{+}SiRho ^{13}C NMR (126 MHz, DMSO-d_6)

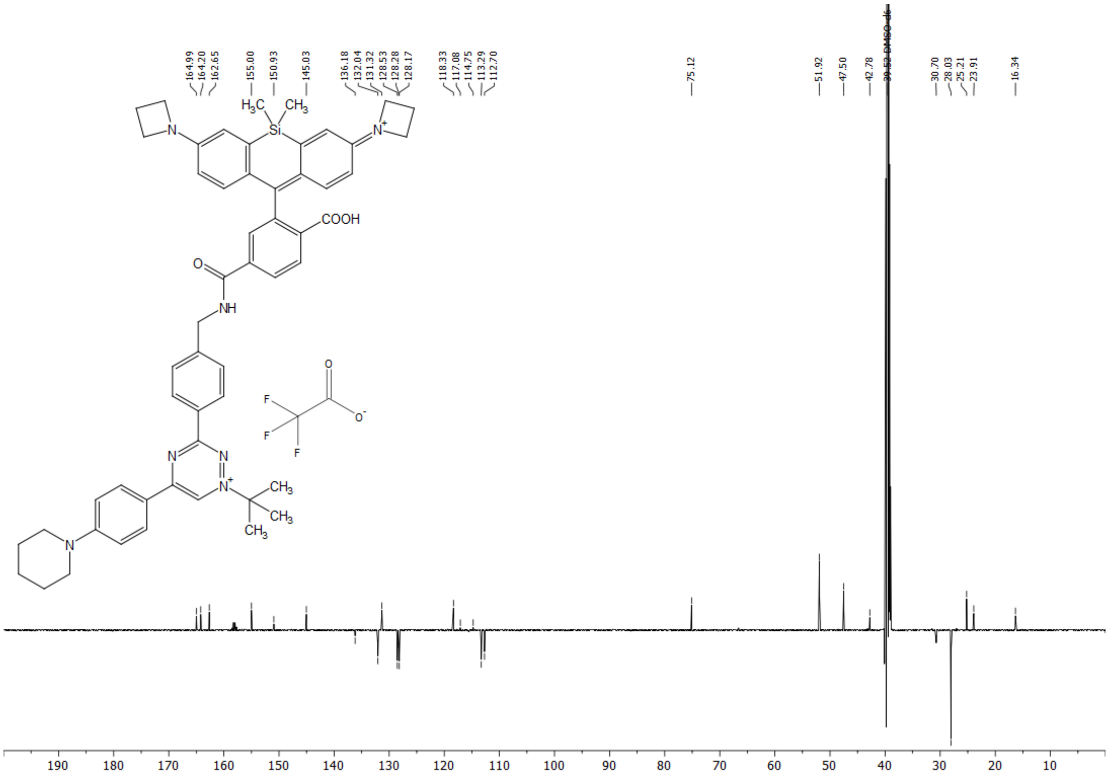

SMeTrz3 ^{1}H NMR (400 MHz, $CDCl_3$)

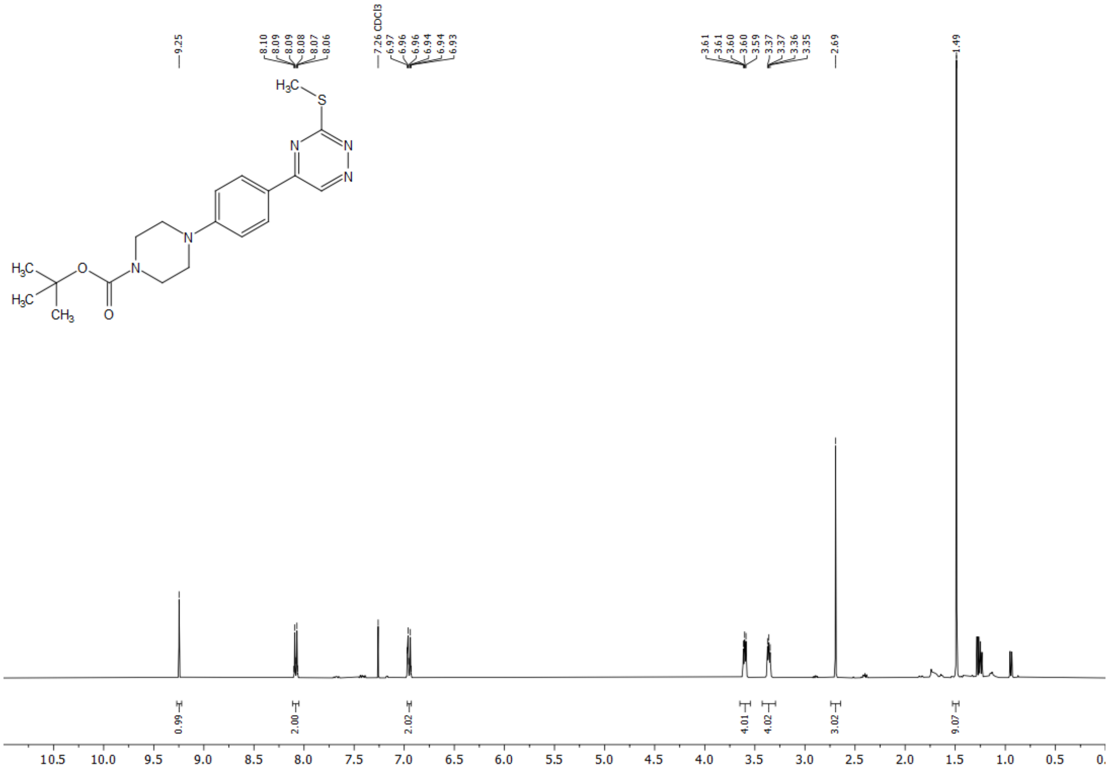

SMeTrz3 ^{13}C NMR (101 MHz, $CDCl_3$)

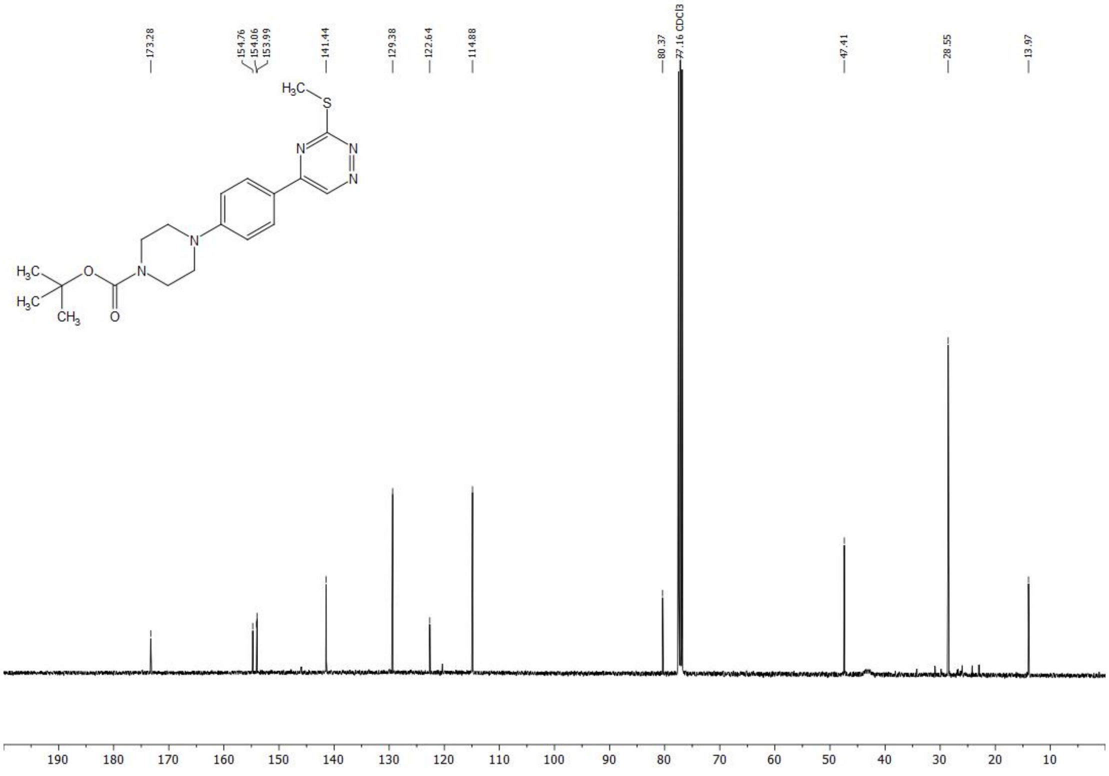

SMeTrz4 ^1H NMR (400 MHz, MeOH-d_4)

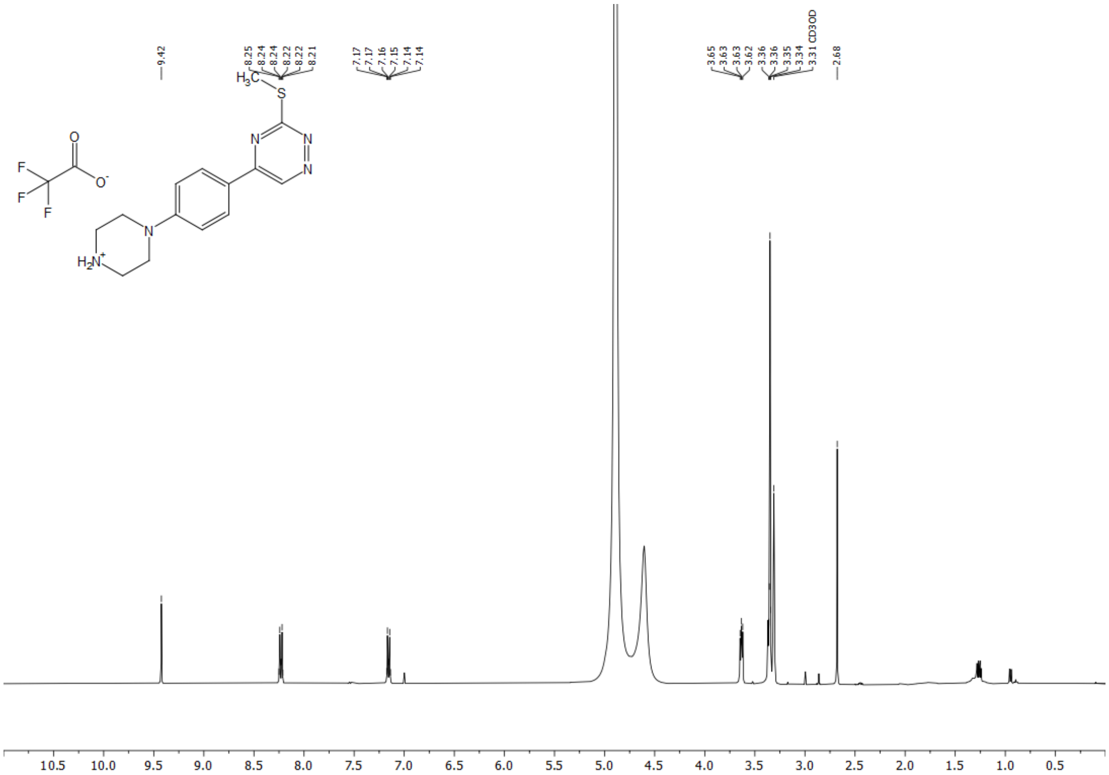

SMeTrz5 ^1H NMR (400 MHz, DMSO-d_6)

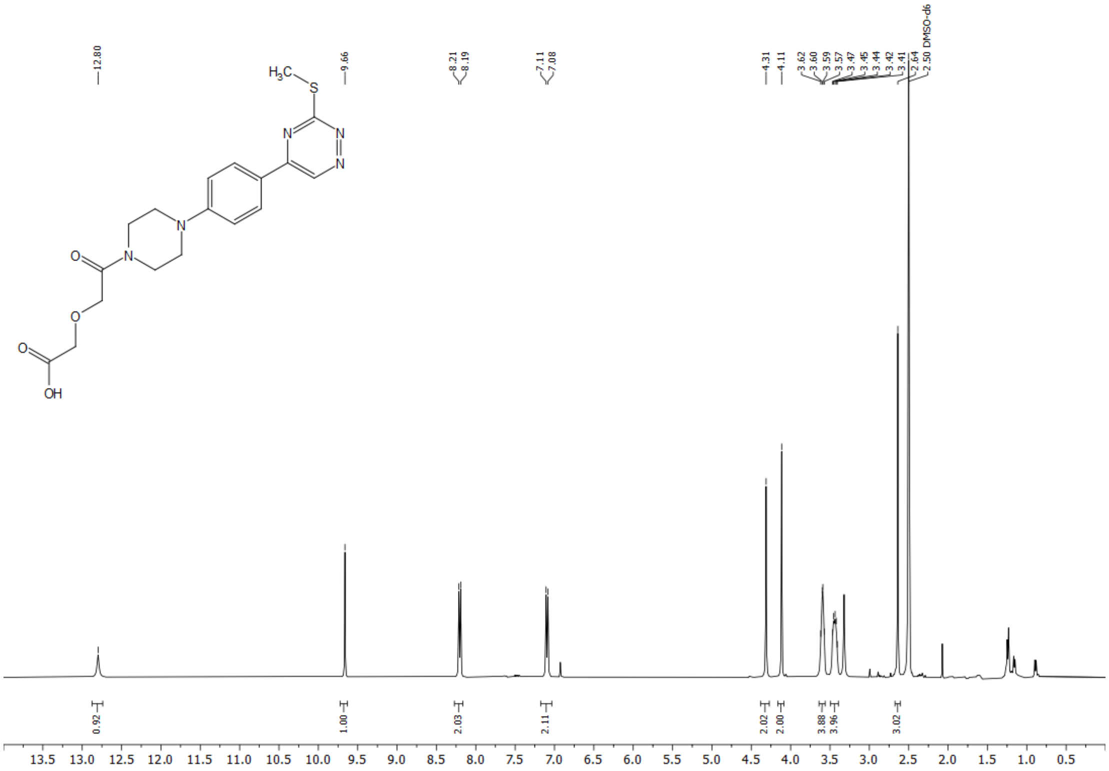

SMeTrz5 ^{13}C NMR (101 MHz, DMSO-d_6)

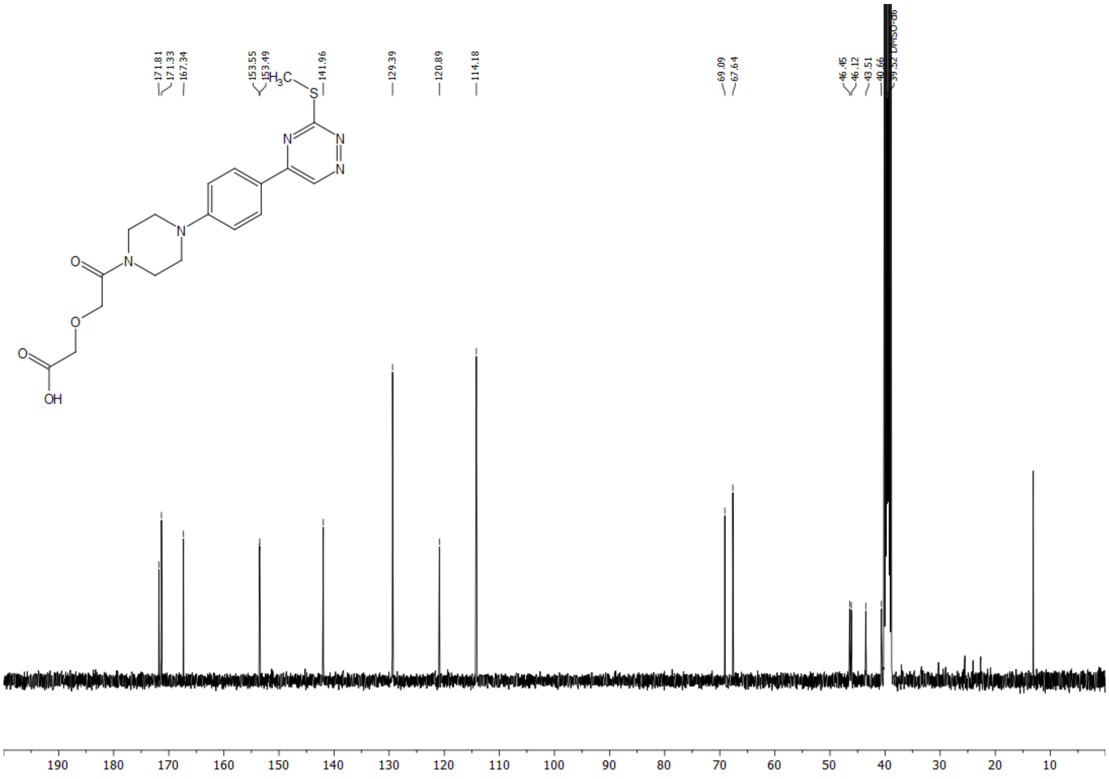

SMeTrz6 ^{1}H NMR (400 MHz, $CDCl_3$)

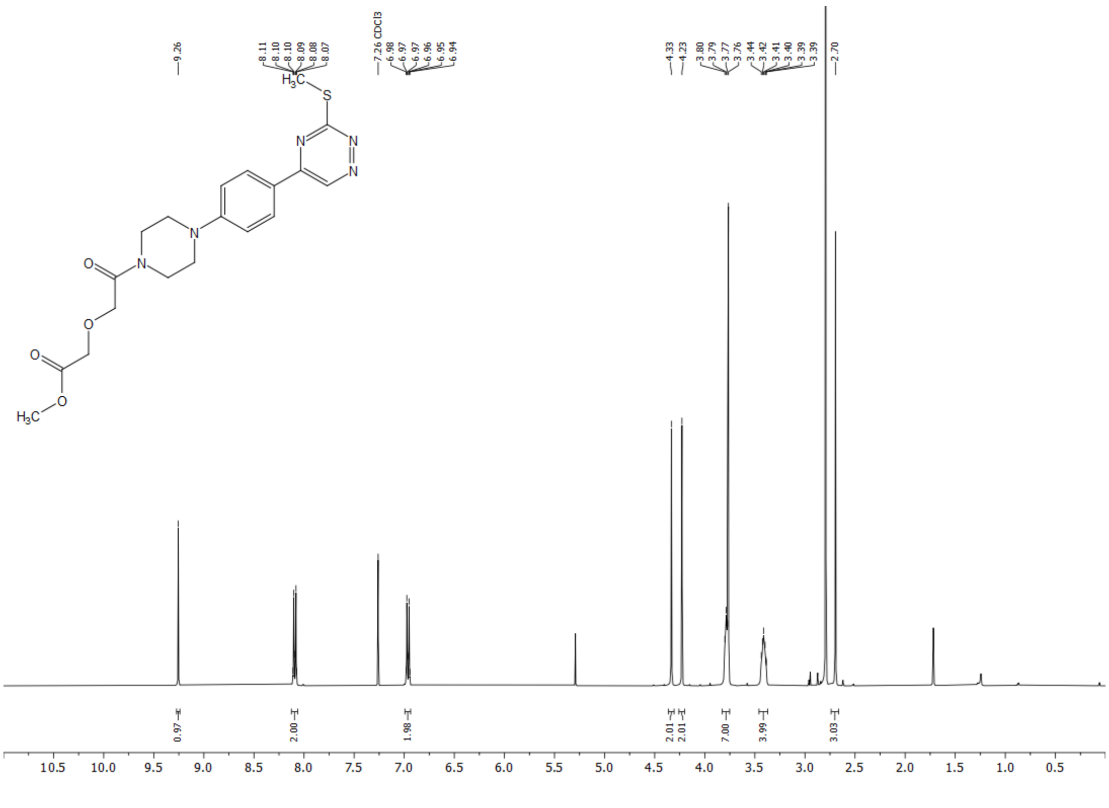

SMeTrz6 ^{13}C NMR (101 MHz, $CDCl_3$)

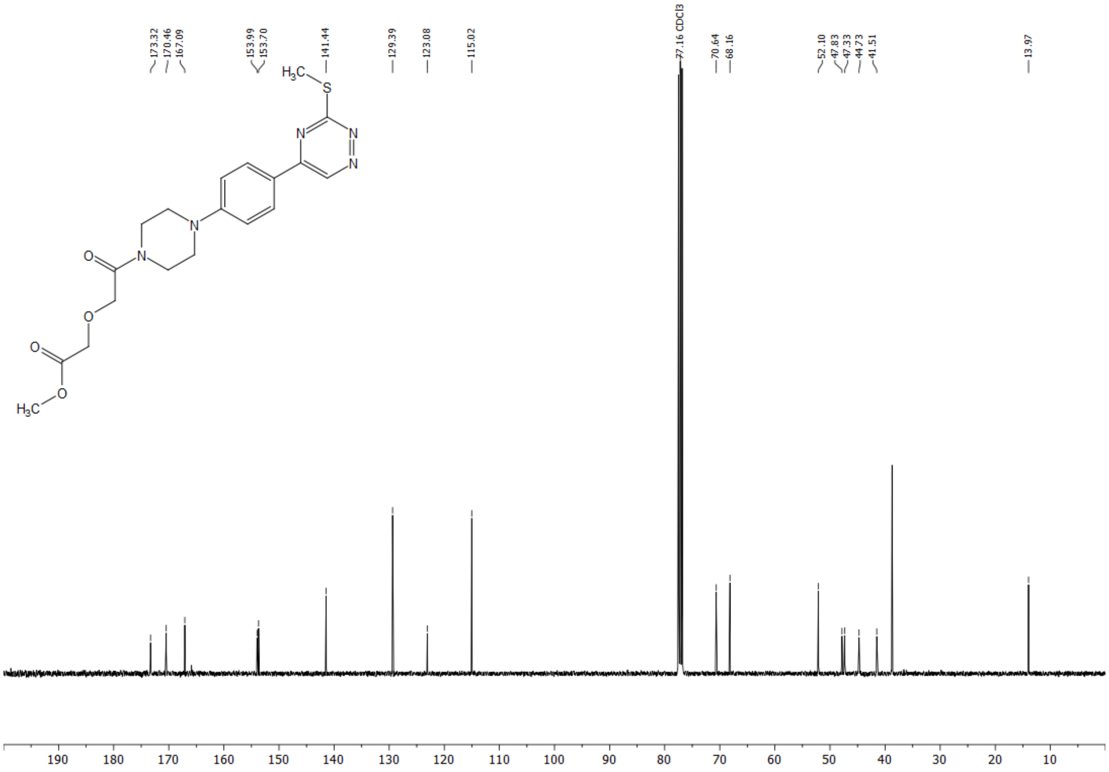

SMeTrz^{+}2 ^{1}H NMR (400 MHz, CD_3CN)

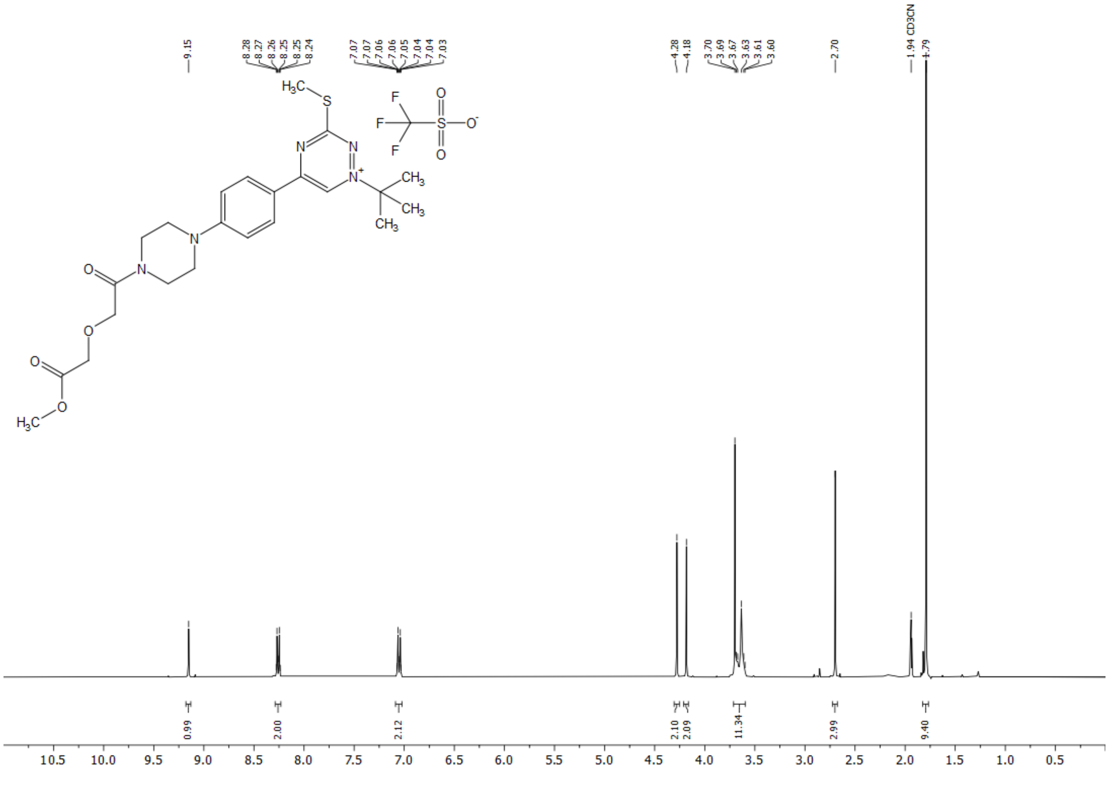

SMeTrz$^+$2 ^{19}F NMR (376 MHz, CD_3CN)

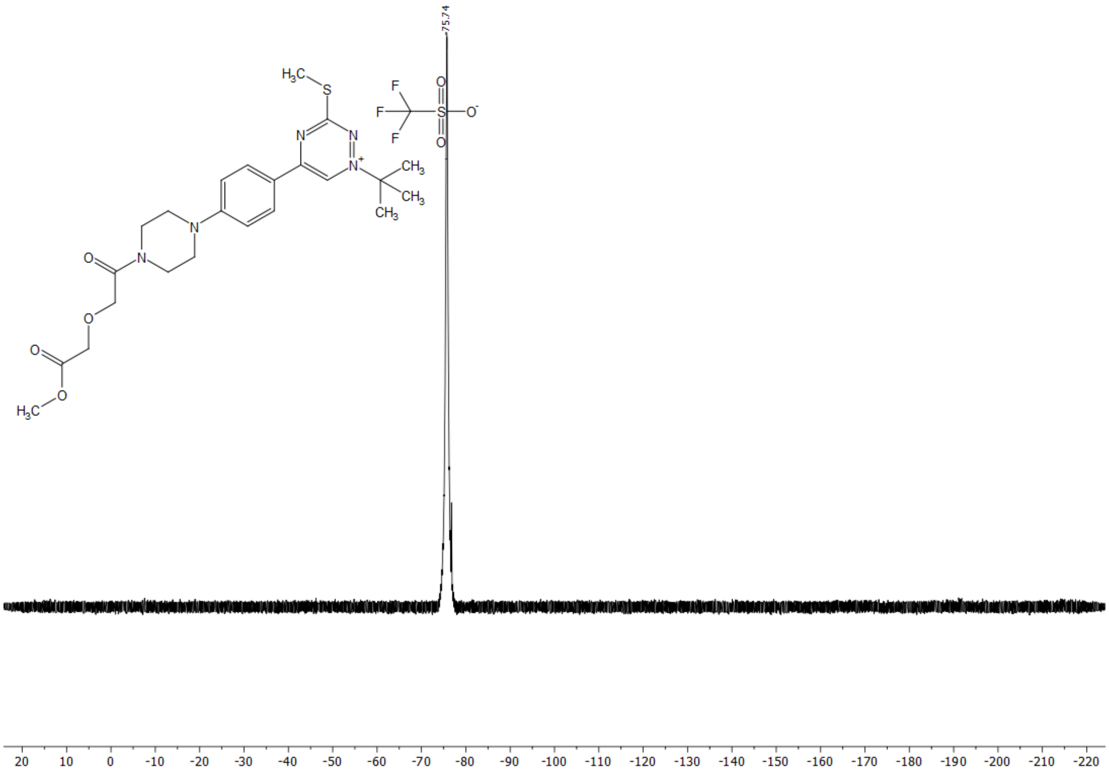

SMeTrz$^+$2 ^{13}C NMR (101 MHz, CD_3CN)

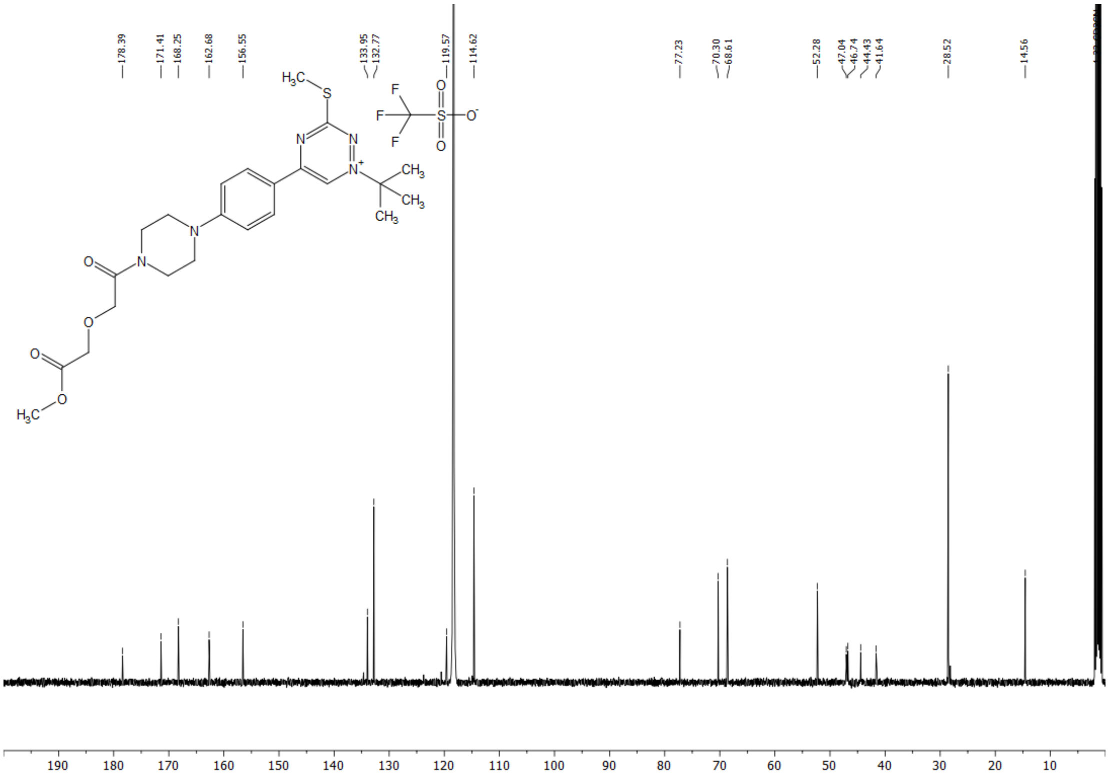

Trz^{+}2 ^{1}H NMR (400 MHz, CD_3CN)

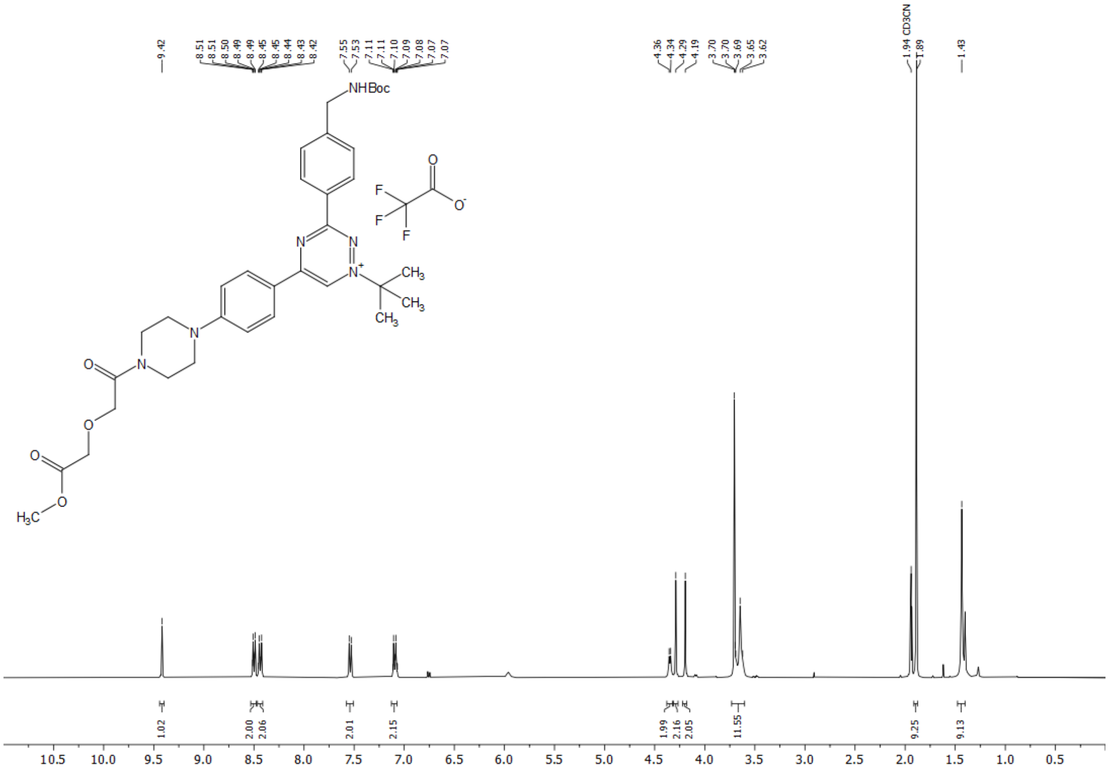

Trz^{+}2 ^{19}F NMR (376 MHz, CD_3CN)

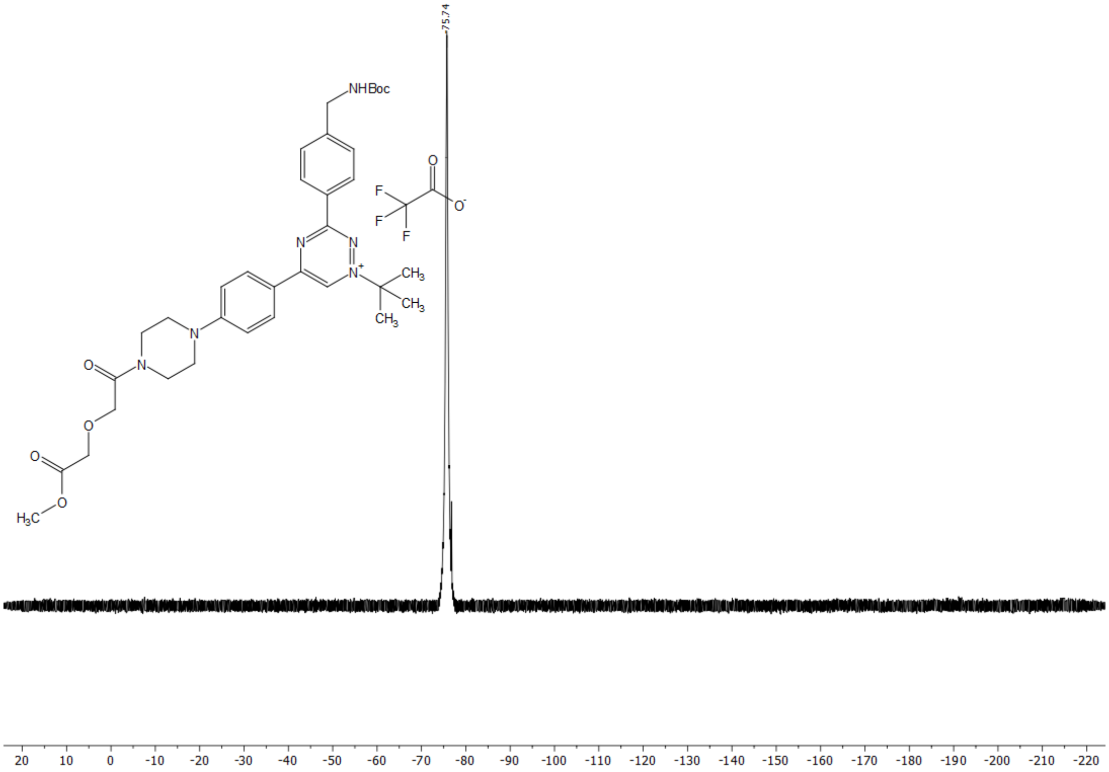

Trz⁺2 ^{13}C NMR (101 MHz, CD_3CN)

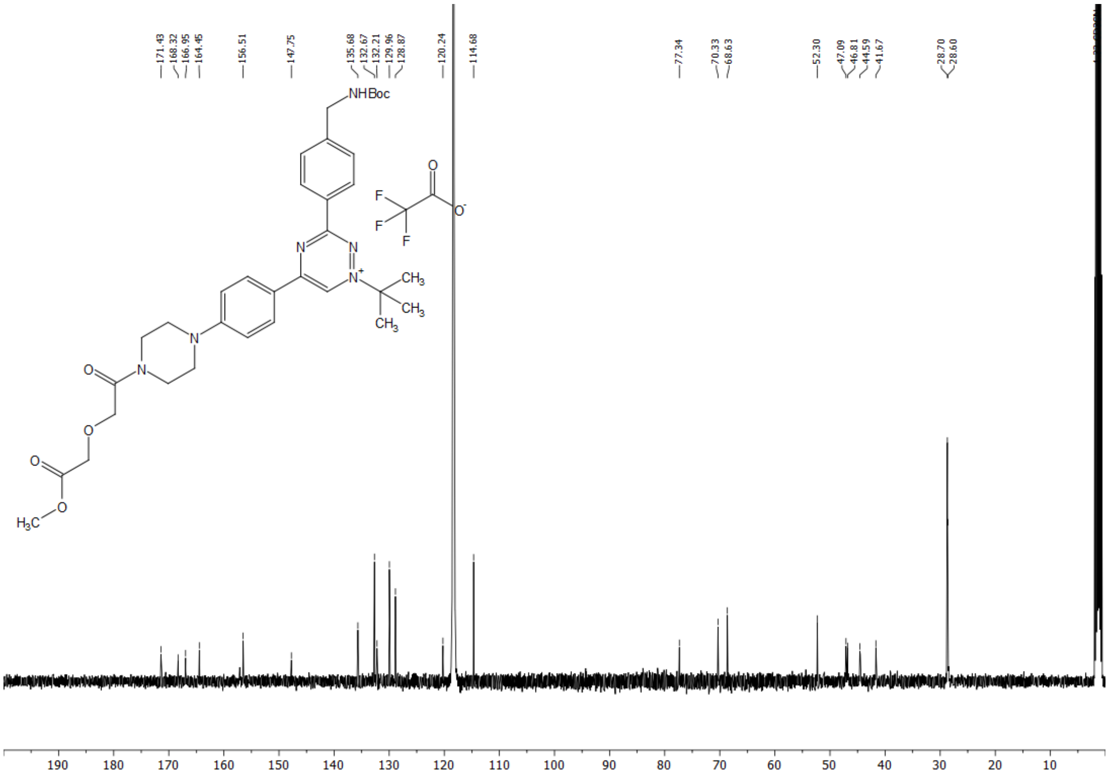

Trz⁺3 ^{1}H NMR (400 MHz, DMSO-d_6)

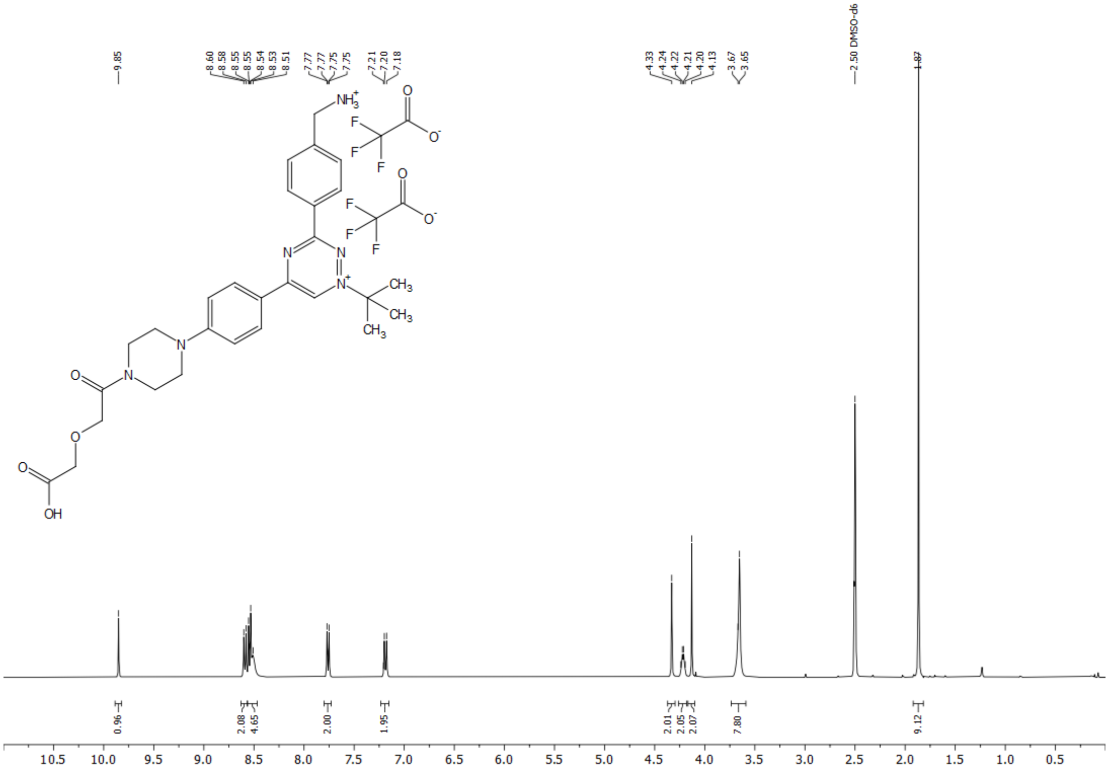

Trz⁺3 ^{19}F NMR (376 MHz, DMSO-*d₆*)

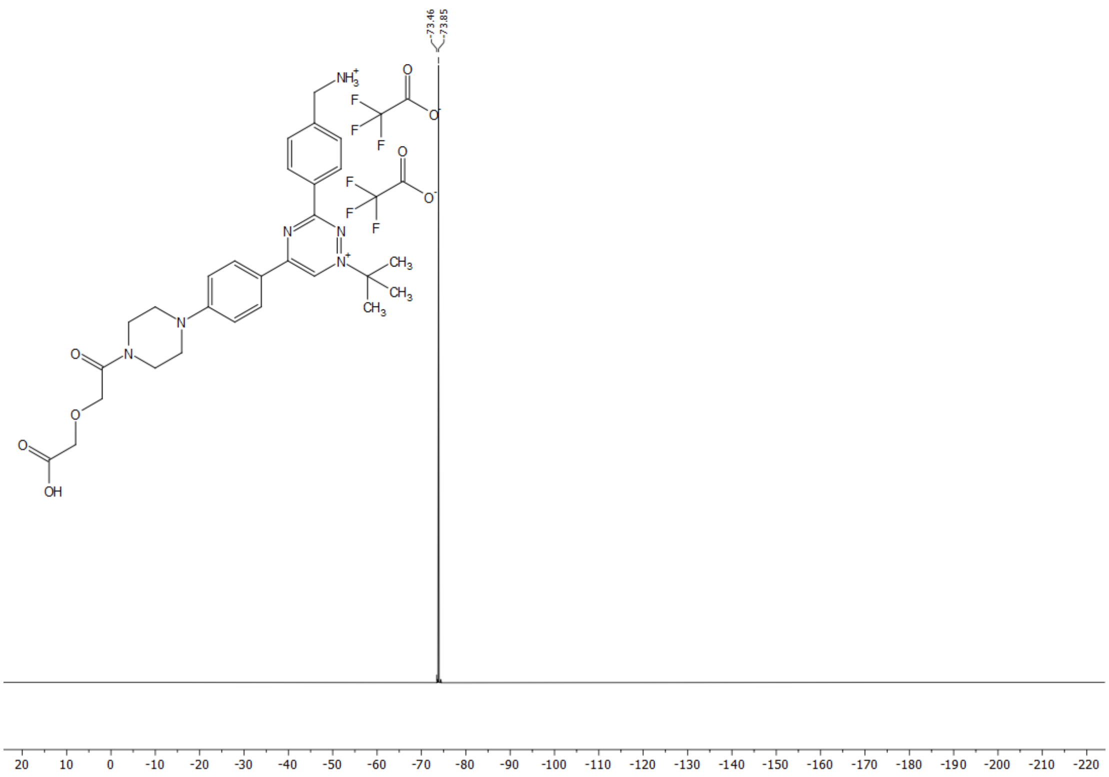

Trz⁺3 ^{13}C NMR (101 MHz, DMSO-*d₆*))

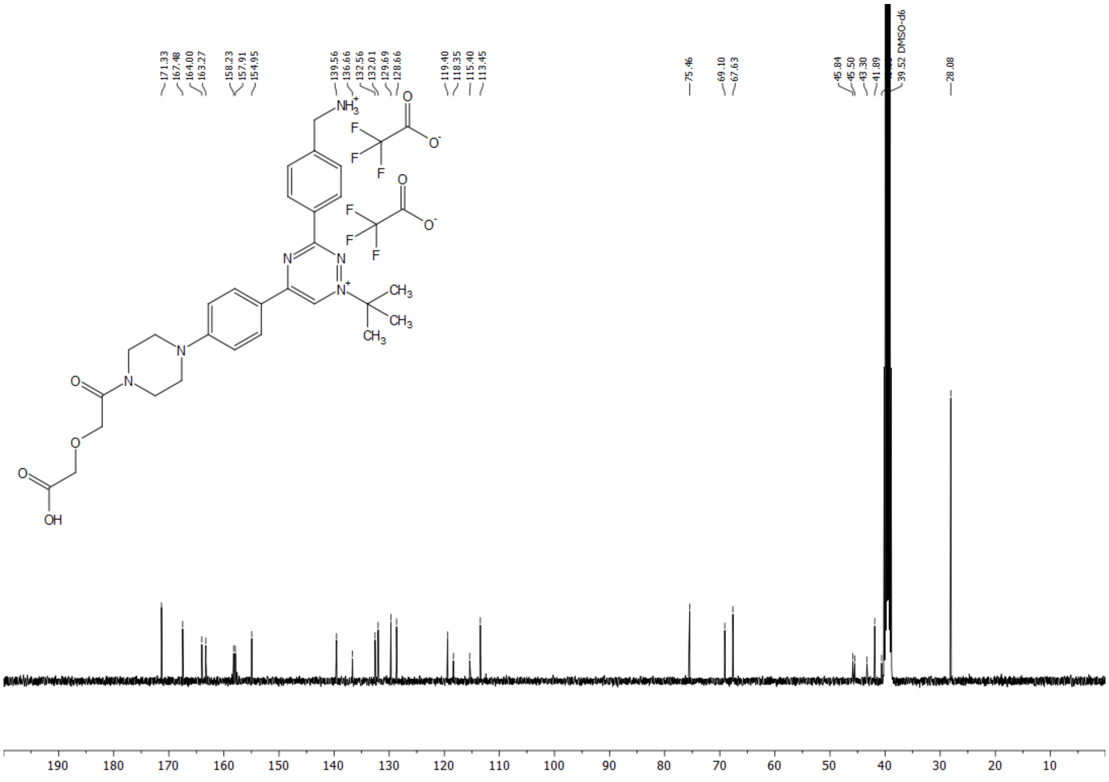

Trz⁺Coum2 ^{1}H NMR (400 MHz, CD_3CN)

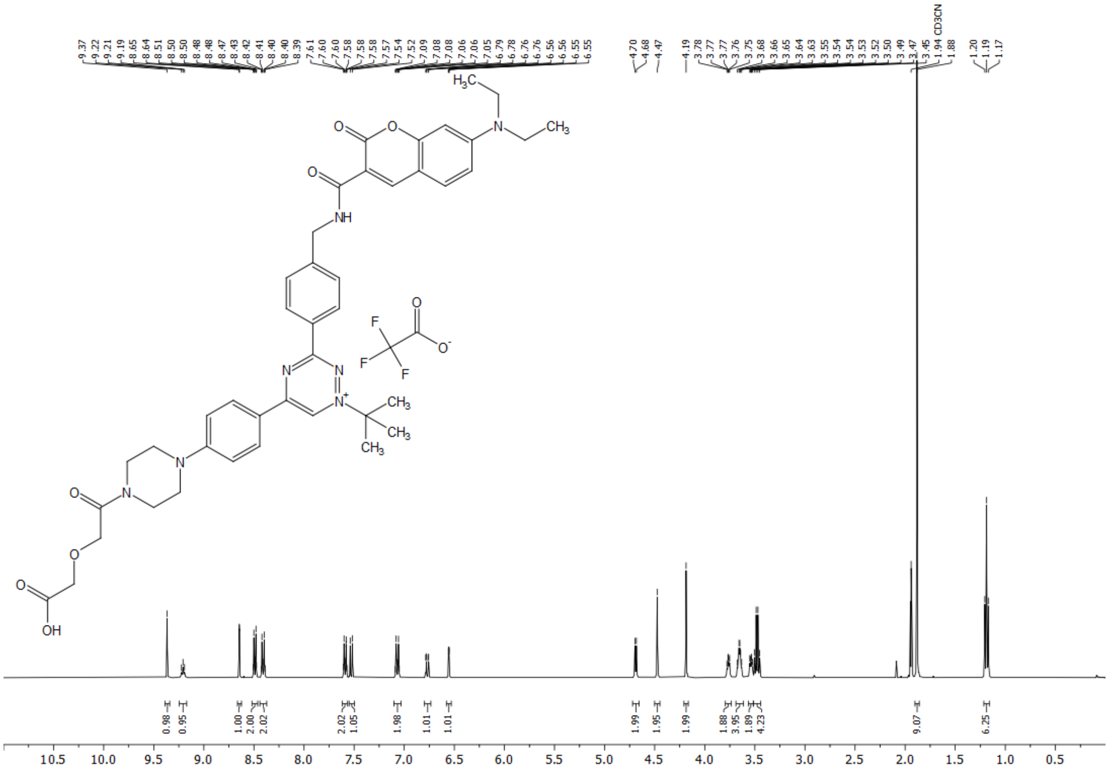

Trz⁺Coum2 ^{19}F NMR (376 MHz, CD_3CN)

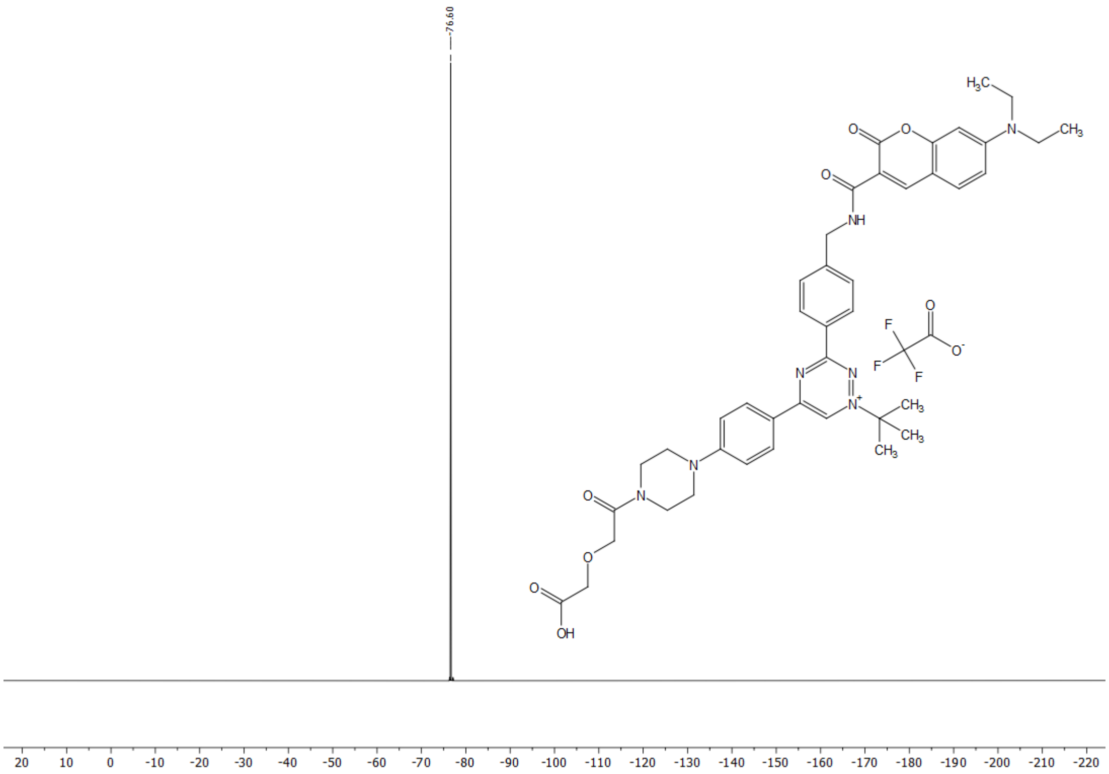

Trz⁺Coum2 ^{13}C NMR (126 MHz, CD_3CN)

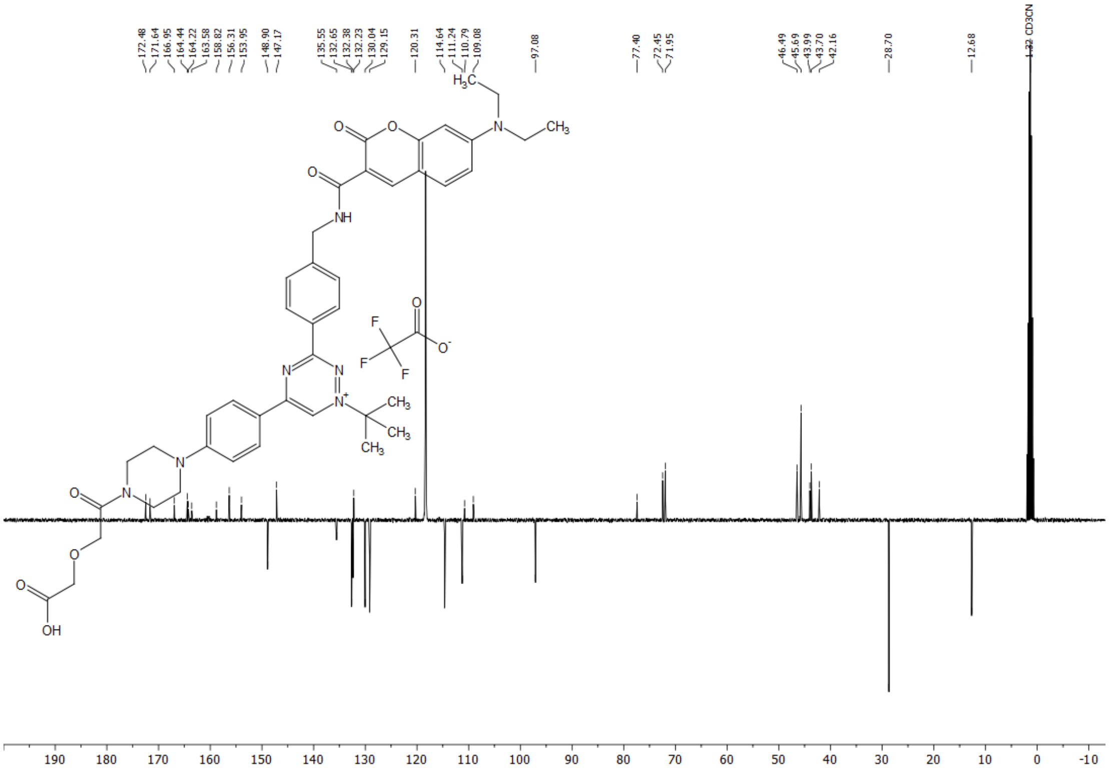

HaloTrz⁺Coum ^{1}H NMR (400 MHz, CD_3CN)

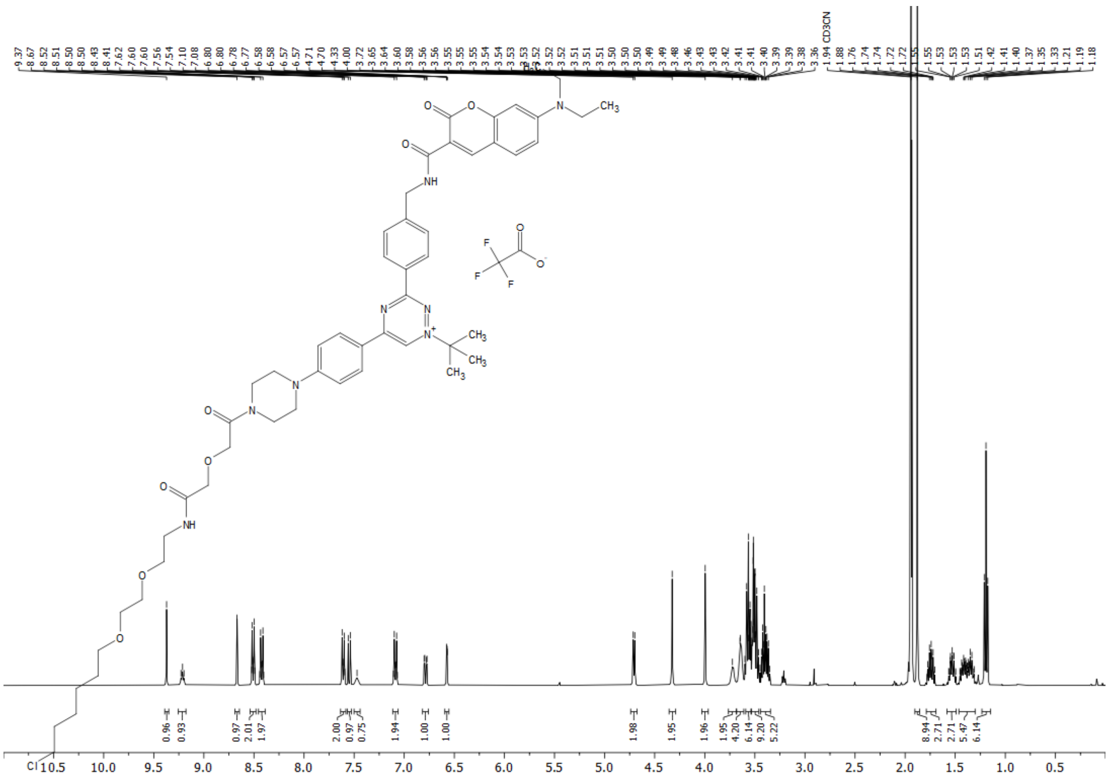

HaloTrz$^+$Coum ^{19}F NMR (376 MHz, CD_3CN)

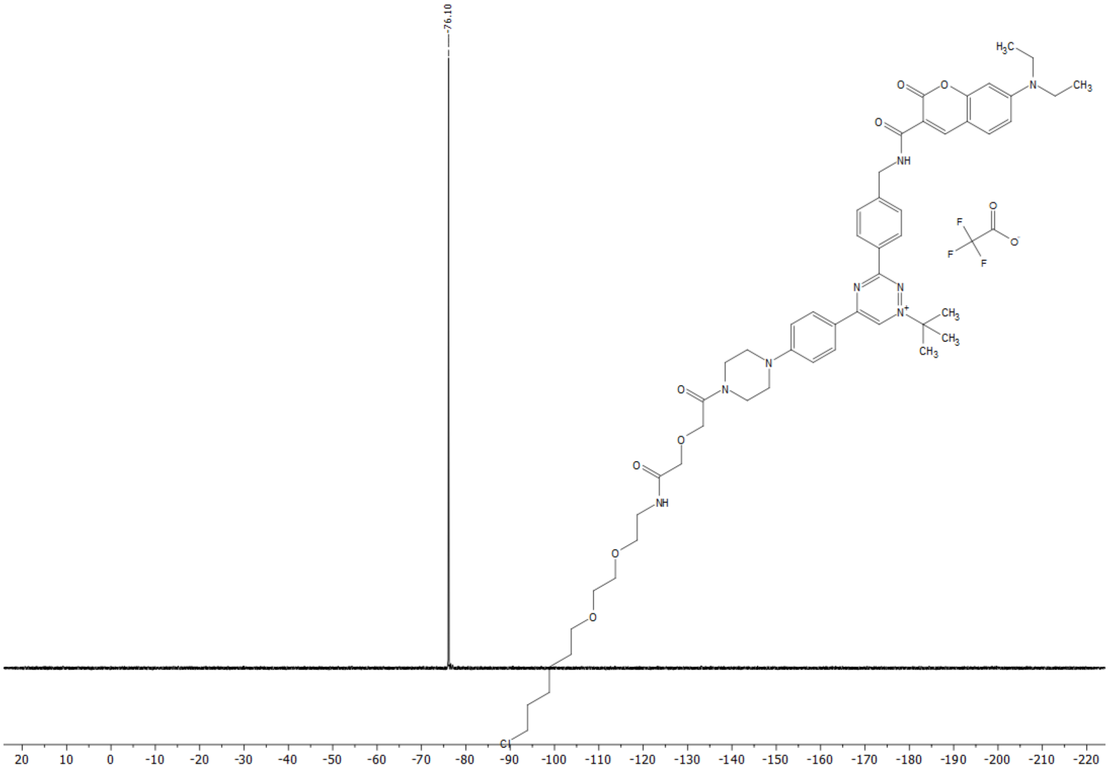

BrPD2 ^{1}H NMR (401 MHz, DMSO-d_6)

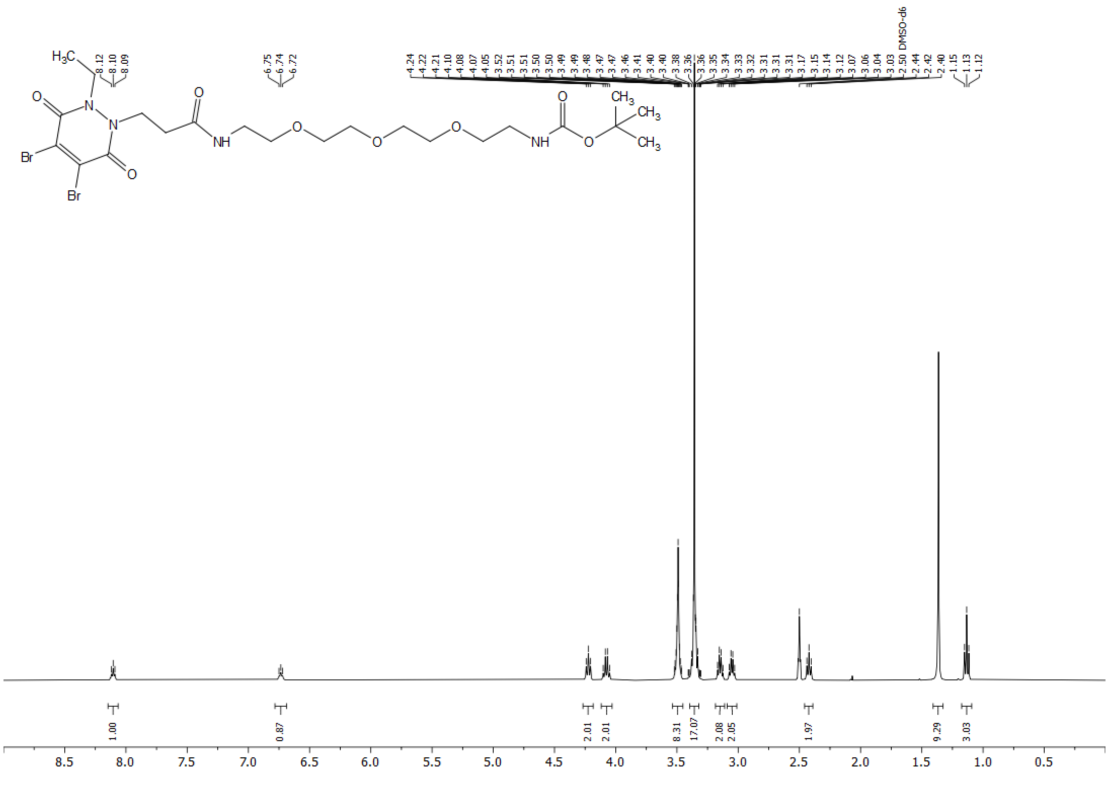

BrPD2 ^{13}C NMR (101 MHz, DMSO-d_6)

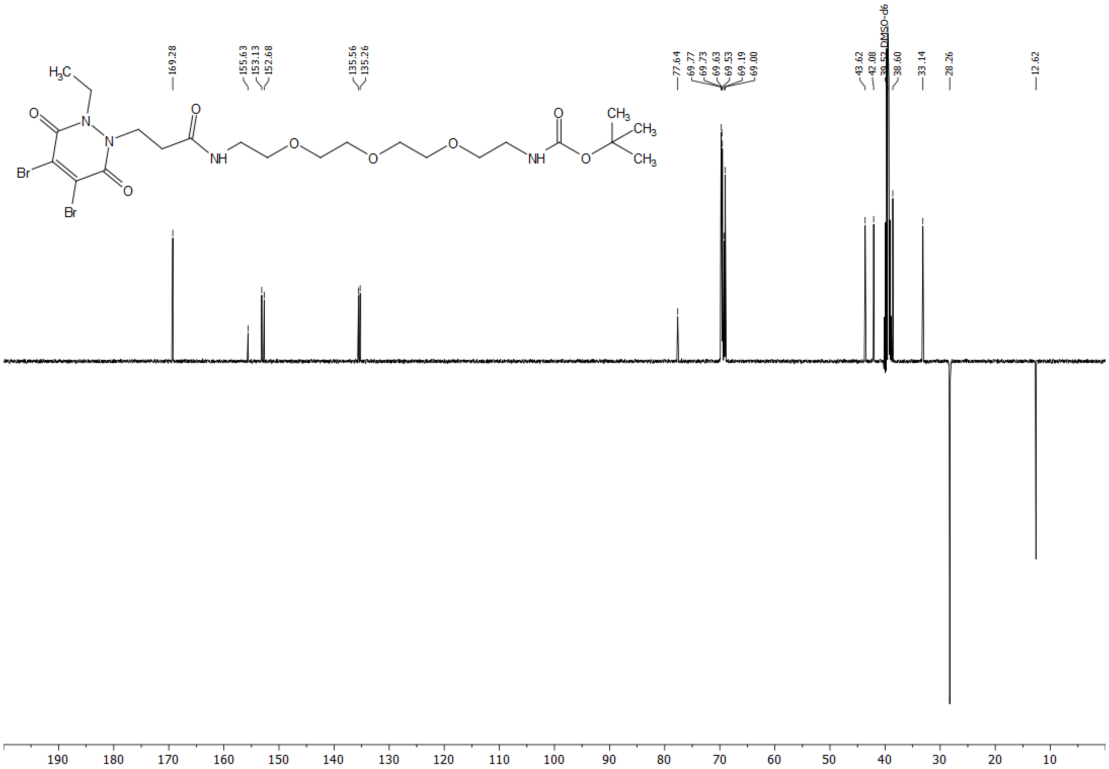

BrPD3 ^{1}H NMR (401 MHz, DMSO-d_6)

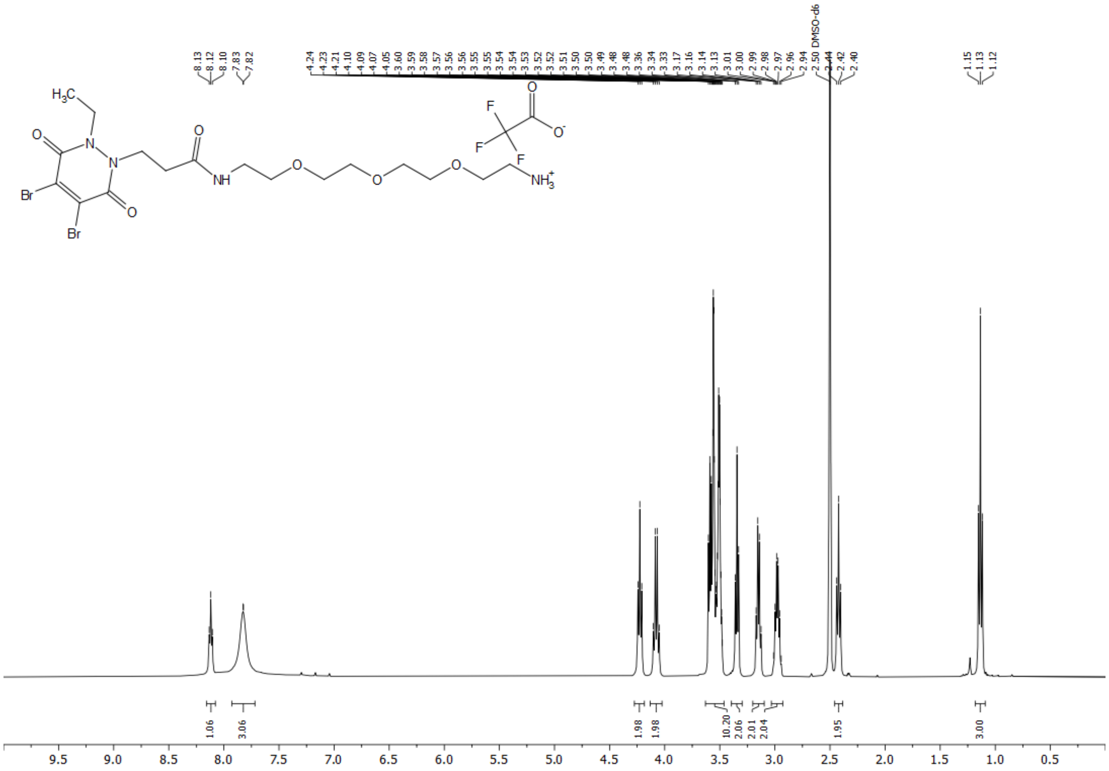

BrPD3 ^{19}F NMR (376 MHz, DMSO-d_6)

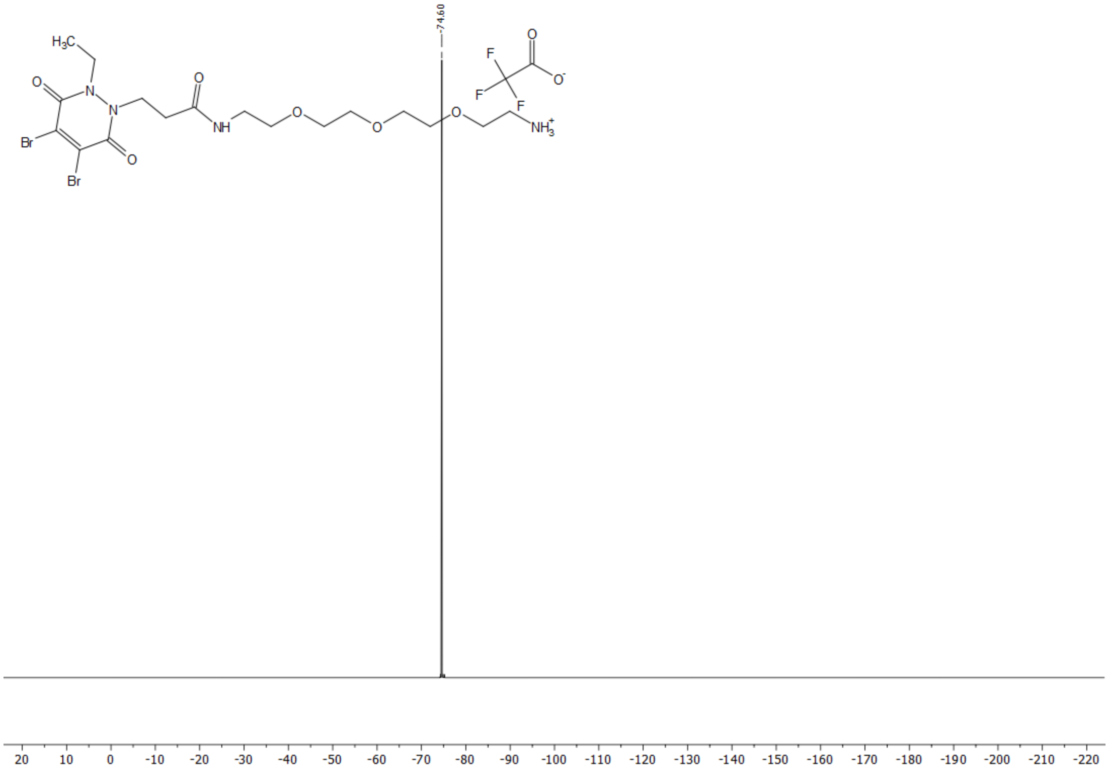

BrPD3 ^{13}C NMR (101 MHz, DMSO-d_6)

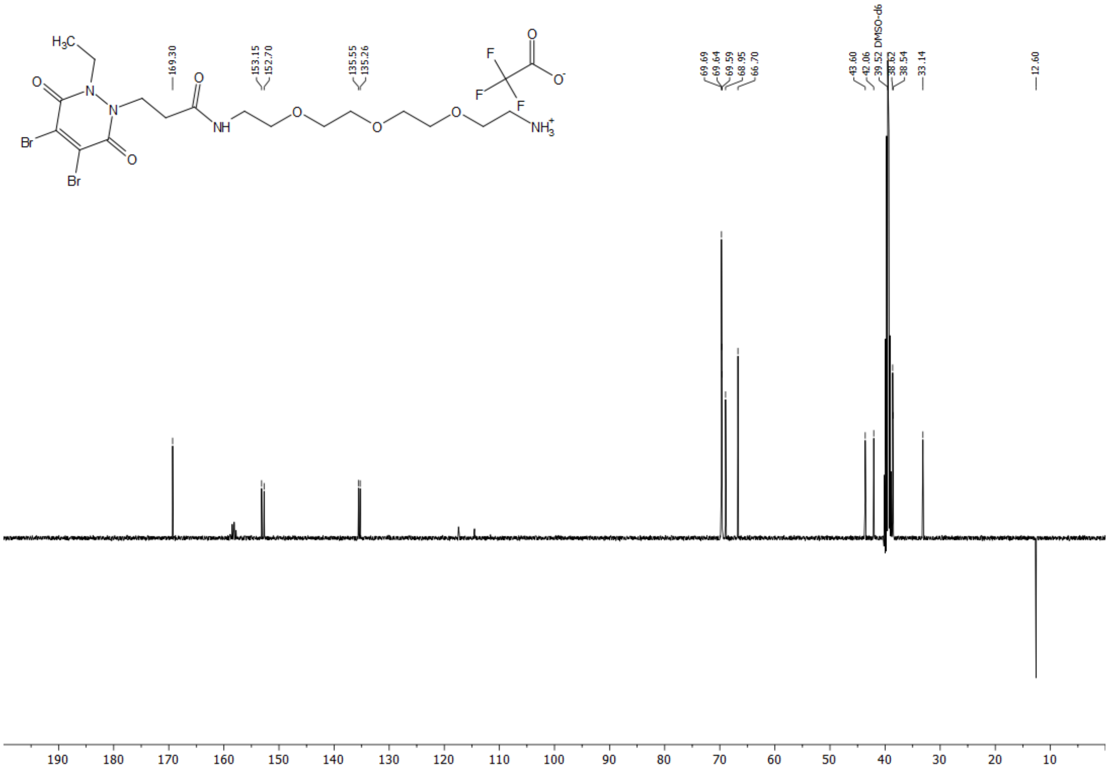

BrPD4 ^{1}H NMR (401 MHz, DMSO-d_6)

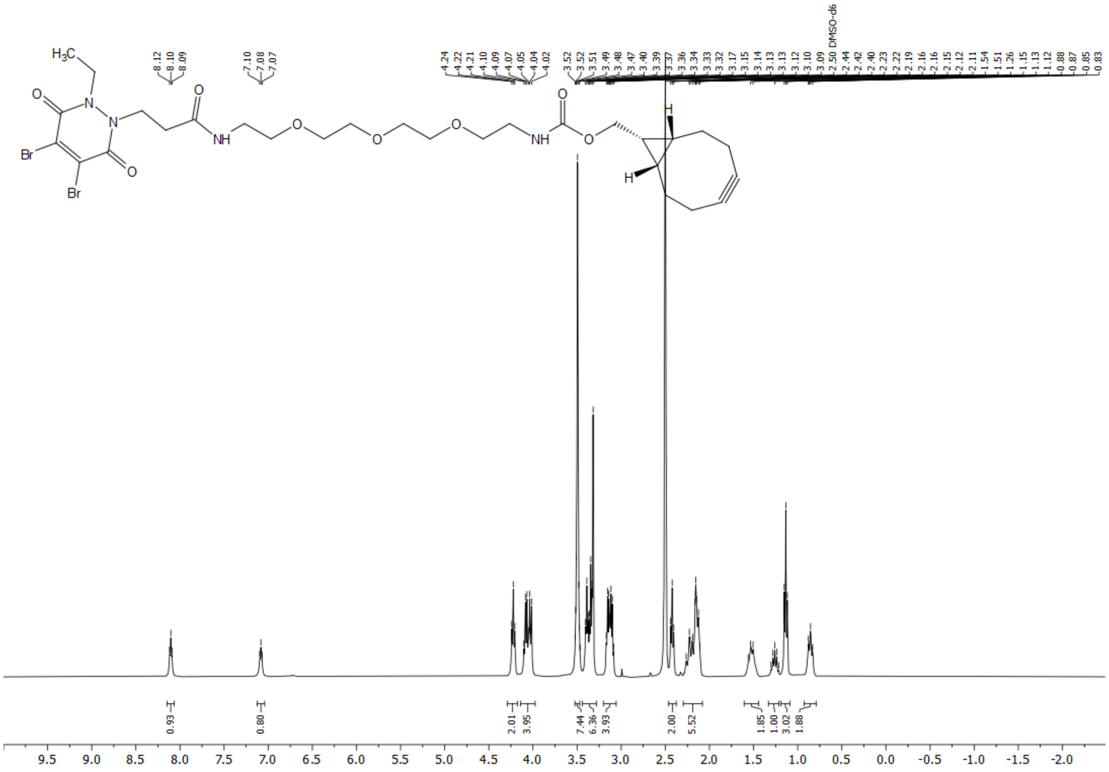

BrPD4 ^{13}C NMR (101 MHz, DMSO-d_6)

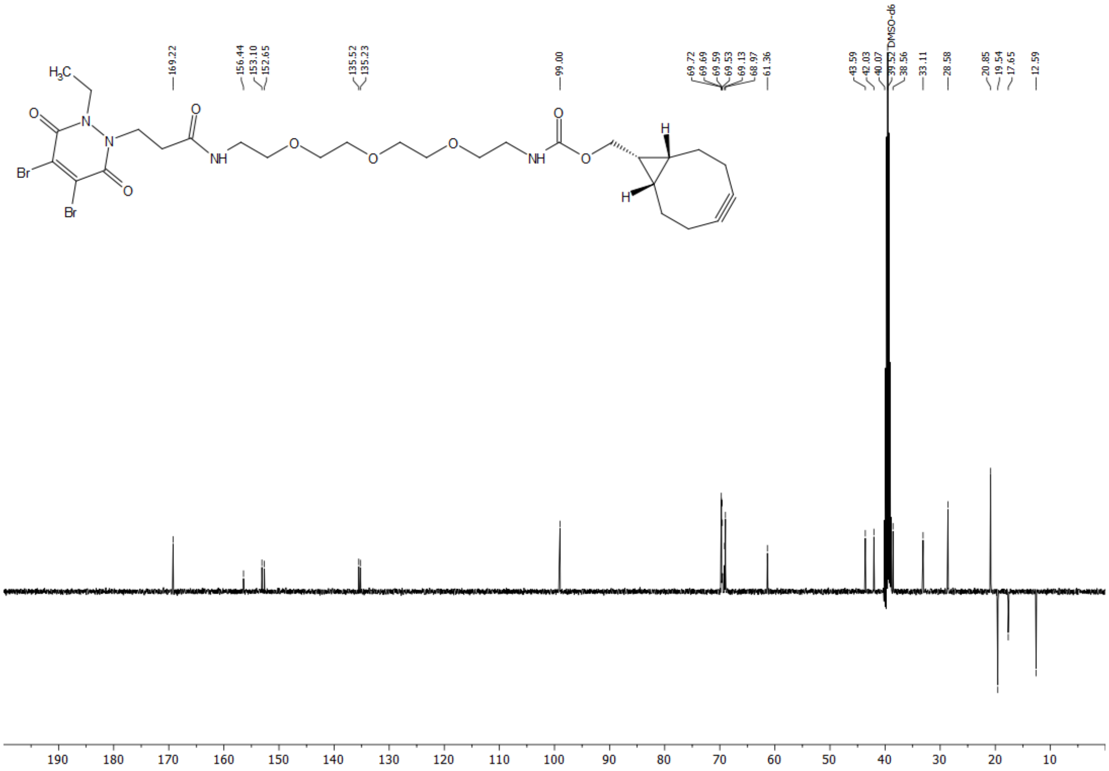

Dasatinib-BCN ^{1}H NMR (401 MHz, MeOD)

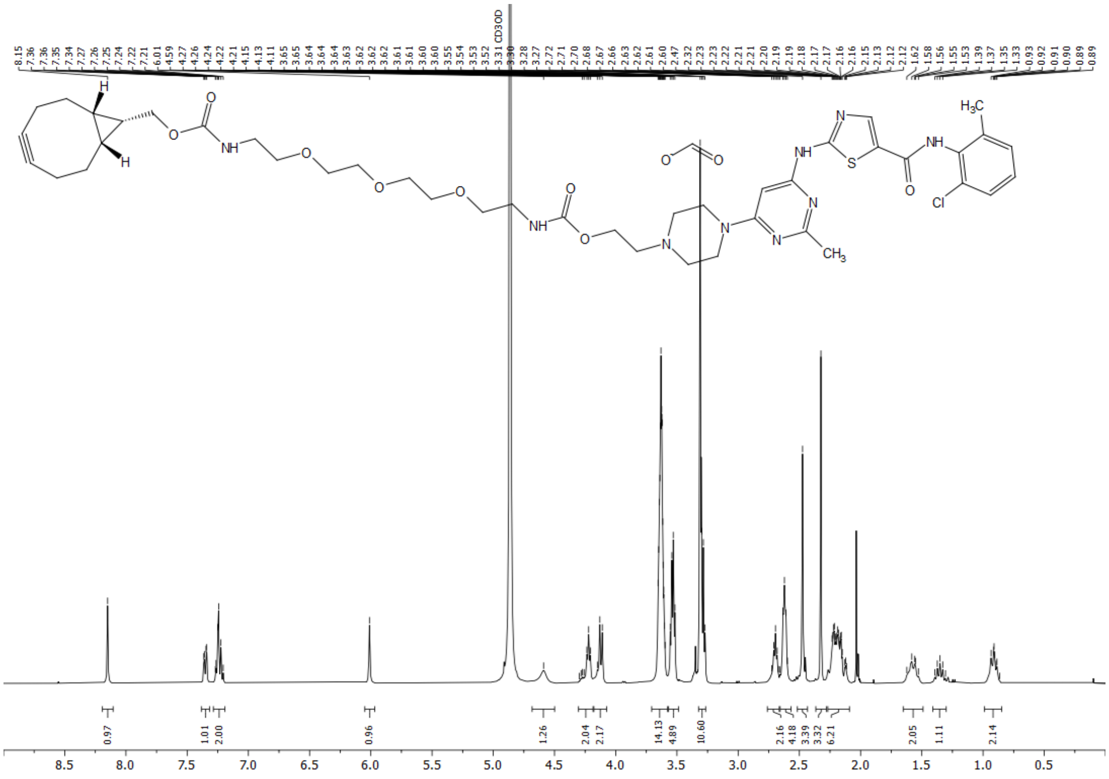

Dasatinib-BCN ^{13}C NMR (101 MHz, MeOD)

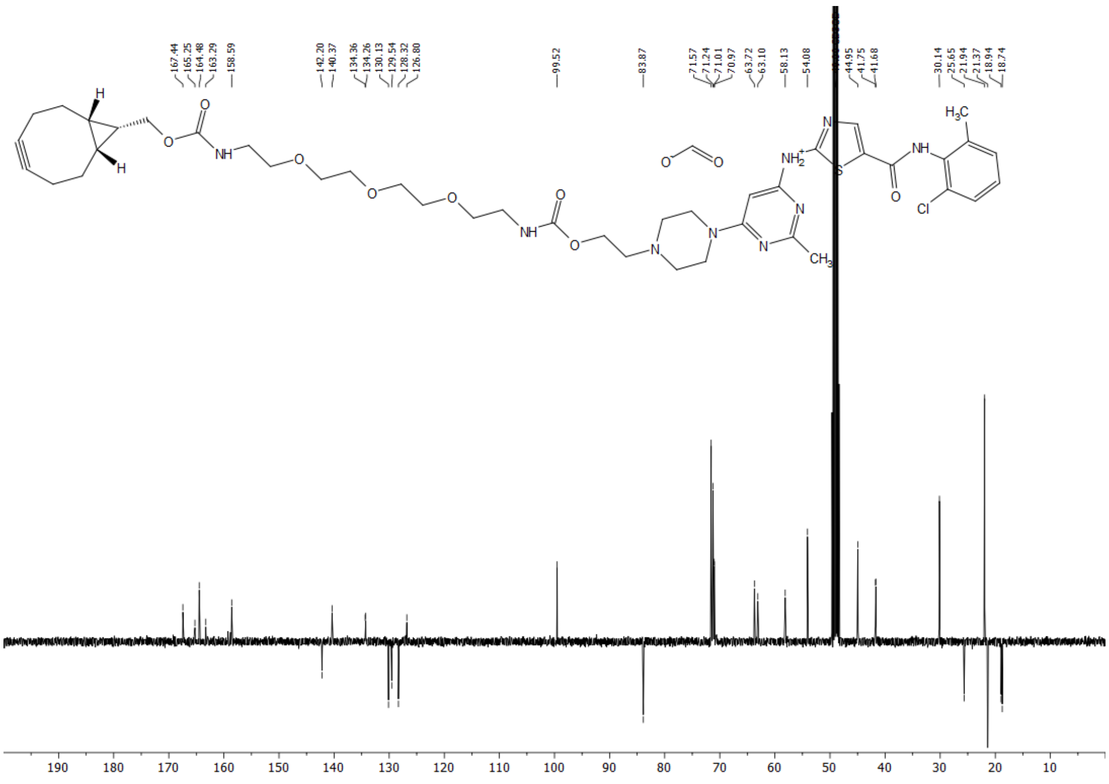

Geldanamycin-BCN ^1H NMR (401 MHz, $CDCl_3$)

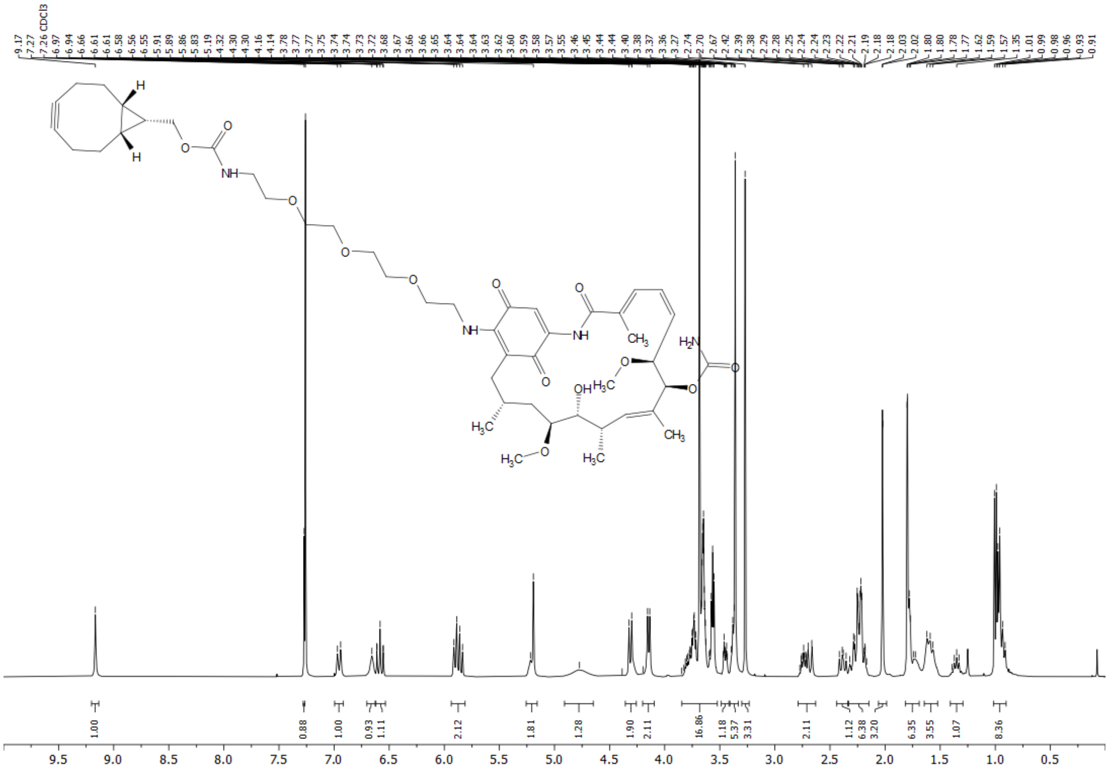

Supplement: Supplementary file 2 [file ja5c17428_si_003.pdf]
